# Simultaneous entry as an adaptation to virulence in a novel satellite-helper system infecting *Streptomyces* species

**DOI:** 10.1038/s41396-023-01548-0

**Published:** 2023-10-31

**Authors:** Tagide deCarvalho, Elia Mascolo, Steven M. Caruso, Júlia López-Pérez, Kathleen Weston-Hafer, Christopher Shaffer, Ivan Erill

**Affiliations:** 1 Keith R. Porter Imaging Facility, College of Natural and Mathematical Sciences, University of Maryland Baltimore County, Baltimore, MD USA; 2 Department of Biological Sciences, University of Maryland Baltimore County, Baltimore, MD USA; 3 Departament de Genètica i Microbiologia, Universitat Autònoma de Barcelona, Bellaterra, Spain; 4 Department of Biology, Washington University in St. Louis, St. Louis, MO USA; 5 Departament d’Enginyeria de la Informació i de les Comunicacions, Universitat Autònoma de Barcelona, Bellaterra, Spain

**Keywords:** Bacteriophages, Virology

## Abstract

Satellites are mobile genetic elements that are dependent upon the replication machinery of their helper viruses. Bacteriophages have provided many examples of satellite nucleic acids that utilize their helper morphogenic genes for propagation. Here we describe two novel satellite-helper phage systems, Mulch and Flayer, that infect *Streptomyces* species. The satellites in these systems encode for encapsidation machinery but have an absence of key replication genes, thus providing the first example of bacteriophage satellite viruses. We also show that codon usage of the satellites matches the tRNA gene content of the helpers. The satellite in one of these systems, Flayer, does not appear to integrate into the host genome, which represents the first example of a virulent satellite phage. The Flayer satellite has a unique tail adaptation that allows it to attach to its helper for simultaneous co-infection. These findings demonstrate an ever-increasing array of satellite strategies for genetic dependence on their helpers in the evolutionary arms race between satellite and helper phages.

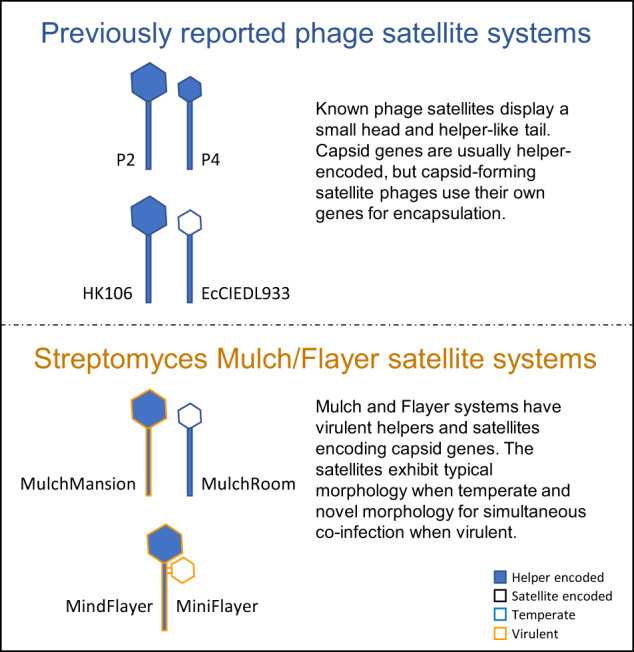

## Introduction

The virosphere contains many instances of virus-like mobile genetic elements (MGEs). Satellites are virus-like MGEs that depend on another virus for replication, and they are classified on the basis of this dependence [1]. Satellite viruses are dependent on helper viruses for genome replication, whereas satellite nucleic acids depend on helper viruses for transmission via encapsidation using helper-derived capsids [2]. No satellite viruses infecting bacterial hosts have been reported to date, but several families of satellite nucleic acids have been described and are commonly referred to as phage satellites [3].

Bacteriophage systems provided some of the first examples of satellite nucleic acids [4]. Enterobacteria phage P4 infects *Escherichia coli* cells and can integrate in the bacterial chromosome or exist as a plasmid, but requires the morphogenic genes of a co-infecting Enterobacteria phage P2 in order to encapsulate and lyse the cell [5]. Many other satellite nucleic acids have been described infecting a diverse range of bacterial hosts. Like P4-like satellites, phage-inducible chromosomal islands (PICIs) and PICI-like elements (PLEs) are widely disseminated satellites that reside in the bacterial chromosome and depend on helper viruses for encapsidation and lysis [6–8]. These satellite nucleic acids often encode genes that confer adaptive advantage to their bacterial host and typically interfere with helper phage replication [6]. Satellites associated with lytic phages, such as the *Vibrio cholerae* PLE, completely restrict production of their helper phage, effectively functioning as abortive infection systems [9].

All known phage satellites integrate into the host chromosome and replicate using satellite-encoded replicases [8]. Phage satellites use several strategies to divert encapsidation from helper phages. Enterobacteria phage P4, for instance, encodes a scaffolding protein (Sid) that redirects P2 capsid assembly, generating smaller capsids that cannot physically accommodate the P2 genome. P4 DNA is then packaged into the smaller capsids by the P2 terminase, which recognizes *cos* sites in the P4 chromosome [10]. Prototypical PICIs, like the staphylococcal pathogenicity islands (SaPIs), also use scaffolding proteins to generate smaller capsids, but display different packaging strategies. Some SaPIs contain *cos* sites and make direct use of the helper’s terminase for packaging, relying primarily on size exclusion to prioritize their preferential packaging [11]. Other SaPIs encode a small terminase subunit (TerS) to redirect packaging to the SaPI-specific *pac* sites and an interference protein to inactivate the phage TerS [3]. Proteobacterial PICIs encode a protein (Rpp) that binds the phage TerS, promoting its specific recognition of PICI *cos* sites [12].

Recently, a new class of PICI has been reported [13]. In contrast with previously described PICIs, capsid-forming PICIs (cf-PICIs) encode all the functional components for capsid formation (major capsid protein, head maturation protease, portal protein and head-tail connectors) as well as packaging (large and small terminase subunits and HNH endonuclease). These PICIs, which are widely distributed across *Bacteria*, therefore depend only on their helper phage for the production of tails [13].

Here, we report the isolation and characterization of a novel group of satellite phages infecting *Streptomyces* species that encode a full repertoire of capsid formation and packaging genes. These satellites present no significant homology with canonical or capsid-forming PICIs, PLEs or P4-elements, and possess several distinctive features: they parasitize virulent *Streptomyces* phages, their genomes contain direct terminal repeats (DTRs) and they present anomalously low %GC content matching their helper’s. Furthermore, our results indicate that loss of the chromosomal integration module in one of these satellites has been compensated by satellite acquisition of specialized tail components that appear to attach to the helper phage to facilitate co-infection via simultaneous entry into the host. To our knowledge, this is the first description of a virulent satellite associated with a virulent helper.

## Methods

### Isolation, characterization, and DNA sequencing

The MulchMansion/MulchRoom and the MindFlayer/MiniFlayer systems were isolated, respectively, from soil samples collected in St. Louis, MO (USA) and Poolesville, MD (USA), using previously reported phage isolation methods [14]. Soil samples were first suspended in phage buffer (10 mM Tris pH7.5, 10 mM MgSO4, 1 mM CaCl2, 68.5 mM NaCl). The resulting suspensions were spun to pellet soil and the supernatant filtered using a ø0.22 μm filter. The filtrate was then added to tryptic soy (TS) soft agar (BD, Sparks, MD, USA) with *Streptomyces lividans* JI 1326 (MulchMansion/MulchRoom) or *Streptomyces mirabilis* NRRL B-2400 (MindFlayer/MiniFlayer) and overlayed onto nutrient agar plates (BD Difco, Sparks, MD) supplemented with 10 mM MgCl2, 8 mM Ca(NO3)2 and 0.5% glucose (NA+). Plates were incubated at 30 °C for 1 to 2 days. A minimum of three rounds of plaque purification was carried out as previously described [14]. In short, isolated plaques were picked into phage buffer and serially diluted, then 10 µL of each dilution was combined with a 48 h culture of host, incubated 10 min at room temperature, combined with TS soft agar and plated on NA+ plates.

Crude stock lysate was harvested as described before [14] from plates demonstrating near-confluent lysis of *S. mirabilis* post infection. Plates were flooded with 5–8 mL phage buffer. The buffer (MulchMansion/MulchRoom) or buffer and soft agar overlay (MindFlayer/MiniFlayer) was collected and centrifuged 20 min at 2,500x *g*, and the supernatant passed through a ø0.22 µm filter. To screen for potential lysogens, MindFlayer crude lysate was serially diluted and the dilutions spotted on a freshly prepared plate of *S. mirabilis* and incubated for seven days at 30 °C.

DNA for the MindFlayer/MiniFlayer and MulchMansion/MulchRoom systems was isolated using the Promega Wizard DNA purification system on freshly prepared high-titer lysates, as reported previously [15]. Sequencing of MulchMansion/MulchRoom was performed by the McDonnell Genome Institute at Washington University with a NovaSeq 6000 (Illumina, USA) using High-Throughput Library Preparation Kit Standard PCR Amp Module (KAPA Biosystems, Boston, USA) and 150 × 2 reads. Sequencing of MindFlayer/MiniFlayer was completed by the Pittsburgh Bacteriophage Institute with the MiSeq (Illumina; v3 reagents) sequencing platform using the NEB Ultra II Library Kit and 150-base single-end reads. For all assemblies, raw sequencing reads were assembled using Newbler v2.9 or CLC Genomics Workbench NGS de novo assembler v6 with default settings. Genome completeness and termini were determined using Consed v. 29 [16, 17].

### Genome assembly and annotation

Genome annotation was performed using DNA Master v5.23.6 [18], using the embedded Glimmer v.3.02b [19] and GeneMark v.4.28 [20] for protein-coding gene calling and Aragorn v1.2.41 for tRNA gene prediction [21]. Protein-coding gene calls were manually refined based on their proximity and directionality, the presence of putative ribosome binding sites and sequence similarity to previously annotated genes. Protein coding genes were functionally annotated through homology search with BLASTp [22] and HHPred v57c87 [23].

### Electron microscopy

The MindFlayer/MiniFlayer system was imaged on a Morgagni M268 Transmission Electron Microscope (FEI, Hillsboro, IL, USA) equipped with an Orius CCD camera (Gatan Inc., Pleasanton, CA, USA). 10 µL of crude lysate was placed on to 200 mesh formvar-covered, carbon-coated copper grids (EMS, Hatfield, PA, USA), incubated for 1 min, briefly rinsed with ultra-pure water, then stained with 2% uranyl acetate for 2 min. Grids were scanned to record the number and position of associated satellites for randomly selected helper virions, as well as images taken for analysis. Morphological measurements were taken in Fiji [24] and all values are expressed as mean ± standard deviation. For certain analyses noted in the results, we also used “picked” plaque samples that are prepared by placing 50 µL of phage buffer directly onto a plaque and incubating for 30 s before pipetting up the liquid, followed by staining methods described above for the crude lysate.

### Comparative genomics and phylogenetic analysis

Gene content similarity among phage genomes was computed using the PhagesDB service [25]. Genome map figures were generated with Easyfig [26], using an *e*-value threshold of 10^–10^ for tBLASTx and considering only hits above 25% identity. To perform phylogenetic inference, a set of phage genomes potentially related to bacteriophages MiniFlayer or MulchRoom was compiled (Table S1). The set was obtained through a combination of BLASTp and tBLASTn searches. tBLASTn was used to identify related phage genomes, by querying the NCBI GenBank database with all the proteins encoded by each satellite. The search was restricted to viruses (taxonomy ID 10239). Any genome containing at least one hit with E-value lower than 10^–10^ and query coverage larger than 75% was included (Tables S2, S3). To identify putatively related satellites integrated in bacterial chromosomes, BLASTp was performed against the proteomes of satellites reported in Ref. [8] using the same limiting thresholds as in the tBLASTn search (Tables S4, S5). The nucleotide sequences of satellites containing at least one hit were extracted from the GenBank genome records of the hosts (Table S6) and added to the set of potentially related phages. For reference, a small set of representatives of classic helper-satellite systems was also included (Table S7).

ViPhy [27] was used to obtain a phylogenetic distance matrix of phage genomes. We then performed agglomerative clustering using scikit-learn to produce a diverse set of 100 phages. A phylogenetic tree for these 100 phages was generated using the VICTOR web service [28]. Intergenomic distances were based on protein sequence distances, computed with the Genome-BLAST Distance Phylogeny (GBDP) method, using the settings recommended for prokaryotic viruses [28] and applying the *d*_*6*_ distance formula [29]. A balanced minimum evolution tree was produced with FASTME from the intergenomic distances [30], using 100 pseudo-bootstrap replicates to obtain branch support values. The phylogenetic tree was annotated and visualized with the iTOL web service [31]. Phage lifestyle (temperate/virulent) was predicted using the BACterioPHage LIfestyle Predictor (BACPHLIP) classifier [32]. The %GC content of phage genomes was computed using BioPython [33]. Lifestyle and %GC content were integrated for visualization in iTOL.

### Genome sequence processing and codon usage bias analysis

Codon usage adaptation to tRNA gene pools was estimated by computing the tRNA Adaptation Index (tAI), as described in Ref. [34] (https://github.com/ErillLab/SPA). The genome sequences of the BE cluster phages and their hosts were downloaded from NCBI (Table S10). Both tRNAscan and Aragorn were used to predict tRNAs in downloaded genomes [21, 35]. Results were filtered for duplicates. When the same tRNA was predicted by both tRNAscan and Aragorn (same anticodon, maximum difference in location of 10 bp), the Aragorn tRNA location was kept. For tRNA genes with a predicted CAT anticodon, the tRNAscan tRNA call was kept. Whenever a tRNA was undetermined by tRNAscan, the Aragorn tRNA call was kept [36].

All the code used in this work can be found in a dedicated GitHub repository (https://github.com/ErillLab/SPA).

## Results

### Identification of a *Streptomyces* satellite-helper system through DNA sequencing

*Streptomyces* phages MindFlayer/MiniFlayer and MulchMansion/MulchRoom were isolated from soil samples collected in Poolesville, MD and St. Louis, MO (USA) using, respectively, *S. mirabilis* and *S. lividans* as isolation hosts. After 48 h at 30 °C on their isolation host, MindFlayer/MiniFlayer formed clear plaques with diameters of 1 to 5 mm (Supplementary Fig. 1A). Small plaques were homogeneously round, whereas larger plaques presented irregular shapes. MulchMansion/MulchRoom formed small (ø0.1 mm), sometimes cloudy plaques (Supplementary Fig. 1B). Following DNA purification, sequencing was performed using the Illumina MiSeq sequencing platform. Read assembly of MindFlayer/MiniFlayer resulted in two well-defined contigs corresponding to *Streptomyces* phages MindFlayer (130,258 bp) and MiniFlayer (17,449 bp), with 73% of the 297,433 reads mapping to the smaller contig. Read assembly of MulchMansion/MulchRoom also yielded two well-defined contigs corresponding to *Streptomyces* phages MulchMansion (134,105 bp) and MulchRoom (14,787 bp), with 78% of the reads mapping to the smaller contig. In all cases, the assembled chromosomes were found to be linear with direct-terminal repeats (DTRs) (Table 1). Plaque purification was attempted for the MindFlayer/MiniFlayer system, but was only successful for the larger phage (MindFlayer) as confirmed by PCR. Based on the sequencing read asymmetry, consistent with known satellite-helper systems [7], and the inability to purify the smaller phages, these two groups of phages were tentatively considered satellite-helper systems, hereafter referred to as the Mulch (MulchMansion/MulchRoom) and Flayer (MindFlayer/MiniFlayer) systems.

**Table 1:** Main characteristics of assembled chromosomes for the Mulch and Flayer satellite-helper systems.

Phage	Sequence Read Archive identifier	Mapped reads	GenBank accession	Genome length (bp)	DTR length (bp)	%GC	Protein-coding genes	tRNA genes
MindFlayer	SRX20165773	80,293	MW291014	130,258	12,198	49.5%	234	42
MiniFlayer	SRX20165773	217,140	OQ357865	17,449	1,057	46.7%	26	0
MulchMansion	SRX20209287	34,337	MT897905	134,105	11,127	49.4%	236	41
MulchRoom	SRX20209287	120,071	OQ784832	14,787	1,609	51.0%	20	0

### Mulch and Flayer systems comprise similar helpers and highly diverged satellites

**Fig. 1:**
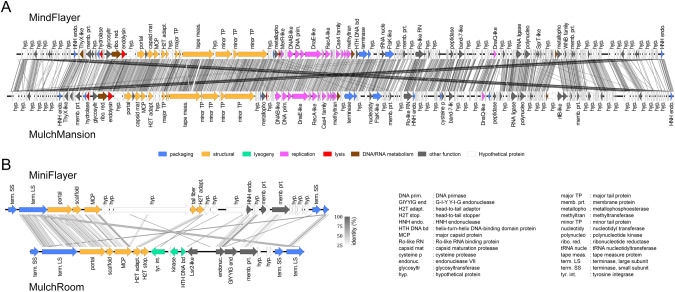
Comparative genome map of isolated phages. Genome organization of (**A**) the helper phages MindFlayer and MulchMansion and (**B**) the satellite phages MiniFlayer and MulchRoom. Coding sequences are colored according to their predicted function category. The percent identity of tBLASTx hits is displayed in gray scale.

Genome annotation of the Mulch and Flayer genomes revealed that the two helper phages, MindFlayer (MW291014.1) and MulchMansion (MT897905.1) encode ~230 protein coding genes, over 40 tRNA genes and one tmRNA gene. Based on gene content similarity, the Actinobacteriophage database [25] assigned these phages to its BE cluster. Phages in this cluster present siphoviral morphology and are known to contain genes for virion structure and assembly, DNA/RNA metabolism, DNA replication, lysis and DNA packaging. Based on multiple independent lysogeny spot tests and on the absence of lysogeny-related genes, BE phages are presumed to be virulent [37]. In agreement with this, no genes in MindFlayer or MulchMansion present homology to known integrases or immunity repressors and lysogeny spot tests performed on MindFlayer were negative. Like other BE phages, MindFlayer and MulchMansion have linear chromosomes with large (11–12 Kbp) direct terminal repeats (DTR). They present an average gene content similarity of 63.41% evenly distributed along their chromosomes with the notable exception of the DTRs (Fig. 1A).

The genomes of the two satellite phage genomes (MiniFlayer (OQ357865) and MulchRoom (OQ784832)) are also linear, with ~1 Kbp DTRs. These genomes encode, respectively, 26 and 20 protein-coding genes and no tRNA genes (Table 1). In contrast with the helper genomes, the two satellite genomes present weak amino acid sequence similarity (Fig. 1B). The main region of sequence similarity corresponds to the structural module, on the proximal end of both chromosomes, which contains small and large terminase subunits, the portal and major capsid proteins, as well as scaffolding protein and a head-to-tail adapter. Other elements showing significant sequence similarity include an HNH endonuclease and a predicted membrane protein on the distal end of the chromosomes. The MulchRoom genome also contains a lysogeny module, composed of a tyrosine integrase and a predicted helix-turn-helix (HTH) domain-containing protein matching the HTH domain of GntR family regulators, located in the central region of the chromosome together with genes encoding a histidine kinase and a Lsr2-like DNA bridging protein. The central region of the MiniFlayer genome does not contain a lysogenic module, but features instead a tail fiber protein and three hypothetical proteins that are absent in MulchRoom and could constitute an adsorption module. The MulchRoom genome also contains a predicted GIY-YIG endonuclease that is absent in MiniFlayer. In contrast with all known phage satellites, both genomes lack any genes showing homology to known primases.

### Mulch and Flayer systems display markedly different virion structures

**Fig. 2:**
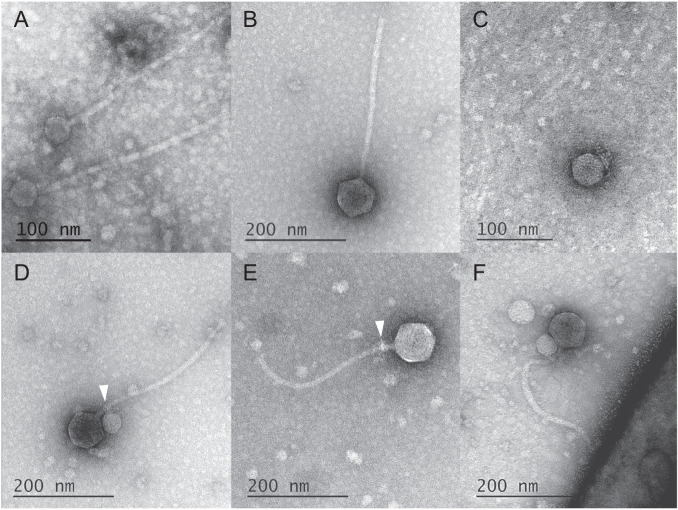
Representative TEM images of isolated phages. Representative TEM images of (**A**) MulchRoom; (**B**) MindFlayer; (**C**) MiniFlayer; (**D**) MiniFlayer adsorbed to MindFlayer neck; (**E**) MindFlayer neck proteins displaying residual MiniFlayer tail fibers; (**F**) MindFlayer/MiniFlayer adsorbed to *S. scabiei*. Arrowheads indicate attachment points.

Transmission electron microscopy (TEM) of MulchRoom revealed small capsids (43.7 ± 3.0 nm, *n* = 5) that were approximately half the size of the helper capsids (81.9 ± 0.5 nm, *n* = 2). Mulchroom also displayed long siphovirus-like tails (330.8 ± 3.8 nm, *n* = 3; Fig. 2A), similar to recently reported capsid forming PICIs [13]. MiniFlayer also showed relatively small capsids (42.5 ± 3.1 nm, *n* = 13) compared to the siphovirus helper (81.4 ± 2.8, *n* = 13, Fig. 2B; see Ref. [38] for additional TEM images); however MiniFlayer virions display short tail fibers reminiscent of podovirus morphology (Fig. 2C). To further characterize the unusual morphology of MiniFlayer, we undertook extensive TEM characterization of mixed MindFlayer/MiniFlayer lysates. Our analysis revealed consistent physical association between the two phages. In particular, we observed that MiniFlayer tail fibers absorb specifically to neck proteins of MindFlayer (Fig. 2D), which were not observed to be attached to any other location on the helper. In a high proportion of cases (79%, *n* = 49/62), MiniFlayer capsids were adjacent to the MindFlayer capsid (Fig. 2D, F), consistent with some form of capsid-to-capsid adsorption. We found that 56% of MindFlayer virions (*n* = 26/50) had MiniFlayer attachment.

We also analyzed picked plaque samples because it is a less disruptive process than lysate preparation, and often yields virions that are more intact and or adsorbed to the host. From the picked plaque sample, we observed a significantly higher amount of MindFlayers virions with attached MiniFlayer (80%, *n* = 40/50). Moreover, this high degree of MiniFlayer association is likely an underestimate due to the observation that some isolated helper virions showed evidence of prior MiniFlayer attachment in the form of residual tail fibers (Fig. 2E) from which the capsid had been broken. We also observed several instances of the helper virion adsorbed to the host bacteria with the satellite virion attached (Fig. 2F), but we found no instances of MiniFlayer directly attached to the host surface. Consistent with DNA sequencing data, where 78% of reads mapped to MiniFlayer, we found 86% of virions surveyed were MiniFlayer in the crude lysate (*n* = 107).

### Mulch and Flayer satellites are not directly related to their helpers or known helper-satellite systems

**Fig. 3:**
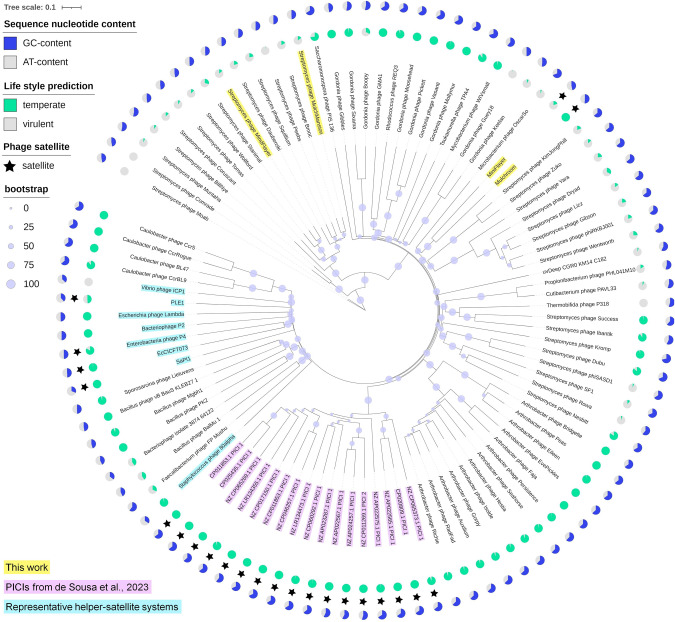
Phylogenetic analysis of helper and satellite phages. Whole-proteome phylogenetic tree of the selected phage genomes (Table S1), reconstructed using VICTOR. The phages described in this work are highlighted in yellow, predicted PICIs are highlighted in purple and the representative helper-satellite systems are highlighted in cyan. The internal ring of pie charts reports the probability of temperateness (in green) predicted by BACPHLIP. The external ring of pie charts shows the genome %GC (in blue). Bootstrap support values are represented as circles of different size in the midpoint of each branch. Satellite phages are indicated by black stars.

To further characterize the Mulch and Flayer systems, we performed whole-genome phylogenetic analysis of the helper and satellite phage genomes. The analysis included genomes of any phages harboring protein-coding genes with significant similarity to any of the satellite encoded proteins, as well as representative genomes for previously characterized satellites and their helpers. The resulting tree (Fig. 3) shows that the helper genomes map to distinct subclusters (BE1, MulchMansion; BE2, MindFlayer) of the well-established Actinobacteriophage database BE cluster, branching together with phages of the sister cluster BK (e.g., *Streptomyces* phage Moab). In contrast, the MulchRoom and MiniFlayer satellite genomes share an ancestral branch with *Microbacterium* phage OscarSo and the two representative members of the Actinobacteriophage database cluster BR (*Streptomyces* phage Zuko and *Streptomyces* phage KimJongPhill). The clustering of MulchRoom and MiniFlayer with OscarSo, Zuko, and KimJongPhill is primarily driven by sequence similarity in the large terminase subunit, the portal protein and the major capsid protein (Supplementary Tables S2, S3 and Supplementary Fig. 2). The proteins in this conserved region of the satellite genomes also match a few bacterial genomes belonging to *Actinomycetota* genera (*Streptomyces*, *Pseudonocardia* and *Rhodococcus*). The most comprehensive hits map to *Rhodococcus qingshengii* JCM 15477 (CP096563.1), and include also a match to the MulchRoom tyrosine integrase. This suggests that phages related to these satellites have the capacity to integrate into bacterial chromosomes, but the homologous region does not extend further in any bacterial genomes (Supplementary Tables S8, S9). Sequence similarity with previously described PICIs was only detected for MulchRoom and MiniFlayer portal protein with the corresponding protein sequence in a group of predicted *Actinomycetota* PICIs. However, the ancestral branching of MulchRoom and MiniFlayer with the aforementioned *Microbacterium* and *Streptomyces* phages reveals that these satellite genomes are not directly related to these *Actinomycetota* PICIs, and share no significant sequence similarity with experimentally reported satellite-helper systems.

### Mulch and Flayer satellites codon usage is adjusted to helper-encoded tRNAs

**Fig. 4:**
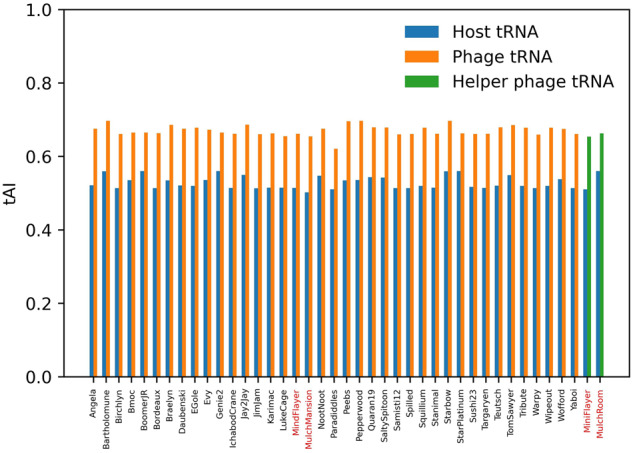
Analysis of codon usage adaptation to host and phage tRNA repertoire. tAI analysis of phages from the BE cluster (Table S10) and the satellites described in this work. For each phage, the tRNA adaptation index (tAI) of the major capsid protein gene is evaluated using the tRNA pool of the host species (blue bars). The resulting tAI value is compared with the one obtained using the tRNA pool carried by the phage (orange bars) or, for satellite phages, using the tRNA pool of their helper phage (green bars). The labels corresponding to the helper and satellite phages described in this work are shown in red.

The helper genomes in both the Mulch and Flayer systems present a low %GC content (MindFlayer: 49.5%; MulchMansion: 49.4%; Table 1) that is remarkably different from their hosts’ %GC content (71.3% and 71.9%, respectively). This anomalous composition is shared by the two satellite genomes (MiniFlayer: 46.7%; MulchRoom: 51.0%; Table 1). Both helpers encode a significant number of tRNA genes (MindFlayer: 42; MulchMansion: 41; Table 1). This is a feature that is shared by all known members of the BE cluster, raising the possibility that these phages may use their tRNA repertoire for translation. To explore this possibility, we used the tRNA Adaptation Index (tAI) to analyze the alignment of the host and phage tRNA repertoires (Tables S11 and S12), with the codon usage of the major capsid protein (MCP) gene, a common proxy for translational optimization in phage genomes [39–41]. We performed this analysis, using both host and phage tRNA repertoires, for the two helper phages and all other available BE cluster genomes. The results (Fig. 4) show that the codon usage in the MCP of BE phages is significantly better aligned with the tRNA repertoire of the phage (orange bars) than that of the host (blue bars; Wilcoxon signed-rank test *p* = 2.6·10^–8^). We then analyzed the translational optimization of the MCP gene of satellite phages MiniFlayer and MulchRoom. These satellites do not carry tRNA genes (Table 1), suggesting they may rely on the helper’s tRNAs for translation. Results (Fig. 4) indicate that codon usage in these satellite phages is aligned with their helper phages’ tRNA repertoire (green bars) instead of their hosts’ (blue bars).

## Discussion

### Novel satellite-helper system in *Actinomycetota*-infecting phages

Phage satellites were initially characterized in the 1980’s, but their general relevance to microbiology only became manifest upon the discovery of staphylococcal pathogenicity islands. The thorough characterization of SaPIs revealed the ability of these MGEs to transform their hosts, as well as the convergent evolutionary mechanisms that satellites have evolved to manipulate their helper phages [3, 42]. Subsequent work on *Vibrio* PLE’s has documented the evolutionary arms race between satellites and their helpers, and the impact of this process on their bacterial hosts [43]. Recently, bioinformatics analyses have shown that homologs of known phage satellites are widespread across the *Pseudomonadota* and *Bacillota* phyla, and present in other major bacterial groups, like the *Actinomycetota* [8]. This work has evidenced the diversity of these MGEs and has led to the description of a novel type of PICIs that generate capsids using PICI-encoded capsid genes [13]. In parallel, sequencing analysis of marine phage capsids has led to the description of an entire new family of phage satellites, known as virion-encapsidated integrative mobile elements (VEIMEs) [44]. Together, these recent developments indicate that the diversity of phage satellites remains largely unexplored.

Here we describe a new family of phage satellites infecting *Streptomyces* species. Satellite phages MulchRoom and MiniFlayer were independently identified through sequencing analysis. In both cases, the frequency of sequencing reads mapping to the assembled satellite genomes was markedly higher than that of reads mapping to the co-lysating helpers. Such an asymmetry in sequencing reads has been documented before in satellite-helper systems [7], and it is an expected consequence of satellite interference with helper multiplication [3]. In agreement with the sequencing results, the MindFlayer helper phage, but not the MiniFlayer satellite, could be effectively isolated through plaque purification, generating a purified lysate capable of infecting their isolation hosts. The genomes of these satellites show no significant sequence similarity to known phage satellites (Fig. 3), but display a region with conserved gene organization encompassing packaging and capsid formation genes that is analogous to the one reported in capsid-forming PICIs (Fig. 1) [13]. Together, these findings establish MulchRoom and MiniFlayer as a new family of phage satellites that are likely encapsidated using satellite-encoded capsids and are associated with virulent helper phages. The presence of capsid genes but absence of any obvious replicases in MulchRoom and MiniFlayer suggests that these satellites may be better defined as satellite viruses. In conjunction with the recently reported capsid-forming PICIs, these findings indicate that satellite phages define a continuous spectrum of association with their helpers that can include dependence on structural and DNA replication components. The lack of homology with previously known phage satellites highlights the challenges in studying satellite diversity through bioinformatics approaches and suggests that the ubiquity of these MGEs remains vastly underestimated.

### Morphological adaptations in response to lifestyle changes of satellite phages

The ability to integrate into the bacterial chromosome is a defining feature of phage satellites, typically mediated by tyrosine integrases [8]. Following integration, satellite phages reside in the host chromosome until they detect co-infection by a helper phage, triggering satellite replication, helper interference and satellite packaging [3]. Recent surveys, however, suggest that a small percentage of phage satellites do not encode any known integrases. While the finding of such satellites in computational analyses of bacterial chromosomes likely points to inactivated satellites [8], the identification of a substantial percentage of integrase-lacking VEIMEs assembled from direct sequencing of virion-encapsidated DNA suggests that satellite phages may be able to maintain a stable association with their helpers without the need for chromosomal integration [44]. A possible mechanism to bypass integration in satellite phages involves achieving stable residence in the host as a plasmid. Enterobacteria phage P4 can exist stably as a plasmid, and plasmid-based satellites have been reported in association with archeal helper phages [5, 45].

The absence of an integrase, as well as any identifiable lysogeny- or plasmid-associated genes, in one of the two phage satellites reported here (MiniFlayer) poses therefore an interesting question regarding its ability to maintain a stable association with a helper phage. A virulent satellite could in principle maintain an association with a temperate helper by co-infecting helper-lysogenic cells, but both experimental and genomic evidence suggest that the two helpers identified in this work are virulent phages. Our quantitative analysis of TEM data for the Flayer system shows MiniFlayer consistently associated with its helper (MindFlayer), with 80% of MindFlayer displaying attached MiniFlayer virions in picked plaque samples. This association appears to be driven by specific adsorption of the short MiniFlayer tail fibers to the MindFlayer neck, and it is likely stabilized by secondary adsorption of the MiniFlayer capsid to the MindFlayer capsid. Beyond the identified tail fiber protein, these interactions could be facilitated by the adjacent hypothetical proteins in the putative adsorption module (Fig. 1). In contrast, TEM data for the MulchRoom satellite, which encodes a putative lysogeny module, reveals virions composed of a small capsid and a tail that is consistent in length with its helper’s (MulchMansion), as it has been described for P2 [46] and capsid-forming PICIs [13]. Furthermore, no genes with homology to known tail components were detected in the MulchMansion genome, suggesting that its tail is encoded by the helper virus. This suggests that MiniFlayer has evolved morphological changes that enable it to enter the host cell concurrently with its helper as the means to guarantee co-infection and continued propagation, although the precise mechanism for satellite entry into the cell remains to be elucidated. In the absence of chromosomal integration or the ability to enter a plasmid stage, co-entry into the host cell is the only plausible mechanism for a satellite phage to maintain a stable association with a virulent helper. Co-entry into the host has been postulated to play a role in the infection cycle of virophages parasitizing giant DNA viruses, even though all known virophages are capable of integrating in either the helper virus or the host cell chromosomes [47]. To our knowledge, this is therefore, the first report of a morphological adaptation enabling a putatively virulent satellite to maintain a stable association with a helper virus.

### Genomic adaptation of satellites to helper phages

Many phage genomes contain multiple tRNA genes, often found in clusters [41]. Computational and experimental analyses have revealed that late phage genes tend to use codons for phage-encoded tRNAs, indicating that phages exploit their tRNA genes to maximize translational throughput of structural genes [48, 49]. The presence and translational impact of tRNA genes is more pronounced in virulent phages, and it may also contribute to increased host range [48, 50]. The observation that helper phages MindFlayer and MulchMansion, encoding a large repertoire of tRNA genes, have remarkably low %GC content when compared to their isolation hosts prompted us to analyze whether structural genes in these phages preferentially used codons recognized by the phage-encoded tRNAs.

Our results indicate that structural genes in both helper phages, as well as related phages from the Actinobacteriophage database BE cluster, display a codon usage bias consistent with the utilization of phage-encoded tRNAs (Fig. 4; orange bars). Interestingly, the genomes of satellite phages MiniFlayer and MulchRoom also present anomalously low %GC content, matching their helpers’ %GC content instead of their hosts’. Our analysis shows that codon usage in the structural genes of both satellites is aligned with the tRNA repertoire of their helpers, rather than the tRNA pool of the host (Fig. 4; green bars). The lack of homology between satellite and helper phages’ structural genes, and the fact that the phages most closely related with both satellites have %GC content matching their hosts (Table 1), suggest that these satellites have evolved to match the %GC and codon usage of their helpers as a means to optimize translational throughput during co-infection. This is significant, because %GC amelioration and codon usage optimization are known to be slow processes in bacteria, spanning millions of years [51]. Together with the phylogenetic analysis (Fig. 3), this points to a long-standing association between ancestors of these satellites and BE cluster helper ancestors. Furthermore, our results indicate that the structural genes of both satellite phages are optimized for translation, suggesting that their capsid genes are actively expressed and used in capsid formation, as recently described for unrelated phage satellites [13]. The co-evolutionary arms race between satellite phages and their helpers has garnered substantial attention in recent years, leading to the identification of multiple phage defense and interference systems [52]. Our results indicate that this co-evolutionary process can also shape other aspects of satellite genomes, like their codon usage bias.

### Supplementary information

Supplementary material captions

Supplementary figures

Supplementary tables (PDF)

## Data Availability

The data used for this study is publicly available and accession numbers for all data items are provided in the text and the Supplementary Material.
