# Supplementary material for: Simultaneous entry as an adaptation to virulence in a novel satellite-helper system infecting Streptomyces species
Source: ISME J. 2023 Oct 31;17(12):2381–8. doi: 10.1038/s41396-023-01548-0 (PMC10690885; doi:10.1038/s41396-023-01548-0)
Supplement: Supplementary file 1 — Supplementary material captions [file 41396_2023_1548_MOESM1_ESM.pdf]

Supplementary File Information

Supplementary figures: Provided as a single PDF document.

Fig. S1 - Plaque morphologies of A) MindFlayer/MiniFlayer; B) MulchMansion/MulchRoom.

Fig. S2 - Comparative genome organization plot of the satellite phage MiniFlayer and ancestrally related phages: *Microbacterium* phage OscarSo, *Streptomyces* phage Zuko and *Streptomyces* phage KimJongPhill.

Supplementary tables: Provided as a single excel file with multiple tabs

Table S1 - Phage genomes collected for this study. The table reports why they were included and whether they were selected as part of the set of 100 representative genomes used to draw the phylogenetic tree.

Table S2 - Results from the tBLASTn search querying the NCBI GenBank database, restricted to viruses (taxonomy ID: 10239), with the proteome of MiniFlayer.

Table S3 - Results from the tBLASTn search querying the NCBI GenBank database, restricted to viruses (taxonomy ID: 10239), with the proteome of MulchRoom.

Table S4 - Results from the BLASTP search querying the proteomes of phage satellites (de Sousa JAM, Fillol-Salom A, Penadés JR, Rocha EPC. Identification and characterization of thousands of bacteriophage satellites across bacteria. *Nucleic Acids Res* 2023; gkad123) with the proteome of MiniFlayer.

Table S5 - Results from the BLASTP search querying the proteomes of phage satellites (de Sousa JAM, Fillol-Salom A, Penadés JR, Rocha EPC. Identification and characterization of thousands of bacteriophage satellites across bacteria. *Nucleic Acids Res* 2023; gkad123) with the proteome of MulchRoom.

Table S6 - PICI sequences selected for this study, based on relatedness to the proteomes of the phage satellites MiniFlayer and MulchRoom. The “TAG” column reports the name used in the phylogenetic tree.

Table S7 - Representatives of the major classes of helper-satellite systems described in the literature, included in the phylogenetic analyses.

Table S8 - Results from the tBLASTn search querying the NCBI GenBank database, restricted to bacteria (taxonomy ID: 2), with the proteome of MiniFlayer.

Table S9 - Results from the tBLASTn search querying the NCBI GenBank database, restricted to bacteria (taxonomy ID: 2), with the proteome of MulchRoom.

Table S10 - Genome accession numbers of the phages from the BE cluster and their corresponding hosts.

Table S11 - Inferred gene copy numbers for tRNA genes (grouped by anticodon) for the phages belonging to the BE cluster.

Table S12 - Inferred gene copy numbers for tRNA genes (grouped by anticodon) for the hosts of the phages belonging to the BE cluster.
