# Supplementary material for: Simultaneous entry as an adaptation to virulence in a novel satellite-helper system infecting Streptomyces species
Source: ISME J. 2023 Oct 31;17(12):2381–8. doi: 10.1038/s41396-023-01548-0 (PMC10690885; doi:10.1038/s41396-023-01548-0)
Supplement: Supplementary file 2 — Supplementary figures [file 41396_2023_1548_MOESM2_ESM.pdf]

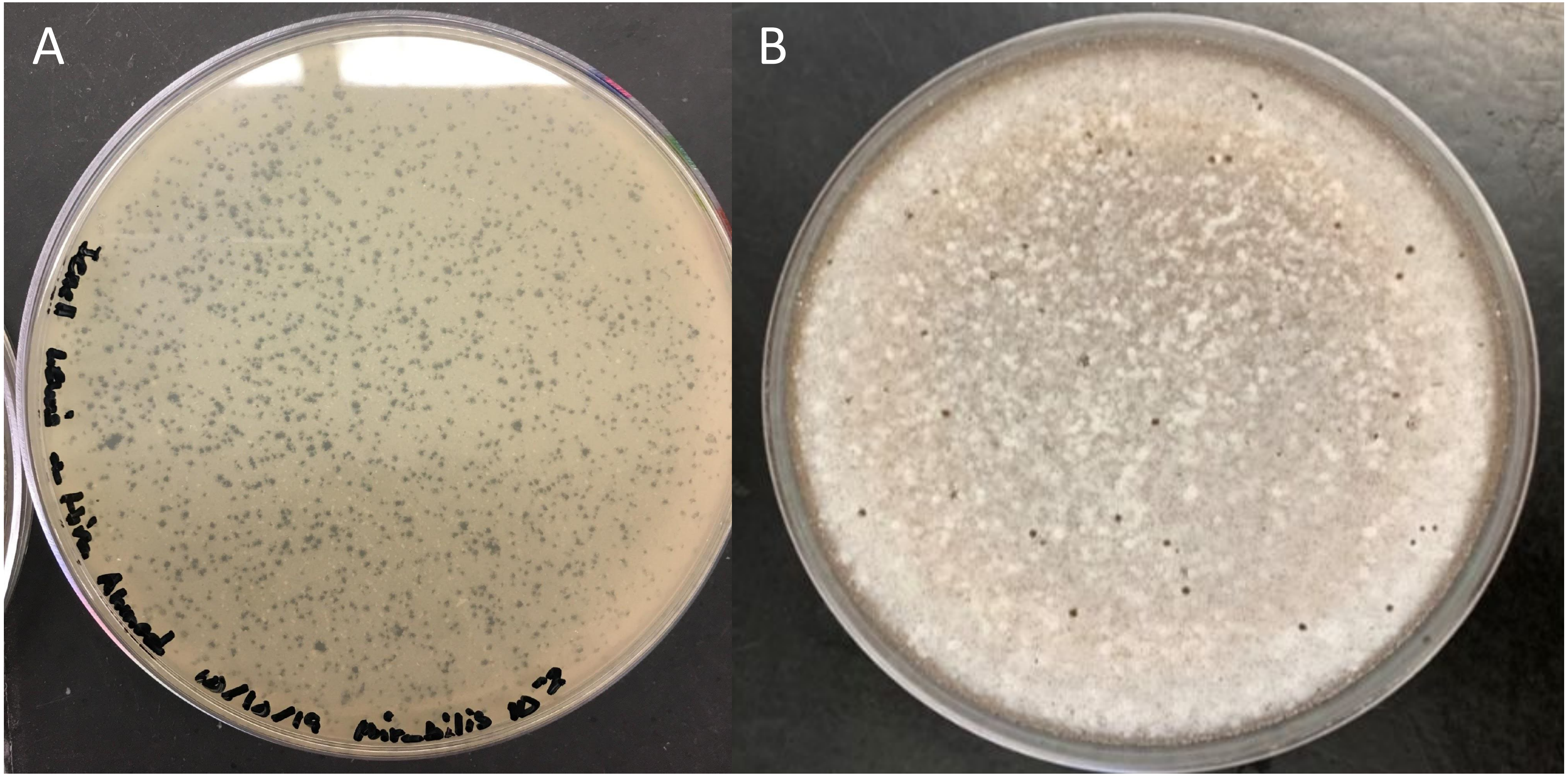


**Fig. S1.** Plaque morphologies of A) MindFlayer/MiniFlayer; B) MulchMansion/MulchRoom

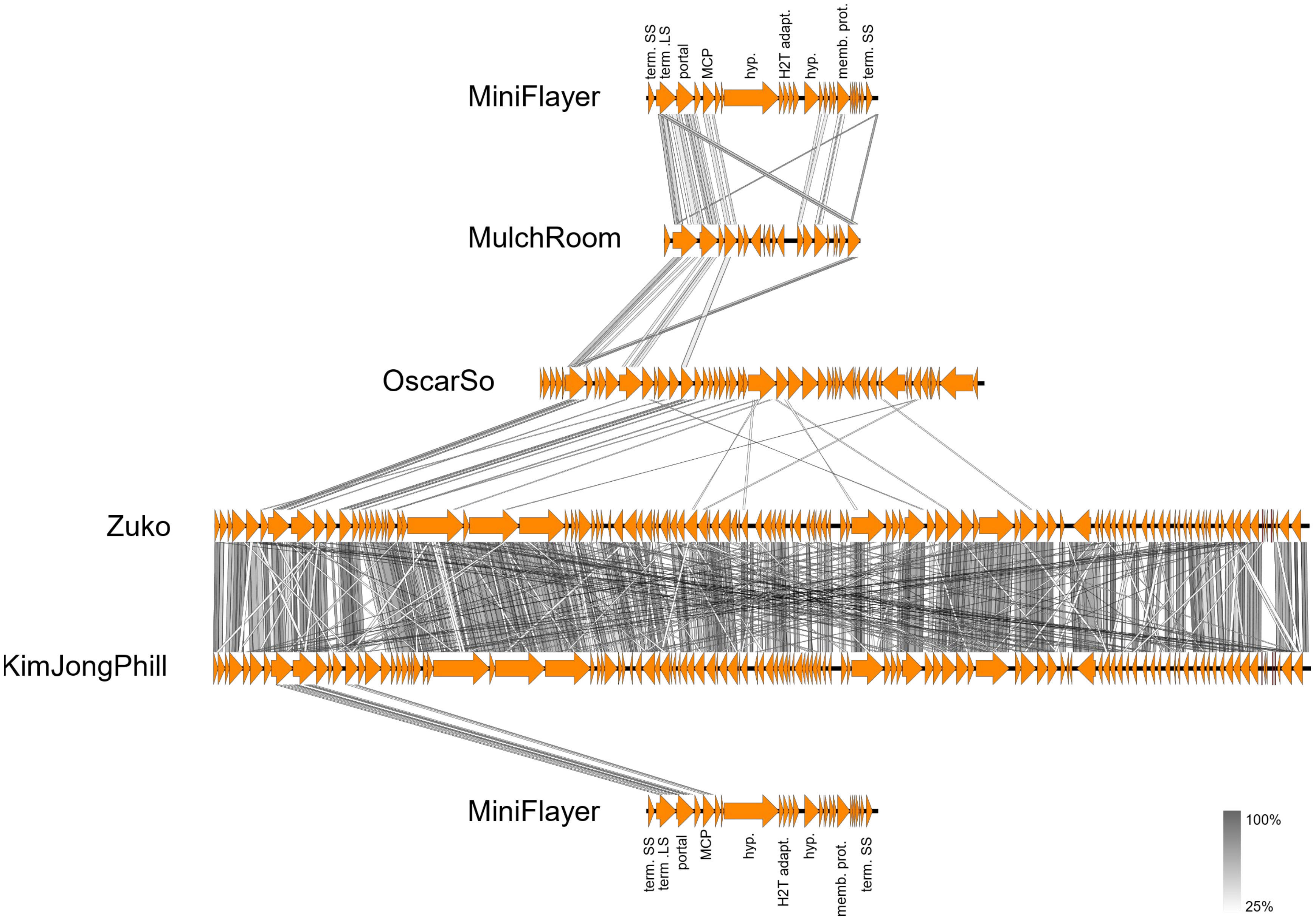


**Fig. S2 -** Comparative genome organization plot of the satellite phage MiniFlayer and ancestrally related phages: *Microbacterium* phage OscarSo, *Streptomyces* phage Zuko and *Streptomyces* phage KimJongPhill.
