# Supplementary material for: Simultaneous entry as an adaptation to virulence in a novel satellite-helper system infecting Streptomyces species
Source: ISME J. 2023 Oct 31;17(12):2381–8. doi: 10.1038/s41396-023-01548-0 (PMC10690885; doi:10.1038/s41396-023-01548-0)
Supplement: Supplementary file 3 — Supplementary tables (PDF) [file 41396_2023_1548_MOESM3_ESM.pdf]

Supplementary Table 1. Phage genomes collected for this study. The table reports why they were included and whetl

Name	Description	Source genome
CP011853.1_PICI_1	PICI CP011853.1_PICI_1 extracted from record CP011853	CP011853
CP020809.1_PICI_1	PICI CP020809.1_PICI_1 extracted from record CP020809	CP020809
CP025435.1_PICI_1	PICI CP025435.1_PICI_1 extracted from record CP025435	CP025435
EcCICFT073	Escherichia coli CFT073 PICI EcCICFT073. The PICI EcCICFT073	AE014075
JX006077	Saccharomonospora phage PIS 136, complete genome	JX006077
KC152960	Vibrio cholerae O1 biovar El Tor strain KS393 PICI-like elemer	KC152960
KT997826	Uncultured Mediterranean phage uvDeep-CGR0-KM14-C182,	KT997826
KU998252	Gordonia phage PatrickStar, complete genome	KU998252
KU998254	Gordonia phage Kampe, complete genome	KU998254
KY555142	Caulobacter phage Ccr10, complete genome	KY555142
KY555143	Caulobacter phage Ccr2, complete genome	KY555143
KY555144	Caulobacter phage Ccr5, complete genome	KY555144
KY555145	Caulobacter phage Ccr29, complete genome	KY555145
KY555146	Caulobacter phage Ccr32, complete genome	KY555146
KY555147	Caulobacter phage Ccr34, complete genome	KY555147
MF358541	Streptomyces phage Warpy, complete genome	MF358541
MF358542	Streptomyces phage Sushi23, complete genome	MF358542
MG820643	Propionibacterium phage pa35, complete genome	MG820643
MG820644	Propionibacterium phage pa33, complete genome	MG820644
MG820645	Propionibacterium phage pa3-SS3, complete genome	MG820645
MH001457	Streptomyces phage Nesbitt, complete genome	MH001457
MH019215	Streptomyces phage Yara, complete genome	MH019215
MH019216	Streptomyces phage Wentworth, complete genome	MH019216
MH155870	Streptomyces phage Ibantik, complete genome	MH155870
MH479923	Gordonia phage RobinSparkles, complete genome	MH479923
MH576964	Streptomyces phage Starbow, complete genome	MH576964
MH590589	Streptomyces phage SparkleGoddess, complete genome	MH590589
MH744420	Streptomyces phage Kromp, complete genome	MH744420
MK240575	Thermobifida phage P318, complete genome	MK240575
MK305891	Streptomyces phage Gibson, complete genome	MK305891
MK359332	Streptomyces phage Genie2, complete genome	MK359332
MK359351	Streptomyces phage BoomerJR, complete genome	MK359351
MK460248	Streptomyces phage Teutsch, complete genome	MK460248
MK620896	Streptomyces phage Circinus, complete genome	MK620896
MK801722	Streptomyces phage Birchlyn, complete genome	MK801722
MK894436	Gordonia phage Boopy, complete genome	MK894436
MK919478	Gordonia phage Ziko, complete genome	MK919478
MN096363	Gordonia phage Gibbles, complete genome	MN096363
MN369743	Streptomyces phage Tribute, complete genome	MN369743
MN369750	Streptomyces phage TomSawyer, complete genome	MN369750
MN369757	Streptomyces phage Bordeaux, complete genome	MN369757
MN428060	Streptomyces phage IchabodCrane, complete genome	MN428060
MN484599	Streptomyces phage Wipeout, complete genome	MN484599
MN586014	Gordonia phage Keelan, complete genome	MN586014
MT104122	Sporosarcina phage Lietuvens, complete genome	MT104122

MT114162	Streptomyces phage Moab, complete genome	MT114162
MT498037	Streptomyces phage Dryad, complete genome	MT498037
MT498053	Streptomyces phage PHTowN, complete genome	MT498053
MT521995	Gordonia phage Moosehead, complete genome	MT521995
MT684590	Streptomyces phage LilMartin, complete genome	MT684590
MT897905	Streptomyces phage MulchMansion, complete genome	MT897905
MT897908	Streptomyces phage ShakeNBake, complete genome	MT897908
MT936332	Streptomyces phage phiRKBJ001, complete genome	MT936332
MW161466	Cutibacterium phage PAVL33, complete genome	MW161466
MW161467	Cutibacterium phage PAVL34, complete genome	MW161467
MW291014	Streptomyces phage MindFlayer, complete genome	MW291014
MW365952	Streptomyces phage Belfort, complete genome	MW365952
MW507134	Streptomyces phage Battuta, complete genome	MW507134
MW507136	Streptomyces phage Cross, complete genome	MW507136
MW601220	Gordonia phage Guey18, complete genome	MW601220
MZ574430	Caulobacter phage C2, complete genome	MZ574430
MZ574432	Caulobacter phage BL47, complete genome	MZ574432
MZ648033	Streptomyces phage Rooney, complete genome	MZ648033
MZ648036	Streptomyces phage Lizz, complete genome	MZ648036
MZ820095	Streptomyces phage Karp, complete genome	MZ820095
MZ958750	Streptomyces phage Targaryen, complete genome	MZ958750
MiniFlayer	Satellite phage MiniFlayer	MiniFlayer
MulchRoom	Pirate Phage Mulchroom	Mulchroom
NC_001416	Enterobacteria phage lambda, complete genome	NC_001416
NC_001895	Enterobacteria phage P2, complete genome	NC_001895
NC_009526	Staphylococcus phage 80alpha, complete genome	NC_009526
NC_014229	Streptomyces phage phiSASD1, complete genome	NC_014229
NC_015157	Vibrio phage ICP1, complete genome	NC_015157
NC_016654	Rhodococcus phage REQ3, complete genome	NC_016654
NC_019405	Caulobacter phage phiCbK, complete genome	NC_019405
NC_019407	Caulobacter phage CcrMagneto, complete genome	NC_019407
NC_019408	Caulobacter phage CcrRogue, complete genome	NC_019408
NC_019410	Caulobacter phage CcrKarma, complete genome	NC_019410
NC_019411	Caulobacter phage CcrSwift, complete genome	NC_019411
NC_021334	Mycobacterium phage WIVsmall, complete genome	NC_021334
NC_027391	Propionibacterium phage PHL041M10, complete genome	NC_027391
NC_028807	Streptomyces phage SF1, complete genome	NC_028807
NC_029098	Streptomyces phage Jay2Jay, complete genome	NC_029098
NC_030902	Gordonia phage GMA1, complete genome	NC_030902
NC_030915	Gordonia phage Orchid, complete genome	NC_030915
NC_030916	Tsukamurella phage TPA4, complete genome	NC_030916
NC_030945	Bacillus phage BalMu-1 copy 1, complete genome	NC_030945
NC_041879	Bacillus phage Mgbh1, complete genome	NC_041879
NC_042008	Streptomyces phage Mildred21, complete genome	NC_042008
NC_042009	Streptomyces phage NootNoot, complete genome	NC_042009
NC_042010	Streptomyces phage Paradiddles, complete genome	NC_042010
NC_042011	Streptomyces phage Peebs, complete genome	NC_042011
NC_042012	Streptomyces phage Samisti12, complete genome	NC_042012
NC_042105	Streptomyces phage BillNye, complete genome	NC_042105

NC_047904	Streptomyces phage AbbeyMikolon, complete genome	NC_047904
NC_047906	Streptomyces phage Rowa, complete genome	NC_047906
NC_047913	Faecalibacterium phage FP_Mushu, complete genome	NC_047913
NC_048027	Gordonia phage Fryberger, complete genome	NC_048027
NC_048028	Gordonia phage Ronaldo, complete genome	NC_048028
NC_048045	Caulobacter phage CcrBL10, complete genome	NC_048045
NC_048047	Caulobacter phage CcrBL9, complete genome	NC_048047
NC_048048	Caulobacter phage CcrSC, complete genome	NC_048048
NC_048091	Arthrobacter phage Bridgette, complete genome	NC_048091
NC_048092	Arthrobacter phage Constance, complete genome	NC_048092
NC_048094	Arthrobacter phage Eileen, complete genome	NC_048094
NC_048095	Arthrobacter phage Judy, complete genome	NC_048095
NC_048096	Arthrobacter phage Peas, complete genome	NC_048096
NC_048721	Streptomyces phage StarPlatinum, complete genome	NC_048721
NC_048722	Streptomyces phage Wofford, complete genome	NC_048722
NC_048723	Streptomyces phage LukeCage, complete genome	NC_048723
NC_048724	Streptomyces phage Karimac, complete genome	NC_048724
NC_048728	Streptomyces phage Comrade, complete genome	NC_048728
NC_048730	Streptomyces phage Yaboi, complete genome	NC_048730
NC_055791	Streptomyces phage EGole, complete genome	NC_055791
NC_055809	Streptomyces phage Evy, complete genome	NC_055809
NC_055813	Streptomyces phage Braelyn, complete genome	NC_055813
NC_055822	Streptomyces phage Daubenski, complete genome	NC_055822
NC_055842	Streptomyces phage Bmoc, complete genome	NC_055842
NC_070762	Gordonia phage Sixama, complete genome	NC_070762
NC_070763	Gordonia phage Forza, complete genome	NC_070763
NC_070781	Streptomyces phage Tomas, complete genome	NC_070781
NC_070782	Streptomyces phage Coruscant, complete genome	NC_070782
NC_070785	Streptomyces phage Wakanda, complete genome	NC_070785
NC_070786	Streptomyces phage Muntaha, complete genome	NC_070786
NC_070797	Gordonia phage Vasanti, complete genome	NC_070797
NC_070799	Gordonia phage Pickett, complete genome	NC_070799
NC_070828	Streptomyces phage Dubu, complete genome	NC_070828
NC_070829	Streptomyces phage Zuko, complete genome	NC_070829
NC_070830	Streptomyces phage KimJongPhill, complete genome	NC_070830
NC_070831	Arthrobacter phage Richie, complete genome	NC_070831
NC_070832	Arthrobacter phage Auxilium, complete genome	NC_070832
NC_070833	Arthrobacter phage Hestia, complete genome	NC_070833
NC_070834	Arthrobacter phage Persistence, complete genome	NC_070834
NC_070835	Arthrobacter phage Isolde, complete genome	NC_070835
NC_070836	Arthrobacter phage Seahorse, complete genome	NC_070836
NC_070837	Arthrobacter phage Faja, complete genome	NC_070837
NC_070948	Gordonia phage Mollymur, complete genome	NC_070948
NZ_AP022565.1_PICI_1	PICI NZ_AP022565.1_PICI_1 extracted from record NZ_AP022	NZ_AP022565
NZ_AP022567.1_PICI_1	PICI NZ_AP022567.1_PICI_1 extracted from record NZ_AP022	NZ_AP022567
NZ_AP022575.1_PICI_1	PICI NZ_AP022575.1_PICI_1 extracted from record NZ_AP022	NZ_AP022575
NZ_AP022604.1_PICI_1	PICI NZ_AP022604.1_PICI_1 extracted from record NZ_AP022	NZ_AP022604
NZ_AP023287.1_PICI_1	PICI NZ_AP023287.1_PICI_1 extracted from record NZ_AP023	NZ_AP023287
NZ_AP024257.1_PICI_1	PICI NZ_AP024257.1_PICI_1 extracted from record NZ_AP024	NZ_AP024257

NZ_CP011269.1_PICI_2	PICI NZ_CP011269.1_PICI_2 extracted from record NZ_CP011	NZ_CP011269
NZ_CP011853.1_PICI_1	PICI NZ_CP011853.1_PICI_1 extracted from record NZ_CP011	NZ_CP011853
NZ_CP017150.1_PICI_1	PICI NZ_CP017150.1_PICI_1 extracted from record NZ_CP017	NZ_CP017150
NZ_CP046257.1_PICI_1	PICI NZ_CP046257.1_PICI_1 extracted from record NZ_CP046	NZ_CP046257
NZ_CP060292.1_PICI_1	PICI NZ_CP060292.1_PICI_1 extracted from record NZ_CP060	NZ_CP060292
NZ_CP065266.1_PICI_1	PICI NZ_CP065266.1_PICI_1 extracted from record NZ_CP065	NZ_CP065266
NZ_CP065269.1_PICI_1	PICI NZ_CP065269.1_PICI_1 extracted from record NZ_CP065	NZ_CP065269
NZ_CP065373.1_PICI_1	PICI NZ_CP065373.1_PICI_1 extracted from record NZ_CP065	NZ_CP065373
NZ_LR134355.1_PICI_1	PICI NZ_LR134355.1_PICI_1 extracted from record NZ_LR134	NZ_LR134355
NZ_LR134473.1_PICI_1	PICI NZ_LR134473.1_PICI_1 extracted from record NZ_LR134	NZ_LR134473
OK310502	Streptomyces phage Bartholomune, complete genome	OK310502
OM112210	Bacillus phage PK2, complete genome	OM112210
OM654379	Bacillus phage vB_BauS_KLEB27-1, complete genome	OM654379
ON108650	Streptomyces phage Squillium, complete genome	ON108650
ON260819	Streptomyces phage SaltySpitoon, complete genome	ON260819
ON260828	Streptomyces phage Quaran19, complete genome	ON260828
ON456343	Streptomyces phage Stigma, complete genome	ON456343
ON724017	Arthrobacter phage Gorpy, complete genome	ON724017
ON970561	Arthrobacter phage GlobiWarming, complete genome	ON970561
ON970579	Streptomyces phage Spilled, complete genome	ON970579
ON970590	Streptomyces phage JimJam, complete genome	ON970590
ON970591	Streptomyces phage Angela, complete genome	ON970591
ON970598	Arthrobacter phage Sakai, complete genome	ON970598
OP021680	Streptomyces phage Stanimal, complete genome	OP021680
OP072786	MAG: Bacteriophage sp. isolate 3874_64122, complete genor	OP072786
OP297539	Gordonia phage BlueNGold, complete genome	OP297539
OP434442	Arthrobacter phage RadFad, complete genome	OP434442
OP434449	Microbacterium phage OscarSo, complete genome	OP434449
OP434463	Arthrobacter phage EvePickles, complete genome	OP434463
OP751148	Streptomyces phage Success, complete genome	OP751148
OP820470	Gordonia phage Mareelih, complete genome	OP820470
U93688	Staphylococcus aureus transposon Tn557 toxic shock syndror	U93688
X51522	Bacteriophage P4 complete DNA genome	X51522

ıer they were selected as part of the set of 100 representative genomes used to draw the phylogenetic tree.

Mind Mulch helper-	From tBLASTn	From de	From satellites	On tree
		X		X
		X		X
		X		X
			X	X
	X			X
			X	X
	X			X
	X			X
	X			
	X			
	X			
	X			
	X			
	X			
	X			
	X			
	X			
	X			
	X			
	X			X
	X			
	X			X
	X			X
	X			X
	X			
	X			
	X			
	X			X
	X			X
	X			X
	X			
	X			X
	X			
	X			X
	X			
	X			X
	X			
	X			
	X			
	X			
	X			
	X			
	X			
	X			X
	X			X

	X		X
	X		X
	X		
	X		X
	X		
X	X		X
	X		
	X		X
	X		X
	X		
X	X		X
	X		
	X		
	X		
	X		X
	X		X
	X		
	X		
	X		X
	X		
	X		
X			X
X			X
		X	X
		X	X
		X	X
	X		X
		X	X
	X		X
	X		
	X		
	X		
	X		X
	X		
	X		X
	X		
	X		X
	X		
	X		X
	X		
	X		X
	X		X
	X		X
	X		
	X		
	X		
	X		
	X		X
	X		

X		X
X		X
X		X
X		
X		
X		X
X		
X		X
X		X
X		
X		X
X		
X		X
X		
X		X
X		
X		
X		
X		
X		
X		
X		
X		X
X		X
X		X
X		
X		X
X		X
X		
X		X
X		X
X		X
X		X
X		X
X		X
X		X
X		X
X		X
X		X
X		X
X		X
X		X
X		X
	X	X
	X	X
	X	X
	X	X
	X	X
	X	X

	X		X
	X		X
	X		X
	X		X
	X		X
	X		
	X		X
	X		X
	X		
	X		X
X			X
X			X
X			X
X			
X			
X			
X			X
X			
X			
X			
X			
X			
X			X
X			
X			X
X			
X			X
X			X
X			X
X			X
X			
		X	X
		X	X

Supplementary Table 2. Results from the tBLASTn search querying the NCBI GenBank database, restricted to viruses (taxonomy ID: 10239), with the prot

Query protein	Hit genome	Start position of	End position of	Phage name	E_value	Q_cov
SEA_MINIFLAYER_2	MT498037	2959	4431	Streptomyces ph	1.15E-100	0.938976378
SEA_MINIFLAYER_2	NC_070830	4497	5918	Streptomyces ph	1.47E-99	0.960629921
SEA_MINIFLAYER_2	MH019216	2632	4134	Streptomyces ph	4.59E-97	0.964566929
SEA_MINIFLAYER_2	MH019215	2397	3854	Streptomyces ph	1.34E-96	0.938976378
SEA_MINIFLAYER_2	MT498053	2843	4333	Streptomyces ph	1.72E-95	0.946850394
SEA_MINIFLAYER_2	MZ648036	2843	4333	Streptomyces ph	1.72E-95	0.946850394
SEA_MINIFLAYER_2	MT897908	2843	4333	Streptomyces ph	1.72E-95	0.946850394
SEA_MINIFLAYER_2	NC_070829	4190	5605	Streptomyces ph	6.96E-94	0.927165354
SEA_MINIFLAYER_2	MK305891	2887	4377	Streptomyces ph	8.09E-94	0.946850394
SEA_MINIFLAYER_2	MT936332	2832	4334	Streptomyces ph	2.36E-93	0.964566929
SEA_MINIFLAYER_2	MZ648033	2887	4377	Streptomyces ph	5.99E-93	0.946850394
SEA_MINIFLAYER_2	OP434449	2024	3448	Microbacterium	2.75E-83	0.942913386
SEA_MINIFLAYER_2	OP072786	2451	3989	MAG: Bacterioph	2.83E-23	0.93503937
SEA_MINIFLAYER_2	NC_041879	7976	9487	Bacillus phage M	3.78E-19	0.942913386
SEA_MINIFLAYER_2	KP063903	14099	15358	Bacillus phage Ba	1.01E-15	0.781496063
SEA_MINIFLAYER_2	NC_030945	14111	15370	Bacillus phage Ba	1.01E-15	0.781496063
SEA_MINIFLAYER_2	OM112210	1947	3383	Bacillus phage Pk	6.62E-15	0.893700787
SEA_MINIFLAYER_2	NC_047913	13573	15063	Faecalibacterium	6.49E-13	0.919291339
SEA_MINIFLAYER_2	MT104122	1780	3144	Sporosarcina pha	9.63E-13	0.816929134
SEA_MINIFLAYER_2	OM654379	369	1730	Bacillus phage vE	9.01E-11	0.811023622
SEA_MINIFLAYER_3	NC_070830	6083	7531	Streptomyces ph	3.38E-57	0.910714286
SEA_MINIFLAYER_3	NC_070829	5852	7285	Streptomyces ph	1.11E-55	0.90625
SEA_MINIFLAYER_3	MH019215	4054	5352	Streptomyces ph	2.53E-41	0.877232143
SEA_MINIFLAYER_3	MK305891	4582	5916	Streptomyces ph	4.95E-39	0.90625
SEA_MINIFLAYER_3	MZ648033	4582	5916	Streptomyces ph	4.95E-39	0.90625
SEA_MINIFLAYER_3	MT897908	4564	5856	Streptomyces ph	5.48E-39	0.875
SEA_MINIFLAYER_3	MZ648036	4564	5856	Streptomyces ph	8.21E-39	0.875
SEA_MINIFLAYER_3	MT498053	4564	5856	Streptomyces ph	9.54E-39	0.875
SEA_MINIFLAYER_3	MH019216	4290	5627	Streptomyces ph	1.10E-37	0.908482143
SEA_MINIFLAYER_3	MT936332	4490	5917	Streptomyces ph	2.83E-36	0.986607143
SEA_MINIFLAYER_3	MT498037	4802	6196	Streptomyces ph	6.22E-36	0.953125
SEA_MINIFLAYER_3	OP434449	5978	7345	Microbacterium	1.02E-27	0.915178571
SEA_MINIFLAYER_3	MH155870	30936	32132	Streptomyces ph	3.18E-26	0.892857143

SEA_MINIFLAYER_3	OP751148	27481	28743	Streptomyces ph	2.46E-23	0.9375
SEA_MINIFLAYER_3	NC_047906	2328	3431	Streptomyces ph	2.72E-19	0.790178571
SEA_MINIFLAYER_3	KT997826	11574	12845	Uncultured Medi	3.03E-19	0.917410714
SEA_MINIFLAYER_3	ON970561	2476	3666	Arthrobacter pha	4.36E-19	0.868303571
SEA_MINIFLAYER_3	OP434463	2437	3543	Arthrobacter pha	2.03E-18	0.810267857
SEA_MINIFLAYER_3	NC_070833	2509	3615	Arthrobacter pha	2.39E-18	0.810267857
SEA_MINIFLAYER_3	NC_048091	2476	3666	Arthrobacter pha	2.96E-18	0.868303571
SEA_MINIFLAYER_3	NC_048095	2476	3666	Arthrobacter pha	2.96E-18	0.868303571
SEA_MINIFLAYER_3	NC_048092	2477	3667	Arthrobacter pha	2.96E-18	0.868303571
SEA_MINIFLAYER_3	NC_070832	2437	3543	Arthrobacter pha	4.83E-18	0.810267857
SEA_MINIFLAYER_3	NC_070831	2437	3543	Arthrobacter pha	4.84E-18	0.810267857
SEA_MINIFLAYER_3	MK240575	18016	19224	Thermobifida ph	1.69E-17	0.917410714
SEA_MINIFLAYER_3	ON970598	2437	3537	Arthrobacter pha	5.54E-17	0.810267857
SEA_MINIFLAYER_3	NC_070837	2436	3536	Arthrobacter pha	5.54E-17	0.810267857
SEA_MINIFLAYER_3	OP434442	2436	3536	Arthrobacter pha	5.54E-17	0.810267857
SEA_MINIFLAYER_3	ON724017	2437	3537	Arthrobacter pha	5.54E-17	0.810267857
SEA_MINIFLAYER_3	NC_014229	2355	3554	Streptomyces ph	2.02E-16	0.825892857
SEA_MINIFLAYER_3	NC_048094	2476	3666	Arthrobacter pha	1.64E-15	0.868303571
SEA_MINIFLAYER_3	NC_048096	2476	3666	Arthrobacter pha	1.65E-15	0.868303571
SEA_MINIFLAYER_3	MH744420	2491	3750	Streptomyces ph	1.86E-15	0.908482143
SEA_MINIFLAYER_3	NC_070828	2263	3546	Streptomyces ph	1.25E-14	0.888392857
SEA_MINIFLAYER_3	NC_070835	2436	3542	Arthrobacter pha	4.67E-14	0.810267857
SEA_MINIFLAYER_3	NC_070836	2433	3539	Arthrobacter pha	5.73E-14	0.810267857
SEA_MINIFLAYER_3	MH001457	2457	3704	Streptomyces ph	1.45E-12	0.890625
SEA_MINIFLAYER_3	NC_047904	2457	3704	Streptomyces ph	1.45E-12	0.890625
SEA_MINIFLAYER_3	NC_028807	2457	3704	Streptomyces ph	1.45E-12	0.890625
SEA_MINIFLAYER_3	NC_070834	2346	3614	Arthrobacter pha	6.13E-12	0.90625
SEA_MINIFLAYER_3	NC_070948	8945	10111	Gordonia phage	6.34E-12	0.808035714
SEA_MINIFLAYER_5	NC_070829	9409	10299	Streptomyces ph	9.79E-38	0.945945946
SEA_MINIFLAYER_5	NC_070830	9976	10866	Streptomyces ph	7.41E-34	0.945945946
SEA_MINIFLAYER_5	MZ648036	8920	9804	Streptomyces ph	7.37E-32	0.949324324
SEA_MINIFLAYER_5	MT897908	8920	9804	Streptomyces ph	7.37E-32	0.949324324
SEA_MINIFLAYER_5	MT498053	8920	9804	Streptomyces ph	7.59E-32	0.949324324
SEA_MINIFLAYER_5	MK305891	9010	9888	Streptomyces ph	6.07E-31	0.942567568
SEA_MINIFLAYER_5	MZ648033	9007	9885	Streptomyces ph	6.07E-31	0.942567568
SEA_MINIFLAYER_5	MH019216	8722	9600	Streptomyces ph	4.26E-28	0.942567568

SEA_MINIFLAYER_5	MT936332	8944	9822	Streptomyces ph	4.17E-27	0.942567568
SEA_MINIFLAYER_5	OP434449	10635	11561	Microbacterium	7.31E-27	0.945945946
SEA_MINIFLAYER_5	MT498037	9434	10312	Streptomyces ph	2.00E-26	0.942567568
SEA_MINIFLAYER_5	MH019215	8027	8905	Streptomyces ph	1.23E-24	0.942567568
SEA_MINIFLAYER_6	NC_070762	63948	64370	Gordonia phage	2.07E-41	0.986111111
SEA_MINIFLAYER_6	OP820470	63944	64366	Gordonia phage	2.38E-40	0.986111111
SEA_MINIFLAYER_6	NC_070763	64442	64864	Gordonia phage	2.38E-40	0.986111111
SEA_MINIFLAYER_6	OP297539	64514	64936	Gordonia phage	2.38E-40	0.986111111
SEA_MINIFLAYER_6	MK894436	64526	64948	Gordonia phage	2.38E-40	0.986111111
SEA_MINIFLAYER_6	NC_030902	21114	21530	Gordonia phage	3.20E-40	0.986111111
SEA_MINIFLAYER_6	MN096363	39192	39590	Gordonia phage	3.71E-38	0.930555556
SEA_MINIFLAYER_6	MH479923	39674	40072	Gordonia phage	3.71E-38	0.930555556
SEA_MINIFLAYER_6	NC_021334	30833	31270	Mycobacterium p	1.68E-36	0.986111111
SEA_MINIFLAYER_6	KC736071	30833	31270	Mycobacterium p	1.68E-36	0.986111111
SEA_MINIFLAYER_6	KU998254	38765	39169	Gordonia phage	9.55E-36	0.9375
SEA_MINIFLAYER_6	NC_030915	38766	39170	Gordonia phage	9.55E-36	0.9375
SEA_MINIFLAYER_6	KU998252	38765	39169	Gordonia phage	9.55E-36	0.9375
SEA_MINIFLAYER_6	NC_016654	37403	37822	Rhodococcus pha	1.01E-35	0.979166667
SEA_MINIFLAYER_6	MW601220	33029	33445	Gordonia phage	1.12E-34	0.986111111
SEA_MINIFLAYER_6	MN586014	31829	32245	Gordonia phage	2.81E-34	0.986111111
SEA_MINIFLAYER_6	NC_048028	32730	33146	Gordonia phage	8.51E-33	0.986111111
SEA_MINIFLAYER_6	NC_048027	31831	32247	Gordonia phage	1.92E-32	0.986111111
SEA_MINIFLAYER_6	MK919478	32716	33132	Gordonia phage	1.92E-32	0.986111111
SEA_MINIFLAYER_6	MT521995	21157	21582	Gordonia phage	6.17E-30	0.986111111
SEA_MINIFLAYER_6	NC_030916	25394	25831	Tsukamurella pha	7.35E-30	0.986111111
SEA_MINIFLAYER_6	NC_070799	20886	21311	Gordonia phage	2.94E-29	0.986111111
SEA_MINIFLAYER_6	NC_070797	21876	22301	Gordonia phage	3.96E-29	0.986111111
SEA_MINIFLAYER_6	NC_048045	36486	36875	Caulobacter phag	4.23E-28	0.923611111
SEA_MINIFLAYER_6	NC_019408	37623	38012	Caulobacter phag	6.00E-28	0.923611111
SEA_MINIFLAYER_6	NC_019407	36366	36755	Caulobacter phag	9.83E-28	0.923611111
SEA_MINIFLAYER_6	JX100812	36366	36755	Caulobacter phag	9.83E-28	0.923611111
SEA_MINIFLAYER_6	KY555142	37102	37491	Caulobacter phag	9.83E-28	0.923611111
SEA_MINIFLAYER_6	KY555143	37577	37966	Caulobacter phag	9.83E-28	0.923611111
SEA_MINIFLAYER_6	KY555145	39744	40133	Caulobacter phag	9.83E-28	0.923611111
SEA_MINIFLAYER_6	NC_048047	66794	67183	Caulobacter phag	1.37E-27	0.923611111
SEA_MINIFLAYER_6	NC_048048	66176	66565	Caulobacter phag	2.05E-27	0.923611111

SEA_MINIFLAYER_6	JX163858	170366	170755	Caulobacter phag	3.33E-27	0.923611111
SEA_MINIFLAYER_6	NC_019405	37058	37447	Caulobacter phag	3.33E-27	0.923611111
SEA_MINIFLAYER_6	KY555146	37102	37491	Caulobacter phag	3.33E-27	0.923611111
SEA_MINIFLAYER_6	KY555147	37101	37490	Caulobacter phag	3.33E-27	0.923611111
SEA_MINIFLAYER_6	KY555144	36975	37364	Caulobacter phag	3.33E-27	0.923611111
SEA_MINIFLAYER_6	NC_019411	36331	36720	Caulobacter phag	3.33E-27	0.923611111
SEA_MINIFLAYER_6	NC_019410	37178	37567	Caulobacter phag	3.33E-27	0.923611111
SEA_MINIFLAYER_6	JX100811	37178	37567	Caulobacter phag	3.33E-27	0.923611111
SEA_MINIFLAYER_6	MZ574432	68311	68697	Caulobacter phag	6.14E-27	0.916666667
SEA_MINIFLAYER_6	MZ574430	63534	63920	Caulobacter phag	6.90E-27	0.916666667
SEA_MINIFLAYER_17	MF358541	114859	115158	Streptomyces ph	2.87E-24	0.953271028
SEA_MINIFLAYER_17	MT684590	113490	113786	Streptomyces ph	4.85E-24	0.925233645
SEA_MINIFLAYER_17	ON970591	114550	114846	Streptomyces ph	4.85E-24	0.925233645
SEA_MINIFLAYER_17	MT897905	115124	115420	Streptomyces ph	4.85E-24	0.925233645
SEA_MINIFLAYER_17	NC_055809	115266	115565	Streptomyces ph	5.45E-24	0.953271028
SEA_MINIFLAYER_17	NC_029098	115421	115720	Streptomyces ph	5.45E-24	0.953271028
SEA_MINIFLAYER_17	MZ958750	116374	116673	Streptomyces ph	5.45E-24	0.953271028
SEA_MINIFLAYER_17	NC_042008	113635	113928	Streptomyces ph	6.61E-24	0.953271028
SEA_MINIFLAYER_17	NC_055842	114998	115285	Streptomyces ph	1.28E-23	0.897196262
SEA_MINIFLAYER_17	NC_042009	113689	113946	Streptomyces ph	3.42E-23	0.803738318
SEA_MINIFLAYER_17	NC_055813	113955	114212	Streptomyces ph	3.42E-23	0.803738318
SEA_MINIFLAYER_17	OK310502	114308	114565	Streptomyces ph	3.42E-23	0.803738318
SEA_MINIFLAYER_17	ON108650	114846	115103	Streptomyces ph	3.42E-23	0.803738318
SEA_MINIFLAYER_17	NC_042010	116064	116321	Streptomyces ph	3.42E-23	0.803738318
SEA_MINIFLAYER_17	NC_070782	115949	116254	Streptomyces ph	5.72E-23	0.943925234
SEA_MINIFLAYER_17	NC_042012	116737	116994	Streptomyces ph	5.94E-23	0.803738318
SEA_MINIFLAYER_17	NC_055791	117282	117539	Streptomyces ph	5.94E-23	0.803738318
SEA_MINIFLAYER_17	NC_055822	115577	115864	Streptomyces ph	6.81E-23	0.897196262
SEA_MINIFLAYER_17	NC_070781	116611	116868	Streptomyces ph	6.81E-23	0.803738318
SEA_MINIFLAYER_17	MK359332	112883	113173	Streptomyces ph	1.33E-22	0.953271028
SEA_MINIFLAYER_17	NC_048723	114704	114994	Streptomyces ph	1.68E-22	0.953271028
SEA_MINIFLAYER_17	MW507136	115091	115348	Streptomyces ph	2.08E-22	0.803738318
SEA_MINIFLAYER_17	MK460248	115566	115823	Streptomyces ph	2.08E-22	0.803738318
SEA_MINIFLAYER_17	NC_042011	115335	115592	Streptomyces ph	2.08E-22	0.803738318
SEA_MINIFLAYER_17	MN369743	115731	115988	Streptomyces ph	2.08E-22	0.803738318
SEA_MINIFLAYER_17	MF358542	116201	116458	Streptomyces ph	2.08E-22	0.803738318

SEA_MINIFLAYER_17	MK359351	112758	113048	Streptomyces ph	2.48E-22	0.953271028
SEA_MINIFLAYER_17	NC_048730	112807	113097	Streptomyces ph	2.48E-22	0.953271028
SEA_MINIFLAYER_17	OP021680	113244	113534	Streptomyces ph	2.48E-22	0.953271028
SEA_MINIFLAYER_17	MK801722	110090	110347	Streptomyces ph	2.78E-22	0.803738318
SEA_MINIFLAYER_17	MW291014	112059	112316	Streptomyces ph	2.78E-22	0.803738318
SEA_MINIFLAYER_17	MN428060	112543	112800	Streptomyces ph	2.78E-22	0.803738318
SEA_MINIFLAYER_17	MW507134	112157	112414	Streptomyces ph	2.78E-22	0.803738318
SEA_MINIFLAYER_17	ON260819	112263	112520	Streptomyces ph	2.78E-22	0.803738318
SEA_MINIFLAYER_17	MN369757	112740	112997	Streptomyces ph	2.78E-22	0.803738318
SEA_MINIFLAYER_17	MH576964	112236	112493	Streptomyces ph	2.78E-22	0.803738318
SEA_MINIFLAYER_17	ON260828	112721	112978	Streptomyces ph	2.78E-22	0.803738318
SEA_MINIFLAYER_17	NC_048724	112859	113116	Streptomyces ph	2.78E-22	0.803738318
SEA_MINIFLAYER_17	ON970579	113396	113653	Streptomyces ph	2.78E-22	0.803738318
SEA_MINIFLAYER_17	MN484599	113659	113916	Streptomyces ph	2.78E-22	0.803738318
SEA_MINIFLAYER_17	NC_048722	115947	116204	Streptomyces ph	2.78E-22	0.803738318
SEA_MINIFLAYER_17	NC_048721	115205	115462	Streptomyces ph	2.78E-22	0.803738318
SEA_MINIFLAYER_17	MN369750	114707	114964	Streptomyces ph	2.78E-22	0.803738318
SEA_MINIFLAYER_17	ON970590	114464	114721	Streptomyces ph	2.78E-22	0.803738318
SEA_MINIFLAYER_17	MW365952	95525	95800	Streptomyces ph	1.17E-19	0.859813084
SEA_MINIFLAYER_17	NC_048728	95727	96002	Streptomyces ph	1.17E-19	0.859813084
SEA_MINIFLAYER_17	ON456343	96172	96447	Streptomyces ph	1.17E-19	0.859813084
SEA_MINIFLAYER_17	MH590589	95935	96210	Streptomyces ph	1.17E-19	0.859813084
SEA_MINIFLAYER_17	MZ820095	94951	95226	Streptomyces ph	1.17E-19	0.859813084
SEA_MINIFLAYER_17	MT114162	97228	97494	Streptomyces ph	7.73E-19	0.831775701
SEA_MINIFLAYER_19	NC_055791	13269	14021	Streptomyces ph	8.56E-25	0.878980892
SEA_MINIFLAYER_19	NC_042012	12876	13628	Streptomyces ph	3.43E-24	0.878980892
SEA_MINIFLAYER_19	ON970590	14457	15224	Streptomyces ph	5.40E-24	0.863057325
SEA_MINIFLAYER_19	MW507134	14494	15261	Streptomyces ph	2.10E-23	0.863057325
SEA_MINIFLAYER_19	MN369757	14509	15276	Streptomyces ph	2.10E-23	0.863057325
SEA_MINIFLAYER_19	MH576964	14494	15261	Streptomyces ph	2.10E-23	0.863057325
SEA_MINIFLAYER_19	ON260819	14518	15285	Streptomyces ph	2.36E-23	0.863057325
SEA_MINIFLAYER_19	MK801722	12375	13142	Streptomyces ph	2.45E-23	0.863057325
SEA_MINIFLAYER_19	ON260828	14509	15276	Streptomyces ph	2.45E-23	0.863057325
SEA_MINIFLAYER_19	MN484599	14134	14901	Streptomyces ph	2.45E-23	0.863057325
SEA_MINIFLAYER_19	MN369750	14112	14879	Streptomyces ph	2.45E-23	0.863057325
SEA_MINIFLAYER_19	MW291014	14128	14895	Streptomyces ph	5.66E-23	0.863057325

SEA_MINIFLAYER_19	MN369743	12792	13574	Streptomyces ph	8.82E-23	0.904458599
SEA_MINIFLAYER_19	MW507136	13227	14009	Streptomyces ph	3.18E-22	0.904458599
SEA_MINIFLAYER_19	NC_042011	12925	13707	Streptomyces ph	3.18E-22	0.904458599
SEA_MINIFLAYER_19	MF358542	13321	14103	Streptomyces ph	3.18E-22	0.904458599
SEA_MINIFLAYER_19	MK359332	14322	15098	Streptomyces ph	4.42E-22	0.863057325
SEA_MINIFLAYER_19	NC_070781	14886	15704	Streptomyces ph	4.42E-22	0.904458599
SEA_MINIFLAYER_19	MK460248	13014	13796	Streptomyces ph	4.85E-22	0.904458599
SEA_MINIFLAYER_19	NC_048723	14198	14974	Streptomyces ph	6.62E-22	0.863057325
SEA_MINIFLAYER_19	NC_042008	13580	14371	Streptomyces ph	8.30E-22	0.878980892
SEA_MINIFLAYER_19	NC_048721	14118	14894	Streptomyces ph	1.39E-21	0.863057325
SEA_MINIFLAYER_19	NC_048724	14615	15391	Streptomyces ph	1.99E-21	0.863057325
SEA_MINIFLAYER_19	MN428060	14212	14988	Streptomyces ph	2.01E-21	0.863057325
SEA_MINIFLAYER_19	ON970579	14218	14994	Streptomyces ph	2.01E-21	0.863057325
SEA_MINIFLAYER_19	MK359351	14319	15095	Streptomyces ph	6.45E-21	0.863057325
SEA_MINIFLAYER_19	NC_048730	14298	15074	Streptomyces ph	6.45E-21	0.863057325
SEA_MINIFLAYER_19	OP021680	14322	15098	Streptomyces ph	6.45E-21	0.863057325
SEA_MINIFLAYER_19	NC_048722	13283	14053	Streptomyces ph	1.64E-20	0.866242038
SEA_MINIFLAYER_19	MZ958750	14043	14783	Streptomyces ph	1.99E-20	0.863057325
SEA_MINIFLAYER_19	NC_042009	12500	13240	Streptomyces ph	3.40E-20	0.863057325
SEA_MINIFLAYER_19	OK310502	13319	14059	Streptomyces ph	3.40E-20	0.863057325
SEA_MINIFLAYER_19	ON108650	13318	14058	Streptomyces ph	3.40E-20	0.863057325
SEA_MINIFLAYER_19	NC_042010	12491	13231	Streptomyces ph	7.85E-20	0.863057325
SEA_MINIFLAYER_19	NC_070782	15136	15852	Streptomyces ph	1.09E-19	0.824840764
SEA_MINIFLAYER_19	NC_055842	13640	14443	Streptomyces ph	2.01E-19	0.904458599
SEA_MINIFLAYER_19	NC_055813	13714	14454	Streptomyces ph	2.44E-19	0.863057325

:eome of MiniFlayer.

Supplementary Table 3. Results from the tBLASTn search querying the NCBI GenBank database, restricted to viruses (taxonomy ID: 10239), with

Query protein	Hit genome	Start position of the	End position of the	Phage name	E_value	Q_cov
CDS_3	NC_070830	6086	7540	Streptomyces phage KimJongPhill	1.05E-78	0.911828
CDS_3	NC_070829	5849	7294	Streptomyces phage Zuko	4.38E-78	0.911828
CDS_3	MH019215	4036	5361	Streptomyces phage Yara	1.11E-53	0.892473
CDS_3	MT897908	4522	5865	Streptomyces phage ShakeNBake	9.74E-48	0.909677
CDS_3	MK305891	4591	5892	Streptomyces phage Gibson	1.15E-47	0.87957
CDS_3	MZ648033	4591	5892	Streptomyces phage Rooney	1.15E-47	0.87957
CDS_3	MZ648036	4522	5865	Streptomyces phage Lizz	1.17E-47	0.909677
CDS_3	MT498053	4522	5865	Streptomyces phage PHTowN	1.21E-47	0.909677
CDS_3	MH019216	4335	5636	Streptomyces phage Wentworth	3.69E-45	0.87957
CDS_3	MT936332	4535	5848	Streptomyces phage phiRKBJ001	2.47E-44	0.888172
CDS_3	MT498037	4802	6115	Streptomyces phage Dryad	4.33E-43	0.87957
CDS_3	OP434449	6017	7351	Microbacterium phage OscarSo	1.20E-35	0.911828
CDS_3	KT997826	11574	12809	Uncultured Mediterranean phage uvDe	1.63E-30	0.896774
CDS_3	OP751148	27436	28698	Streptomyces phage Success	1.05E-20	0.946237
CDS_3	NC_070833	2413	3588	Arthrobacter phage Hestia	1.87E-18	0.860215
CDS_3	OP434463	2341	3516	Arthrobacter phage EvePickles	3.64E-18	0.860215
CDS_3	NC_070835	2334	3515	Arthrobacter phage Isolde	2.19E-17	0.864516
CDS_3	NC_070832	2341	3516	Arthrobacter phage Auxilium	2.85E-17	0.860215
CDS_3	NC_070831	2341	3516	Arthrobacter phage Richie	2.86E-17	0.860215
CDS_3	NC_047906	2289	3389	Streptomyces phage Rowa	5.37E-16	0.789247
CDS_3	ON970598	2341	3510	Arthrobacter phage Sakai	1.83E-15	0.860215
CDS_3	NC_070837	2340	3509	Arthrobacter phage Faja	1.83E-15	0.860215
CDS_3	OP434442	2340	3509	Arthrobacter phage RadFad	1.83E-15	0.860215
CDS_3	ON724017	2341	3510	Arthrobacter phage Gorpy	1.83E-15	0.860215
CDS_3	NC_070836	2433	3512	Arthrobacter phage Seahorse	1.90E-13	0.791398
CDS_3	NC_070834	2406	3587	Arthrobacter phage Persistence	2.56E-13	0.864516
CDS_3	NC_027391	1969	3120	Propionibacterium phage PHL041M10	6.54E-13	0.860215
CDS_3	KJ578761	1969	3120	Propionibacterium phage PHL041M10	6.54E-13	0.860215
CDS_3	MG820643	1970	3124	Propionibacterium phage pa35	8.75E-13	0.862366
CDS_3	MW161467	23414	24568	Cutibacterium phage PAVL34	1.98E-12	0.862366
CDS_3	MW161466	23506	24660	Cutibacterium phage PAVL33	1.98E-12	0.862366
CDS_3	MG820644	1965	3119	Propionibacterium phage pa33	2.92E-12	0.862366
CDS_3	MG820645	1965	3119	Propionibacterium phage pa3-SS3	2.92E-12	0.862366

CDS_3	NC_070828	2218	3639	Streptomyces phage Dubu	4.79E-11	0.963441
CDS_5	NC_070829	9385	10299	Streptomyces phage Zuko	1.49E-68	0.945338
CDS_5	NC_070830	9952	10866	Streptomyces phage KimJongPhill	1.12E-61	0.945338
CDS_5	MK305891	8998	9888	Streptomyces phage Gibson	7.72E-56	0.92926
CDS_5	MZ648033	8995	9885	Streptomyces phage Rooney	7.72E-56	0.92926
CDS_5	MT498053	8854	9804	Streptomyces phage PHTowN	1.32E-55	0.96463
CDS_5	MZ648036	8854	9804	Streptomyces phage Lizz	1.40E-55	0.96463
CDS_5	MT897908	8854	9804	Streptomyces phage ShakeNBake	1.40E-55	0.96463
CDS_5	MT498037	9422	10312	Streptomyces phage Dryad	5.92E-55	0.92926
CDS_5	MH019215	7955	8905	Streptomyces phage Yara	4.15E-52	0.96463
CDS_5	MH019216	8710	9600	Streptomyces phage Wentworth	5.34E-51	0.92926
CDS_5	OP434449	10611	11561	Microbacterium phage OscarSo	1.32E-49	0.945338
CDS_5	MT936332	8932	9822	Streptomyces phage phiRKBJ001	1.62E-48	0.92926
CDS_11	JX006077	69941	70147	Saccharomonospora phage PIS 136	4.98E-11	0.775281
CDS_15	NC_042008	13538	14368	Streptomyces phage Mildred21	4.22E-45	0.927673
CDS_15	MZ958750	13974	14783	Streptomyces phage Targaryen	2.57E-43	0.946541
CDS_15	MF358541	13437	14246	Streptomyces phage Warpy	7.50E-43	0.946541
CDS_15	NC_029098	13417	14226	Streptomyces phage Jay2Jay	7.50E-43	0.946541
CDS_15	ON970590	14376	15275	Streptomyces phage JimJam	1.22E-42	1
CDS_15	MK801722	12294	13193	Streptomyces phage Birchlyn	1.42E-42	1
CDS_15	ON260828	14428	15327	Streptomyces phage Quaran19	1.42E-42	1
CDS_15	MN484599	14053	14952	Streptomyces phage Wipeout	1.42E-42	1
CDS_15	MN369750	14031	14930	Streptomyces phage TomSawyer	1.42E-42	1
CDS_15	MW507134	14413	15312	Streptomyces phage Battuta	1.55E-42	1
CDS_15	MN369757	14428	15327	Streptomyces phage Bordeaux	1.55E-42	1
CDS_15	MH576964	14413	15312	Streptomyces phage Starbow	1.55E-42	1
CDS_15	MW291014	14047	14946	Streptomyces phage MindFlayer	1.69E-42	1
CDS_15	ON260819	14437	15336	Streptomyces phage SaltySpitoon	1.84E-42	1
CDS_15	NC_048723	14117	15025	Streptomyces phage LukeCage	2.68E-42	1
CDS_15	NC_048721	14037	14945	Streptomyces phage StarPlatinum	8.60E-42	1
CDS_15	NC_042010	12422	13231	Streptomyces phage Paradiddles	9.93E-42	0.946541
CDS_15	NC_042009	12431	13240	Streptomyces phage NootNoot	1.25E-41	0.946541
CDS_15	OK310502	13250	14059	Streptomyces phage Bartholomune	1.25E-41	0.946541
CDS_15	ON108650	13249	14058	Streptomyces phage Squillium	1.25E-41	0.946541
CDS_15	NC_055813	13645	14454	Streptomyces phage Braelyn	1.26E-41	0.946541
CDS_15	NC_070782	15043	15846	Streptomyces phage Coruscant	1.89E-41	0.927673

CDS_15	NC_055809	13227	14036	Streptomyces phage Evy	2.07E-41	0.946541
CDS_15	MN428060	14167	15039	Streptomyces phage IchabodCrane	2.35E-41	0.962264
CDS_15	ON970579	14173	15045	Streptomyces phage Spilled	2.35E-41	0.962264
CDS_15	NC_048724	14570	15442	Streptomyces phage Karimac	2.56E-41	0.962264
CDS_15	MK359351	14274	15146	Streptomyces phage BoomerJR	4.37E-41	0.962264
CDS_15	NC_048730	14253	15125	Streptomyces phage Yaboi	4.37E-41	0.962264
CDS_15	OP021680	14277	15149	Streptomyces phage Stanimal	4.37E-41	0.962264
CDS_15	MK359332	14277	15149	Streptomyces phage Genie2	6.92E-41	0.962264
CDS_15	MN369743	12765	13574	Streptomyces phage Tribute	1.78E-40	0.946541
CDS_15	MK460248	12987	13796	Streptomyces phage Teutsch	2.38E-40	0.946541
CDS_15	NC_055791	13209	14018	Streptomyces phage EGole	3.62E-40	0.946541
CDS_15	NC_042012	12816	13625	Streptomyces phage Samisti12	3.98E-40	0.946541
CDS_15	MW507136	13200	14009	Streptomyces phage Cross	5.36E-40	0.946541
CDS_15	NC_042011	12898	13707	Streptomyces phage Peebs	5.36E-40	0.946541
CDS_15	MF358542	13294	14103	Streptomyces phage Sushi23	5.36E-40	0.946541
CDS_15	NC_055822	13628	14416	Streptomyces phage Daubenski	6.61E-40	0.927673
CDS_15	MT684590	13821	14636	Streptomyces phage LilMartin	9.78E-40	0.927673
CDS_15	MT897905	13822	14637	Streptomyces phage MulchMansion	9.78E-40	0.927673
CDS_15	ON970591	13874	14689	Streptomyces phage Angela	1.01E-39	0.927673
CDS_15	NC_048722	13208	14041	Streptomyces phage Wofford	1.61E-39	0.943396
CDS_15	NC_055842	13631	14443	Streptomyces phage Bmoc	2.94E-38	0.927673
CDS_15	NC_070781	14877	15695	Streptomyces phage Tomas	3.54E-36	0.918239
CDS_15	NC_070786	102133	103011	Streptomyces phage Muntaha	3.56E-19	0.959119
CDS_15	NC_070785	102368	103246	Streptomyces phage Wakanda	4.37E-19	0.959119
CDS_15	MK620896	102269	103114	Streptomyces phage Circinus	1.85E-18	0.927673
CDS_15	NC_042105	102462	103307	Streptomyces phage BillNye	1.85E-18	0.927673
CDS_15	MH744420	51395	52285	Streptomyces phage Kromp	4.42E-13	0.930818
CDS_20_trunc	MT498037	2959	3747	Streptomyces phage Dryad	7.46E-68	0.78481
CDS_20_trunc	MK305891	2887	3648	Streptomyces phage Gibson	2.48E-66	0.756329
CDS_20_trunc	MZ648033	2887	3648	Streptomyces phage Rooney	2.55E-66	0.756329
CDS_20_trunc	MH019216	2632	3393	Streptomyces phage Wentworth	1.95E-65	0.756329
CDS_20_trunc	MT498053	2843	3604	Streptomyces phage PHTowN	2.57E-65	0.756329
CDS_20_trunc	MZ648036	2843	3604	Streptomyces phage Lizz	2.57E-65	0.756329
CDS_20_trunc	MT897908	2843	3604	Streptomyces phage ShakeNBake	2.57E-65	0.756329

the proteome of MulchRoom.

Supplementary Table 4. Results from the BLASTP search querying the proteomes of phage satellites (de Sousa JAM, Fillol-Salom A, Penadés JR, Rocha EP(

	qseqid	sseqid	pident	length	mismatch	gapopen	qstart
0	SEA_MINIFLAYER_3	MYSP019.0321.00001.C001.PICI.TypeB.variant0001.Set2_10	26.841	421	274	14	15
1	SEA_MINIFLAYER_3	MYMA004.0321.00001.C001.PICI.TypeB.variant0001.Set3_07	26.005	423	282	14	13
2	SEA_MINIFLAYER_3	MYAB001.0321.00046.C001.PICI.TypeB.variant0001.Set1_09	27.546	432	270	17	5
3	SEA_MINIFLAYER_3	MYDI001.0321.00001.C001.PICI.TypeB.variant0001.Set1_10	27.211	441	276	15	5
4	SEA_MINIFLAYER_3	MYSH002.0321.00001.C001.PICI.TypeB.variant0001.Set1_07	26.905	420	278	13	24
6	SEA_MINIFLAYER_3	GOSP001.0321.00001.C001.PICI.TypeB.variant0001.Set1_11	24.711	433	281	15	5
7	SEA_MINIFLAYER_3	DESP024.0321.00001.C001.PICI.TypeB.variant0001.Set1_06	25.792	442	288	15	4
9	SEA_MINIFLAYER_3	MYLI002.0321.00002.C001.PICI.TypeB.variant0001.Set1_09	25	432	287	12	16
10	SEA_MINIFLAYER_3	MYAB001.0321.00045.C001.PICI.TypeB.variant0001.Set5_09	27.546	432	270	17	5
11	SEA_MINIFLAYER_3	MYSP009.0321.00001.C001.PICI.TypeB.variant0001.Set1_10	26.914	431	277	15	13
13	SEA_MINIFLAYER_3	MYCH002.0321.00002.C001.PICI.TypeB.variant0001.Set1_08	25.344	363	231	13	85
14	SEA_MINIFLAYER_3	BRAU001.0321.00001.C001.PICI.TypeB.variant0001.Set4_11	27.65	434	278	16	3
15	SEA_MINIFLAYER_3	MYPA001.0321.00005.C001.PICI.TypeB.variant0001.Set1_06	26.914	431	266	16	13
17	SEA_MINIFLAYER_3	MYCH002.0321.00001.C001.PICI.TypeB.variant0001.Set1_08	25.344	363	231	13	85
18	SEA_MINIFLAYER_3	GOPH001.0321.00001.C001.PICI.TypeB.variant0001.Set1_12	25.814	430	265	17	16
19	SEA_MINIFLAYER_3	ACJE001.0321.00004.C001.PICI.TypeB.variant0001.Set1_07	24.138	406	283	12	24
20	SEA_MINIFLAYER_3	MYFO001.0321.00001.C001.PICI.TypeB.variant0001.Set2_06	26.392	413	275	13	13
21	SEA_MINIFLAYER_3	MYAL002.0321.00001.C001.PICI.TypeB.variant0001.Set2_09	25.576	434	295	13	1

:. Identification and characterization of thousands of bacteriophage satellites across bacteria. Nucleic Acids Res 2023; gkad123) with the proteome of Mini

qend	sstart	send	evalue	bitscore	qcov
412	18	427	3.34E-31	114	0.886161
415	6	417	4.78E-28	104	0.897321
417	15	422	6.63E-31	112	0.919643
416	14	438	2.43E-37	131	0.917411
423	17	427	1.21E-34	123	0.890625
423	15	416	2.79E-31	114	0.933036
423	9	432	9.10E-32	115	0.935268
423	9	427	7.36E-33	118	0.908482
417	15	422	6.63E-31	112	0.919643
423	14	426	1.04E-31	115	0.915179
423	80	426	1.40E-24	94.4	0.754464
421	6	418	3.19E-36	128	0.933036
414	22	432	9.84E-31	112	0.895089
423	80	426	1.40E-24	94.4	0.754464
422	17	415	1.01E-29	109	0.90625
414	52	447	1.48E-27	103	0.870536
406	21	423	1.48E-26	100	0.877232
414	1	426	3.91E-33	119	0.921875

Flayer.

Supplementary Table 5. Results from the BLASTP search querying the proteomes of phage satellites (de Sousa JAM, Fillol-Salom A, Penadés JR, Rocha

	qseqid	sseqid	pident	length	mismatch	gapopen	qstart	qend
0	CDS_3	ACJE001.0321.00004.C001.PICI.TypeB.variant0001.Set1_07	22.837	416	270	14	24	418
1	CDS_3	GOSP001.0321.00001.C001.PICI.TypeB.variant0001.Set1_11	25.442	452	287	18	1	437
2	CDS_3	GOSP003.0321.00001.C001.PICI.TypeB.variant0001.Set1_08	25.307	407	264	13	1	384
3	CDS_3	MYSH002.0321.00001.C001.PICI.TypeB.variant0001.Set1_07	24.731	465	267	16	16	440
4	CDS_3	GOPH001.0321.00001.C001.PICI.TypeB.variant0001.Set1_12	25.57	395	224	16	1	373
5	CDS_3	BRAU001.0321.00001.C001.PICI.TypeB.variant0001.Set4_11	25.319	470	291	14	2	455
6	CDS_3	DESP024.0321.00001.C001.PICI.TypeB.variant0001.Set1_06	25	408	262	15	24	413
7	CDS_3	MYFO001.0321.00001.C001.PICI.TypeB.variant0001.Set2_06	25.056	447	273	16	3	420
8	CDS_3	GOPH001.0321.00001.C001.PICI.TypeB.variant0001.Set2_08	23.961	409	267	12	23	407
9	CDS_3	MYMA004.0321.00001.C001.PICI.TypeB.variant0001.Set3_07	26.398	447	262	17	16	431
11	CDS_3	MYCH002.0321.00001.C001.PICI.TypeB.variant0001.Set1_08	23.414	457	298	15	1	436
12	CDS_3	MYCH002.0321.00002.C001.PICI.TypeB.variant0001.Set1_08	23.414	457	298	15	1	436
13	CDS_3	MYAB001.0321.00045.C001.PICI.TypeB.variant0001.Set5_09	24.123	456	287	17	1	431
14	CDS_3	MYAB001.0321.00046.C001.PICI.TypeB.variant0001.Set1_09	24.123	456	287	17	1	431
15	CDS_3	MYPA001.0321.00005.C001.PICI.TypeB.variant0001.Set1_06	24.569	464	273	19	16	445
17	CDS_3	MYSP009.0321.00001.C001.PICI.TypeB.variant0001.Set1_10	23.991	446	296	14	4	433
18	CDS_3	MYDI001.0321.00001.C001.PICI.TypeB.variant0001.Set1_10	23.312	459	290	17	1	425
19	CDS_3	MYSP019.0321.00001.C001.PICI.TypeB.variant0001.Set2_10	25.328	458	272	17	1	425
20	CDS_3	MYLI002.0321.00002.C001.PICI.TypeB.variant0001.Set1_09	25.277	451	277	15	16	437
21	CDS_3	MYAL002.0321.00001.C001.PICI.TypeB.variant0001.Set2_09	23.956	455	282	16	1	425

EPC. Identification and characterization of thousands of bacteriophage satellites across bacteria. Nucleic Acids Res 2023; gkad123) with the proteome of M

sstart	send	evalue	bitscore	qcov
52	437	5.43E-21	84	0.847312
1	417	2.62E-20	81.6	0.937634
1	390	1.32E-14	63.9	0.823656
9	430	9.91E-25	95.1	0.911828
1	347	3.72E-19	78.2	0.8
5	430	3.19E-26	99.8	0.974194
26	407	9.17E-17	70.9	0.836559
10	423	1.58E-22	88.6	0.896774
25	413	5.47E-15	65.1	0.825806
9	419	2.03E-21	85.1	0.892473
1	426	8.66E-16	67.8	0.935484
1	426	8.66E-16	67.8	0.935484
1	422	4.60E-22	87	0.924731
1	422	4.60E-22	87	0.924731
25	445	3.42E-21	84.7	0.922581
5	423	1.32E-22	89	0.922581
4	434	5.23E-22	87	0.911828
7	427	4.52E-26	100	0.911828
9	428	1.33E-24	94.7	0.905376
4	424	3.35E-22	87.8	0.911828

ulchRoom.

Supplementary Table 6. PICI sequences selected for this study, based on relatedness to the proteomes of the phage satellites MiniFlayer and Mul

File_name	Prophage_start	Prophage_end	Genome accession	Strain
ACER001.0321.00001.C001.PICI.TypeB.variant0001.Set1.prt	197540	203855	NZ_CP022752.1	YIM 90600
ACIN001.0321.00001.C001.PICI.TypeB.variant0002.Set1.prt	1415366	1431157	NC_016077.1	RyC-MR95
ACIN004.0321.00004.C001.PICI.TypeB.variant0003.Set1.prt	2582608	2590922	NZ_CP045135.1	XG01
ACJE001.0321.00004.C001.PICI.TypeB.variant0001.Set1.prt	1916050	1924794	NZ_LR134473.1	NCTC13652
ACSP027.0321.00001.C001.PICI.TypeB.variant0001.Set1.prt	5398565	5417226	NZ_CP025030.1	DSM 23806
ALDE001.0321.00004.C001.PICI.TypeB.variant0002.Set1.prt	3588708	3596602	NC_015422.1	K601
ALEH001.0321.00001.C001.PICI.TypeB.variant0001.Set1.prt	878948	887244	NC_008340.1	MLHE-1
ALSP012.0321.00001.C001.PICI.TypeB.variant0001.Set1.prt	1272697	1281928	NZ_CP049259.1	BO-6
AMBU001.0321.00001.C001.PICI.TypeB.variant0002.Set1.prt	16777	22895	NZ_CP048649.1	DSM 103574
ANCE001.0321.00001.C001.PICI.TypeB.variant0002.Set2.prt	442706	450243	NZ_AP023367.1	SN021
ARCI001.0321.00001.C001.PICI.TypeB.variant0002.Set1.prt	2443539	2451143	CP053688.1	NEB 577
ARNA001.0321.00001.C001.PICI.TypeB.variant0001.Set1.prt	717050	731782	NZ_CP038613.1	FIN
AZPU001.0321.00001.C001.PICI.TypeB.variant0001.Set1.prt	1771614	1778214	CP025682.1	SY39
BACE001.0321.00004.C001.PICI.TypeB.variant0003.Set2.prt	4819859	4833306	NZ_CP009596.1	3a
BACE001.0321.00005.C001.PICI.TypeB.variant0003.Set2.prt	93656	107103	NZ_CP009605.1	S2-8
BACE001.0321.00044.C001.PICI.TypeB.variant0003.Set2.prt	3589019	3602466	NZ_CP053991.1	FDAARGOS_781
BACE001.0321.00045.C001.PICI.TypeB.variant0003.Set2.prt	4346503	4359950	NZ_CP053997.1	FDAARGOS_780
BASP059.0321.00001.C001.PICI.TypeB.variant0001.Set2.prt	1803052	1824663	NZ_CP059725.1	HY038
BATH001.0321.00012.C001.PICI.TypeB.variant0002.Set1.prt	2064316	2071862	NZ_CP015350.1	MYBT18246
BIAN002.0321.00001.C001.PICI.TypeB.variant0001.Set1.prt	1362863	1370642	NZ_CP007755.1	RH
BIAN002.0321.00002.C001.PICI.TypeB.variant0001.Set1.prt	1367953	1375732	NZ_CP010433.1	A6
BIAN002.0321.00003.C001.PICI.TypeB.variant0001.Set1.prt	1367949	1375728	NZ_CP017098.1	BL3
BIAN002.0321.00004.C001.PICI.TypeB.variant0001.Set1.prt	1367953	1375732	NZ_CP010433.1	A6
BIAN002.0321.00005.C001.PICI.TypeB.variant0001.Set1.prt	1307763	1315542	NZ_CP042940.1	B06
BIAN002.0321.00006.C001.PICI.TypeB.variant0001.Set1.prt	1369714	1377493	NZ_CP047190.1	Probio-M8
BIAN002.0321.00007.C001.PICI.TypeB.variant0001.Set1.prt	1367743	1375522	NZ_CP045589.1	TK-J6A
BIAN002.0321.00008.C001.PICI.TypeB.variant0001.Set1.prt	1367953	1375732	NZ_CP010433.1	A6
BIAN002.0321.00009.C001.PICI.TypeB.variant0001.Set1.prt	1362299	1370078	NZ_CP009045.1	BF052
BIAN002.0321.00010.C001.PICI.TypeB.variant0001.Set1.prt	1870035	1877814	NZ_CP022724.1	S7
BIAN002.0321.00011.C001.PICI.TypeB.variant0001.Set1.prt	796646	804425	NZ_CP031703.1	IDCC4301
BIAN002.0321.00012.C001.PICI.TypeB.variant0001.Set1.prt	1367807	1375586	NZ_CP031154.1	HN019
BIAN002.0321.00013.C001.PICI.TypeB.variant0001.Set1.prt	1367947	1375726	NZ_CP069248.1	H1
BIAN002.0321.00014.C001.PICI.TypeB.variant0001.Set1.prt	1367946	1375725	NZ_CP069249.1	H3

BIAN002.0321.00017.C001.PICI.TypeB.variant0001.Set1.prt	971355	979134	NC_011835.1	AD011
BIAN002.0321.00018.C001.PICI.TypeB.variant0001.Set1.prt	1367826	1375605	NC_017214.2	BB-12
BIAN002.0321.00019.C001.PICI.TypeB.variant0001.Set1.prt	1367951	1375730	NC_017217.1	V9
BIAN002.0321.00020.C001.PICI.TypeB.variant0001.Set1.prt	1362448	1370227	NC_012814.1	Bl-04; ATCC SD5219
BIAN002.0321.00022.C001.PICI.TypeB.variant0001.Set1.prt	1362503	1370282	NC_017867.1	Bi-07
BIAN002.0321.00023.C001.PICI.TypeB.variant0001.Set1.prt	1367314	1375093	NC_017215.1	CNCM I-2494
BIAN002.0321.00024.C001.PICI.TypeB.variant0001.Set1.prt	1362308	1370087	NC_017216.2	BLC1
BIAN002.0321.00026.C001.PICI.TypeB.variant0001.Set1.prt	1362499	1370278	NC_017866.1	B420
BIAN002.0321.00027.C001.PICI.TypeB.variant0001.Set1.prt	1362362	1370141	NC_021593.1	Bl12
BIAN002.0321.00028.C001.PICI.TypeB.variant0001.Set1.prt	1362413	1370192	NZ_CP007522.1	KLDS 2.0603
BRAU001.0321.00001.C001.PICI.TypeB.variant0001.Set4.prt	2118469	2127441	NZ_CP017150.1	SMQ-1335
BUCO001.0321.00001.C002.PICI.TypeB.variant0001.Set1.prt	3194516	3202980	NZ_CP009744.1	MS14
BUDI001.0321.00001.C002.PICI.TypeB.variant0001.Set1.prt	1693155	1713887	NZ_CP013363.1	RF2-non-BP9
BULA001.0321.00002.C001.PICI.TypeB.variant0002.Set1.prt	2577196	2584311	NZ_CP013404.1	FL-7-5-30-S1-D0
BUPS002.0321.00002.C001.PICI.TypeB.variant0004.Set1.prt	1301323	1314260	NZ_CP009128.1	BSR
BUPS002.0321.00016.C001.PICI.TypeB.variant0001.Set1.prt	1936838	1951640	NZ_CP016638.1	M1
BUPS002.0321.00017.C001.PICI.TypeB.variant0001.Set1.prt	1935718	1950520	NZ_CP016636.1	MS
BUPS002.0321.00055.C001.PICI.TypeB.variant0004.Set1.prt	2430965	2443902	NZ_CP033702.1	FDAARGOS_594
BUPS002.0321.00084.C001.PICI.TypeB.variant0004.Set1.prt	145426	158363	NZ_CP009298.1	406E
BUSP007.0321.00001.C001.PICI.TypeB.variant0001.Set2.prt	1565985	1584785	NZ_CP013417.1	MSMB0266
BUSP018.0321.00001.C001.PICI.TypeB.variant0003.Set3.prt	790061	812007	NZ_CP033076.1	3AFRM03
BUTH001.0321.00004.C001.PICI.TypeB.variant0002.Set2.prt	1349723	1357000	NZ_CP013409.1	2002721121
BUTH001.0321.00008.C001.PICI.TypeB.variant0002.Set2.prt	2564207	2571484	NZ_CP020392.1	FDAARGOS_238
BUTH001.0321.00019.C001.PICI.TypeB.variant0002.Set1.prt	1663964	1671241	NZ_CP004089.1	H0587
CHMI001.0321.00001.C001.PICI.TypeB.variant0002.Set2.prt	2657969	2667260	CP029256.1	DSM 22607
CHPH002.0321.00002.C001.PICI.TypeB.variant0002.Set1.prt	206045	213472	unknown	unknown
CIAM001.0321.00007.C001.PICI.TypeB.variant0001.SetR1.prt	5324498	5332239	NZ_CP011132.1	Y19
CIFR005.0321.00052.C001.PICI.TypeB.variant0001.SetR1.prt	448508	456161	NZ_CP056289.1	RHBSTW-00858
CIFR005.0321.00053.C001.PICI.TypeB.variant0001.SetR1.prt	448508	456161	NZ_CP056289.1	RHBSTW-00858
CIFR005.0321.00101.C001.PICI.TypeB.variant0001.SetR1.prt	4605676	4613464	NZ_CP054278.1	IDR1900015725-01-02
CLBO002.0321.00041.C001.PICI.TypeB.variant0001.Set1.prt	148239	154991	NC_010723.1	Alaska E43
CLBO002.0321.00049.C001.PICI.TypeB.variant0002.Set2.prt	3001928	3008465	NZ_CP006903.1	202F
CLCE001.0321.00001.C001.PICI.TypeB.variant0002.Set1.prt	4812661	4837828	NC_014393.1	743B
CLKL001.0321.00002.C001.PICI.TypeA.Set3.prt	3003379	3014942	NC_009706.1	DSM 555
CLKL001.0321.00003.C001.PICI.TypeA.Set3.prt	2934881	2946444	NC_011837.1	NBRC 12016
CLMI001.0321.00013.C001.PICI.TypeB.variant0001.Set1.prt	2026112	2033979	NZ_CP012573.1	PF008

CLNO001.0321.00002.C001.PICI.TypeA.Set1.prt	2515992	2523014	NC_008593.1	NT
CLPO001.0321.00001.C001.PICI.TypeB.variant0002.Set1.prt	1258524	1268417	NZ_CP016757.1	CL-84 (T)
CLSP004.0321.00001.C001.PICI.TypeB.variant0001.Set1.prt	153736	162895	NZ_CP025746.1	CT4
COAM002.0321.00001.C001.PICI.TypeB.variant0001.Set1.prt	876985	884316	NZ_CP065628.1	FDAARGOS_938
COAM002.0321.00002.C001.PICI.TypeB.variant0001.Set1.prt	991653	998984	NZ_CP066023.1	FDAARGOS_991
COJE001.0321.00001.C001.PICI.TypeB.variant0001.Set2.prt	2175736	2183659	NZ_CP022054.2	FDAARGOS_328
COJE001.0321.00002.C001.PICI.TypeB.variant0001.Set1.prt	291374	298700	NZ_CP033784.1	FDAARGOS_574
COPS002.0321.00001.C001.PICI.TypeB.variant0001.Set1.prt	515192	521873	NZ_CP033898.1	812CH
COSP009.0321.00001.C001.PICI.TypeB.variant0002.Set1.prt	2732201	2737213	NZ_CP028924.1	Arc7-D
COST002.0321.00001.C001.PICI.TypeB.variant0001.Set3.prt	1950912	1960856	NZ_CP014279.1	ATCC 6872
COST002.0321.00003.C001.PICI.TypeB.variant0001.Set1.prt	1773576	1783520	NZ_CP019963.1	LMG 21670
COUL001.0321.00009.C001.PICI.TypeB.variant0001.Set1.prt	1799378	1806744	NZ_CP046863.1	MRi49
COUL001.0321.00010.C001.PICI.TypeB.variant0001.Set1.prt	1874065	1881431	NZ_CP054583.1	LIV-14050
CRSP004.0321.00001.C001.PICI.TypeB.variant0001.Set2.prt	2573983	2581348	NZ_CP067087.1	YJ47
DEAC006.0321.00003.C001.PICI.TypeB.variant0001.Set1.prt	2802532	2809780	NZ_CP065668.1	FDAARGOS_909
DESP024.0321.00001.C001.PICI.TypeB.variant0001.Set1.prt	2557365	2566035	NZ_CP060292.1	PAMC28757
DEVU001.0321.00001.C001.PICI.TypeB.variant0001.SetR1.prt	223208	247002	NC_002937.3	Hildenborough
DEVU001.0321.00001.C001.PICI.TypeB.variant0001.SetR2.prt	2265579	2293843	NC_002937.3	Hildenborough
DEVU001.0321.00004.C001.PICI.TypeB.variant0001.SetR1.prt	2269100	2297364	NC_017310.1	RCH1
DISP008.0321.00001.C001.PICI.TypeB.variant0001.Set1.prt	3096778	3103190	NZ_CP027238.1	W5195
EPMO001.0321.00002.P002.PICI.TypeB.variant0002.Set1.prt	822175	835606	NZ_CP015231.1	F1926
ERLI001.0321.00001.C001.PICI.TypeB.variant0001.Set1.prt	2108279	2115624	NZ_CP017057.1	DSM 8509
ESCO001.0321.00009.C001.PICI.TypeB.variant0003.Set1.prt	805753	815467	NZ_CP009106.2	94-3024
ESCO001.0321.00026.C001.PICI.TypeA.Set1.prt	4947859	4957089	NZ_CP007275.1	O18
ESCO001.0321.00035.C001.PICI.TypeA.Set1.prt	3116717	3125946	NZ_CP012633.1	SF-166
ESCO001.0321.00051.C001.PICI.TypeA.Set1.prt	5314262	5324161	NZ_CP015020.1	28RC1
ESCO001.0321.00057.C001.PICI.TypeA.Set1.prt	4462843	4472824	NZ_CP015240.1	2011C-3911
ESCO001.0321.00058.C001.PICI.TypeA.Set1.prt	4403632	4413533	NZ_CP015241.1	2013C-4465
ESCO001.0321.00065.C001.PICI.TypeA.Set1.prt	2895825	2904859	NZ_CP015229.1	06-00048
ESCO001.0321.00065.C001.PICI.TypeB.variant0001.Set2.prt	3963521	3971912	NZ_CP015229.1	06-00048
ESCO001.0321.00066.C001.PICI.TypeA.Set1.prt	1046261	1055188	NZ_CP015228.1	09-00049
ESCO001.0321.00069.C001.PICI.TypeA.Set1.prt	1137266	1146193	NZ_CP013663.1	GB089
ESCO001.0321.00069.C001.PICI.TypeB.variant0001.Set2.prt	2769845	2778764	NZ_CP013663.1	GB089
ESCO001.0321.00075.C001.PICI.TypeA.Set1.prt	2637331	2647110	NZ_CP012693.1	FORC_028
ESCO001.0321.00075.C001.PICI.TypeA.Set3.prt	1826142	1834403	NZ_CP012693.1	FORC_028
ESCO001.0321.00092.C001.PICI.TypeA.Set2.prt	1537286	1547185	NZ_CP018237.1	

ESCO001.0321.00094.C001.PICI.TypeA.Set1.prt	1060554	1069024	NZ_CP018245.1		472
ESCO001.0321.00094.C001.PICI.TypeA.Set4.prt	2135951	2145849	NZ_CP018245.1		472
ESCO001.0321.00095.C001.PICI.TypeA.Set1.prt	1547693	1557592	NZ_CP018243.1		350
ESCO001.0321.00096.C001.PICI.TypeA.Set1.prt	1058979	1067387	unknown	unknown	
ESCO001.0321.00096.C001.PICI.TypeA.Set2.prt	1534702	1544601	unknown	unknown	
ESCO001.0321.00097.C001.PICI.TypeA.SetR1.prt	1567652	1586408	NZ_CP018247.1		7784
ESCO001.0321.00098.C001.PICI.TypeA.Set1.prt	1589823	1599722	NZ_CP018241.1		319
ESCO001.0321.00099.C001.PICI.TypeA.Set1.prt	1521305	1531204	NZ_CP018250.1		10671
ESCO001.0321.00113.C001.PICI.TypeB.variant0002.Set1.prt	2186601	2195957	NZ_CP010122.1	C5	
ESCO001.0321.00156.C001.PICI.TypeB.variant0001.Set1.prt	1413796	1422303	NZ_CP019778.1	NCTC86	
ESCO001.0321.00180.C001.PICI.TypeA.Set1.prt	3614838	3623238	NZ_CP020106.1	13E0780	
ESCO001.0321.00191.C001.PICI.TypeA.Set2.prt	1403080	1412980	NZ_CP021335.1	95JB1	
ESCO001.0321.00192.C001.PICI.TypeA.Set2.prt	1403080	1412980	NZ_CP021335.1	95JB1	
ESCO001.0321.00196.C001.PICI.TypeA.Set1.prt	679311	688583	NZ_CP019560.1	KSC1031	
ESCO001.0321.00201.C001.PICI.TypeA.Set1.prt	2484818	2494050	NZ_CP034966.1	WCHEC020032	
ESCO001.0321.00229.C001.PICI.TypeA.Set1.prt	907410	916641	NZ_CP021288.1	PA45B	
ESCO001.0321.00231.C001.PICI.TypeA.Set3.prt	1508201	1517406	NZ_CP023349.1	ETEC-2264	
ESCO001.0321.00260.C001.PICI.TypeA.Set1.prt	2648928	2658222	NZ_CP024830.1	CREC-532	
ESCO001.0321.00263.C001.PICI.TypeA.Set1.prt	3767602	3776896	CP024815.1	CREC-629	
ESCO001.0321.00263.C001.PICI.TypeA.Set3.prt	3788572	3797866	CP024815.1	CREC-629	
ESCO001.0321.00263.C001.PICI.TypeA.Set4.prt	3799059	3808353	CP024815.1	CREC-629	
ESCO001.0321.00263.C001.PICI.TypeA.Set5.prt	3809546	3818840	CP024815.1	CREC-629	
ESCO001.0321.00263.C001.PICI.TypeA.Set6.prt	3820033	3829327	NZ_CP024815.1	CREC-629	
ESCO001.0321.00263.C001.PICI.TypeB.variant0002.Set2.prt	3778089	3787379	CP024815.1	CREC-629	
ESCO001.0321.00278.C001.PICI.TypeA.Set1.prt	2927152	2937051	NZ_CP024134.1	14EC017	
ESCO001.0321.00290.C001.PICI.TypeA.Set1.prt	1599999	1608259	NZ_CP023673.1	SMN013SH2	
ESCO001.0321.00308.C001.PICI.TypeB.variant0003.Set1.prt	759912	769233	NZ_CP026755.1	AR_0077	
ESCO001.0321.00309.C001.PICI.TypeB.variant0001.SetR1.prt	2004425	2013193	NZ_CP019273.1	13P477T	
ESCO001.0321.00310.C001.PICI.TypeA.Set1.prt	4897740	4906985	NZ_CP019243.1	Combat2C1	
ESCO001.0321.00319.C001.PICI.TypeA.Set2.prt	1676812	1684702	NZ_CP025318.1	FORC_042	
ESCO001.0321.00351.C001.PICI.TypeB.variant0003.Set1.prt	3351703	3361084	NZ_CP027219.1	2015C-3163	
ESCO001.0321.00352.C001.PICI.TypeA.Set1.prt	3345202	3354137	NZ_CP027221.1	2015C-3101	
ESCO001.0321.00353.C001.PICI.TypeA.Set1.prt	658676	667611	NZ_CP027307.1	2015C-3108	
ESCO001.0321.00354.C001.PICI.TypeA.Set1.prt	3740921	3750776	NZ_CP027310.1	2014C-4135	
ESCO001.0321.00357.C001.PICI.TypeA.Set1.prt	854201	863976	NZ_CP027338.1	2014C-3051	
ESCO001.0321.00357.C001.PICI.TypeA.Set2.prt	2059785	2067973	NZ_CP027338.1	2014C-3051	

ESCO001.0321.00358.C001.PICI.TypeA.Set2.prt	4714499	4722687	NZ_CP027340.1	2015C-3121	
ESCO001.0321.00359.C001.PICI.TypeA.Set1.prt	2257109	2266045	NZ_CP027347.1	2013C-4361	
ESCO001.0321.00361.C001.PICI.TypeA.Set2.prt	1425321	1435100	NZ_CP027390.1	2015C-4944	
ESCO001.0321.00361.C001.PICI.TypeA.Set4.prt	124794	132983	NZ_CP027390.1	2015C-4944	
ESCO001.0321.00362.C001.PICI.TypeA.Set1.prt	368443	378222	NZ_CP027442.1	2013C-3252	
ESCO001.0321.00362.C001.PICI.TypeA.Set2.prt	1524704	1532893	NZ_CP027442.1	2013C-3252	
ESCO001.0321.00364.C001.PICI.TypeB.variant0001.Set1.prt	52316	60474	NZ_CP027452.1	2014C-3338	
ESCO001.0321.00366.C001.PICI.TypeA.Set1.prt	511528	519787	NZ_CP027472.1	2014C-3050	
ESCO001.0321.00367.C001.PICI.TypeA.Set1.prt	2579937	2588406	NZ_CP027548.1	2014C-3061	
ESCO001.0321.00367.C001.PICI.TypeA.Set2.prt	2191797	2201573	NZ_CP027548.1	2014C-3061	
ESCO001.0321.00369.C001.PICI.TypeA.Set1.prt	1259662	1269878	NZ_CP027371.1	2015C-3905	
ESCO001.0321.00369.C001.PICI.TypeA.Set2.prt	1756316	1765777	NZ_CP027371.1	2015C-3905	
ESCO001.0321.00370.C001.PICI.TypeB.variant0001.Set1.prt	940743	949458	NZ_CP027437.1	2012C-4221	
ESCO001.0321.00372.C001.PICI.TypeA.Set1.prt	4469047	4479321	NZ_CP027449.1	2014C-3097	
ESCO001.0321.00373.C001.PICI.TypeA.Set1.prt	1396463	1404651	NZ_CP027555.1	2013C-3513	
ESCO001.0321.00374.C001.PICI.TypeA.Set1.prt	1436222	1446475	NZ_CP027579.1	2013C-4282	
ESCO001.0321.00378.C001.PICI.TypeA.Set3.prt	3535250	3545029	NZ_CP027599.1	97-3250	
ESCO001.0321.00378.C001.PICI.TypeA.Set4.prt	2053428	2061687	NZ_CP027599.1	97-3250	
ESCO001.0321.00381.C001.PICI.TypeA.Set1.prt	4995508	5004444	NZ_CP027573.1	2013C-4081	
ESCO001.0321.00382.C001.PICI.TypeA.Set2.prt	4445272	4453465	NZ_CP027577.1	2013C-4225	
ESCO001.0321.00383.C001.PICI.TypeA.Set3.prt	506254	514510	NZ_CP027582.1	2013C-4538	
ESCO001.0321.00386.C001.PICI.TypeA.Set1.prt	1405202	1415419	NZ_CP027312.1	2013C-3181	
ESCO001.0321.00388.C001.PICI.TypeA.Set3.prt	683415	693013	NZ_CP027331.1	2013C-3277	
ESCO001.0321.00388.C001.PICI.TypeA.Set4.prt	2006429	2014687	NZ_CP027331.1	2013C-3277	
ESCO001.0321.00389.C001.PICI.TypeA.Set1.prt	2431021	2441491	NZ_CP027342.1	2014C-4587	
ESCO001.0321.00389.C001.PICI.TypeA.Set2.prt	3204169	3213725	NZ_CP027342.1	2014C-4587	
ESCO001.0321.00391.C001.PICI.TypeA.Set2.prt	1816730	1824919	NZ_CP027352.1	2012C-4606	
ESCO001.0321.00391.C001.PICI.TypeA.Set3.prt	571704	581482	NZ_CP027352.1	2012C-4606	
ESCO001.0321.00393.C001.PICI.TypeB.variant0003.Set1.prt	53733	63877	NZ_CP027373.1		29-May
ESCO001.0321.00394.C001.PICI.TypeA.Set2.prt	1568270	1577206	NZ_CP027380.1	2013C-3250	
ESCO001.0321.00395.C001.PICI.TypeA.Set1.prt	3808293	3816483	NZ_CP027387.1	2014C-3057	
ESCO001.0321.00396.C001.PICI.TypeA.Set3.prt	708051	717820	NZ_CP027388.1	2011C-4251	
ESCO001.0321.00398.C001.PICI.TypeA.Set2.prt	625521	635420	NZ_CP027362.1	95-3192	
ESCO001.0321.00405.C001.PICI.TypeA.Set2.prt	712020	721799	NZ_CP027544.1	2013C-3264	
ESCO001.0321.00405.C001.PICI.TypeB.variant0001.Set1.prt	4154585	4166446	NZ_CP027544.1	2013C-3264	
ESCO001.0321.00406.C001.PICI.TypeA.Set2.prt	2025914	2034103	NZ_CP027546.1	2013C-4187	

ESCO001.0321.00406.C001.PICI.TypeA.Set4.prt	603395	613118	NZ_CP027546.1	2013C-4187
ESCO001.0321.00410.C001.PICI.TypeB.variant0003.Set1.prt	1140467	1150339	NZ_CP028192.1	CFSAN018748
ESCO001.0321.00431.C001.PICI.TypeA.Set1.prt	1366046	1376356	NZ_CP028381.1	RM10466
ESCO001.0321.00436.C001.PICI.TypeA.Set1.prt	2777397	2786333	NZ_CP029692.1	SD134209
ESCO001.0321.00437.C001.PICI.TypeB.variant0001.Set1.prt	3788197	3796896	NZ_CP029741.1	AR_0085
ESCO001.0321.00443.C001.PICI.TypeA.Set1.prt	2701367	2710302	NZ_CP028432.1	RM9975
ESCO001.0321.00448.C001.PICI.TypeA.SetR1.prt	1157352	1167251	NZ_CP030767.1	2017C-4109
ESCO001.0321.00450.C001.PICI.TypeA.Set2.prt	2241437	2251217	NZ_CP030939.1	AMSHJX01
ESCO001.0321.00452.C001.PICI.TypeA.Set2.prt	77579	86667	CP031134.1	CFSAN064035
ESCO001.0321.00456.C001.PICI.TypeA.Set1.prt	2186483	2196634	NZ_CP031256.1	M16807
ESCO001.0321.00460.C001.PICI.TypeA.Set2.prt	3310482	3319773	NZ_CP031546.1	cq9
ESCO001.0321.00482.C001.PICI.TypeB.variant0001.Set1.prt	2383850	2391545	NZ_CP033635.1	unknown
ESCO001.0321.00482.C001.PICI.TypeB.variant0002.Set2.prt	1593993	1602925	NZ_CP033635.1	unknown
ESCO001.0321.00501.C001.PICI.TypeB.variant0001.Set2.prt	1897996	1906369	NZ_CP025903.1	300709
ESCO001.0321.00506.C001.PICI.TypeA.Set1.prt	1107815	1116748	NZ_CP025859.1	504239
ESCO001.0321.00512.C001.PICI.TypeA.Set1.prt	2736922	2745856	NZ_AP018802.1	E2863
ESCO001.0321.00513.C001.PICI.TypeA.Set1.prt	1618943	1628722	NZ_AP018808.1	E2865
ESCO001.0321.00513.C001.PICI.TypeA.Set2.prt	2985957	2994147	NZ_AP018808.1	E2865
ESCO001.0321.00520.C001.PICI.TypeA.Set1.prt	2243017	2252916	NZ_CP034792.1	2009C-3378
ESCO001.0321.00522.C001.PICI.TypeA.Set1.prt	1335363	1345272	NZ_CP034794.1	Jun-62
ESCO001.0321.00523.C001.PICI.TypeA.Set1.prt	5279619	5289518	NZ_CP034799.1	2009C-4687
ESCO001.0321.00525.C001.PICI.TypeA.Set1.prt	5272853	5282752	NZ_CP034801.1	2010C-3142
ESCO001.0321.00526.C001.PICI.TypeA.Set1.prt	5315595	5325498	NZ_CP034808.1	14-Aug
ESCO001.0321.00531.C001.PICI.TypeA.Set1.prt	2535465	2545616	NZ_CP035486.1	NissleGFP_pZE21
ESCO001.0321.00542.C001.PICI.TypeA.Set1.prt	491173	500952	NZ_CP037945.1	CFSAN027346
ESCO001.0321.00542.C001.PICI.TypeA.Set3.prt	4753878	4762068	NZ_CP037945.1	CFSAN027346
ESCO001.0321.00543.C001.PICI.TypeA.Set1.prt	4137884	4146144	NZ_CP037941.1	CFSAN027350
ESCO001.0321.00543.C001.PICI.TypeA.Set3.prt	2805596	2815194	NZ_CP037941.1	CFSAN027350
ESCO001.0321.00544.C001.PICI.TypeA.Set2.prt	1443586	1451776	NZ_CP037943.1	CFSAN027343
ESCO001.0321.00553.C001.PICI.TypeA.Set1.prt	3467041	3476103	NZ_CP038505.1	28Eco12
ESCO001.0321.00563.C001.PICI.TypeA.SetR1.prt	3249497	3259384	NZ_CP028592.1	150
ESCO001.0321.00564.C001.PICI.TypeA.Set1.prt	3126057	3135955	NZ_CP028596.1	149
ESCO001.0321.00565.C001.PICI.TypeA.Set1.prt	3125997	3135895	NZ_CP028598.1	148
ESCO001.0321.00566.C001.PICI.TypeA.Set1.prt	3126057	3135955	NZ_CP028596.1	149
ESCO001.0321.00567.C001.PICI.TypeA.Set2.prt	3191808	3201695	NZ_CP028603.1	144
ESCO001.0321.00568.C001.PICI.TypeA.Set1.prt	3103472	3113359	NZ_CP028614.1	141

ESCO001.0321.00569.C001.PICI.TypeA.Set2.prt	3103835	3113722	NZ_CP028626.1		137
ESCO001.0321.00570.C001.PICI.TypeA.Set2.prt	3103837	3113724	NZ_CP028623.1		138
ESCO001.0321.00571.C001.PICI.TypeA.Set1.prt	3101075	3110962	NZ_CP028632.1		135
ESCO001.0321.00572.C001.PICI.TypeA.Set2.prt	3101070	3110957	NZ_CP028635.1		134
ESCO001.0321.00573.C001.PICI.TypeA.Set1.prt	3101068	3110955	NZ_CP028620.1		139
ESCO001.0321.00574.C001.PICI.TypeA.Set2.prt	3101070	3110957	NZ_CP028635.1		134
ESCO001.0321.00575.C001.PICI.TypeA.Set2.prt	3101171	3111058	NZ_CP028647.1		130
ESCO001.0321.00578.C001.PICI.TypeA.Set1.prt	3103837	3113724	NZ_CP028623.1		138
ESCO001.0321.00579.C001.PICI.TypeA.Set2.prt	3103838	3113725	NZ_CP028668.1		117
ESCO001.0321.00580.C001.PICI.TypeA.Set1.prt	3043668	3053555	NZ_CP028674.1		115
ESCO001.0321.00581.C001.PICI.TypeA.Set2.prt	3101076	3110963	NZ_CP028671.1		116
ESCO001.0321.00584.C001.PICI.TypeA.Set2.prt	3103844	3113731	NZ_CP028617.1		140
ESCO001.0321.00585.C001.PICI.TypeA.Set2.prt	3101068	3110955	NZ_CP028620.1		139
ESCO001.0321.00586.C001.PICI.TypeA.Set2.prt	3103837	3113724	NZ_CP028623.1		138
ESCO001.0321.00587.C001.PICI.TypeA.Set1.prt	3101169	3111056	NZ_CP028644.1		131
ESCO001.0321.00589.C001.PICI.TypeA.Set2.prt	3103837	3113724	NZ_CP028623.1		138
ESCO001.0321.00590.C001.PICI.TypeA.Set2.prt	3101062	3110949	NZ_CP028662.1		119
ESCO001.0321.00591.C001.PICI.TypeA.Set2.prt	3101062	3110949	NZ_CP028662.1		119
ESCO001.0321.00592.C001.PICI.TypeA.Set2.prt	3101069	3110956	NZ_CP028677.1		114
ESCO001.0321.00598.C001.PICI.TypeA.Set1.prt	3100995	3110882	NZ_CP028680.1		113
ESCO001.0321.00604.C001.PICI.TypeA.Set1.prt	2514480	2523542	NZ_CP040390.1	A1_136	
ESCO001.0321.00611.C001.PICI.TypeA.Set1.prt	1618005	1628156	NZ_CP040919.1	FC853_EC	
ESCO001.0321.00615.C001.PICI.TypeB.variant0002.Set1.prt	3120160	3129763	NZ_CP041304.1	MSHS 133	
ESCO001.0321.00616.C001.PICI.TypeA.Set1.prt	1154548	1164447	NZ_CP015853.1	ATCC 43889	
ESCO001.0321.00639.C001.PICI.TypeB.variant0002.Set4.prt	2317013	2326748	NZ_CP044311.1	RM13752	
ESCO001.0321.00641.C001.PICI.TypeB.variant0002.Set1.prt	1485368	1495103	NZ_CP044313.1	RM11911	
ESCO001.0321.00642.C001.PICI.TypeA.Set2.prt	4599760	4609787	NZ_CP044314.1	RM9245	
ESCO001.0321.00643.C001.PICI.TypeA.Set1.prt	2209281	2219050	NZ_CP044315.1	SJ7	
ESCO001.0321.00647.C001.PICI.TypeA.Set1.prt	956893	965955	NZ_CP032145.1	15.TR.026_OXA	
ESCO001.0321.00650.C001.PICI.TypeA.Set1.prt	3148546	3158433	NZ_CP032789.1	NZRM4169	
ESCO001.0321.00651.C001.PICI.TypeA.Set1.prt	2876854	2886753	NZ_CP032791.1	NZRM4165	
ESCO001.0321.00652.C001.PICI.TypeA.Set2.prt	3043609	3053505	NZ_CP032793.1	NZRM3614	
ESCO001.0321.00653.C001.PICI.TypeA.Set2.prt	3730204	3738621	NZ_CP032795.1	ERL06-2503	
ESCO001.0321.00653.C001.PICI.TypeA.Set3.prt	3190459	3200358	NZ_CP032795.1	ERL06-2503	
ESCO001.0321.00655.C001.PICI.TypeA.Set1.prt	3164092	3173991	NZ_CP032801.1	ERL06-2442	
ESCO001.0321.00655.C001.PICI.TypeA.Set2.prt	3700108	3708070	NZ_CP032801.1	ERL06-2442	

ESCO001.0321.00656.C001.PICI.TypeA.Set1.prt	3250776	3260675	NZ_CP032803.1	ERL05-1306
ESCO001.0321.00659.C001.PICI.TypeA.Set2.prt	3171014	3180913	NZ_CP032811.1	ERL03-1416
ESCO001.0321.00659.C001.PICI.TypeA.Set3.prt	3610554	3619025	NZ_CP032811.1	ERL03-1416
ESCO001.0321.00668.C001.PICI.TypeA.Set1.prt	3057404	3067291	NZ_CP040305.1	HB6
ESCO001.0321.00669.C001.PICI.TypeA.Set2.prt	1831406	1839876	NZ_CP040307.1	F1 E4
ESCO001.0321.00669.C001.PICI.TypeA.Set3.prt	2310862	2320749	NZ_CP040307.1	F1 E4
ESCO001.0321.00670.C001.PICI.TypeA.Set1.prt	2301352	2311239	NZ_CP040309.1	21B8
ESCO001.0321.00671.C001.PICI.TypeA.Set1.prt	2302854	2312753	NZ_CP040311.1	F3398
ESCO001.0321.00672.C001.PICI.TypeA.Set2.prt	2340754	2350641	NZ_CP040313.1	M7638
ESCO001.0321.00673.C001.PICI.TypeA.Set2.prt	1834926	1843387	NZ_CP040314.1	MA11
ESCO001.0321.00673.C001.PICI.TypeA.Set3.prt	2309767	2319663	NZ_CP040314.1	MA11
ESCO001.0321.00674.C001.PICI.TypeA.Set1.prt	2339508	2349395	NZ_CP040316.1	CV261
ESCO001.0321.00674.C001.PICI.TypeA.Set3.prt	1868262	1876721	NZ_CP040316.1	CV261
ESCO001.0321.00677.C001.PICI.TypeA.Set2.prt	2274029	2283928	NZ_CP045827.1	AUSMDU00014361
ESCO001.0321.00681.C001.PICI.TypeA.Set1.prt	1537298	1547197	NZ_CP035545.1	FSIS11705876
ESCO001.0321.00682.C001.PICI.TypeA.Set1.prt	3206896	3216793	NZ_CP045975.1	AUSMDU00002545
ESCO001.0321.00686.C001.PICI.TypeA.Set1.prt	2978139	2987196	NZ_CP046009.1	1919D62
ESCO001.0321.00690.C001.PICI.TypeA.Set1.prt	4152949	4162848	NZ_CP046527.1	RM19259
ESCO001.0321.00697.C001.PICI.TypeA.Set1.prt	3133445	3142502	unknown	unknown
ESCO001.0321.00713.C001.PICI.TypeA.Set1.prt	3339634	3349205	NZ_CP041431.1	STEC316
ESCO001.0321.00714.C001.PICI.TypeA.Set1.prt	3613867	3623438	NZ_CP041433.1	STEC313
ESCO001.0321.00719.C001.PICI.TypeA.Set1.prt	2105876	2116024	NZ_CP048304.1	9
ESCO001.0321.00722.C001.PICI.TypeB.variant0001.Set1.prt	2949699	2958233	NZ_CP048337.1	142
ESCO001.0321.00737.C001.PICI.TypeA.Set1.prt	4619054	4628116	NZ_CP048934.1	190693
ESCO001.0321.00768.C001.PICI.TypeA.Set1.prt	1967801	1977033	NZ_CP050862.1	8-3-DC15
ESCO001.0321.00768.C001.PICI.TypeA.Set2.prt	1043027	1051955	NZ_CP050862.1	8-3-DC15
ESCO001.0321.00769.C001.PICI.TypeA.Set1.prt	215423	224655	NZ_CP050865.1	8-3-Ti3
ESCO001.0321.00769.C001.PICI.TypeA.Set2.prt	4210304	4219232	NZ_CP050865.1	8-3-Ti3
ESCO001.0321.00778.C001.PICI.TypeA.Set1.prt	3926426	3936105	NZ_CP051725.1	SCU-112
ESCO001.0321.00781.C001.PICI.TypeA.Set1.prt	3116700	3125929	NZ_CP051714.1	SCU-122
ESCO001.0321.00791.C001.PICI.TypeA.Set1.prt	1725718	1735196	NZ_CP052877.1	C21
ESCO001.0321.00793.C001.PICI.TypeA.Set1.prt	280144	290295	NZ_CP037449.1	ATCC 25922
ESCO001.0321.00797.C001.PICI.TypeA.Set1.prt	705185	715287	NZ_CP053234.1	SCU-106
ESCO001.0321.00826.C001.PICI.TypeB.variant0003.Set1.prt	993836	1003727	NZ_CP054363.1	SCU-171
ESCO001.0321.00832.C001.PICI.TypeA.Set2.prt	3074714	3083770	NZ_AP023190.1	TUM18530
ESCO001.0321.00833.C001.PICI.TypeA.Set2.prt	3074735	3083791	NZ_AP023197.1	TUM18780

ESCO001.0321.00837.C001.PICI.TypeB.variant0003.Set1.prt	1609281	1618662	NZ_CP050498.1	RM13322
ESCO001.0321.00847.C001.PICI.TypeB.variant0001.Set1.prt	2724678	2733508	NZ_CP054236.1	EcPF5
ESCO001.0321.00878.C001.PICI.TypeA.Set2.prt	2261299	2271078	NZ_CP058682.2	MBT-5
ESCO001.0321.00878.C001.PICI.TypeB.variant0004.Set1.prt	1777560	1785748	NZ_CP058682.2	MBT-5
ESCO001.0321.00917.C001.PICI.TypeB.variant0001.Set1.prt	2792415	2801115	NZ_AP022261.1	WP8-S18-ESBL-07
ESCO001.0321.00918.C001.PICI.TypeA.Set1.prt	3345395	3354457	NZ_AP022287.1	WP9-S17-ESBL-11
ESCO001.0321.00920.C001.PICI.TypeA.Set1.prt	1828182	1838333	NZ_AP022298.1	BEC1-S17-ESBL-09
ESCO001.0321.00938.C001.PICI.TypeA.Set1.prt	930442	940121	NZ_CP061206.1	DA61218
ESCO001.0321.00940.C001.PICI.TypeA.Set3.prt	2218754	2228653	NZ_CP024659.1	RM9872-C1
ESCO001.0321.00942.C001.PICI.TypeA.Set2.prt	2958159	2967458	NZ_CP061337.1	M11957
ESCO001.0321.00943.C001.PICI.TypeA.Set2.prt	2958398	2967697	NZ_CP061339.1	M00057
ESCO001.0321.00946.C001.PICI.TypeB.variant0001.Set1.prt	2431646	2440531	NZ_CP061914.1	SMBL
ESCO001.0321.00949.C001.PICI.TypeB.variant0001.Set2.prt	1703771	1712200	NZ_CP062204.1	C311
ESCO001.0321.00965.C001.PICI.TypeB.variant0001.Set1.prt	2976340	2984417	NZ_CP063046.1	KC-Dl-1
ESCO001.0321.00966.C001.PICI.TypeA.Set2.prt	2920123	2929422	NZ_CP063153.1	M7424
ESCO001.0321.00972.C001.PICI.TypeA.Set1.prt	1711294	1721445	NZ_AP022533.1	THO-006
ESCO001.0321.00976.C001.PICI.TypeA.Set1.prt	1711179	1721329	NZ_CP063983.1	25DN
ESCO001.0321.00978.C001.PICI.TypeB.variant0001.Set1.prt	1497950	1506457	NZ_AP022811.1	JE86-ST02
ESCO001.0321.00979.C001.PICI.TypeB.variant0001.Set1.prt	1494948	1503454	NZ_AP022815.1	JE86-ST05
ESCO001.0321.00994.C001.PICI.TypeA.Set1.prt	489428	497330	NZ_CP065607.1	FDAARGOS_946
ESCO001.0321.00994.C001.PICI.TypeA.Set2.prt	4448166	4458066	NZ_CP065607.1	FDAARGOS_946
ESCO001.0321.01001.C001.PICI.TypeA.Set1.prt	1711195	1721346	NZ_CP066144.1	ZLWT
ESCO001.0321.01002.C001.PICI.TypeB.variant0003.Set1.prt	687999	697631	NZ_CP061101.1	1EC213
ESCO001.0321.01006.C001.PICI.TypeA.Set1.prt	1881333	1890246	NZ_CP066806.1	TCM3e1
ESCO001.0321.01007.C001.PICI.TypeA.Set1.prt	3051426	3060720	NZ_AP024205.1	TA8571
ESCO001.0321.01009.C001.PICI.TypeA.Set1.prt	1295180	1304593	CP068035.1	U90
ESCO001.0321.01009.C001.PICI.TypeA.Set6.prt	3372779	3382079	NZ_CP068035.1	U90
ESCO001.0321.01009.C001.PICI.TypeB.variant0001.Set2.prt	1096831	1104442	NZ_CP068035.1	U90
ESCO001.0321.01019.C001.PICI.TypeA.Set1.prt	3328208	3337176	NZ_CP068829.1	RIVM_C030453
ESCO001.0321.01027.C001.PICI.TypeA.Set1.prt	3340688	3349745	NZ_CP068804.1	RIVM_C018563
ESCO001.0321.01028.C001.PICI.TypeA.Set1.prt	3391358	3400420	NZ_CP068800.1	RIVM_C017997
ESCO001.0321.01037.C001.PICI.TypeA.Set1.prt	3422105	3431167	NZ_CP068810.1	RIVM_C028536
ESCO001.0321.01042.C001.PICI.TypeA.Set1.prt	3060366	3069418	NZ_CP068803.1	RIVM_C018404
ESCO001.0321.01047.C001.PICI.TypeA.Set1.prt	3386495	3395462	NZ_CP068796.1	RIVM_C012087
ESCO001.0321.01060.C001.PICI.TypeA.Set1.prt	2021622	2031773	NZ_CP069447.1	FDAARGOS_1255
ESCO001.0321.01066.C001.PICI.TypeA.Set1.prt	2120591	2129827	NZ_CP069500.1	FDAARGOS_1258

ESCO001.0321.01066.C001.PICI.TypeA.Set2.prt	4701619	4710544	NZ_CP069500.1	FDAARGOS_1258	
ESCO001.0321.01073.C001.PICI.TypeA.Set1.prt	3022722	3031958	NZ_CP069517.1	FDAARGOS_1262	
ESCO001.0321.01074.C001.PICI.TypeA.Set1.prt	3235920	3244980	NZ_CP069522.1	FDAARGOS_1267	
ESCO001.0321.01096.C001.PICI.TypeA.Set1.prt	4898476	4908628	NZ_CP070041.1	FDAARGOS_1293	
ESCO001.0321.01097.C001.PICI.TypeA.Set1.prt	841283	850513	NZ_CP070045.1	FDAARGOS_1303	
ESCO001.0321.01139.C001.PICI.TypeB.variant0001.Set1.prt	1810536	1819043	NZ_LT601384.1	unknown	
ESCO001.0321.01169.C001.PICI.TypeA.Set1.prt	3136814	3146358	NZ_LR134079.1	NCTC9112	
ESCO001.0321.01169.C001.PICI.TypeA.Set2.prt	1794434	1803893	NZ_LR134079.1	NCTC9112	
ESCO001.0321.01172.C001.PICI.TypeA.Set1.prt	1752224	1761309	NZ_LR134092.1	NCTC10444	
ESCO001.0321.01193.C001.PICI.TypeB.variant0001.Set1.prt	2424892	2433399	NZ_LR217818.1	NCTC86	
ESCO001.0321.01219.C001.PICI.TypeA.Set1.prt	2705718	2714923	NZ_LR882978.1	unknown	
ESCO001.0321.01268.C001.PICI.TypeA.Set1.prt	2376809	2386708	NZ_CP014314.1	JEONG-1266	
ESCO001.0321.01269.C001.PICI.TypeA.Set3.prt	1629348	1639235	NZ_CP015846.1	FRIK2069	
ESCO001.0321.01269.C001.PICI.TypeA.Set4.prt	1077175	1085583	NZ_CP015846.1	FRIK2069	
ESCO001.0321.01270.C001.PICI.TypeA.Set1.prt	1077179	1085587	NZ_CP015842.1	FRIK2533	
ESCO001.0321.01270.C001.PICI.TypeA.Set3.prt	1629337	1639224	NZ_CP015842.1	FRIK2533	
ESCO001.0321.01271.C001.PICI.TypeA.Set2.prt	1629355	1639242	NZ_CP015843.2	FRIK2455	
ESCO001.0321.01271.C001.PICI.TypeA.Set3.prt	1077187	1085594	NZ_CP015843.2	FRIK2455	
ESCO001.0321.01272.C001.PICI.TypeA.Set2.prt	1694687	1704574	NZ_CP016625.1	FRIK944	
ESCO001.0321.01273.C001.PICI.TypeA.Set1.prt	3118182	3128081	NZ_CP017434.1		1130
ESCO001.0321.01274.C001.PICI.TypeA.Set1.prt	3116886	3126785	NZ_CP017436.1		2149
ESCO001.0321.01276.C001.PICI.TypeA.Set1.prt	3118406	3128305	NZ_CP017438.1		2159
ESCO001.0321.01277.C001.PICI.TypeA.Set1.prt	3117074	3126973	NZ_CP017446.1		9234
ESCO001.0321.01279.C001.PICI.TypeA.Set1.prt	3114550	3124449	NZ_CP017442.1		4276
ESCO001.0321.01280.C001.PICI.TypeA.Set1.prt	1536529	1546428	NZ_CP033605.1	TR01	
ESCO001.0321.01281.C001.PICI.TypeA.Set1.prt	1031452	1039286	NZ_AP018488.1	PV15-279	
ESCO001.0321.01281.C001.PICI.TypeA.SetR1.prt	1479987	1489886	NZ_AP018488.1	PV15-279	
ESCO001.0321.01282.C001.PICI.TypeA.Set3.prt	1553563	1563450	NZ_CP035366.1	C1-057	
ESCO001.0321.01285.C001.PICI.TypeA.Set2.prt	2442111	2452010	NZ_CP040572.1	ECP17-46	
ESCO001.0321.01290.C001.PICI.TypeA.Set1.prt	3191182	3201091	NZ_CP038282.1	F8492	
ESCO001.0321.01291.C001.PICI.TypeA.Set1.prt	3196743	3206630	NZ_CP038284.1	YB14-1	
ESCO001.0321.01292.C001.PICI.TypeA.Set2.prt	3146939	3156838	NZ_CP038287.1	TX 376-2	
ESCO001.0321.01292.C001.PICI.TypeA.Set3.prt	3626502	3634971	NZ_CP038287.1	TX 376-2	
ESCO001.0321.01293.C001.PICI.TypeA.Set1.prt	2461212	2471099	NZ_CP038290.1	TX 265-1	
ESCO001.0321.01294.C001.PICI.TypeA.Set2.prt	2641602	2651489	NZ_CP038292.1	TB21-1	
ESCO001.0321.01295.C001.PICI.TypeA.Set1.prt	3022303	3032203	NZ_CP038295.1	TB182A	

ESCO001.0321.01296.C001.PICI.TypeA.Set1.prt	3207380	3217276	NZ_CP038300.1	SS TX 754-1	
ESCO001.0321.01297.C001.PICI.TypeA.Set1.prt	3246279	3256178	NZ_CP038302.1	SS TX 313-1	
ESCO001.0321.01297.C001.PICI.TypeA.Set4.prt	3802078	3810095	NZ_CP038302.1	SS TX 313-1	
ESCO001.0321.01298.C001.PICI.TypeA.Set1.prt	3181706	3191593	NZ_CP038305.1	SS NE 1040-1	
ESCO001.0321.01299.C001.PICI.TypeA.Set1.prt	3244710	3254597	NZ_CP038309.1	Show KS 470-1	
ESCO001.0321.01301.C001.PICI.TypeA.Set1.prt	3111173	3121042	NZ_CP038316.1	NE92	
ESCO001.0321.01302.C001.PICI.TypeA.Set1.prt	3114673	3124572	NZ_CP038319.1	NE122	
ESCO001.0321.01305.C001.PICI.TypeA.Set1.prt	1781181	1789633	NZ_CP038328.1	NE 1092-2	
ESCO001.0321.01305.C001.PICI.TypeA.Set2.prt	3246638	3256537	NZ_CP038328.1	NE 1092-2	
ESCO001.0321.01306.C001.PICI.TypeA.Set2.prt	2283795	2293682	NZ_CP038333.1	N8B7-2	
ESCO001.0321.01307.C001.PICI.TypeA.Set2.prt	3204831	3214730	NZ_CP038336.1	LSU61	
ESCO001.0321.01308.C001.PICI.TypeA.Set2.prt	3181022	3190921	NZ_CP038339.1	H6437	
ESCO001.0321.01310.C001.PICI.TypeA.Set1.prt	2774527	2784426	NZ_CP038344.1	Gim1-1	
ESCO001.0321.01311.C001.PICI.TypeA.Set1.prt	3241540	3251439	NZ_CP038346.1	G5295	
ESCO001.0321.01314.C001.PICI.TypeA.Set1.prt	3184696	3194605	NZ_CP038353.1	F8797	
ESCO001.0321.01316.C001.PICI.TypeA.Set1.prt	3106356	3116254	NZ_CP038360.1	F7386	
ESCO001.0321.01318.C001.PICI.TypeA.Set1.prt	3212208	3222107	NZ_CP038366.1	F6667	
ESCO001.0321.01319.C001.PICI.TypeA.Set1.prt	3732279	3740771	NZ_CP038369.1	F6321	
ESCO001.0321.01322.C001.PICI.TypeA.Set2.prt	2328902	2338789	NZ_CP038376.1	F1273	
ESCO001.0321.01323.C001.PICI.TypeA.Set2.prt	3173212	3183111	NZ_CP038380.1	E32511	
ESCO001.0321.01324.C001.PICI.TypeA.Set1.prt	2292162	2302062	NZ_CP038383.1	DEC5E	
ESCO001.0321.01326.C001.PICI.TypeA.Set2.prt	3112686	3122585	NZ_CP038389.1	DEC5B	
ESCO001.0321.01327.C001.PICI.TypeA.Set1.prt	1782405	1790307	NZ_CP038394.1	DEC5A	
ESCO001.0321.01327.C001.PICI.TypeA.Set2.prt	3134308	3144208	NZ_CP038394.1	DEC5A	
ESCO001.0321.01328.C001.PICI.TypeB.variant0001.Set1.prt	3078460	3086847	NZ_CP038398.1	DEC4E	
ESCO001.0321.01329.C001.PICI.TypeA.Set2.prt	3229800	3239687	NZ_CP038402.1	BB24-1	
ESCO001.0321.01332.C001.PICI.TypeA.Set2.prt	2367704	2377603	NZ_CP038412.1	493/89	
ESCO001.0321.01333.C001.PICI.TypeA.Set1.prt	2958359	2968258	NZ_CP038414.1	17B6-2	
ESCO001.0321.01334.C001.PICI.TypeA.Set2.prt	3178457	3188356	NZ_CP038416.1		3/5/2001
ESCO001.0321.01335.C001.PICI.TypeA.Set2.prt	3132497	3142384	NZ_CP038419.1		2/6/2002
ESCO001.0321.01336.C001.PICI.TypeA.Set1.prt	3151679	3161577	NZ_CP038421.1		7636
ESCO001.0321.01337.C001.PICI.TypeA.Set1.prt	3184605	3194492	NZ_CP038423.1		7409
ESCO001.0321.01340.C001.PICI.TypeA.Set1.prt	3689254	3697714	NZ_CP039834.1	MB41-1	
ESCO001.0321.01340.C001.PICI.TypeA.Set2.prt	3209858	3219757	NZ_CP039834.1	MB41-1	
ESCO001.0321.01341.C001.PICI.TypeA.Set1.prt	3127395	3137295	NZ_CP039837.1	USDA5905	
ESCO001.0321.01342.C001.PICI.TypeA.Set1.prt	3206629	3216516	NZ_CP040107.1	MB9-1	

ESCO001.0321.01346.C001.PICI.TypeA.Set1.prt	3191680	3201579	NZ_CP066753.1	ECP19-598
ESCO001.0321.01347.C001.PICI.TypeA.Set1.prt	3124014	3133901	NZ_CP066748.1	ECP19-2498
ESCO001.0321.01348.C001.PICI.TypeA.Set2.prt	3163318	3182074	NZ_CP062778.1	Z1836
ESCO001.0321.01349.C001.PICI.TypeA.Set1.prt	3115777	3134533	NZ_CP062700.1	Z1835
ESCO001.0321.01350.C001.PICI.TypeA.Set1.prt	3155615	3165514	NZ_CP062702.1	Z1834
ESCO001.0321.01351.C001.PICI.TypeA.Set3.prt	3221327	3231226	NZ_CP062705.1	Z1833
ESCO001.0321.01352.C001.PICI.TypeA.Set2.prt	3221322	3231221	NZ_CP062708.1	Z1832
ESCO001.0321.01353.C001.PICI.TypeA.Set1.prt	3221334	3231233	NZ_CP062711.1	Z1831
ESCO001.0321.01354.C001.PICI.TypeA.Set1.prt	3220009	3229908	NZ_CP062713.1	Z1830
ESCO001.0321.01355.C001.PICI.TypeA.Set1.prt	3236460	3246359	NZ_CP062715.1	Z1826
ESCO001.0321.01356.C001.PICI.TypeA.Set2.prt	3258883	3268782	NZ_CP062717.1	Z1825
ESCO001.0321.01357.C001.PICI.TypeA.Set2.prt	3020021	3029920	NZ_CP062719.1	Z1816
ESCO001.0321.01358.C001.PICI.TypeA.Set2.prt	3021407	3031306	NZ_CP062721.1	Z1815
ESCO001.0321.01359.C001.PICI.TypeA.Set1.prt	3222183	3232081	NZ_CP062723.1	Z1814
ESCO001.0321.01359.C001.PICI.TypeA.Set2.prt	3698972	3707431	NZ_CP062723.1	Z1814
ESCO001.0321.01360.C001.PICI.TypeA.Set1.prt	3305779	3315678	NZ_CP062725.1	Z1813
ESCO001.0321.01361.C001.PICI.TypeA.Set2.prt	3215654	3225553	NZ_CP062727.1	Z1812
ESCO001.0321.01362.C001.PICI.TypeA.Set2.prt	3691924	3700391	NZ_CP062729.1	Z1811
ESCO001.0321.01362.C001.PICI.TypeA.Set3.prt	3215905	3225804	NZ_CP062729.1	Z1811
ESCO001.0321.01363.C001.PICI.TypeA.Set1.prt	3694429	3702837	NZ_CP062780.1	Z1769
ESCO001.0321.01363.C001.PICI.TypeA.Set3.prt	3217216	3227115	NZ_CP062780.1	Z1769
ESCO001.0321.01364.C001.PICI.TypeA.Set1.prt	3694433	3702841	NZ_CP062731.1	Z1768
ESCO001.0321.01364.C001.PICI.TypeA.Set3.prt	3217219	3227118	NZ_CP062731.1	Z1768
ESCO001.0321.01365.C001.PICI.TypeA.Set1.prt	2092216	2102115	NZ_CP062733.1	Z1767
ESCO001.0321.01365.C001.PICI.TypeA.Set2.prt	3694406	3702813	NZ_CP062733.1	Z1767
ESCO001.0321.01366.C001.PICI.TypeA.Set1.prt	2092230	2102129	CP062739.1	Z1723
ESCO001.0321.01366.C001.PICI.TypeA.Set3.prt	3907654	3916062	NZ_CP062739.1	Z1723
ESCO001.0321.01366.C001.PICI.TypeA.Set4.prt	3430442	3440341	NZ_CP062739.1	Z1723
ESCO001.0321.01367.C001.PICI.TypeA.Set1.prt	3694434	3702842	NZ_CP062736.1	Z1766
ESCO001.0321.01367.C001.PICI.TypeA.Set3.prt	3217220	3227119	NZ_CP062736.1	Z1766
ESCO001.0321.01368.C001.PICI.TypeA.Set1.prt	3218552	3228451	NZ_CP062742.1	Z1626
ESCO001.0321.01369.C001.PICI.TypeA.Set2.prt	2485578	2495477	NZ_CP062744.1	Z1615
ESCO001.0321.01369.C001.PICI.TypeA.Set3.prt	3696867	3705274	NZ_CP062744.1	Z1615
ESCO001.0321.01370.C001.PICI.TypeA.Set1.prt	2481706	2491605	NZ_CP062746.1	Z1504
ESCO001.0321.01371.C001.PICI.TypeA.Set1.prt	3217202	3227101	NZ_CP062749.1	Z1486
ESCO001.0321.01372.C001.PICI.TypeA.Set1.prt	3219881	3229780	NZ_CP062752.1	Z910

ESCO001.0321.01373.C001.PICI.TypeA.Set1.prt	2671889	2681788	NZ_CP062755.1	Z903	
ESCO001.0321.01374.C001.PICI.TypeA.Set2.prt	2619106	2629005	NZ_CP062761.1	Z887	
ESCO001.0321.01375.C001.PICI.TypeA.Set1.prt	3206369	3216268	NZ_CP062758.1	Z892	
ESCO001.0321.01376.C001.PICI.TypeA.Set2.prt	2091296	2101195	NZ_CP062763.1	Z885	
ESCO001.0321.01377.C001.PICI.TypeA.Set1.prt	3261436	3271335	NZ_CP062766.1	Z866	
ESCO001.0321.01378.C001.PICI.TypeA.Set1.prt	3300890	3310789	NZ_CP062782.1	Z869	
ESCO001.0321.01379.C001.PICI.TypeA.Set1.prt	3161217	3171116	NZ_CP062769.1	Z852	
ESCO001.0321.01380.C001.PICI.TypeA.Set2.prt	3255331	3265230	NZ_CP062771.1	Z570	
ESCO001.0321.01381.C001.PICI.TypeA.Set2.prt	2094731	2104630	NZ_CP062774.1	Z563	
ESCO001.0321.01385.C001.PICI.TypeA.Set1.prt	2740694	2749628	NZ_AP019761.1		110512
ESCO001.0321.01388.C001.PICI.TypeB.variant0001.Set2.prt	1834918	1843370	NC_017626.1		42
ESCO001.0321.01390.C001.PICI.TypeA.Set3.prt	3604640	3614419	NZ_CP031922.1	FWSEC0001	
ESCO001.0321.01390.C001.PICI.TypeB.variant0004.Set2.prt	1781417	1789605	NZ_CP031922.1	FWSEC0001	
ESCO001.0321.01394.C001.PICI.TypeA.Set1.prt	1311062	1328858	CP007799.1	Nissle 1917	
ESCO001.0321.01395.C001.PICI.TypeA.Set1.prt	937780	947931	NZ_CP022686.1	Nissle 1917	
ESCO001.0321.01398.C001.PICI.TypeA.Set1.prt	1893233	1902835	NZ_CP035751.1	E110019	
ESCO001.0321.01403.C001.PICI.TypeA.Set1.prt	2485199	2494134	NZ_CP031912.1	FWSEC0005	
ESCO001.0321.01404.C001.PICI.TypeA.Set1.prt	3548022	3556491	NZ_CP031908.1	FWSEC0007	
ESCO001.0321.01411.C001.PICI.TypeA.Set1.prt	1535679	1545578	NC_011353.1	EC4115	
ESCO001.0321.01432.C001.PICI.TypeA.Set1.prt	1535967	1545866	NC_013008.1	TW14359	
ESCO001.0321.01436.C001.PICI.TypeA.Set2.prt	3041748	3049938	NC_013361.1		11368
ESCO001.0321.01436.C001.PICI.TypeA.Set4.prt	1619628	1629414	NC_013361.1		11368
ESCO001.0321.01443.C001.PICI.TypeA.Set2.prt	1326056	1335825	NC_013353.1		12009
ESCO001.0321.01444.C001.PICI.TypeA.Set1.prt	2855322	2864257	NC_013364.1		11128
ESCO001.0321.01456.C001.PICI.TypeA.Set1.prt	1444753	1454654	NC_013941.1	CB9615	
ESCO001.0321.01457.C001.PICI.TypeA.Set1.prt	920456	929686	NC_017628.1	IHE3034	
ESCO001.0321.01463.C001.PICI.TypeA.Set1.prt	2722573	2731800	NC_017632.1	UM146	
ESCO001.0321.01464.C001.PICI.TypeA.Set1.prt	249094	258776	NC_017651.1	clone D i2	
ESCO001.0321.01465.C001.PICI.TypeA.Set1.prt	249094	258776	NC_017651.1	clone D i2	
ESCO001.0321.01476.C001.PICI.TypeA.Set2.prt	3233341	3243240	NZ_CP031919.1	FWSEC0002	
ESCO001.0321.01481.C001.PICI.TypeA.Set1.prt	3222497	3232396	NZ_CP015832.1	180-PT54	
ESCO001.0321.01482.C001.PICI.TypeA.Set1.prt	3475888	3485787	NZ_CP015831.1	644-PT8	
ESCO001.0321.01482.C001.PICI.TypeA.Set2.prt	2674204	2684103	unknown	unknown	
ESCO001.0321.01483.C001.PICI.TypeA.Set3.prt	4607537	4617436	NZ_CP022050.2	FDAARGOS_293	
ESCO001.0321.01484.C001.PICI.TypeA.Set1.prt	900756	910655	NZ_CP043539.1	Al Ain	
ESCO001.0321.01488.C001.PICI.TypeA.Set1.prt	5285694	5295593	NZ_CP044148.1	AR-0427	

ESCO001.0321.01488.C001.PICI.TypeA.Set5.prt	4798373	4806305	NZ_CP044148.1	AR-0427
ESCO001.0321.01490.C001.PICI.TypeA.Set1.prt	1438336	1448236	NC_017656.1	RM12579
ESCO001.0321.01491.C001.PICI.TypeA.Set1.prt	1623369	1633147	NZ_CP028116.1	RM8426
ESCO001.0321.01491.C001.PICI.TypeA.Set3.prt	3009193	3017381	NZ_CP028116.1	RM8426
ESCO001.0321.01492.C001.PICI.TypeA.Set1.prt	1672486	1682264	NZ_CP028126.1	RM10386
ESCO001.0321.01492.C001.PICI.TypeA.Set2.prt	3063066	3071256	NZ_CP028126.1	RM10386
ESCO001.0321.01493.C001.PICI.TypeA.Set3.prt	2218746	2228645	NZ_CP031343.1	RM10425-C1
ESCO001.0321.01494.C001.PICI.TypeA.Set3.prt	3320884	3332095	NZ_CP031355.1	RM8995-C1
ESCO001.0321.01495.C001.PICI.TypeA.Set3.prt	1496784	1506682	NZ_CP035767.1	RM12522-C8
ESCO001.0321.01496.C001.PICI.TypeB.variant0001.Set2.prt	1491523	1501212	NZ_CP031341.1	RM12275-C1
ESCO001.0321.01497.C001.PICI.TypeA.Set2.prt	2073799	2085011	NZ_CP035772.1	RM8843-C1
ESCO001.0321.01498.C001.PICI.TypeA.Set1.prt	1747508	1758720	NZ_CP035770.1	RM8988-C1
ESCO001.0321.01499.C001.PICI.TypeA.Set1.prt	3292026	3301925	NZ_CP035768.1	RM11626-C1
ESCO001.0321.01500.P002.PICI.TypeA.Set1.prt	30167	40066	NZ_CP031350.1	RM9154-C1
ESCO001.0321.01501.C001.PICI.TypeA.Set1.prt	1445423	1455322	NZ_CP031349.1	RM9467-C1
ESCO001.0321.01502.C001.PICI.TypeA.Set2.prt	1446669	1456567	NZ_CP031345.1	RM12367-C1
ESCO001.0321.01504.C001.PICI.TypeA.Set3.prt	862281	870474	NZ_CP028112.1	RM8385
ESCO001.0321.01506.C001.PICI.TypeA.Set1.prt	2673279	2682214	NZ_CP028117.1	RM9322
ESCO001.0321.01508.C001.PICI.TypeA.Set1.prt	1445430	1455329	NZ_CP028379.1	RM9872
ESCO001.0321.01509.C001.PICI.TypeA.Set2.prt	2458228	2467406	NZ_CP015244.1	RM7190
ESCO001.0321.01512.C001.PICI.TypeA.Set2.prt	2347105	2356874	NZ_CP031916.1	FWSEC0003
ESCO001.0321.01518.C001.PICI.TypeA.Set3.prt	1462425	1472324	NZ_CP006027.1	RM13514
ESCO001.0321.01519.C001.PICI.TypeA.Set3.prt	1462425	1472324	NZ_CP006027.1	RM13514
ESCO001.0321.01520.C001.PICI.TypeA.Set1.prt	1438956	1448854	NZ_CP006262.1	RM13516
ESCO001.0321.01521.C001.PICI.TypeA.Set1.prt	1438956	1448854	NZ_CP006262.1	RM13516
ESCO001.0321.01524.C001.PICI.TypeA.Set1.prt	106836	116987	NZ_CP009072.1	ATCC 25922
ESCO001.0321.01525.C001.PICI.TypeB.variant0003.Set1.prt	1533870	1543766	NZ_CP008805.1	SS17
ESCO001.0321.01527.C001.PICI.TypeA.Set1.prt	1534662	1544561	NZ_CP010304.1	SS52
ESCO001.0321.01528.C001.PICI.TypeB.variant0003.Set1.prt	805755	815469	NZ_CP023541.1	ATCC BAA-178
ESCO001.0321.01530.C001.PICI.TypeA.Set1.prt	3167781	3177668	NZ_CP038355.1	F8092B
ESCO001.0321.01530.C001.PICI.TypeA.Set3.prt	3678515	3686984	NZ_CP038355.1	F8092B
ESCO001.0321.01548.C001.PICI.TypeA.Set3.prt	384840	394045	NZ_CP024240.1	90-9280
ESCO001.0321.01554.C001.PICI.TypeB.variant0001.Set1.prt	2657630	2666460	NZ_CP028320.1	CFSAN067215
ESCO001.0321.01586.C001.PICI.TypeA.Set2.prt	3160531	3170116	NZ_CP062855.1	Res13-Lact-PEB17-18
EUCA001.0321.00001.C001.PICI.TypeB.variant0002.Set1.prt	2990924	3008132	NC_014624.2	KIST612
FRLI001.0321.00001.C001.PICI.TypeB.variant0002.Set1.prt	1306893	1314942	NZ_CP014872.1	TMW 1.1993

FRTU001.0321.00029.C001.PICI.TypeB.variant0002.Set1.prt	9372	15027	NC_008601.1	U112	
FRTU001.0321.00030.C001.PICI.TypeB.variant0002.Set1.prt	205124	210779	NZ_CP009633.1	U112	
FUMA001.0321.00001.C001.PICI.TypeA.Set1.prt	155772	163627	NZ_CP017641.1	NH11	
FUNU001.0321.00006.C001.PICI.TypeB.variant0002.Set1.prt	1490353	1501298	NZ_LN831027.1	NCTC10562	
FUPS001.0321.00003.C001.PICI.TypeB.variant0002.Set1.prt	1152955	1163775	NZ_CP024700.1	KCOM 1263	
FUPS001.0321.00008.C001.PICI.TypeB.variant0002.Set1.prt	1196122	1206942	NZ_CP024731.1	KCOM 1262	
FUPS001.0321.00009.C001.PICI.TypeB.variant0002.Set1.prt	272892	282135	NZ_CP024705.1	KCOM 2653	
GEMA001.0321.00001.C001.PICI.TypeB.variant0002.Set4.prt	1362619	1372124	NZ_LR593886.1	unknown	
GIBE001.0321.00001.C001.PICI.TypeB.variant0004.Set1.prt	18393	26413	NZ_CP043930.1	E7	
GOPH001.0321.00001.C001.PICI.TypeB.variant0001.Set1.prt	3572243	3582296	CP011853.1	QH-11	
GOPH001.0321.00001.C001.PICI.TypeB.variant0001.Set2.prt	2022643	2031874	NZ_CP011853.1	QH-11	
GOPO001.0321.00001.C001.PICI.TypeB.variant0001.Set1.prt	2919788	2926936	NC_016906.1	VH2	
GOSP001.0321.00001.C001.PICI.TypeB.variant0001.Set1.prt	3813344	3822993	CP025435.1	YC-JH1	
GOSP003.0321.00001.C001.PICI.TypeB.variant0001.Set1.prt	1376610	1385849	NZ_CP046257.1		135
GOTE001.0321.00001.C001.PICI.TypeB.variant0001.Set1.prt	3954715	3962205	CP016594.1		3612
GOTE001.0321.00002.C001.PICI.TypeB.variant0001.Set1.prt	3957556	3965046	CP029604.1	NRRL B-16283	
HECO001.0321.00001.C001.PICI.TypeB.variant0001.Set1.prt	1232433	1238927	NZ_CP045875.1	HH	
HUSA001.0321.00001.C001.PICI.TypeB.variant0001.Set1.prt	3538005	3544324	NZ_CP025197.1	GGR1	
HYNI001.0321.00001.C001.PICI.TypeA.Set1.prt	57670	65201	NC_022997.1	NL23	
JESP002.0321.00001.C001.PICI.TypeB.variant0002.Set2.prt	996311	1003169	NZ_CP019433.1	PTS2502	
KOPA002.0321.00001.C001.PICI.TypeB.variant0003.Set1.prt	1098156	1105185	NZ_CP012507.1	MU14/1	
KORO001.0321.00001.C001.PICI.TypeB.variant0001.Set1.prt	1476227	1483862	NZ_CP035103.1	ATCC 186	
LACR001.0321.00002.C001.PICI.TypeB.variant0001.Set1.prt	315741	329329	NZ_CP015907.1	UC109	
LACR001.0321.00003.C001.PICI.TypeB.variant0002.Set1.prt	282531	294774	NZ_CP015899.1	JM1	
LACR001.0321.00005.C001.PICI.TypeB.variant0002.Set1.prt	2242269	2254010	NZ_CP015901.1	JM3	
LACR001.0321.00009.C001.PICI.TypeB.variant0002.Set4.prt	34991	48238	NZ_CP032430.1	W34	
LADE001.0321.00007.C001.PICI.TypeB.variant0002.Set1.prt	377713	389963	NZ_CP032451.1	KLDS1.0207	
LADE001.0321.00008.C001.PICI.TypeB.variant0002.Set1.prt	373468	385713	NZ_CP041280.1	KLDS1.1011	
LAHE001.0321.00001.C001.PICI.TypeB.variant0002.Set1.prt	1258082	1272591	NZ_CP029544.1	ESL0183	
LALA002.0321.00013.C001.PICI.TypeB.variant0002.Set1.prt	2117065	2128806	NC_008527.1	SK11	
LALA002.0321.00014.C001.PICI.TypeB.variant0002.Set1.prt	2212957	2227565	NC_009004.1	MG1363	
LALA002.0321.00015.C001.PICI.TypeB.variant0002.Set1.prt	2213774	2228382	NC_017949.1	NZ9000	
LAPL001.0321.00073.C001.PICI.TypeB.variant0003.SetR1.prt	1110559	1137672	NZ_CP035223.1	SRCM103472	
LAPL001.0321.00074.C001.PICI.TypeB.variant0003.SetR1.prt	2164110	2191222	NZ_CP035224.1	SRCM103473	
LARA001.0321.00005.C001.PICI.TypeB.variant0002.Set1.prt	529393	549207	NZ_CP047628.1	Lr_19_14	
LARA001.0321.00006.C001.PICI.TypeB.variant0002.Set1.prt	777326	794297	NZ_CP047630.1	Lr_18_12S	

LEFE001.0321.00001.C001.PICI.TypeB.variant0002.Set1.prt	809421	819518	NC_018649.1	ML-04
LOBA001.0321.00003.P004.PICI.TypeB.variant0002.Set1.prt	4557	27251	NZ_CP014886.1	TMW 1.1991
MAAU001.0321.00001.C001.PICI.TypeB.variant0002.Set1.prt	590265	601949	NC_015520.1	50-1 BON
MACA003.0321.00002.C001.PICI.TypeB.variant0002.Set1.prt	1374617	1383200	NZ_CP065729.1	FDAARGOS_868
MAOV001.0321.00001.C001.PICI.TypeB.variant0002.Set1.prt	1460816	1470634	CP046531.1	ZY170218
MASC001.0321.00003.C001.PICI.TypeB.variant0002.Set1.prt	487977	496547	NZ_CP065792.1	GDK8D6P
MASP004.0321.00001.C001.PICI.TypeB.variant0001.Set1.prt	3994917	4003621	NZ_CP030092.1	YMA4
MASP013.0321.00001.C001.PICI.TypeB.variant0002.Set2.prt	919218	928682	NZ_CP017156.1	IME1552
MAST001.0321.00001.C001.PICI.TypeB.variant0002.Set1.prt	1972397	1980346	NZ_LT906462.1	NCTC13839
MESP018.0321.00001.C001.PICI.TypeB.variant0001.Set1.prt	880247	888218	NZ_CP027667.1	SC2-9
MODI001.0321.00001.C001.PICI.TypeB.variant0002.Set1.prt	1115678	1131601	NZ_CP027228.1	CCUG 47132
MYAB001.0321.00012.C001.PICI.TypeB.variant0001.Set1.prt	2786707	2792885	NZ_CP014960.1	FLAC049
MYAB001.0321.00045.C001.PICI.TypeB.variant0001.Set5.prt	2482488	2492021	NZ_CP065266.1	GD69B
MYAB001.0321.00046.C001.PICI.TypeB.variant0001.Set1.prt	2482487	2492020	NZ_CP065269.1	GD69A
MYAL002.0321.00001.C001.PICI.TypeB.variant0001.Set2.prt	4614584	4624755	NZ_AP022565.1	JCM 12272
MYBO001.0321.00001.C001.PICI.TypeB.variant0001.Set4.prt	3125676	3133981	NZ_AP022579.1	JCM 15653
MYCE001.0321.00001.C001.PICI.TypeB.variant0001.Set2.prt	96692	105665	NZ_AP022591.1	JCM 18439
MYCE001.0321.00001.C001.PICI.TypeB.variant0004.Set1.prt	1909930	1917548	NZ_AP022591.1	JCM 18439
MYCH002.0321.00001.C001.PICI.TypeB.variant0001.Set1.prt	4533165	4542654	NZ_AP022604.1	JCM 12403
MYCH002.0321.00002.C001.PICI.TypeB.variant0001.Set1.prt	2964098	2973587	NZ_LR134355.1	NCTC10485
MYCO005.0321.00001.C001.PICI.TypeB.variant0001.Set2.prt	2870679	2876304	AP022612.1	JCM 13671
MYCO006.0321.00001.C001.PICI.TypeB.variant0001.Set1.prt	969063	974768	NZ_CP020821.1	CECT 3035
MYCO007.0321.00001.C001.PICI.TypeB.variant0001.Set3.prt	170774	176631	NZ_AP022613.1	JCM 14738
MYDI001.0321.00001.C001.PICI.TypeB.variant0001.Set1.prt	6248325	6258271	CP020809.1	PH-06
MYFA001.0321.00001.C001.PICI.TypeB.variant0001.Set2.prt	3237821	3245918	NZ_AP022603.1	JCM 6405
MYFO001.0321.00001.C001.PICI.TypeB.variant0001.Set1.prt	1134279	1142454	NZ_CP011269.1	CT6
MYFO001.0321.00001.C001.PICI.TypeB.variant0001.Set2.prt	3167659	3177402	NZ_CP011269.1	CT6
MYGA004.0321.00001.C001.PICI.TypeB.variant0001.Set4.prt	4004347	4012623	AP022601.1	JCM 6399
MYGI001.0321.00002.C001.PICI.TypeB.variant0001.Set2.prt	4157180	4165290	CP000656.1	PYR-GCK
MYHA005.0321.00001.C001.PICI.TypeB.variant0001.Set2.prt	2435782	2441422	NZ_LR026975.1	unknown
MYHI001.0321.00001.C001.PICI.TypeB.variant0003.Set1.prt	1327983	1335359	NZ_AP022609.1	JCM 13571
MYIN001.0321.00004.C001.PICI.TypeB.variant0001.Set1.prt	1613461	1619284	NZ_AP024254.1	M016
MYIN001.0321.00005.C001.PICI.TypeB.variant0001.Set1.prt	417934	424436	NZ_AP024255.1	M018
MYIN001.0321.00011.C001.PICI.TypeB.variant0001.Set3.prt	1693948	1699771	NZ_AP024244.1	M005
MYIN001.0321.00024.C001.PICI.TypeA.Set1.prt	3609477	3616908	NC_016946.1	ATCC 13950
MYLI002.0321.00002.C001.PICI.TypeB.variant0001.Set1.prt	5115855	5125891	NZ_AP023287.1	NIIDNTM18

MYMA004.0321.00001.C001.PICI.TypeB.variant0001.Set3.prt	5143588	5153641	NZ_AP022567.1	JCM 12375	
MYMA005.0321.00001.C001.PICI.TypeB.variant0001.Set1.prt	2870763	2879270	NZ_AP022590.1	JCM 18113	
MYNO002.0321.00001.C001.PICI.TypeB.variant0001.Set1.prt	3633784	3639197	NZ_AP022583.1	JCM 16367	
MYPA001.0321.00005.C001.PICI.TypeB.variant0001.Set1.prt	3293517	3303240	NZ_AP024257.1	M019	
MYPA001.0321.00005.C001.PICI.TypeB.variant0001.Set3.prt	3834528	3839694	NZ_AP024257.1	M019	
MYPA002.0321.00001.C001.PICI.TypeB.variant0001.Set3.prt	2980464	2987770	NZ_CP025546.1		49061
MYPO001.0321.00001.C001.PICI.TypeB.variant0001.Set2.prt	3389340	3396530	NZ_AP022570.1	JCM 12603	
MYPS001.0321.00001.C001.PICI.TypeB.variant0001.Set2.prt	2482405	2491820	NZ_AP022574.1	JCM 13323	
MYSH002.0321.00001.C001.PICI.TypeB.variant0001.Set1.prt	202555	212326	NZ_AP022575.1	JCM 14233	
MYSP003.0321.00001.C001.PICI.TypeB.variant0001.Set1.prt	3676602	3682751	NZ_CP011773.1	EPa45	
MYSP007.0321.00001.C001.PICI.TypeB.variant0001.Set2.prt	5466247	5476312	NZ_CP015596.1	YC-RL4	
MYSP009.0321.00001.C001.PICI.TypeB.variant0001.Set1.prt	3211232	3221119	unknown	unknown	
MYSP019.0321.00001.C001.PICI.TypeB.variant0001.Set2.prt	5341918	5360418	NZ_CP065373.1	Z-34	
MYTU002.0321.00001.C001.PICI.TypeB.variant0004.Set1.prt	1771537	1779399	NZ_CP015773.2	SP38	
MYTU002.0321.00002.C001.PICI.TypeB.variant0004.Set1.prt	1770543	1778405	NZ_CP012095.1		1595
MYTU002.0321.00004.C001.PICI.TypeB.variant0004.Set1.prt	1774603	1782465	NZ_CP027035.1	2002/0476	
MYTU002.0321.00008.C001.PICI.TypeB.variant0003.Set1.prt	2964389	2971412	NZ_CP007809.1	KIT87190	
MYTU002.0321.00009.C001.PICI.TypeB.variant0003.Set1.prt	2973805	2980828	NC_018143.2	H37Rv	
MYTU002.0321.00009.C001.PICI.TypeB.variant0004.Set2.prt	1780623	1788485	NZ_CP009100.1	ZMC13-264	
MYTU002.0321.00010.C001.PICI.TypeB.variant0003.Set1.prt	2973816	2980839	NZ_CP009101.1	ZMC13-88	
MYTU002.0321.00010.C001.PICI.TypeB.variant0004.Set2.prt	1780639	1788501	NZ_CP009101.1	ZMC13-88	
MYTU002.0321.00011.C001.PICI.TypeB.variant0003.Set1.prt	2951080	2958103	NZ_CP009426.1		96075
MYTU002.0321.00012.C001.PICI.TypeB.variant0003.Set2.prt	2982644	2989667	NZ_CP009427.1		96121
MYTU002.0321.00012.C001.PICI.TypeB.variant0004.Set1.prt	1780195	1788057	NZ_CP009427.1		96121
MYTU002.0321.00013.C001.PICI.TypeB.variant0003.Set1.prt	2968818	2975841	NZ_CP012506.2	SCAID 187.0	
MYTU002.0321.00014.C001.PICI.TypeB.variant0003.Set2.prt	2982807	2989830	NZ_CP010330.1	F28	
MYTU002.0321.00014.C001.PICI.TypeB.variant0004.Set1.prt	1782130	1789992	NZ_CP010330.1	F28	
MYTU002.0321.00015.C001.PICI.TypeB.variant0003.Set1.prt	2976662	2984066	NZ_CP046529.1	SIT745/EAI1-MYS	
MYTU002.0321.00015.C001.PICI.TypeB.variant0004.Set2.prt	1779653	1787515	NZ_CP046529.1	SIT745/EAI1-MYS	
MYTU002.0321.00016.C001.PICI.TypeB.variant0003.Set1.prt	2969627	2976650	NZ_CP016794.1	SCAID 320.0	
MYTU002.0321.00017.C001.PICI.TypeB.variant0003.Set1.prt	2971798	2978821	NZ_CP016888.1	SCAID 252.0	
MYTU002.0321.00018.C001.PICI.TypeB.variant0003.Set1.prt	389660	396683	NZ_CP011510.1	Beijing	
MYTU002.0321.00019.C001.PICI.TypeB.variant0003.Set1.prt	2955505	2962528	NZ_CP013475.1		1458
MYTU002.0321.00020.C001.PICI.TypeB.variant0003.Set1.prt	396957	403981	NZ_CP017920.1	TB282	
MYTU002.0321.00021.C001.PICI.TypeB.variant0003.Set1.prt	2957036	2964059	NZ_CP018303.1	I0004241-1	
MYTU002.0321.00022.C001.PICI.TypeB.variant0003.Set1.prt	2957793	2964816	NZ_CP018305.1	M0018684-2	

MYTU002.0321.00023.C001.PICI.TypeB.variant0003.Set1.prt	2957937	2964960	NZ_CP018302.1	I0004000-1
MYTU002.0321.00024.C001.PICI.TypeB.variant0003.Set1.prt	2953984	2961007	NZ_CP018301.1	I0002801-4
MYTU002.0321.00024.C001.PICI.TypeB.variant0004.Set2.prt	3853971	3861833	NZ_CP018301.1	I0002801-4
MYTU002.0321.00025.C001.PICI.TypeB.variant0003.Set1.prt	2957957	2964980	NZ_CP018300.1	I0002353-6
MYTU002.0321.00026.C001.PICI.TypeB.variant0003.Set1.prt	2961319	2968342	NZ_CP018304.1	M0002959-6
MYTU002.0321.00027.C001.PICI.TypeB.variant0003.Set2.prt	2973798	2980821	NZ_CP018778.1	DK9897
MYTU002.0321.00028.C001.PICI.TypeB.variant0003.Set2.prt	2973341	2980364	NZ_CP020381.2	MTB1
MYTU002.0321.00029.C001.PICI.TypeB.variant0003.Set1.prt	2981090	2988113	NZ_CP017593.1	Beijing-like/35049
MYTU002.0321.00030.C001.PICI.TypeB.variant0003.Set1.prt	2994923	3001946	NZ_CP017594.1	Beijing-like/36918
MYTU002.0321.00031.C001.PICI.TypeB.variant0003.Set1.prt	2984222	2991245	NZ_CP017595.1	Beijing-like/38774
MYTU002.0321.00032.C001.PICI.TypeB.variant0003.Set1.prt	2966986	2974009	NZ_CP017596.1	Beijing/391
MYTU002.0321.00033.C001.PICI.TypeB.variant0003.Set1.prt	2998724	3005747	NZ_CP017597.1	Beijing-like/50148
MYTU002.0321.00034.C001.PICI.TypeB.variant0003.Set1.prt	2971460	2978483	NZ_CP017598.1	Beijing-like/1104
MYTU002.0321.00035.C001.PICI.TypeB.variant0003.Set1.prt	2977064	2984087	NZ_CP022014.1	MTB2
MYTU002.0321.00036.C001.PICI.TypeB.variant0003.Set1.prt	2959726	2966749	NZ_AP017901.1	NCGM946K2
MYTU002.0321.00038.C001.PICI.TypeB.variant0003.Set1.prt	2960805	2967828	NZ_AP018034.1	HN-205
MYTU002.0321.00039.C001.PICI.TypeB.variant0003.Set1.prt	2974342	2981365	NZ_AP018035.1	HN-321
MYTU002.0321.00040.C001.PICI.TypeB.variant0003.Set1.prt	2962595	2969618	NZ_AP018036.1	HN-506
MYTU002.0321.00041.C001.PICI.TypeB.variant0003.Set1.prt	2973564	2980587	NZ_CP023573.1	CSV4519
MYTU002.0321.00041.C001.PICI.TypeB.variant0004.Set2.prt	1780459	1788321	NZ_CP023573.1	CSV4519
MYTU002.0321.00042.C001.PICI.TypeB.variant0003.Set2.prt	2973603	2980626	NZ_CP023574.1	CSV4644
MYTU002.0321.00042.C001.PICI.TypeB.variant0004.Set1.prt	1780519	1788381	NZ_CP023574.1	CSV4644
MYTU002.0321.00043.C001.PICI.TypeB.variant0003.Set2.prt	2973638	2980661	NZ_CP023575.1	CSV5769
MYTU002.0321.00043.C001.PICI.TypeB.variant0004.Set1.prt	1780495	1788357	NZ_CP023575.1	CSV5769
MYTU002.0321.00044.C001.PICI.TypeB.variant0003.Set1.prt	2973525	2980548	NZ_CP023576.1	CSV10399
MYTU002.0321.00044.C001.PICI.TypeB.variant0004.Set2.prt	1780432	1788294	NZ_CP023576.1	CSV10399
MYTU002.0321.00045.C001.PICI.TypeB.variant0003.Set2.prt	2973718	2980741	NZ_CP023577.1	CSV11678
MYTU002.0321.00045.C001.PICI.TypeB.variant0004.Set1.prt	1780591	1788453	NZ_CP023577.1	CSV11678
MYTU002.0321.00046.C001.PICI.TypeB.variant0003.Set1.prt	2973495	2980518	NZ_CP023578.1	LE486
MYTU002.0321.00046.C001.PICI.TypeB.variant0004.Set2.prt	1780422	1788284	NZ_CP023578.1	LE486
MYTU002.0321.00047.C001.PICI.TypeB.variant0003.Set1.prt	2973512	2980535	NZ_CP023579.1	LE492
MYTU002.0321.00047.C001.PICI.TypeB.variant0004.Set2.prt	1780439	1788301	NZ_CP023579.1	LE492
MYTU002.0321.00048.C001.PICI.TypeB.variant0003.Set2.prt	2973723	2980746	NZ_CP023580.1	LN180
MYTU002.0321.00048.C001.PICI.TypeB.variant0004.Set1.prt	1780596	1788458	NZ_CP023580.1	LN180
MYTU002.0321.00049.C001.PICI.TypeB.variant0003.Set1.prt	2973676	2980699	NZ_CP023581.1	LN2358
MYTU002.0321.00049.C001.PICI.TypeB.variant0004.Set2.prt	1780521	1788383	NZ_CP023581.1	LN2358

MYTU002.0321.00050.C001.PICI.TypeB.variant0003.Set2.prt	2973646	2980669	NZ_CP023582.1	LN3756
MYTU002.0321.00050.C001.PICI.TypeB.variant0004.Set1.prt	1780516	1788378	NZ_CP023582.1	LN3756
MYTU002.0321.00051.C001.PICI.TypeB.variant0003.Set2.prt	2973611	2980634	NZ_CP023583.1	MDRDM260
MYTU002.0321.00051.C001.PICI.TypeB.variant0004.Set1.prt	1780480	1788342	NZ_CP023583.1	MDRDM260
MYTU002.0321.00052.C001.PICI.TypeB.variant0003.Set1.prt	2973558	2980581	NZ_CP023584.1	MDRDM627
MYTU002.0321.00052.C001.PICI.TypeB.variant0004.Set2.prt	1780489	1788351	NZ_CP023584.1	MDRDM627
MYTU002.0321.00053.C001.PICI.TypeB.variant0003.Set2.prt	2973489	2980512	NZ_CP023585.1	MDRDM1098
MYTU002.0321.00053.C001.PICI.TypeB.variant0004.Set1.prt	1780413	1788275	NZ_CP023585.1	MDRDM1098
MYTU002.0321.00054.C001.PICI.TypeB.variant0003.Set1.prt	2973476	2980499	NZ_CP023586.1	MDRMA2491
MYTU002.0321.00054.C001.PICI.TypeB.variant0004.Set2.prt	1780356	1788218	NZ_CP023586.1	MDRMA2491
MYTU002.0321.00055.C001.PICI.TypeB.variant0003.Set1.prt	2973535	2980558	NZ_CP023587.1	ME1473
MYTU002.0321.00055.C001.PICI.TypeB.variant0004.Set2.prt	1780455	1788317	NZ_CP023587.1	ME1473
MYTU002.0321.00056.C001.PICI.TypeB.variant0003.Set1.prt	2973460	2980483	NZ_CP023588.1	TBDM425
MYTU002.0321.00056.C001.PICI.TypeB.variant0004.Set2.prt	1780383	1788245	NZ_CP023588.1	TBDM425
MYTU002.0321.00057.C001.PICI.TypeB.variant0003.Set2.prt	2973587	2980610	NZ_CP023589.1	TBV5000
MYTU002.0321.00057.C001.PICI.TypeB.variant0004.Set1.prt	1780499	1788361	NZ_CP023589.1	TBV5000
MYTU002.0321.00058.C001.PICI.TypeB.variant0003.Set2.prt	2973614	2980637	NZ_CP023590.1	TBV5362
MYTU002.0321.00058.C001.PICI.TypeB.variant0004.Set1.prt	1780532	1788394	NZ_CP023590.1	TBV5362
MYTU002.0321.00059.C001.PICI.TypeB.variant0003.Set1.prt	2973690	2980713	NZ_CP023591.1	TBV5365
MYTU002.0321.00059.C001.PICI.TypeB.variant0004.Set2.prt	1780587	1788449	NZ_CP023591.1	TBV5365
MYTU002.0321.00060.C001.PICI.TypeB.variant0003.Set1.prt	2973611	2980634	NZ_CP023583.1	MDRDM260
MYTU002.0321.00060.C001.PICI.TypeB.variant0004.Set2.prt	1780503	1788365	NZ_CP023592.1	SLM036
MYTU002.0321.00061.C001.PICI.TypeB.variant0003.Set2.prt	2973729	2980752	NZ_CP023593.1	SLM040
MYTU002.0321.00061.C001.PICI.TypeB.variant0004.Set1.prt	1780594	1788456	NZ_CP023593.1	SLM040
MYTU002.0321.00062.C001.PICI.TypeB.variant0003.Set2.prt	2973705	2980728	NZ_CP023594.1	SLM056
MYTU002.0321.00062.C001.PICI.TypeB.variant0004.Set1.prt	1780566	1788428	NZ_CP023594.1	SLM056
MYTU002.0321.00063.C001.PICI.TypeB.variant0003.Set2.prt	2973502	2980525	NZ_CP023595.1	SLM060
MYTU002.0321.00063.C001.PICI.TypeB.variant0004.Set1.prt	1780380	1788242	NZ_CP023595.1	SLM060
MYTU002.0321.00064.C001.PICI.TypeB.variant0003.Set1.prt	2973700	2980723	NZ_CP023596.1	SLM063
MYTU002.0321.00064.C001.PICI.TypeB.variant0004.Set2.prt	1780563	1788425	NZ_CP023596.1	SLM063
MYTU002.0321.00065.C001.PICI.TypeB.variant0003.Set1.prt	2973729	2980752	NZ_CP023593.1	SLM040
MYTU002.0321.00065.C001.PICI.TypeB.variant0004.Set2.prt	1780593	1788455	NZ_CP023597.1	SLM088
MYTU002.0321.00066.C001.PICI.TypeB.variant0003.Set2.prt	2973729	2980752	NZ_CP023593.1	SLM040
MYTU002.0321.00066.C001.PICI.TypeB.variant0004.Set1.prt	1780593	1788455	NZ_CP023597.1	SLM088
MYTU002.0321.00067.C001.PICI.TypeB.variant0003.Set1.prt	2973486	2980509	NZ_CP023599.1	CSV383
MYTU002.0321.00067.C001.PICI.TypeB.variant0004.Set2.prt	1780411	1788273	NZ_CP023599.1	CSV383

MYTU002.0321.00068.C001.PICI.TypeB.variant0003.Set2.prt	2973699	2980722	NZ_CP023600.1	CSV3611
MYTU002.0321.00068.C001.PICI.TypeB.variant0004.Set1.prt	1780567	1788429	NZ_CP023600.1	CSV3611
MYTU002.0321.00069.C001.PICI.TypeB.variant0003.Set2.prt	2973630	2980653	NZ_CP023601.1	CSV9577
MYTU002.0321.00069.C001.PICI.TypeB.variant0004.Set1.prt	1780544	1788406	NZ_CP023601.1	CSV9577
MYTU002.0321.00070.C001.PICI.TypeB.variant0003.Set2.prt	2973695	2980718	NZ_CP023602.1	LE13
MYTU002.0321.00070.C001.PICI.TypeB.variant0004.Set1.prt	1780618	1788480	NZ_CP023602.1	LE13
MYTU002.0321.00071.C001.PICI.TypeB.variant0003.Set1.prt	2973717	2980740	NZ_CP023603.1	LE63
MYTU002.0321.00071.C001.PICI.TypeB.variant0004.Set2.prt	1780574	1788436	NZ_CP023603.1	LE63
MYTU002.0321.00072.C001.PICI.TypeB.variant0003.Set1.prt	2973537	2980560	NZ_CP023604.1	LE76
MYTU002.0321.00072.C001.PICI.TypeB.variant0004.Set2.prt	1780497	1788359	NZ_CP023604.1	LE76
MYTU002.0321.00073.C001.PICI.TypeB.variant0003.Set1.prt	2973747	2980770	NZ_CP023605.1	LE79
MYTU002.0321.00073.C001.PICI.TypeB.variant0004.Set2.prt	1780618	1788480	NZ_CP023602.1	LE13
MYTU002.0321.00074.C001.PICI.TypeB.variant0003.Set2.prt	2973639	2980662	NZ_CP023606.1	LE103
MYTU002.0321.00074.C001.PICI.TypeB.variant0004.Set1.prt	1780500	1788362	NZ_CP023606.1	LE103
MYTU002.0321.00075.C001.PICI.TypeB.variant0003.Set1.prt	2973544	2980567	NZ_CP023607.1	LE371
MYTU002.0321.00075.C001.PICI.TypeB.variant0004.Set2.prt	1780418	1788280	NZ_CP023607.1	LE371
MYTU002.0321.00076.C001.PICI.TypeB.variant0003.Set2.prt	2970460	2979218	NZ_CP023608.1	LE410
MYTU002.0321.00076.C001.PICI.TypeB.variant0004.Set1.prt	1780557	1788419	NZ_CP023608.1	LE410
MYTU002.0321.00077.C001.PICI.TypeB.variant0003.Set2.prt	2973555	2980578	NZ_CP023609.1	LN55
MYTU002.0321.00077.C001.PICI.TypeB.variant0004.Set1.prt	1780470	1788332	NZ_CP023609.1	LN55
MYTU002.0321.00078.C001.PICI.TypeB.variant0003.Set1.prt	2973665	2980688	NZ_CP023610.1	LN317
MYTU002.0321.00078.C001.PICI.TypeB.variant0004.Set2.prt	1780527	1788389	NZ_CP023610.1	LN317
MYTU002.0321.00079.C001.PICI.TypeB.variant0003.Set1.prt	2973595	2980618	NZ_CP023611.1	LN763
MYTU002.0321.00079.C001.PICI.TypeB.variant0004.Set2.prt	1780509	1788371	NZ_CP023611.1	LN763
MYTU002.0321.00080.C001.PICI.TypeB.variant0003.Set1.prt	2973680	2980703	NZ_CP023612.1	LN2978
MYTU002.0321.00080.C001.PICI.TypeB.variant0004.Set2.prt	1780536	1788398	NZ_CP023612.1	LN2978
MYTU002.0321.00081.C001.PICI.TypeB.variant0003.Set1.prt	2973676	2980699	NZ_CP023581.1	LN2358
MYTU002.0321.00081.C001.PICI.TypeB.variant0004.Set2.prt	1780556	1788418	NZ_CP023613.1	LN3584
MYTU002.0321.00082.C001.PICI.TypeB.variant0003.Set2.prt	2973658	2980681	NZ_CP023614.1	LN3588
MYTU002.0321.00082.C001.PICI.TypeB.variant0004.Set1.prt	1780566	1788428	NZ_CP023594.1	SLM056
MYTU002.0321.00083.C001.PICI.TypeB.variant0003.Set2.prt	2973672	2980695	NZ_CP023615.1	LN3589
MYTU002.0321.00083.C001.PICI.TypeB.variant0004.Set1.prt	1780615	1788477	NZ_CP023615.1	LN3589
MYTU002.0321.00084.C001.PICI.TypeB.variant0003.Set1.prt	2973773	2980796	NZ_CP023616.1	LN3668
MYTU002.0321.00084.C001.PICI.TypeB.variant0004.Set2.prt	1780607	1788469	NZ_CP023616.1	LN3668
MYTU002.0321.00085.C001.PICI.TypeB.variant0003.Set1.prt	2973747	2980770	NZ_CP023605.1	LE79
MYTU002.0321.00085.C001.PICI.TypeB.variant0004.Set2.prt	1780610	1788472	NZ_CP023617.1	LN3672

MYTU002.0321.00086.C001.PICI.TypeB.variant0003.Set1.prt	2973723	2980746	NZ_CP023580.1	LN180
MYTU002.0321.00086.C001.PICI.TypeB.variant0004.Set2.prt	1780596	1788458	NZ_CP023580.1	LN180
MYTU002.0321.00087.C001.PICI.TypeB.variant0003.Set2.prt	2973732	2980755	NZ_CP023619.1	LN1100
MYTU002.0321.00087.C001.PICI.TypeB.variant0004.Set1.prt	1780597	1788459	NZ_CP023619.1	LN1100
MYTU002.0321.00088.C001.PICI.TypeB.variant0003.Set1.prt	2973583	2980606	NZ_CP023620.1	LN1856
MYTU002.0321.00088.C001.PICI.TypeB.variant0004.Set2.prt	1780496	1788358	NZ_CP023620.1	LN1856
MYTU002.0321.00089.C001.PICI.TypeB.variant0003.Set1.prt	2973594	2980617	NZ_CP023621.1	LN2900
MYTU002.0321.00089.C001.PICI.TypeB.variant0004.Set2.prt	1780513	1788375	NZ_CP023621.1	LN2900
MYTU002.0321.00090.C001.PICI.TypeB.variant0003.Set2.prt	2973584	2980607	NZ_CP023622.1	MDRDM827
MYTU002.0321.00090.C001.PICI.TypeB.variant0004.Set1.prt	1780501	1788363	NZ_CP023622.1	MDRDM827
MYTU002.0321.00091.C001.PICI.TypeB.variant0003.Set1.prt	2973687	2980710	NZ_CP023623.1	MDRMA203
MYTU002.0321.00091.C001.PICI.TypeB.variant0004.Set2.prt	1780568	1788430	NZ_CP023623.1	MDRMA203
MYTU002.0321.00092.C001.PICI.TypeB.variant0003.Set2.prt	2973607	2980630	NZ_CP023624.1	MDRMA701
MYTU002.0321.00092.C001.PICI.TypeB.variant0004.Set1.prt	1780498	1788360	NZ_CP023624.1	MDRMA701
MYTU002.0321.00093.C001.PICI.TypeB.variant0003.Set1.prt	2973736	2980759	NZ_CP023625.1	MDRMA863
MYTU002.0321.00093.C001.PICI.TypeB.variant0004.Set2.prt	1780599	1788461	NZ_CP023625.1	MDRMA863
MYTU002.0321.00094.C001.PICI.TypeB.variant0003.Set1.prt	2973518	2980541	NZ_CP023626.1	MDRMA1565
MYTU002.0321.00094.C001.PICI.TypeB.variant0004.Set2.prt	1780395	1788257	NZ_CP023626.1	MDRMA1565
MYTU002.0321.00095.C001.PICI.TypeB.variant0003.Set2.prt	2973522	2980545	NZ_CP023627.1	MDRMA2019
MYTU002.0321.00095.C001.PICI.TypeB.variant0004.Set1.prt	1780446	1788308	NZ_CP023627.1	MDRMA2019
MYTU002.0321.00096.C001.PICI.TypeB.variant0003.Set2.prt	2973762	2980785	NZ_CP023628.1	MDRMA2082
MYTU002.0321.00096.C001.PICI.TypeB.variant0004.Set1.prt	1780630	1788492	NZ_CP023628.1	MDRMA2082
MYTU002.0321.00097.C001.PICI.TypeB.variant0003.Set1.prt	2973628	2980651	NZ_CP023629.1	MDRMA2260
MYTU002.0321.00097.C001.PICI.TypeB.variant0004.Set2.prt	1780522	1788384	NZ_CP023629.1	MDRMA2260
MYTU002.0321.00098.C001.PICI.TypeB.variant0003.Set1.prt	2973711	2980734	NZ_CP023630.1	MDRMA2441
MYTU002.0321.00098.C001.PICI.TypeB.variant0004.Set2.prt	1780580	1788442	NZ_CP023630.1	MDRMA2441
MYTU002.0321.00099.C001.PICI.TypeB.variant0003.Set1.prt	2973579	2980602	NZ_CP023631.1	TBDM1506
MYTU002.0321.00099.C001.PICI.TypeB.variant0004.Set2.prt	1780506	1788368	NZ_CP023631.1	TBDM1506
MYTU002.0321.00100.C001.PICI.TypeB.variant0003.Set1.prt	2973629	2980652	NZ_CP023632.1	TBDM2189
MYTU002.0321.00100.C001.PICI.TypeB.variant0004.Set2.prt	1780488	1788350	NZ_CP023632.1	TBDM2189
MYTU002.0321.00101.C001.PICI.TypeB.variant0003.Set2.prt	2973586	2980609	NZ_CP023633.1	TBDM2444
MYTU002.0321.00101.C001.PICI.TypeB.variant0004.Set1.prt	1780496	1788358	NZ_CP023620.1	LN1856
MYTU002.0321.00102.C001.PICI.TypeB.variant0003.Set1.prt	2973660	2980683	NZ_CP023634.1	TBDM2487
MYTU002.0321.00102.C001.PICI.TypeB.variant0004.Set2.prt	1780494	1788356	NZ_CP023634.1	TBDM2487
MYTU002.0321.00103.C001.PICI.TypeB.variant0003.Set2.prt	2973635	2980658	NZ_CP023635.1	TBDM2489
MYTU002.0321.00103.C001.PICI.TypeB.variant0004.Set1.prt	1780552	1788414	NZ_CP023635.1	TBDM2489

MYTU002.0321.00104.C001.PICI.TypeB.variant0003.Set2.prt	2973758	2980781	NZ_CP023636.1	TBDM2699
MYTU002.0321.00104.C001.PICI.TypeB.variant0004.Set1.prt	1780630	1788492	NZ_CP023628.1	MDRMA2082
MYTU002.0321.00105.C001.PICI.TypeB.variant0003.Set1.prt	2973744	2980767	NZ_CP023637.1	TBDM2717
MYTU002.0321.00105.C001.PICI.TypeB.variant0004.Set2.prt	1780616	1788478	NZ_CP023637.1	TBDM2717
MYTU002.0321.00106.C001.PICI.TypeB.variant0003.Set2.prt	2973580	2980603	NZ_CP023638.1	TBV4766
MYTU002.0321.00106.C001.PICI.TypeB.variant0004.Set1.prt	1780491	1788353	NZ_CP023638.1	TBV4766
MYTU002.0321.00107.C001.PICI.TypeB.variant0003.Set2.prt	2973518	2980541	NZ_CP023626.1	MDRMA1565
MYTU002.0321.00107.C001.PICI.TypeB.variant0004.Set1.prt	1780406	1788268	NZ_CP023639.1	TBV4768
MYTU002.0321.00108.C001.PICI.TypeB.variant0003.Set1.prt	2973728	2980751	NZ_CP023640.1	TBV4952
MYTU002.0321.00108.C001.PICI.TypeB.variant0004.Set2.prt	1780593	1788455	NZ_CP023597.1	SLM088
MYTU002.0321.00109.C001.PICI.TypeB.variant0003.Set2.prt	2973798	2980821	NZ_CP018778.1	DK9897
MYTU002.0321.00109.C001.PICI.TypeB.variant0004.Set1.prt	1780616	1788478	NZ_CP023637.1	TBDM2717
MYTU002.0321.00110.C001.PICI.TypeB.variant0003.Set2.prt	2973746	2980769	NZ_CP025594.1	GG-5-10
MYTU002.0321.00110.C001.PICI.TypeB.variant0004.Set1.prt	1780579	1788441	NZ_CP025594.1	GG-5-10
MYTU002.0321.00111.C001.PICI.TypeB.variant0003.Set1.prt	2973782	2980805	NZ_CP025595.1	GG-20-11
MYTU002.0321.00111.C001.PICI.TypeB.variant0004.Set2.prt	1780618	1788480	NZ_CP023602.1	LE13
MYTU002.0321.00112.C001.PICI.TypeB.variant0003.Set1.prt	2973775	2980798	NZ_CP025600.1	GG-77-11
MYTU002.0321.00112.C001.PICI.TypeB.variant0004.Set2.prt	1780585	1788447	NZ_CP025600.1	GG-77-11
MYTU002.0321.00113.C001.PICI.TypeB.variant0003.Set2.prt	2973819	2980850	NZ_CP025601.1	GG-90-10
MYTU002.0321.00113.C001.PICI.TypeB.variant0004.Set1.prt	1780636	1788498	NZ_CP025601.1	GG-90-10
MYTU002.0321.00114.C001.PICI.TypeB.variant0003.Set2.prt	2973693	2980716	NZ_CP025604.1	GG-129-11
MYTU002.0321.00114.C001.PICI.TypeB.variant0004.Set1.prt	1780528	1788390	NZ_CP025604.1	GG-129-11
MYTU002.0321.00115.C001.PICI.TypeB.variant0003.Set1.prt	2973741	2980764	NZ_CP025605.1	GG-134-11
MYTU002.0321.00115.C001.PICI.TypeB.variant0004.Set2.prt	1780576	1788438	NZ_CP025605.1	GG-134-11
MYTU002.0321.00116.C001.PICI.TypeB.variant0003.Set2.prt	2973773	2980799	NZ_CP025608.1	GG-229-10
MYTU002.0321.00116.C001.PICI.TypeB.variant0004.Set1.prt	1780607	1788469	NZ_CP025608.1	GG-229-10
MYTU002.0321.00117.C001.PICI.TypeB.variant0003.Set1.prt	2973792	2980823	NZ_CP025598.1	GG-37-11
MYTU002.0321.00117.C001.PICI.TypeB.variant0004.Set2.prt	1780628	1788490	NZ_CP025598.1	GG-37-11
MYTU002.0321.00118.C001.PICI.TypeB.variant0003.Set1.prt	2973770	2980793	NZ_CP025603.1	GG-121-10
MYTU002.0321.00118.C001.PICI.TypeB.variant0004.Set2.prt	1780608	1788470	NZ_CP025603.1	GG-121-10
MYTU002.0321.00119.C001.PICI.TypeB.variant0003.Set1.prt	2973786	2980809	NZ_CP025607.1	GG-186-10
MYTU002.0321.00119.C001.PICI.TypeB.variant0004.Set2.prt	1780619	1788481	NZ_CP025607.1	GG-186-10
MYTU002.0321.00120.C001.PICI.TypeB.variant0003.Set2.prt	2973700	2980723	NZ_CP023596.1	SLM063
MYTU002.0321.00120.C001.PICI.TypeB.variant0004.Set1.prt	1780548	1788410	NZ_CP025596.1	GG-27-11
MYTU002.0321.00121.C001.PICI.TypeB.variant0003.Set2.prt	2973765	2980788	NZ_CP025597.1	GG-36-11
MYTU002.0321.00121.C001.PICI.TypeB.variant0004.Set1.prt	1780600	1788462	NZ_CP025597.1	GG-36-11

MYTU002.0321.00122.C001.PICI.TypeB.variant0003.Set2.prt	2973766	2980789	NZ_CP025599.1	GG-45-11	
MYTU002.0321.00122.C001.PICI.TypeB.variant0004.Set1.prt	1780593	1788455	NZ_CP023597.1	SLM088	
MYTU002.0321.00123.C001.PICI.TypeB.variant0003.Set2.prt	2973734	2980757	NZ_CP025602.1	GG-109-10	
MYTU002.0321.00123.C001.PICI.TypeB.variant0004.Set1.prt	1780553	1788415	NZ_CP025602.1	GG-109-10	
MYTU002.0321.00124.C001.PICI.TypeB.variant0003.Set2.prt	2973766	2980789	NZ_CP025599.1	GG-45-11	
MYTU002.0321.00124.C001.PICI.TypeB.variant0004.Set1.prt	1780597	1788459	NZ_CP023619.1	LN1100	
MYTU002.0321.00125.C001.PICI.TypeB.variant0003.Set1.prt	572292	579315	NZ_CP022578.1	WC059	
MYTU002.0321.00126.C001.PICI.TypeB.variant0003.Set1.prt	716767	723790	NZ_CP022577.1	WC078	
MYTU002.0321.00127.C001.PICI.TypeB.variant0003.Set1.prt	2746895	2753918	NZ_CP030093.1	RUS_B0	
MYTU002.0321.00128.C001.PICI.TypeB.variant0003.Set1.prt	3985130	3992153	NZ_CP019610.1	H54	
MYTU002.0321.00129.C001.PICI.TypeB.variant0003.Set2.prt	3752724	3759747	NZ_CP019611.1	H83	
MYTU002.0321.00129.C001.PICI.TypeB.variant0004.Set1.prt	247387	255249	NZ_CP019611.1	H83	
MYTU002.0321.00130.C001.PICI.TypeB.variant0003.Set1.prt	2839160	2846183	NZ_CP019612.1	H107	
MYTU002.0321.00131.C001.PICI.TypeB.variant0003.Set1.prt	449851	456874	NZ_CP019613.1	H112	
MYTU002.0321.00132.C001.PICI.TypeB.variant0003.Set1.prt	2948027	2955050	NZ_CP029065.1	TBMENG-03	
MYTU002.0321.00132.C001.PICI.TypeB.variant0004.Set2.prt	1760314	1768176	NZ_CP029065.1	TBMENG-03	
MYTU002.0321.00133.C001.PICI.TypeB.variant0003.Set1.prt	2980514	2987914	NZ_CP041207.1	MT-0080	
MYTU002.0321.00133.C001.PICI.TypeB.variant0004.Set2.prt	3889577	3897439	NZ_CP041207.1	MT-0080	
MYTU002.0321.00134.C001.PICI.TypeB.variant0004.Set1.prt	2227764	2235626	NZ_CP044345.1	L	
MYTU002.0321.00136.C001.PICI.TypeB.variant0003.Set1.prt	1647691	1654714	NZ_CP046308.1	FDAARGOS_751	
MYTU002.0321.00136.C001.PICI.TypeB.variant0004.Set2.prt	434334	442196	NZ_CP046308.1	FDAARGOS_751	
MYTU002.0321.00137.C001.PICI.TypeB.variant0003.Set1.prt	1061569	1068592	NZ_CP046309.1	FDAARGOS_750	
MYTU002.0321.00137.C001.PICI.TypeB.variant0004.Set2.prt	4299667	4307528	NZ_CP046309.1	FDAARGOS_750	
MYTU002.0321.00138.C001.PICI.TypeB.variant0003.Set1.prt	2968468	2975491	NZ_CP046728.2	TCDC11	
MYTU002.0321.00139.C001.PICI.TypeB.variant0003.Set1.prt	2969148	2976171	NZ_CP048071.1	RW-TB008	
MYTU002.0321.00140.C001.PICI.TypeB.variant0003.Set1.prt	2959991	2967014	NZ_CP053092.1		4860
MYTU002.0321.00141.C001.PICI.TypeB.variant0003.Set1.prt	3817962	3824985	NZ_CP054013.1	FDAARGOS_757	
MYTU002.0321.00141.C001.PICI.TypeB.variant0004.Set2.prt	2621385	2629247	NZ_CP054013.1	FDAARGOS_757	
MYTU002.0321.00142.C001.PICI.TypeB.variant0003.Set1.prt	254488	261512	NZ_CP054014.1	FDAARGOS_756	
MYTU002.0321.00143.C001.PICI.TypeB.variant0003.Set1.prt	1434247	1441270	NZ_CP043995.1	4-0041P6C4	
MYTU002.0321.00144.C001.PICI.TypeB.variant0003.Set1.prt	2973085	2980108	NZ_CP043996.1	1-0072P6C4	
MYTU002.0321.00145.C001.PICI.TypeB.variant0003.Set1.prt	2967259	2974282	NZ_CP043997.1	1-0066P6C4	
MYTU002.0321.00146.C001.PICI.TypeB.variant0003.Set1.prt	2975875	2982898	NZ_CP041788.1	SEA17020030P6C4	
MYTU002.0321.00147.C001.PICI.TypeB.variant0003.Set1.prt	2969783	2976806	NZ_CP041789.1	SEA17020028P6C4	
MYTU002.0321.00148.C001.PICI.TypeB.variant0004.Set1.prt	1788084	1795946	NZ_CP041790.1	SEA17020024P6C4	
MYTU002.0321.00149.C001.PICI.TypeB.variant0004.Set1.prt	1782129	1789991	NZ_CP041792.1	SEA15230P6C4	

MYTU002.0321.00150.C001.PICI.TypeB.variant0004.Set1.prt	1788091	1795953	NZ_CP041791.1	SEA17020023P6C4
MYTU002.0321.00151.C001.PICI.TypeB.variant0004.Set1.prt	1786195	1794057	NZ_CP041793.1	SEA15229P6C4
MYTU002.0321.00154.C001.PICI.TypeB.variant0003.Set1.prt	2982412	2989434	NZ_CP041796.1	SEA14318P6C4
MYTU002.0321.00155.C001.PICI.TypeB.variant0003.Set1.prt	2958598	2965621	NZ_CP041797.1	SEA14117P6C4
MYTU002.0321.00157.C001.PICI.TypeB.variant0003.Set1.prt	2746901	2753924	NZ_CP041799.1	SEA13020298P6C4
MYTU002.0321.00158.C001.PICI.TypeB.variant0003.Set1.prt	2993486	3000507	NZ_CP041800.1	SEA12334P6C4
MYTU002.0321.00158.C001.PICI.TypeB.variant0004.Set2.prt	1788503	1796365	NZ_CP041800.1	SEA12334P6C4
MYTU002.0321.00159.C001.PICI.TypeB.variant0003.Set2.prt	2996873	3003896	NZ_CP041801.1	SEA12202P6C4
MYTU002.0321.00159.C001.PICI.TypeB.variant0004.Set1.prt	1790004	1797866	NZ_CP041801.1	SEA12202P6C4
MYTU002.0321.00162.C001.PICI.TypeB.variant0003.Set2.prt	2973847	2980870	NZ_CP041804.1	SEA11020092P6C4
MYTU002.0321.00162.C001.PICI.TypeB.variant0004.Set1.prt	1781103	1788965	NZ_CP041804.1	SEA11020092P6C4
MYTU002.0321.00163.C001.PICI.TypeB.variant0003.Set2.prt	2970215	2977237	NZ_CP041805.1	SEA11020068P6C4
MYTU002.0321.00163.C001.PICI.TypeB.variant0004.Set1.prt	3865748	3873610	NZ_CP041805.1	SEA11020068P6C4
MYTU002.0321.00164.C001.PICI.TypeB.variant0003.Set1.prt	2959275	2966298	NZ_CP041806.1	SEA11020057P6C4
MYTU002.0321.00164.C001.PICI.TypeB.variant0004.Set2.prt	3867552	3875414	NZ_CP041806.1	SEA11020057P6C4
MYTU002.0321.00165.C001.PICI.TypeB.variant0004.Set1.prt	1784038	1791900	NZ_CP041807.1	SEA11020038P6C4
MYTU002.0321.00166.C001.PICI.TypeB.variant0003.Set1.prt	2958478	2965501	NZ_CP041808.1	SEA09167P6C4
MYTU002.0321.00167.C001.PICI.TypeB.variant0003.Set1.prt	2960646	2967669	NZ_CP041809.1	SEA09020048P6C4
MYTU002.0321.00168.C001.PICI.TypeB.variant0003.Set1.prt	2969876	2976899	NZ_CP041810.1	SEA08162P6C4
MYTU002.0321.00169.C001.PICI.TypeB.variant0003.Set1.prt	2976538	2983561	NZ_CP041811.1	SEA08151P6C4
MYTU002.0321.00170.C001.PICI.TypeB.variant0003.Set1.prt	2974102	2981125	NZ_CP041812.1	SEA07020250P6C4
MYTU002.0321.00171.C001.PICI.TypeB.variant0003.Set1.prt	2995696	3002719	NZ_CP041813.1	SEA07010354P6C4
MYTU002.0321.00171.C001.PICI.TypeB.variant0004.Set2.prt	1789357	1797218	NZ_CP041813.1	SEA07010354P6C4
MYTU002.0321.00172.C001.PICI.TypeB.variant0003.Set1.prt	2976377	2983400	NZ_CP041814.1	SEA06535P6C4
MYTU002.0321.00173.C001.PICI.TypeB.variant0003.Set1.prt	2746907	2753930	NZ_CP041815.1	SEA02010036P6C4
MYTU002.0321.00174.C001.PICI.TypeB.variant0003.Set1.prt	2979800	2986823	NZ_CP041816.1	SEA00042P6C4
MYTU002.0321.00174.C001.PICI.TypeB.variant0004.Set2.prt	3888334	3896085	NZ_CP041816.1	SEA00042P6C4
MYTU002.0321.00175.C001.PICI.TypeB.variant0003.Set1.prt	2966986	2974009	NZ_CP017596.1	Beijing/391
MYTU002.0321.00176.C001.PICI.TypeB.variant0003.Set1.prt	2979215	2986238	NZ_CP041818.1	4-0087P6C4
MYTU002.0321.00176.C001.PICI.TypeB.variant0004.Set2.prt	3891652	3899514	NZ_CP041818.1	4-0087P6C4
MYTU002.0321.00177.C001.PICI.TypeB.variant0003.Set1.prt	2961825	2968848	NZ_CP041819.1	4-0077P6C4
MYTU002.0321.00178.C001.PICI.TypeB.variant0003.Set1.prt	2982838	2989860	NZ_CP041820.1	4-0073P6C4
MYTU002.0321.00179.C001.PICI.TypeB.variant0003.Set1.prt	2960215	2967238	NZ_CP041821.1	4-0062P6C4
MYTU002.0321.00180.C001.PICI.TypeB.variant0003.Set1.prt	1434197	1441220	NZ_CP041822.1	4-0024P6C4
MYTU002.0321.00181.C001.PICI.TypeB.variant0003.Set1.prt	1434253	1441276	NZ_CP041823.1	4-0019P6C4
MYTU002.0321.00182.C001.PICI.TypeB.variant0003.Set1.prt	2970589	2977612	NZ_CP041824.1	4-0012P6C4

MYTU002.0321.00183.C001.PICI.TypeB.variant0003.Set1.prt	1434253	1441276	NZ_CP041823.1	4-0019P6C4
MYTU002.0321.00184.C001.PICI.TypeB.variant0003.Set1.prt	3005937	3012960	NZ_CP041826.1	3-0124P6C4
MYTU002.0321.00184.C001.PICI.TypeB.variant0004.Set2.prt	1788679	1796541	NZ_CP041826.1	3-0124P6C4
MYTU002.0321.00185.C001.PICI.TypeB.variant0003.Set1.prt	2999022	3006045	NZ_CP041827.1	3-0096P6C4
MYTU002.0321.00185.C001.PICI.TypeB.variant0004.Set2.prt	1787995	1795857	NZ_CP041827.1	3-0096P6C4
MYTU002.0321.00186.C001.PICI.TypeB.variant0003.Set2.prt	2987705	2994728	NZ_CP041828.1	3-0090P6C4
MYTU002.0321.00186.C001.PICI.TypeB.variant0004.Set1.prt	1788644	1796506	NZ_CP041828.1	3-0090P6C4
MYTU002.0321.00187.C001.PICI.TypeB.variant0003.Set1.prt	2963173	2970196	NZ_CP041829.1	3-0059P6C4
MYTU002.0321.00188.C001.PICI.TypeB.variant0003.Set1.prt	2970846	2977869	NZ_CP041830.1	2-0068P6C4
MYTU002.0321.00189.C001.PICI.TypeB.variant0003.Set1.prt	2984692	2991714	NZ_CP041831.1	2-0059P6C4
MYTU002.0321.00190.C001.PICI.TypeB.variant0003.Set1.prt	2962892	2969915	NZ_CP041832.1	2-0052P6C4
MYTU002.0321.00191.C001.PICI.TypeB.variant0003.Set1.prt	2985791	2992814	NZ_CP041833.1	2-0046P6C4
MYTU002.0321.00191.C001.PICI.TypeB.variant0004.Set2.prt	3903803	3911665	NZ_CP041833.1	2-0046P6C4
MYTU002.0321.00192.C001.PICI.TypeB.variant0003.Set2.prt	2970135	2977158	NZ_CP041834.1	2-0043-unknownP6C4
MYTU002.0321.00192.C001.PICI.TypeB.variant0004.Set1.prt	3881287	3889149	NZ_CP041834.1	2-0043-unknownP6C4
MYTU002.0321.00193.C001.PICI.TypeB.variant0003.Set1.prt	2963014	2970038	NZ_CP041835.1	2-0034P6C4
MYTU002.0321.00194.C001.PICI.TypeB.variant0003.Set1.prt	2746883	2753906	NZ_CP041836.1	2-0031P6C4
MYTU002.0321.00195.C001.PICI.TypeB.variant0003.Set1.prt	2985919	2992942	NZ_CP041837.1	2-0029P6C4
MYTU002.0321.00195.C001.PICI.TypeB.variant0004.Set2.prt	3903937	3911799	NZ_CP041837.1	2-0029P6C4
MYTU002.0321.00196.C001.PICI.TypeB.variant0003.Set1.prt	2985834	2992857	NZ_CP041838.1	2-0028P6C4
MYTU002.0321.00196.C001.PICI.TypeB.variant0004.Set2.prt	3903855	3911717	NZ_CP041838.1	2-0028P6C4
MYTU002.0321.00197.C001.PICI.TypeB.variant0003.Set1.prt	2985921	2992944	NZ_CP041839.1	2-0023P6C4
MYTU002.0321.00197.C001.PICI.TypeB.variant0004.Set2.prt	3903941	3911803	NZ_CP041839.1	2-0023P6C4
MYTU002.0321.00198.C001.PICI.TypeB.variant0003.Set2.prt	2985905	2992928	NZ_CP041840.1	2-0022P6C4
MYTU002.0321.00198.C001.PICI.TypeB.variant0004.Set1.prt	3903919	3911781	NZ_CP041840.1	2-0022P6C4
MYTU002.0321.00199.C001.PICI.TypeB.variant0003.Set1.prt	2985842	2992865	NZ_CP041841.1	2-0021P6C4
MYTU002.0321.00199.C001.PICI.TypeB.variant0004.Set2.prt	3903864	3911726	NZ_CP041841.1	2-0021P6C4
MYTU002.0321.00200.C001.PICI.TypeB.variant0003.Set1.prt	2969337	2976359	NZ_CP041842.1	2-0013P6C4
MYTU002.0321.00201.C001.PICI.TypeB.variant0003.Set1.prt	2971004	2978027	NZ_CP041843.1	1-0168P6C4
MYTU002.0321.00202.C001.PICI.TypeB.variant0003.Set2.prt	2989272	2996293	NZ_CP041844.1	1-0160P6C4
MYTU002.0321.00202.C001.PICI.TypeB.variant0004.Set1.prt	1787581	1795442	NZ_CP041844.1	1-0160P6C4
MYTU002.0321.00203.C001.PICI.TypeB.variant0003.Set2.prt	2982373	2989396	NZ_CP041845.1	1-0156P6C4
MYTU002.0321.00203.C001.PICI.TypeB.variant0004.Set1.prt	1785246	1793108	NZ_CP041845.1	1-0156P6C4
MYTU002.0321.00204.C001.PICI.TypeB.variant0003.Set1.prt	2968382	2975405	NZ_CP041846.1	1-0153P6C4
MYTU002.0321.00205.C001.PICI.TypeB.variant0003.Set1.prt	2970092	2977115	NZ_CP041847.1	1-0149P6C4
MYTU002.0321.00206.C001.PICI.TypeB.variant0003.Set1.prt	2968617	2975640	NZ_CP041848.1	1-0137P6C4

MYTU002.0321.00207.C001.PICI.TypeB.variant0003.Set1.prt	2972852	2979875	NZ_CP041849.1	1-0123P6C4	
MYTU002.0321.00208.C001.PICI.TypeB.variant0003.Set1.prt	2967074	2974097	NZ_CP041850.1	1-0116P6C4	
MYTU002.0321.00209.C001.PICI.TypeB.variant0003.Set1.prt	2971650	2978671	NZ_CP041851.1	1-0112P6C4	
MYTU002.0321.00210.C001.PICI.TypeB.variant0003.Set1.prt	2970505	2977526	NZ_CP041852.1	1-0110P6C4	
MYTU002.0321.00211.C001.PICI.TypeB.variant0003.Set1.prt	3000609	3007632	NZ_CP041853.1	1-0107P6C4	
MYTU002.0321.00211.C001.PICI.TypeB.variant0004.Set2.prt	1790050	1797911	NZ_CP041853.1	1-0107P6C4	
MYTU002.0321.00212.C001.PICI.TypeB.variant0003.Set1.prt	2971444	2978466	NZ_CP041854.1	1-0102P6C4	
MYTU002.0321.00213.C001.PICI.TypeB.variant0003.Set1.prt	2970098	2977121	NZ_CP041855.1	1-0084P6C4	
MYTU002.0321.00214.C001.PICI.TypeB.variant0003.Set1.prt	2966716	2973739	NZ_CP041856.1	1-0071P6C4	
MYTU002.0321.00215.C001.PICI.TypeB.variant0003.Set1.prt	2966712	2973735	NZ_CP041857.1	1-0069P6C4	
MYTU002.0321.00216.C001.PICI.TypeB.variant0003.Set1.prt	2969447	2976471	NZ_CP041858.1	1-0064P6C4	
MYTU002.0321.00217.C001.PICI.TypeB.variant0004.Set1.prt	1782882	1790744	NZ_CP041859.1	1-0061P6C4	
MYTU002.0321.00218.C001.PICI.TypeB.variant0003.Set1.prt	2971841	2978864	NZ_CP041860.1	1-0056P6C4	
MYTU002.0321.00219.C001.PICI.TypeB.variant0003.Set1.prt	2969986	2977009	NZ_CP041861.1	1-0054P6C4	
MYTU002.0321.00220.C001.PICI.TypeB.variant0003.Set1.prt	2989884	2996907	NZ_CP041862.1	1-0047P6C4	
MYTU002.0321.00220.C001.PICI.TypeB.variant0004.Set2.prt	1782754	1790615	NZ_CP041862.1	1-0047P6C4	
MYTU002.0321.00221.C001.PICI.TypeB.variant0003.Set1.prt	2967252	2974275	NZ_CP041863.1	1-0045P6C4	
MYTU002.0321.00222.C001.PICI.TypeB.variant0003.Set1.prt	2953939	2960962	NZ_CP041864.1	1-0044P6C4	
MYTU002.0321.00223.C001.PICI.TypeB.variant0003.Set1.prt	2973014	2980035	NZ_CP041865.1	1-0039P6C4	
MYTU002.0321.00224.C001.PICI.TypeB.variant0003.Set1.prt	2971020	2978043	NZ_CP041866.1	1-0036P6C4	
MYTU002.0321.00225.C001.PICI.TypeB.variant0003.Set1.prt	2970093	2977116	NZ_CP041867.1	1-0031P6C4	
MYTU002.0321.00226.C001.PICI.TypeB.variant0004.Set1.prt	105212	113074	NZ_CP041868.1	1-0030P6C4	
MYTU002.0321.00227.C001.PICI.TypeB.variant0003.Set1.prt	2967260	2974283	NZ_CP041869.1	1-0028P6C4	
MYTU002.0321.00228.C001.PICI.TypeB.variant0003.Set1.prt	2972650	2979673	NZ_CP041870.1	1-0023P6C4	
MYTU002.0321.00229.C001.PICI.TypeB.variant0003.Set2.prt	2984011	2991033	NZ_CP041871.1	1-0021P6C4	
MYTU002.0321.00229.C001.PICI.TypeB.variant0004.Set1.prt	1784848	1792709	NZ_CP041871.1	1-0021P6C4	
MYTU002.0321.00230.C001.PICI.TypeB.variant0003.Set1.prt	2970097	2977120	NZ_CP041872.1	1-0017P6C4	
MYTU002.0321.00231.C001.PICI.TypeB.variant0003.Set2.prt	2977921	2984944	NZ_CP041873.1	1-0013P6C4	
MYTU002.0321.00231.C001.PICI.TypeB.variant0004.Set1.prt	1789273	1797134	NZ_CP041873.1	1-0013P6C4	
MYTU002.0321.00232.C001.PICI.TypeB.variant0003.Set1.prt	2970090	2977113	NZ_CP041874.1	1-0009P6C4	
MYTU002.0321.00233.C001.PICI.TypeB.variant0003.Set1.prt	2994700	3001723	NZ_CP041875.1	1-0007P6C4	
MYTU002.0321.00233.C001.PICI.TypeB.variant0004.Set2.prt	1789086	1796947	NZ_CP041875.1	1-0007P6C4	
MYTU002.0321.00234.C001.PICI.TypeB.variant0003.Set1.prt	2972581	2979604	NZ_CP041876.1	1-0006P6C4	
MYTU002.0321.00235.C001.PICI.TypeB.variant0003.Set1.prt	2749560	2756583	NZ_CP070338.1		11502
MYTU002.0321.00236.C001.PICI.TypeB.variant0003.Set1.prt	2973787	2980810	NZ_CP071127.1	120/26CAO	
MYTU002.0321.00236.C001.PICI.TypeB.variant0004.Set2.prt	1780639	1788501	NZ_CP009101.1	ZMC13-88	

MYTU002.0321.00237.C001.PICI.TypeB.variant0003.Set2.prt	2973791	2980814	NZ_CP071128.1	267/47W148
MYTU002.0321.00237.C001.PICI.TypeB.variant0004.Set1.prt	1780640	1788502	NZ_CP071128.1	267/47W148
MYTU002.0321.00238.C001.PICI.TypeB.variant0003.Set2.prt	2980436	2987459	NZ_LR027516.1	DKC2
MYTU002.0321.00238.C001.PICI.TypeB.variant0004.Set1.prt	105183	113045	NZ_LR027516.1	DKC2
MYTU002.0321.00246.C001.PICI.TypeB.variant0003.Set2.prt	2968581	2975604	NC_002755.2	CDC1551
MYTU002.0321.00247.C001.PICI.TypeB.variant0003.Set1.prt	2970551	2979309	NC_000962.3	H37Rv
MYTU002.0321.00247.C001.PICI.TypeB.variant0004.Set2.prt	1780643	1788505	NC_000962.3	H37Rv
MYTU002.0321.00248.C001.PICI.TypeB.variant0003.Set1.prt	2973805	2980828	NC_018143.2	H37Rv
MYTU002.0321.00248.C001.PICI.TypeB.variant0004.Set2.prt	1780650	1788512	NC_018143.2	H37Rv
MYTU002.0321.00249.C001.PICI.TypeB.variant0003.Set1.prt	2985754	2992777	NC_009565.1	F11
MYTU002.0321.00250.C001.PICI.TypeB.variant0003.Set1.prt	2967697	2974720	NC_022350.1	Haarlem
MYTU002.0321.00250.C001.PICI.TypeB.variant0004.Set2.prt	3873621	3881483	NC_022350.1	Haarlem
MYTU002.0321.00253.C001.PICI.TypeB.variant0003.Set1.prt	2985763	2992786	NC_009525.1	H37Ra; ATCC 25177
MYTU002.0321.00253.C001.PICI.TypeB.variant0004.Set2.prt	1782161	1790023	NC_009525.1	H37Ra; ATCC 25177
MYTU002.0321.00254.C001.PICI.TypeB.variant0003.Set1.prt	2986959	2993982	NZ_CP016972.1	H37Ra
MYTU002.0321.00254.C001.PICI.TypeB.variant0004.Set2.prt	1782215	1790077	NZ_CP016972.1	H37Ra
MYTU002.0321.00255.C001.PICI.TypeB.variant0003.Set1.prt	2966971	2973994	NC_021251.1	CCDC5079
MYTU002.0321.00256.C001.PICI.TypeB.variant0003.Set1.prt	2960641	2967664	NC_017522.1	CCDC5180
MYTU002.0321.00257.C001.PICI.TypeB.variant0003.Set1.prt	2965216	2972239	NZ_CP002885.1	CCDC5180
MYTU002.0321.00258.C001.PICI.TypeB.variant0003.Set1.prt	1434299	1441322	NC_016768.1	KZN 4207
MYTU002.0321.00259.C001.PICI.TypeB.variant0003.Set1.prt	1434355	1441378	NC_012943.1	KZN MDR
MYTU002.0321.00260.C001.PICI.TypeB.variant0003.Set1.prt	1434467	1441490	NC_018078.1	KZN XDR
MYTU002.0321.00264.C001.PICI.TypeB.variant0003.Set1.prt	2960678	2967701	NC_020559.1	Erdman
MYTU002.0321.00264.C001.PICI.TypeB.variant0004.Set2.prt	3869086	3876948	NC_020559.1	Erdman
MYTU002.0321.00265.C001.PICI.TypeB.variant0003.Set1.prt	2746860	2753883	NZ_CP012090.1	W-148
MYTU002.0321.00266.C001.PICI.TypeB.variant0003.Set1.prt	2967339	2974362	NC_017524.1	CTRI-2
MYTU002.0321.00268.C001.PICI.TypeB.variant0003.Set1.prt	2964404	2971427	NZ_CP002871.1	HKBS1
MYTU002.0321.00269.C001.PICI.TypeB.variant0003.Set1.prt	2954634	2961657	NZ_CP002882.1	BT2
MYTU002.0321.00270.C001.PICI.TypeB.variant0003.Set1.prt	2962031	2969054	NZ_CP002883.1	BT1
MYTU002.0321.00271.C001.PICI.TypeB.variant0003.Set2.prt	2973033	2980056	NC_020089.1	7199-99
MYTU002.0321.00271.C001.PICI.TypeB.variant0004.Set1.prt	3884724	3892586	NC_020089.1	7199-99
MYTU002.0321.00273.C001.PICI.TypeB.variant0003.Set1.prt	2955050	2962073	NZ_CP007803.1	K
MYTU002.0321.00274.C001.PICI.TypeB.variant0003.Set2.prt	2960225	2967248	NC_021740.1	EAI5
MYTU002.0321.00274.C001.PICI.TypeB.variant0004.Set1.prt	1777941	1785803	NC_021740.1	EAI5
MYTU002.0321.00276.C001.PICI.TypeB.variant0003.Set1.prt	2967867	2974891	NZ_HG813240.1	49-02
MYTU002.0321.00277.C001.PICI.TypeB.variant0003.Set2.prt	2973183	2980206	NZ_CP007027.1	H37RvSiena

MYTU002.0321.00277.C001.PICI.TypeB.variant0004.Set1.prt	1780648	1788510	NZ_CP007027.1	H37RvSiena	
MYTU002.0321.00278.C001.PICI.TypeB.variant0003.Set1.prt	2978194	2985217	NZ_AP014573.1	Kurono	
MYTU002.0321.00278.C001.PICI.TypeB.variant0004.Set2.prt	1786006	1793868	NZ_AP014573.1	Kurono	
MYVA001.0321.00001.C001.PICI.TypeB.variant0001.Set1.prt	2655123	2661941	NC_008726.1	PYR-1	
NIHA002.0321.00001.C001.PICI.TypeB.variant0001.SetR1.prt	2389893	2397440	NC_007964.1	X14	
NIHA002.0321.00001.C001.PICI.TypeB.variant0002.Set1.prt	3683400	3702617	NC_007964.1	X14	
NIJA001.0321.00001.C001.PICI.TypeB.variant0002.Set1.prt	1166404	1177454	NZ_LT828648.1	unknown	
NISP005.0321.00001.C001.PICI.TypeB.variant0002.Set1.prt	1580924	1586799	NZ_CP042301.2	SY7	
NIWA001.0321.00001.C001.PICI.TypeB.variant0002.Set1.prt	375757	381461	NZ_CP038033.1	D1FHS	
NOFA001.0321.00003.C001.PICI.TypeB.variant0001.Set1.prt	248379	258200	NC_006361.1	IFM 10152	
NOOT001.0321.00001.C001.PICI.TypeB.variant0001.Set1.prt	6761433	6769679	NZ_CP041695.1	NEB252	
PAAT001.0321.00001.C001.PICI.TypeB.variant0001.Set1.prt	3318022	3324202	NC_014117.1	CCGE1002	
PACA004.0321.00004.C001.PICI.TypeB.variant0002.Set3.prt	3270217	3277284	NZ_CP015958.1		852011
PAKO001.0321.00001.C001.PICI.TypeB.variant0004.SetR1.prt	1628585	1647010	NZ_CP045072.1	BJQ0001	
PAKO001.0321.00001.C002.PICI.TypeB.variant0004.SetR1.prt	684256	701965	NZ_CP045073.1	BJQ0001	
PAME002.0321.00001.C001.PICI.TypeB.variant0001.Set1.prt	1652019	1667427	NZ_CP041745.1	LMG 23650	
PAYO001.0321.00001.C001.PICI.TypeB.variant0001.Set1.prt	3358940	3374960	NZ_CP014167.1	DCY84	
PEDA001.0321.00002.P007.PICI.TypeB.variant0002.Set1.prt	2828	9389	NZ_CP012277.1	TMW 2.1533	
PEDA001.0321.00004.P005.PICI.TypeB.variant0002.Set1.prt	4361	10922	NZ_CP012289.1	TMW 2.1535	
PEPA002.0321.00001.C001.PICI.TypeB.variant0002.Set1.prt	2971866	2978462	NZ_CP065915.1	SM1903	
POSP004.0321.00001.C001.PICI.TypeB.variant0001.Set1.prt	1793155	1798752	NZ_CP017113.1	LM 6	
POSP014.0321.00001.C001.PICI.TypeB.variant0001.Set1.prt	3126147	3132812	NC_007948.1	JS666	
PRFR001.0321.00018.C001.PICI.TypeB.variant0001.Set1.prt	501875	509977	NC_014215.1	CIRM-B1AI	
PSSP115.0321.00001.C001.PICI.TypeB.variant0001.Set1.prt	1061408	1069283	NZ_CP045553.1		13159349
PSSY002.0321.00013.C001.PICI.TypeB.variant0001.Set1.prt	6116675	6121746	NZ_CP024712.1	MAFF212063	
RHAE001.0321.00002.C001.PICI.TypeB.variant0004.Set1.prt	4251373	4258293	NZ_CP069306.1	unknown	
RHBA002.0321.00001.C001.PICI.TypeB.variant0001.Set2.prt	2256738	2265136	NZ_CP024899.1	alga05	
RHER001.0321.00002.C001.PICI.TypeB.variant0001.Set1.prt	4131387	4137815	NZ_CP044284.1	X5	
RHER001.0321.00003.C001.PICI.TypeB.variant0001.Set2.prt	341350	349776	NZ_CP050124.1	KB1	
RHET001.0321.00004.C001.PICI.TypeB.variant0001.SetR1.prt	1392696	1418586	NZ_CP017241.1	8C-3	
RHPY001.0321.00003.C001.PICI.TypeA.Set3.prt	991429	999308	NZ_CP063450.1	5Ap	
RHPY001.0321.00004.C001.PICI.TypeB.variant0004.Set1.prt	377927	385140	NC_023150.1	SB3094	
RHSE001.0321.00001.C001.PICI.TypeB.variant0002.Set1.prt	4131213	4150883	NZ_CP035503.1	CHu59-6-5	
RHSP005.0321.00001.C001.PICI.TypeB.variant0001.Set1.prt	2614567	2621454	NZ_CP032762.1	P1Y	
RHSP010.0321.00001.C001.PICI.TypeB.variant0001.Set2.prt	1046627	1054705	NZ_CP018063.1	2G	
RHSP015.0321.00001.C001.PICI.TypeB.variant0001.Set1.prt	522367	528256	NZ_CP013511.1	N1314	

RHSP019.0321.00001.C001.PICI.TypeB.variant0001.Set1.prt	522367	528256	NZ_CP013511.1	N1314
RHSP023.0321.00001.C001.PICI.TypeB.variant0001.Set1.prt	6187139	6195586	NZ_CP012749.1	8
RHSP031.0321.00001.C001.PICI.TypeB.variant0004.Set1.prt	252368	259913	NZ_CP064065.1	M8
RHSP045.0321.00001.C001.PICI.TypeB.variant0001.Set1.prt	2091128	2097389	NZ_CP034766.1	X156
RHSP060.0321.00001.C001.PICI.TypeB.variant0003.Set1.prt	2446036	2452970	NZ_CP069535.1	FDAARGOS_1247
ROMU003.0321.00002.C001.PICI.TypeB.variant0001.Set1.prt	120348	140011	NZ_CP023510.1	FDAARGOS_369
SAEN001.0321.00096.C001.PICI.TypeB.variant0002.Set1.prt	632861	641532	NZ_CP030180.1	SA20100201
SASP016.0321.00001.C001.PICI.TypeB.variant0003.Set1.prt	2262539	2269281	NZ_CP061771.1	ZJ450
SEMA003.0321.00012.C001.PICI.TypeB.variant0001.Set1.prt	4404648	4413964	NZ_CP018917.1	UMH5
SHBO001.0321.00001.C001.PICI.TypeB.variant0001.Set2.prt	2231386	2239605	NZ_CP011511.1	ATCC 9210
SHBO001.0321.00003.C001.PICI.TypeB.variant0001.Set1.prt	562222	570826	NZ_CP026766.1	59-248
SHBO001.0321.00008.C001.PICI.TypeB.variant0001.Set1.prt	2832747	2840966	NZ_CP049285.1	602068
SHBO001.0321.00008.C001.PICI.TypeB.variant0001.Set2.prt	2526521	2535055	NZ_CP049285.1	602068
SHBO001.0321.00009.C001.PICI.TypeB.variant0001.Set1.prt	674991	683895	NZ_CP068090.1	FDAARGOS_1139
SHBO001.0321.00010.C001.PICI.TypeB.variant0001.Set2.prt	2100297	2108517	NC_007613.1	Sb227
SHDY001.0321.00002.C001.PICI.TypeB.variant0001.Set1.prt	148412	156629	unknown	unknown
SIME002.0321.00002.C001.PICI.TypeB.variant0001.SetR1.prt	211375	233745	NZ_CP021793.1	USDA1157
SIME002.0321.00003.C001.PICI.TypeB.variant0001.Set1.prt	2907180	2917062	NZ_CP021808.1	Rm41
SIME002.0321.00009.C001.PICI.TypeB.variant0001.SetR1.prt	2613841	2635217	CP021800.1	USDA1021
SIME002.0321.00015.C001.PICI.TypeB.variant0001.Set1.prt	1744341	1755310	NZ_CP065020.1	S35m
SIME002.0321.00018.C001.PICI.TypeB.variant0001.SetR1.prt	264479	286864	NC_015590.1	AK83
SIME002.0321.00021.C001.PICI.TypeB.variant0001.Set1.prt	89293	99175	NC_018700.1	Rm41
SIME002.0321.00024.C001.PICI.TypeB.variant0001.SetR1.prt	528893	551287	NZ_CP021219.1	RU11/001
SPRI001.0321.00001.C001.PICI.TypeB.variant0002.Set1.prt	1227367	1237221	NZ_CP020105.1	KCTC 12531
SPYA001.0321.00006.C001.PICI.TypeB.variant0001.SetR1.prt	5183342	5194493	CP060122.1	A3
STAG002.0321.00002.C001.PICI.TypeB.variant0002.Set1.prt	2274689	2284651	NZ_CP031266.1	12B
STAR001.0321.00001.C001.PICI.TypeB.variant0002.SetR1.prt	2038109	2047660	NZ_AP019698.1	P2
STAR002.0321.00002.C001.PICI.TypeB.variant0002.Set1.prt	2000403	2010819	NZ_CP025023.1	XNO106
STAR002.0321.00003.C001.PICI.TypeB.variant0002.Set1.prt	2000398	2010814	NZ_CP023076.1	XNO62
STAR002.0321.00005.C001.PICI.TypeB.variant0002.Set1.prt	1036639	1046721	NZ_CP042286.1	B3-25B
STAU002.0321.00001.C001.PICI.TypeB.variant0002.Set1.prt	925028	936984	NC_021670.1	Bmb9393
STAU002.0321.00003.C001.PICI.TypeB.variant0002.Set1.prt	1468348	1481027	NZ_CP007539.2	FDAARGOS_5
STAU002.0321.00006.C001.PICI.TypeB.variant0002.Set1.prt	908471	921150	NZ_CP007499.1	2395 USA500
STAU002.0321.00007.C001.PICI.TypeB.variant0002.Set1.prt	360983	371823	NZ_CP009361.1	ATCC 25923
STAU002.0321.00007.C001.PICI.TypeB.variant0002.Set2.prt	2043481	2054340	NZ_CP009361.1	ATCC 25923
STAU002.0321.00009.C001.PICI.TypeB.variant0002.Set1.prt	357246	368108	NZ_CP009554.1	FORC_001

STAU002.0321.00016.C001.PICI.TypeB.variant0002.Set1.prt	426620	437166	NZ_LN626917.1	ILRI_Eymole1/1
STAU002.0321.00024.C001.PICI.TypeB.variant0002.Set1.prt	2529166	2540468	NZ_CP026960.1	FDAARGOS_15
STAU002.0321.00028.C001.PICI.TypeB.variant0002.Set1.prt	118393	129186	NZ_CP026071.1	FDAARGOS_30
STAU002.0321.00033.C001.PICI.TypeB.variant0002.Set1.prt	1615947	1626284	NZ_CP026079.1	FDAARGOS_9
STAU002.0321.00036.C001.PICI.TypeB.variant0002.Set1.prt	1403340	1413490	NZ_CP026069.1	FDAARGOS_25
STAU002.0321.00036.C001.PICI.TypeB.variant0002.Set3.prt	2673216	2683553	NZ_CP026069.1	FDAARGOS_25
STAU002.0321.00037.C001.PICI.TypeB.variant0002.Set2.prt	727575	740254	NZ_CP026072.1	FDAARGOS_35
STAU002.0321.00038.C001.PICI.TypeB.variant0002.Set1.prt	1934419	1944756	NZ_CP026958.1	FDAARGOS_40
STAU002.0321.00038.C001.PICI.TypeB.variant0002.Set3.prt	325978	336128	NZ_CP026958.1	FDAARGOS_40
STAU002.0321.00040.C001.PICI.TypeB.variant0002.Set1.prt	1743786	1754556	NZ_CP026074.1	FDAARGOS_47
STAU002.0321.00041.C001.PICI.TypeB.variant0002.Set2.prt	410613	421169	NZ_CP007670.1	M121
STAU002.0321.00050.C001.PICI.TypeB.variant0002.Set1.prt	401962	412834	NZ_CP012593.1	HOU1444-VR
STAU002.0321.00050.C001.PICI.TypeB.variant0002.Set3.prt	2060396	2070804	NZ_CP012593.1	HOU1444-VR
STAU002.0321.00051.C001.PICI.TypeB.variant0002.Set1.prt	799240	809408	NZ_CP010890.1	SA564
STAU002.0321.00054.C001.PICI.TypeB.variant0002.Set1.prt	555447	569192	NZ_CP012756.1	JS395
STAU002.0321.00054.C001.PICI.TypeB.variant0002.Set2.prt	2232487	2242534	NZ_CP012756.1	JS395
STAU002.0321.00055.C001.PICI.TypeB.variant0002.Set1.prt	2657301	2667822	NZ_CP013137.1	XQ
STAU002.0321.00056.C001.PICI.TypeB.variant0002.Set1.prt	870566	883241	NZ_CP009828.1	MS4
STAU002.0321.00061.C001.PICI.TypeB.variant0002.Set2.prt	925192	937146	NZ_CP012015.1	Gv51
STAU002.0321.00062.C001.PICI.TypeB.variant0002.Set2.prt	924520	936476	NZ_CP012013.1	Be62
STAU002.0321.00063.C001.PICI.TypeB.variant0002.Set1.prt	924786	936742	NZ_CP012018.1	Gv88
STAU002.0321.00065.C001.PICI.TypeB.variant0002.Set2.prt	926284	938240	NZ_CP012012.1	HC1335
STAU002.0321.00066.C001.PICI.TypeB.variant0002.Set1.prt	843233	855475	NZ_AP014942.1	FDA209P
STAU002.0321.00081.C001.PICI.TypeB.variant0002.Set2.prt	2113873	2124210	NZ_CP011685.1	ZJ5499
STAU002.0321.00081.C001.PICI.TypeB.variant0002.Set3.prt	843568	853718	NZ_CP011685.1	ZJ5499
STAU002.0321.00082.C001.PICI.TypeB.variant0002.Set1.prt	2208277	2218614	NZ_CP013953.1	NCCP14558
STAU002.0321.00082.C001.PICI.TypeB.variant0002.Set2.prt	876227	886377	NZ_CP013953.1	NCCP14558
STAU002.0321.00083.C001.PICI.TypeB.variant0002.Set1.prt	2168989	2179326	NZ_CP013955.1	NCCP14562
STAU002.0321.00083.C001.PICI.TypeB.variant0002.Set2.prt	883579	893729	NZ_CP013955.1	NCCP14562
STAU002.0321.00093.C001.PICI.TypeB.variant0002.Set1.prt	2163974	2174311	NZ_CP012692.1	FORC_027
STAU002.0321.00096.C001.PICI.TypeB.variant0002.Set1.prt	850977	863653	NZ_CP013182.1	SA40TW
STAU002.0321.00121.C001.PICI.TypeB.variant0002.Set1.prt	842716	854958	NZ_CP020020.1	ATCC 6538
STAU002.0321.00129.C001.PICI.TypeB.variant0002.Set2.prt	911815	923926	NZ_CP015447.2	M92
STAU002.0321.00131.C001.PICI.TypeB.variant0002.Set1.prt	360983	371823	NZ_CP009361.1	ATCC 25923
STAU002.0321.00131.C001.PICI.TypeB.variant0002.Set2.prt	2043487	2054346	NZ_CP021905.1	Seattle 1945
STAU002.0321.00132.C001.PICI.TypeB.variant0002.Set1.prt	360983	371823	NZ_CP009361.1	ATCC 25923

STAU002.0321.00132.C001.PICI.TypeB.variant0002.Set2.prt	2043487	2054346	NZ_CP021905.1	Seattle 1945	
STAU002.0321.00139.C001.PICI.TypeB.variant0002.Set2.prt	385407	396158	NZ_AP014921.1	JH4899	
STAU002.0321.00139.C001.PICI.TypeB.variant0002.Set3.prt	846038	856008	NZ_AP014921.1	JH4899	
STAU002.0321.00140.C001.PICI.TypeB.variant0002.Set1.prt	580228	592907	NZ_CP023500.1	FDAARGOS_412	
STAU002.0321.00141.C001.PICI.TypeB.variant0002.Set1.prt	888752	901427	NZ_CP020741.1	HZW450	
STAU002.0321.00143.C001.PICI.TypeB.variant0002.Set2.prt	2121487	2132280	NZ_CP017684.1	CFSAN007847	
STAU002.0321.00143.C001.PICI.TypeB.variant0002.Set3.prt	356715	367576	NZ_CP017684.1	CFSAN007847	
STAU002.0321.00144.C001.PICI.TypeB.variant0002.Set1.prt	360704	371577	NZ_CP017682.1	CFSAN007850	
STAU002.0321.00146.C001.PICI.TypeB.variant0002.Set1.prt	835583	848259	NZ_CP017679.1	CFSAN007883	
STAU002.0321.00147.C001.PICI.TypeB.variant0002.Set1.prt	794531	804612	NZ_CP017677.1	CFSAN007894	
STAU002.0321.00154.C001.PICI.TypeB.variant0002.Set1.prt	1578228	1590339	NZ_CP018629.1	MRSA107	
STAU002.0321.00155.C001.PICI.TypeB.variant0002.Set1.prt	2258428	2268699	NZ_CP020656.1	K5	
STAU002.0321.00156.C001.PICI.TypeB.variant0002.Set1.prt	1981427	1991993	NZ_CP020354.1	FORC59	
STAU002.0321.00159.C001.PICI.TypeB.variant0002.Set2.prt	791063	801288	NZ_CP028165.1	CFSAN064037	
STAU002.0321.00161.C001.PICI.TypeB.variant0002.Set2.prt	1162225	1173568	NZ_CP023561.1	TF3198	
STAU002.0321.00172.C001.PICI.TypeB.variant0002.Set1.prt	424266	435836	NZ_CP029166.1	SVH7513	
STAU002.0321.00172.C001.PICI.TypeB.variant0002.Set3.prt	935299	947978	NZ_CP029166.1	SVH7513	
STAU002.0321.00174.C001.PICI.TypeB.variant0002.Set2.prt	2011737	2022530	NZ_CP028468.1	IT1-S	
STAU002.0321.00175.C001.PICI.TypeB.variant0002.Set2.prt	2011737	2022530	NZ_CP028468.1	IT1-S	
STAU002.0321.00176.C001.PICI.TypeB.variant0002.Set1.prt	719873	730589	NZ_CP029629.1	MOK063	
STAU002.0321.00177.C001.PICI.TypeB.variant0002.Set1.prt	2116454	2127170	NZ_CP029627.1	MOK042	
STAU002.0321.00178.C001.PICI.TypeB.variant0002.Set1.prt	1365712	1375944	NZ_CP029649.1	AR_0472	
STAU002.0321.00180.C001.PICI.TypeB.variant0002.Set2.prt	2137777	2148112	NZ_CP029675.1	AR_0219	
STAU002.0321.00180.C001.PICI.TypeB.variant0002.Set3.prt	867939	878083	NZ_CP029675.1	AR_0219	
STAU002.0321.00184.C001.PICI.TypeB.variant0002.Set1.prt	1991782	2002014	NZ_CP029681.1	AR_0473	
STAU002.0321.00193.C001.PICI.TypeB.variant0002.Set1.prt	625048	635376	NZ_CP029671.1	AR_0222	
STAU002.0321.00195.C001.PICI.TypeB.variant0002.Set2.prt	1001190	1013301	NZ_CP029685.1	CMRSA-3	
STAU002.0321.00196.C001.PICI.TypeB.variant0002.Set2.prt	953998	966109	NZ_CP027788.1	CMRSA-6	
STAU002.0321.00197.C001.PICI.TypeB.variant0002.Set1.prt	2413997	2426673	NZ_CP030323.1	AR_475	
STAU002.0321.00202.C001.PICI.TypeB.variant0002.Set1.prt	2165383	2175720	NZ_CP022582.1	FORC_062	
STAU002.0321.00204.C001.PICI.TypeB.variant0002.Set1.prt	2440821	2451521	NZ_CP022903.1		187
STAU002.0321.00204.C001.PICI.TypeB.variant0002.Set2.prt	1986477	1996559	NZ_CP022903.1		187
STAU002.0321.00205.C001.PICI.TypeB.variant0002.Set1.prt	1986477	1996559	NZ_CP022903.1		187
STAU002.0321.00205.C001.PICI.TypeB.variant0002.Set2.prt	2440821	2451521	NZ_CP022903.1		187
STAU002.0321.00207.C001.PICI.TypeB.variant0002.Set1.prt	2079582	2090365	NZ_CP022900.1		468
STAU002.0321.00207.C001.PICI.TypeB.variant0002.Set2.prt	360480	371342	NZ_CP022900.1		468

STAU002.0321.00209.C001.PICI.TypeB.variant0002.Set1.prt	870566	883245	NZ_CP009828.1	MS4	
STAU002.0321.00211.C001.PICI.TypeB.variant0002.Set1.prt	401087	411787	NZ_CP022893.1		61
STAU002.0321.00211.C001.PICI.TypeB.variant0002.Set2.prt	856049	866131	NZ_CP022893.1		61
STAU002.0321.00212.C001.PICI.TypeB.variant0002.Set1.prt	360481	371343	NZ_CP022899.1		143
STAU002.0321.00212.C001.PICI.TypeB.variant0002.Set2.prt	2079557	2090340	NZ_CP022899.1		143
STAU002.0321.00213.C001.PICI.TypeB.variant0002.Set1.prt	360480	371342	NZ_CP022900.1		468
STAU002.0321.00213.C001.PICI.TypeB.variant0002.Set2.prt	2079583	2090366	NZ_CP022901.1		466
STAU002.0321.00214.C001.PICI.TypeB.variant0002.Set1.prt	401087	411787	NZ_CP022893.1		61
STAU002.0321.00214.C001.PICI.TypeB.variant0002.Set2.prt	856050	866132	NZ_CP022894.1		191
STAU002.0321.00216.C001.PICI.TypeB.variant0002.Set1.prt	832940	845619	NZ_CP022892.1		54
STAU002.0321.00218.C001.PICI.TypeB.variant0002.Set2.prt	828788	841466	NZ_CP022910.1		164
STAU002.0321.00221.C001.PICI.TypeB.variant0002.Set1.prt	870567	883246	NZ_CP022908.1		545
STAU002.0321.00222.C001.PICI.TypeB.variant0002.Set1.prt	870570	883249	NZ_CP022906.1		546
STAU002.0321.00223.C001.PICI.TypeB.variant0002.Set1.prt	401060	411760	NZ_CP022905.1		628
STAU002.0321.00223.C001.PICI.TypeB.variant0002.Set2.prt	856022	866104	NZ_CP022905.1		628
STAU002.0321.00224.C001.PICI.TypeB.variant0002.Set1.prt	2408872	2419241	NZ_CP031537.1	WCH-SK2	
STAU002.0321.00224.C001.PICI.TypeB.variant0002.Set3.prt	785651	801677	NZ_CP031537.1	WCH-SK2	
STAU002.0321.00224.C001.PICI.TypeB.variant0002.Set4.prt	2457982	2470093	NZ_CP031537.1	WCH-SK2	
STAU002.0321.00226.C001.PICI.TypeB.variant0002.Set2.prt	2079780	2090482	NZ_CP031661.1		82
STAU002.0321.00227.C001.PICI.TypeB.variant0002.Set2.prt	2079780	2090482	NZ_CP031661.1		82
STAU002.0321.00229.C001.PICI.TypeB.variant0002.Set1.prt	829396	840189	NZ_CP031673.1	MOZ66	
STAU002.0321.00245.C001.PICI.TypeB.variant0002.Set1.prt	2143654	2154226	NZ_AP017891.1	GN3	
STAU002.0321.00246.C001.PICI.TypeB.variant0002.Set1.prt	2143816	2154388	NZ_AP018349.1	GN1	
STAU002.0321.00247.C001.PICI.TypeB.variant0002.Set1.prt	417953	428724	NZ_CP032481.1	O326	
STAU002.0321.00248.C001.PICI.TypeB.variant0002.Set1.prt	320256	332396	NZ_CP033865.1	FDAARGOS_504	
STAU002.0321.00249.C001.PICI.TypeB.variant0002.Set1.prt	814719	826859	NZ_CP034098.1	80wphwpl_v1	
STAU002.0321.00250.C001.PICI.TypeB.variant0002.Set1.prt	814785	826925	NZ_CP034349.1	80wphwpl	
STAU002.0321.00251.C001.PICI.TypeB.variant0002.Set2.prt	767112	777449	NZ_AP019305.1	TUM9458	
STAU002.0321.00251.C001.PICI.TypeB.variant0002.Set3.prt	2331620	2341770	NZ_AP019305.1	TUM9458	
STAU002.0321.00252.C001.PICI.TypeB.variant0002.Set2.prt	761529	771866	NZ_AP019306.1	TUM9463	
STAU002.0321.00252.C001.PICI.TypeB.variant0002.Set3.prt	2439716	2449866	NZ_AP019306.1	TUM9463	
STAU002.0321.00255.C001.PICI.TypeB.variant0002.Set1.prt	2511303	2521215	NZ_CP034486.1	PMB 64-1	
STAU002.0321.00256.C001.PICI.TypeB.variant0002.Set1.prt	1556119	1568248	NZ_CP029474.1	USA 100	
STAU002.0321.00261.C001.PICI.TypeB.variant0002.Set1.prt	919519	931630	NZ_CP030138.1	M48	
STAU002.0321.00267.C001.PICI.TypeB.variant0002.Set3.prt	2125419	2136212	NZ_CP038021.1	04-002	
STAU002.0321.00272.C001.PICI.TypeB.variant0002.Set1.prt	417953	428724	NZ_CP032481.1	O326	

STAU002.0321.00273.C001.PICI.TypeB.variant0002.Set1.prt	820309	832985	NZ_CP038269.1	O331
STAU002.0321.00274.C001.PICI.TypeB.variant0002.Set1.prt	874572	886281	NZ_CP038268.1	O55
STAU002.0321.00276.C001.PICI.TypeB.variant0002.Set1.prt	389162	399932	NZ_CP039156.1	WCUH29
STAU002.0321.00277.C001.PICI.TypeB.variant0002.Set1.prt	419235	429922	NZ_CP038819.1	O82
STAU002.0321.00280.C001.PICI.TypeB.variant0004.SetR1.prt	2905127	2931524	CP039157.1	P10
STAU002.0321.00281.C001.PICI.TypeB.variant0002.Set1.prt	824848	835343	unknown	unknown
STAU002.0321.00282.C001.PICI.TypeB.variant0002.Set1.prt	763493	773999	NZ_CP039167.1	R50
STAU002.0321.00284.C001.PICI.TypeB.variant0002.Set1.prt	812632	823131	NZ_CP039992.1	Lr3
STAU002.0321.00287.C001.PICI.TypeB.variant0002.Set2.prt	915674	927785	NZ_CP040622.1	JKD6004
STAU002.0321.00287.C001.PICI.TypeB.variant0002.Set3.prt	866564	876933	NZ_CP040622.1	JKD6004
STAU002.0321.00293.C001.PICI.TypeB.variant0002.Set1.prt	898160	908310	NZ_CP029198.1	aureus
STAU002.0321.00293.C001.PICI.TypeB.variant0002.Set3.prt	2235357	2245694	NZ_CP029198.1	aureus
STAU002.0321.00295.C001.PICI.TypeB.variant0002.Set1.prt	860966	873643	NZ_CP040801.1	S15
STAU002.0321.00297.C001.PICI.TypeB.variant0002.Set1.prt	18916	29534	NZ_CP041010.1	FDAARGOS_766
STAU002.0321.00298.C001.PICI.TypeB.variant0002.Set2.prt	2175524	2186176	NZ_AP019751.1	JRA307
STAU002.0321.00299.C001.PICI.TypeB.variant0002.Set1.prt	866564	876933	NZ_CP040622.1	JKD6004
STAU002.0321.00299.C001.PICI.TypeB.variant0002.Set2.prt	915674	927785	NZ_CP040622.1	JKD6004
STAU002.0321.00302.C001.PICI.TypeB.variant0002.Set1.prt	1069283	1081218	NZ_CP042043.1	B2-15A
STAU002.0321.00303.C001.PICI.TypeB.variant0002.Set1.prt	443668	454895	NZ_CP042153.1	B4-59C
STAU002.0321.00303.C001.PICI.TypeB.variant0002.Set2.prt	914737	926672	NZ_CP042153.1	B4-59C
STAU002.0321.00304.C001.PICI.TypeB.variant0002.Set1.prt	1402214	1414149	NZ_CP042107.1	B8-13D
STAU002.0321.00305.C001.PICI.TypeB.variant0002.Set1.prt	1201785	1213013	NZ_CP042157.1	B3-17D
STAU002.0321.00305.C001.PICI.TypeB.variant0002.Set2.prt	1672867	1684802	NZ_CP042157.1	B3-17D
STAU002.0321.00306.C001.PICI.TypeB.variant0002.Set1.prt	2007528	2018777	NZ_CP031265.1	13
STAU002.0321.00307.C001.PICI.TypeB.variant0002.Set1.prt	1990362	2000861	NZ_CP041037.1	NP66
STAU002.0321.00310.C001.PICI.TypeB.variant0002.Set1.prt	900514	912449	NZ_CP042650.1	X22
STAU002.0321.00316.C001.PICI.TypeB.variant0002.Set1.prt	863285	875960	NZ_CP039448.1	VGC1
STAU002.0321.00316.C001.PICI.TypeB.variant0002.Set2.prt	2083494	2093993	NZ_CP039448.1	VGC1
STAU002.0321.00318.C001.PICI.TypeB.variant0002.Set1.prt	266323	279002	NZ_CP043302.1	16445
STAU002.0321.00320.C001.PICI.TypeB.variant0002.Set1.prt	2873205	2883504	NZ_AP020324.1	KUN1163
STAU002.0321.00321.C001.PICI.TypeB.variant0002.Set2.prt	1642601	1653344	NZ_AP020311.1	KUH140013
STAU002.0321.00321.C001.PICI.TypeB.variant0002.Set3.prt	1190837	1200816	NZ_AP020311.1	KUH140013
STAU002.0321.00325.C001.PICI.TypeB.variant0002.Set2.prt	1661206	1671876	NZ_AP020318.1	KUH180038
STAU002.0321.00326.C001.PICI.TypeB.variant0002.Set2.prt	2898357	2908655	NZ_AP020320.1	KUH180062
STAU002.0321.00327.C001.PICI.TypeB.variant0002.Set1.prt	2836040	2846377	NZ_AP020322.1	KUH180129
STAU002.0321.00328.C001.PICI.TypeB.variant0002.Set1.prt	1406466	1419142	NZ_CP044106.1	FDAARGOS_660

STAU002.0321.00329.C001.PICI.TypeB.variant0002.Set2.prt	2155057	2165394	NZ_AP019542.1	KG-03
STAU002.0321.00329.C001.PICI.TypeB.variant0002.Set3.prt	847667	857817	NZ_AP019542.1	KG-03
STAU002.0321.00330.C001.PICI.TypeB.variant0002.Set1.prt	852994	863144	NZ_AP019543.1	KG-18
STAU002.0321.00330.C001.PICI.TypeB.variant0002.Set3.prt	2166200	2176537	NZ_AP019543.1	KG-18
STAU002.0321.00331.C001.PICI.TypeB.variant0002.Set1.prt	851393	861543	NZ_AP019545.1	KG-22
STAU002.0321.00331.C001.PICI.TypeB.variant0002.Set3.prt	2165549	2175886	NZ_AP019545.1	KG-22
STAU002.0321.00332.C001.PICI.TypeB.variant0002.Set1.prt	71802	81883	NZ_CP045866.1	CFSAN007894
STAU002.0321.00333.C001.PICI.TypeB.variant0002.Set1.prt	680751	693425	NZ_CP045472.1	ZY05
STAU002.0321.00334.C001.PICI.TypeB.variant0002.Set2.prt	2162095	2172888	NZ_CP033112.1	ST20130944
STAU002.0321.00334.C001.PICI.TypeB.variant0002.Set3.prt	357254	368116	NZ_CP033112.1	ST20130944
STAU002.0321.00335.C001.PICI.TypeB.variant0002.Set2.prt	2120257	2131050	NZ_CP033114.1	ST20130945
STAU002.0321.00335.C001.PICI.TypeB.variant0002.Set3.prt	357254	368116	NZ_CP033112.1	ST20130944
STAU002.0321.00336.C001.PICI.TypeB.variant0002.Set1.prt	857810	869950	NZ_AP019712.1	Tokyo12480
STAU002.0321.00337.C001.PICI.TypeB.variant0002.Set1.prt	437459	447677	NZ_CP033977.1	P2D15C1
STAU002.0321.00338.C001.PICI.TypeB.variant0002.Set1.prt	437459	447677	NZ_CP033977.1	P2D15C1
STAU002.0321.00339.C001.PICI.TypeB.variant0002.Set1.prt	437459	447677	NZ_CP033977.1	P2D15C1
STAU002.0321.00350.C001.PICI.TypeB.variant0002.Set1.prt	2543716	2554324	NZ_CP047321.1	RJ1267
STAU002.0321.00350.C001.PICI.TypeB.variant0002.Set2.prt	2058317	2068749	NZ_CP047321.1	RJ1267
STAU002.0321.00350.C001.PICI.TypeB.variant0002.Set3.prt	814594	824891	NZ_CP047321.1	RJ1267
STAU002.0321.00357.C001.PICI.TypeB.variant0002.Set1.prt	2817672	2828465	NZ_CP047778.1	UP_1572
STAU002.0321.00357.C001.PICI.TypeB.variant0002.Set2.prt	1725674	1736430	NZ_CP047778.1	UP_1572
STAU002.0321.00359.C001.PICI.TypeB.variant0002.Set1.prt	2819187	2829980	NZ_CP047805.1	UP_1654
STAU002.0321.00359.C001.PICI.TypeB.variant0002.Set3.prt	1725981	1736842	NZ_CP047805.1	UP_1654
STAU002.0321.00362.C001.PICI.TypeB.variant0002.Set1.prt	68408	79270	NZ_CP047779.1	UP_1559
STAU002.0321.00366.C001.PICI.TypeB.variant0002.Set1.prt	825190	837866	NZ_CP047783.1	UP_1313
STAU002.0321.00370.C001.PICI.TypeB.variant0002.Set1.prt	2909070	2919797	NZ_CP047781.1	UP_1452
STAU002.0321.00370.C001.PICI.TypeB.variant0002.Set3.prt	1815391	1826253	NZ_CP047781.1	UP_1452
STAU002.0321.00372.C001.PICI.TypeB.variant0002.Set1.prt	68410	79272	NZ_CP047782.1	UP_1352
STAU002.0321.00372.C001.PICI.TypeB.variant0002.Set2.prt	1839392	1850184	NZ_CP047782.1	UP_1352
STAU002.0321.00374.C001.PICI.TypeB.variant0002.Set1.prt	2745923	2756343	NZ_CP047786.1	UP_1150
STAU002.0321.00376.C001.PICI.TypeB.variant0002.Set1.prt	2806674	2817467	NZ_CP047788.1	UP_1033
STAU002.0321.00380.C001.PICI.TypeB.variant0002.Set1.prt	1687277	1698139	NZ_CP047793.1	UP_764
STAU002.0321.00380.C001.PICI.TypeB.variant0002.Set2.prt	2777838	2788631	NZ_CP047793.1	UP_764
STAU002.0321.00382.C001.PICI.TypeB.variant0002.Set2.prt	1834953	1845746	NZ_CP047791.1	UP_818
STAU002.0321.00382.C001.PICI.TypeB.variant0002.Set3.prt	69603	80465	NZ_CP047791.1	UP_818
STAU002.0321.00386.C001.PICI.TypeB.variant0002.Set1.prt	1796314	1807176	NZ_CP047801.1	UP_248

STAU002.0321.00386.C001.PICI.TypeB.variant0002.Set2.prt	2892215	2903008	NZ_CP047801.1	UP_248
STAU002.0321.00387.C001.PICI.TypeB.variant0002.Set1.prt	1629688	1640311	NZ_CP047798.1	UP_378
STAU002.0321.00398.C001.PICI.TypeB.variant0002.Set1.prt	1635101	1646180	NZ_CP047852.1	UP_274
STAU002.0321.00400.C001.PICI.TypeB.variant0002.Set1.prt	1669662	1680388	NZ_CP047799.1	UP_322
STAU002.0321.00407.C001.PICI.TypeB.variant0002.Set1.prt	375338	386200	NZ_CP047851.1	UP_338
STAU002.0321.00409.C001.PICI.TypeB.variant0002.Set1.prt	2809163	2819331	NZ_CP047809.1	UP_1591
STAU002.0321.00409.C001.PICI.TypeB.variant0002.Set2.prt	1193177	1203453	NZ_CP047809.1	UP_1591
STAU002.0321.00410.C001.PICI.TypeB.variant0002.Set2.prt	2333735	2343885	NZ_CP048643.1	SR153
STAU002.0321.00410.C001.PICI.TypeB.variant0002.Set3.prt	1109695	1120031	NZ_CP048643.1	SR153
STAU002.0321.00416.C001.PICI.TypeB.variant0002.Set1.prt	871059	883734	NZ_CP053070.1	HL20709
STAU002.0321.00417.C001.PICI.TypeB.variant0002.Set1.prt	871804	883941	NZ_CP053075.1	SA01
STAU002.0321.00419.C001.PICI.TypeB.variant0002.Set1.prt	897989	910665	NZ_CP053185.1	Guangzhou-SAU749
STAU002.0321.00422.C001.PICI.TypeB.variant0002.Set1.prt	1151793	1163933	NZ_CP053634.1	14638
STAU002.0321.00423.C001.PICI.TypeB.variant0002.Set1.prt	1151798	1163938	NZ_CP053636.1	14640
STAU002.0321.00432.C001.PICI.TypeB.variant0002.Set1.prt	901905	913861	NZ_CP045435.1	08-028
STAU002.0321.00433.C001.PICI.TypeB.variant0002.Set1.prt	356791	367550	NZ_CP059156.1	WKZ-1
STAU002.0321.00433.C001.PICI.TypeB.variant0002.Set3.prt	2119170	2129963	NZ_CP059156.1	WKZ-1
STAU002.0321.00434.C001.PICI.TypeB.variant0002.Set1.prt	381039	391798	NZ_CP059155.1	WKZ-2
STAU002.0321.00434.C001.PICI.TypeB.variant0002.Set3.prt	2143419	2154212	NZ_CP059155.1	WKZ-2
STAU002.0321.00435.C001.PICI.TypeB.variant0002.Set1.prt	860117	870414	NZ_CP058615.1	110900
STAU002.0321.00435.C001.PICI.TypeB.variant0002.Set2.prt	2101883	2112316	NZ_CP058615.1	110900
STAU002.0321.00435.C001.PICI.TypeB.variant0002.Set3.prt	2547871	2558479	NZ_CP058615.1	110900
STAU002.0321.00436.C001.PICI.TypeB.variant0002.Set1.prt	2522871	2533479	NZ_CP058613.1	128254
STAU002.0321.00436.C001.PICI.TypeB.variant0002.Set2.prt	830433	840730	NZ_CP058613.1	128254
STAU002.0321.00436.C001.PICI.TypeB.variant0002.Set3.prt	2035576	2046009	NZ_CP058613.1	128254
STAU002.0321.00437.C001.PICI.TypeB.variant0002.Set1.prt	2673259	2683410	NZ_CP062469.1	MRSA - AMRF 4
STAU002.0321.00438.C001.PICI.TypeB.variant0002.Set1.prt	77457	87956	NZ_CP062467.1	MRSA - AMRF 5
STAU002.0321.00439.C001.PICI.TypeB.variant0002.Set1.prt	2618265	2628416	NZ_CP062465.1	MRSA - AMRF 6
STAU002.0321.00440.C001.PICI.TypeB.variant0002.Set1.prt	2502649	2513148	NZ_CP062471.1	MRSA - AMRF 3
STAU002.0321.00441.C001.PICI.TypeB.variant0002.Set1.prt	826932	837230	NZ_CP060584.1	WH52
STAU002.0321.00441.C001.PICI.TypeB.variant0002.Set2.prt	2501020	2511491	NZ_CP060584.1	WH52
STAU002.0321.00450.C001.PICI.TypeB.variant0002.Set3.prt	366208	377262	NZ_CP066488.1	ncr_155_F133
STAU002.0321.00450.C001.PICI.TypeB.variant0002.Set4.prt	836953	847166	NZ_CP066488.1	ncr_155_F133
STAU002.0321.00451.C001.PICI.TypeB.variant0002.Set1.prt	821419	831632	NZ_CP066492.1	nan_175_F371_ch
STAU002.0321.00451.C001.PICI.TypeB.variant0002.Set2.prt	364459	375345	NZ_CP066492.1	nan_175_F371_ch
STAU002.0321.00452.C001.PICI.TypeB.variant0002.Set1.prt	2527384	2537992	NZ_CP060491.1	WH39

STAU002.0321.00452.C001.PICI.TypeB.variant0002.Set2.prt	860292	870589	NZ_CP060491.1	WH39	
STAU002.0321.00453.C001.PICI.TypeB.variant0002.Set2.prt	2099021	2109358	NZ_AP024170.1		834
STAU002.0321.00457.C001.PICI.TypeB.variant0002.Set1.prt	365872	376951	NZ_CP069799.1	014S_SA	
STAU002.0321.00492.C001.PICI.TypeB.variant0002.Set1.prt	412830	423526	NZ_LS483301.1	NCTC13394	
STAU002.0321.00492.C001.PICI.TypeB.variant0002.Set2.prt	2184038	2194537	NZ_LS483301.1	NCTC13394	
STAU002.0321.00493.C001.PICI.TypeB.variant0002.Set2.prt	388313	399068	NZ_LS483300.1	NCTC7485	
STAU002.0321.00496.C001.PICI.TypeB.variant0002.Set1.prt	431960	442538	NZ_LS483309.1	NCTC9944	
STAU002.0321.00498.C001.PICI.TypeB.variant0002.Set2.prt	2041487	2051228	NZ_LS483314.1	NCTC3761	
STAU002.0321.00499.C001.PICI.TypeB.variant0002.Set1.prt	2075891	2086101	NZ_LS483317.1	NCTC5663	
STAU002.0321.00500.C001.PICI.TypeB.variant0002.Set2.prt	405878	415975	NZ_LS483311.1	NCTC6136	
STAU002.0321.00503.C001.PICI.TypeB.variant0002.Set1.prt	2101016	2110757	NZ_LS483324.1	NCTC10344	
STAU002.0321.00504.C001.PICI.TypeB.variant0002.Set2.prt	857013	869124	NZ_LS483350.1	NCTC11940	
STAU002.0321.00505.C001.PICI.TypeB.variant0002.Set1.prt	409663	420524	NZ_LS483484.1	NCTC13277	
STAU002.0321.00506.C001.PICI.TypeB.variant0002.Set2.prt	392170	402695	NZ_LR027876.1	JKD6009	
STAU002.0321.00506.C001.PICI.TypeB.variant0002.Set3.prt	904975	917086	NZ_LR027876.1	JKD6009	
STAU002.0321.00507.C001.PICI.TypeB.variant0002.Set2.prt	858049	868418	NZ_LR027870.1	BPH2019	
STAU002.0321.00508.C001.PICI.TypeB.variant0002.Set2.prt	954879	966990	NZ_LR027878.1	BPH2003	
STAU002.0321.00509.C001.PICI.TypeB.variant0002.Set1.prt	2164305	2174804	NZ_LR027873.1	BPH2070	
STAU002.0321.00509.C001.PICI.TypeB.variant0002.Set2.prt	934240	946351	NZ_LR027873.1	BPH2070	
STAU002.0321.00510.C001.PICI.TypeB.variant0002.Set1.prt	905796	917906	NZ_LR027877.1	unknown	
STAU002.0321.00510.C001.PICI.TypeB.variant0002.Set2.prt	856645	867014	NZ_LR027877.1	unknown	
STAU002.0321.00511.C001.PICI.TypeB.variant0002.Set1.prt	393512	404037	NZ_LR027869.1	unknown	
STAU002.0321.00511.C001.PICI.TypeB.variant0002.Set3.prt	870727	881095	NZ_LR027869.1	unknown	
STAU002.0321.00512.C001.PICI.TypeB.variant0002.Set1.prt	916598	928709	NZ_LR027874.1	BPH2056	
STAU002.0321.00513.C001.PICI.TypeB.variant0002.Set1.prt	369418	379330	NZ_LR130509.1	BPH2760	
STAU002.0321.00514.C001.PICI.TypeB.variant0002.Set1.prt	790321	800368	NZ_LR130511.1	BPH2819	
STAU002.0321.00516.C001.PICI.TypeB.variant0002.Set1.prt	956193	968304	NZ_LR130515.1	BPH2947	
STAU002.0321.00518.C001.PICI.TypeB.variant0002.Set1.prt	365930	377009	NZ_LR133917.1	NCTC8317	
STAU002.0321.00521.C001.PICI.TypeB.variant0002.Set1.prt	2101006	2110747	NZ_LR134087.1	NCTC7121	
STAU002.0321.00524.C001.PICI.TypeB.variant0002.Set1.prt	358459	369321	NZ_LR134093.1	NCTC11965	
STAU002.0321.00524.C001.PICI.TypeB.variant0002.Set4.prt	2097959	2108752	NZ_LR134093.1	NCTC11965	
STAU002.0321.00525.C001.PICI.TypeB.variant0002.Set1.prt	842710	854952	NZ_LR134139.1	NCTC4163	
STAU002.0321.00529.C001.PICI.TypeB.variant0002.Set1.prt	61720	72583	NZ_LR134351.1	NCTC13811	
STAU002.0321.00529.C001.PICI.TypeB.variant0002.Set3.prt	1892473	1903266	NZ_LR134351.1	NCTC13811	
STAU002.0321.00530.C001.PICI.TypeB.variant0002.Set1.prt	1254098	1266054	NZ_LR822060.1	unknown	
STAU002.0321.00530.C001.PICI.TypeB.variant0002.Set2.prt	1748459	1759321	NZ_LR822060.1	unknown	

STAU002.0321.00530.C001.PICI.TypeB.variant0002.Set3.prt	1299042	1309124	NZ_LR822060.1	unknown	
STAU002.0321.00531.C001.PICI.TypeB.variant0002.Set1.prt	1749794	1760656	NZ_LR822061.1	unknown	
STAU002.0321.00531.C001.PICI.TypeB.variant0002.Set2.prt	1255433	1267389	NZ_LR822061.1	unknown	
STAU002.0321.00531.C001.PICI.TypeB.variant0002.Set3.prt	1300377	1310459	NZ_LR822061.1	unknown	
STAU002.0321.00534.C001.PICI.TypeB.variant0002.Set1.prt	903491	916170	NC_002951.2	COL	
STAU002.0321.00535.C001.PICI.TypeB.variant0002.Set1.prt	868462	878612	NC_002758.2	Mu50	
STAU002.0321.00535.C001.PICI.TypeB.variant0002.Set3.prt	2138368	2148702	NC_002758.2	Mu50	
STAU002.0321.00536.C001.PICI.TypeB.variant0002.Set2.prt	2061935	2072272	NC_002745.2	N315	
STAU002.0321.00538.C001.PICI.TypeB.variant0002.Set2.prt	388840	399595	NC_007622.1	RF122	
STAU002.0321.00539.C001.PICI.TypeB.variant0002.Set1.prt	410176	421037	NC_002952.2	MRSA252	
STAU002.0321.00543.C001.PICI.TypeB.variant0002.Set1.prt	869907	880057	NC_009782.1	Mu3	
STAU002.0321.00543.C001.PICI.TypeB.variant0002.Set3.prt	2139992	2150340	NC_009782.1	Mu3	
STAU002.0321.00549.C001.PICI.TypeB.variant0002.Set1.prt	904438	916548	NC_017341.1	JKD6008	
STAU002.0321.00549.C001.PICI.TypeB.variant0002.Set2.prt	391631	402156	NC_017341.1	JKD6008	
STAU002.0321.00550.C001.PICI.TypeB.variant0002.Set1.prt	2464885	2477024	NC_017342.1	MRSA_TCH60	
STAU002.0321.00551.C001.PICI.TypeB.variant0002.Set1.prt	778770	790910	NC_022113.1	55/2053	
STAU002.0321.00552.C001.PICI.TypeB.variant0002.Set1.prt	952272	964383	NC_017331.1	TW20	
STAU002.0321.00553.C001.PICI.TypeB.variant0002.Set1.prt	830711	842646	NC_013450.1	ED98	
STAU002.0321.00559.C001.PICI.TypeB.variant0002.Set1.prt	419237	429925	NZ_CP025395.1	O46	
STAU002.0321.00560.C001.PICI.TypeB.variant0002.Set1.prt	852047	862196	NC_017349.1	LGA251	
STAU002.0321.00561.C001.PICI.TypeB.variant0002.Set2.prt	2117350	2134114	NC_017347.1	T0131	
STAU002.0321.00561.C001.PICI.TypeB.variant0002.Set3.prt	387681	398260	NC_017347.1	T0131	
STAU002.0321.00572.C001.PICI.TypeB.variant0002.Set1.prt	871515	884190	NC_016928.2	M013	
STAU002.0321.00575.C001.PICI.TypeB.variant0002.Set1.prt	847044	859720	NC_022443.1	SA40	
STAU002.0321.00576.C001.PICI.TypeB.variant0002.Set1.prt	865434	878109	NC_022442.1	SA957	
STAU002.0321.00579.C001.PICI.TypeB.variant0002.Set1.prt	885905	896404	NZ_CP010526.1	DAR4145	
STAU002.0321.00580.C001.PICI.TypeB.variant0002.Set1.prt	855298	867973	NZ_CP006630.1	SA268	
STAU002.0321.00581.C001.PICI.TypeB.variant0002.Set1.prt	1200508	1210725	NZ_CP012409.1	Tager 104	
STAU002.0321.00582.C001.PICI.TypeB.variant0002.Set1.prt	1978513	1989086	NC_022222.1		6850
STAU002.0321.00583.C001.PICI.TypeB.variant0002.Set1.prt	909214	921325	NC_022604.1	Z172	
STCA001.0321.00002.C001.PICI.TypeB.variant0002.Set1.prt	721046	731475	NZ_CP065711.1	FDAARGOS_883	
STCA001.0321.00005.C001.PICI.TypeB.variant0002.Set1.prt	1545018	1555446	NC_012121.1	TM300	
STCA006.0321.00002.C001.PICI.TypeB.variant0002.Set1.prt	987401	998102	NZ_AP018586.1	JMUB590	
STCA006.0321.00002.C001.PICI.TypeB.variant0002.Set2.prt	2565082	2577204	NZ_AP018586.1	JMUB590	
STCA006.0321.00003.C001.PICI.TypeB.variant0002.Set1.prt	972571	983272	NZ_AP018587.1	JMUB898	
STCA006.0321.00004.C001.PICI.TypeB.variant0002.Set2.prt	2599720	2611169	NZ_CP031271.1	26D	

STCA006.0321.00005.C001.PICI.TypeB.variant0002.Set1.prt	97973	108829	NZ_CP051643.1	SY333
STCA007.0321.00003.C001.PICI.TypeB.variant0002.Set2.prt	2418323	2428474	NZ_CP053957.1	FDAARGOS_753
STCA007.0321.00004.C001.PICI.TypeB.variant0002.Set1.prt	1991460	2001747	NZ_CP042341.1	BN2
STCH004.0321.00001.C001.PICI.TypeB.variant0002.Set1.prt	1896123	1906506	NZ_CP031274.1	17A
STCO001.0321.00001.C001.PICI.TypeB.variant0001.Set2.prt	1480766	1487901	NZ_CP023694.1	ATCC 13740
STCO003.0321.00001.C001.PICI.TypeB.variant0002.Set1.prt	177333	188448	NZ_CP027422.1	FDAARGOS_334
STCO003.0321.00002.C001.PICI.TypeB.variant0002.Set1.prt	539463	549276	NZ_CP033735.1	FDAARGOS_538
STCO003.0321.00002.C001.PICI.TypeB.variant0002.Set2.prt	506800	518330	NZ_CP033735.1	FDAARGOS_538
STCO005.0321.00002.C001.PICI.TypeB.variant0002.Set1.prt	1087184	1099328	NZ_CP018776.1	StO 2014-01
STDE001.0321.00001.C001.PICI.TypeB.variant0002.Set1.prt	1207691	1219280	NZ_CP033460.1	SDB 2975
STEP001.0321.00002.C001.PICI.TypeB.variant0002.Set2.prt	551797	562835	NZ_CP018842.1	14.1.R1
STEP001.0321.00004.C001.PICI.TypeB.variant0002.Set1.prt	1418903	1428267	NZ_CP022247.1	AMT
STEP001.0321.00005.C001.PICI.TypeB.variant0002.Set1.prt	1658206	1668906	NZ_CP013943.1	DAR1907
STEP001.0321.00008.C001.PICI.TypeB.variant0002.Set1.prt	2004776	2014116	NZ_CP030246.1	CSF41498
STEP001.0321.00009.C001.PICI.TypeB.variant0002.Set1.prt	1467600	1476964	NZ_CP033782.1	FDAARGOS_529
STEP001.0321.00011.C001.PICI.TypeB.variant0002.Set1.prt	1573690	1583817	NZ_CP034115.1	CDC121
STEP001.0321.00018.C001.PICI.TypeB.variant0002.Set1.prt	1605606	1615746	NZ_CP035643.1	E73
STEP001.0321.00020.C001.PICI.TypeB.variant0002.Set1.prt	869282	880928	NZ_CP060528.1	LM087
STEP001.0321.00021.C001.PICI.TypeB.variant0002.Set2.prt	792940	805430	NZ_CP060794.1	Z0118SE0260
STEP001.0321.00022.C001.PICI.TypeB.variant0002.Set1.prt	262433	273239	NZ_CP061029.1	Z0118SE0132
STEP001.0321.00024.C001.PICI.TypeB.variant0002.Set1.prt	1587005	1597279	NZ_CP066303.1	SE48
STEP001.0321.00027.C001.PICI.TypeB.variant0002.Set1.prt	866100	876894	NZ_CP069215.1	Z0118SE0269
STEP001.0321.00029.C001.PICI.TypeB.variant0002.Set1.prt	551314	562514	NZ_CP069473.1	FDAARGOS_1243
STEP001.0321.00030.C001.PICI.TypeB.variant0002.Set1.prt	2428316	2439516	NZ_CP069951.1	FDAARGOS_1363
STEP001.0321.00031.C001.PICI.TypeB.variant0002.Set1.prt	2099937	2109936	NZ_CP069954.1	FDAARGOS_1364
STEP001.0321.00032.C001.PICI.TypeB.variant0002.Set1.prt	1792273	1803055	NZ_CP070057.1	FDAARGOS_1361
STEP001.0321.00033.C001.PICI.TypeB.variant0002.Set1.prt	1749852	1759972	NZ_LT571449.1	unknown
STEP001.0321.00034.C001.PICI.TypeB.variant0002.Set2.prt	1754585	1765757	NZ_LR134536.1	NCTC13924
STEP001.0321.00036.C001.PICI.TypeB.variant0002.Set1.prt	2237685	2249566	NZ_LR735429.1	none
STEQ003.0321.00007.C001.PICI.TypeB.variant0002.Set1.prt	531170	540887	NZ_CP068069.1	FDAARGOS_1149
STHA002.0321.00009.C001.PICI.TypeB.variant0002.Set1.prt	604234	614599	CP063443.1	GDY8P80P
STHA002.0321.00010.C001.PICI.TypeB.variant0002.Set1.prt	2122930	2133151	NC_007168.1	JCSC1435
STHO001.0321.00001.C001.PICI.TypeB.variant0002.Set2.prt	1388518	1399794	NZ_CP033732.1	FDAARGOS_575
STHO001.0321.00003.C001.PICI.TypeB.variant0002.Set2.prt	1750062	1761522	NZ_CP046301.1	FDAARGOS_747
STHO001.0321.00005.C001.PICI.TypeB.variant0002.Set1.prt	1422955	1435413	NZ_CP054006.1	FDAARGOS_762
STHO001.0321.00006.C001.PICI.TypeB.variant0002.Set1.prt	638575	649670	NZ_CP020618.1	K1

STLU004.0321.00001.C001.PICI.TypeB.variant0002.Set1.prt	1363569	1373889	NZ_CP014022.1	FDAARGOS_141
STLU004.0321.00001.C001.PICI.TypeB.variant0002.Set2.prt	2448896	2458211	NZ_CP014022.1	FDAARGOS_141
STLU004.0321.00004.C001.PICI.TypeB.variant0002.Set1.prt	2519008	2529327	NZ_CP017069.2	K93G
STLU004.0321.00004.C001.PICI.TypeB.variant0002.Set2.prt	1021354	1030669	NZ_CP017069.2	K93G
STLU004.0321.00005.C001.PICI.TypeB.variant0002.Set1.prt	1933669	1943988	NZ_CP023539.1	FDAARGOS_377
STLU004.0321.00005.C001.PICI.TypeB.variant0002.Set2.prt	447182	456497	NZ_CP023539.1	FDAARGOS_377
STLU004.0321.00006.C001.PICI.TypeB.variant0002.Set1.prt	2445041	2454356	NZ_CP023970.1	FDAARGOS_381
STLU004.0321.00006.C001.PICI.TypeB.variant0002.Set2.prt	1370518	1380838	NZ_CP023970.1	FDAARGOS_381
STLU004.0321.00010.C001.PICI.TypeB.variant0002.Set1.prt	598045	608514	NZ_CP041726.1	SL118
STLU004.0321.00010.C001.PICI.TypeB.variant0002.Set2.prt	2115087	2125784	NZ_CP041726.1	SL118
STLU004.0321.00012.C001.PICI.TypeB.variant0002.Set1.prt	2349750	2360341	NZ_CP041722.1	SL13
STLU004.0321.00012.C001.PICI.TypeB.variant0002.Set2.prt	1292672	1302425	NZ_CP041722.1	SL13
STLU004.0321.00013.C001.PICI.TypeB.variant0002.Set1.prt	2423961	2433276	NZ_CP038807.1	APC 3758
STLU004.0321.00013.C001.PICI.TypeB.variant0002.Set2.prt	937153	947473	NZ_CP038807.1	APC 3758
STLU004.0321.00014.C001.PICI.TypeB.variant0002.Set1.prt	2621293	2631612	NZ_AP021848.1	JICS135
STLU004.0321.00014.C001.PICI.TypeB.variant0002.Set2.prt	1122237	1131552	NZ_AP021848.1	JICS135
STLU004.0321.00015.C001.PICI.TypeB.variant0002.Set1.prt	2503280	2513600	NZ_CP063143.1	IVK28
STLU004.0321.00016.C001.PICI.TypeB.variant0002.Set1.prt	2541009	2551329	NZ_CP060160.1	MBAZ2
STLU004.0321.00016.C001.PICI.TypeB.variant0002.Set2.prt	1011277	1020592	NZ_CP060160.1	MBAZ2
STLU004.0321.00017.C001.PICI.TypeB.variant0002.Set1.prt	2039656	2049972	NZ_LS483312.1	NCTC7990
STLU004.0321.00019.C001.PICI.TypeB.variant0002.Set2.prt	2077277	2086790	NC_013893.1	HKU09-01
STNO003.0321.00001.C001.PICI.TypeB.variant0001.Set1.prt	2048113	2056407	NC_014217.1	DSM 506
STPA007.0321.00001.C001.PICI.TypeB.variant0002.Set1.prt	1929734	1939098	NZ_CP017463.1	JS7
STPA007.0321.00003.C001.PICI.TypeB.variant0002.Set1.prt	524891	534514	NC_022737.1	SP1
STSA001.0321.00002.C001.PICI.TypeB.variant0002.Set1.prt	672076	690387	NZ_CP009913.1	NCTC 8618
STSA001.0321.00003.C001.PICI.TypeB.variant0002.Set1.prt	828977	847287	NZ_CP014144.1	JF
STSA001.0321.00015.C001.PICI.TypeB.variant0002.Set1.prt	671728	690038	NZ_LR134274.1	NCTC8618
STSA003.0321.00002.C001.PICI.TypeB.variant0002.Set1.prt	903648	913305	NZ_CP014057.2	FDAARGOS_137
STSA003.0321.00003.C001.PICI.TypeB.variant0002.Set1.prt	1437452	1447109	NZ_CP022056.1	FDAARGOS_336
STSA003.0321.00004.C001.PICI.TypeB.variant0002.Set1.prt	1041485	1052369	NZ_CP022093.1	FDAARGOS_355
STSA003.0321.00008.C001.PICI.TypeB.variant0002.SetR1.prt	2129	11546	NZ_CP054444.1	UTI-056
STSA003.0321.00009.C001.PICI.TypeB.variant0002.Set2.prt	897493	907386	NZ_CP054440.1	UTI-058y
STSA003.0321.00010.C001.PICI.TypeB.variant0002.Set1.prt	2078689	2088795	NZ_CP054575.1	UTI-050
STSA003.0321.00010.C001.PICI.TypeB.variant0002.Set2.prt	2046160	2055204	NZ_CP054575.1	UTI-050
STSA003.0321.00010.C001.PICI.TypeB.variant0002.Set3.prt	875245	884899	NZ_CP054575.1	UTI-050
STSA003.0321.00011.C001.PICI.TypeB.variant0002.Set1.prt	2104792	2114763	NZ_CP054831.1	UTI-045

STSA003.0321.00015.C001.PICI.TypeB.variant0002.Set1.prt	1995297	2006181	NC_007350.1	ATCC 15305	
STSA004.0321.00003.C001.PICI.TypeB.variant0002.Set1.prt	960377	971107	NZ_CP066042.1	FDAARGOS_1074	
STSI001.0321.00003.C001.PICI.TypeB.variant0001.SetR1.prt	1027781	1037385	NZ_CP017428.1	MR3	
STSI001.0321.00004.C001.PICI.TypeB.variant0001.SetR1.prt	1027781	1037385	NZ_CP017428.1	MR3	
STSI001.0321.00005.C001.PICI.TypeB.variant0001.SetR1.prt	1027882	1037486	NZ_CP015642.1	MR1	
STSI001.0321.00006.C001.PICI.TypeB.variant0001.SetR1.prt	1027882	1037486	NZ_CP015642.1	MR1	
STSP040.0321.00001.C001.PICI.TypeB.variant0002.Set1.prt	1923927	1938673	NZ_CP022881.1	M0911	
STSP040.0321.00001.C001.PICI.TypeB.variant0002.Set2.prt	2363841	2374749	NZ_CP022881.1	M0911	
STSP098.0321.00001.C001.PICI.TypeB.variant0002.Set1.prt	2899732	2906165	NZ_CP048834.1	ORNL1	
STSP110.0321.00001.C001.PICI.TypeA.Set6.prt	5164063	5173532	CP054916.1	NA02950	
STSP126.0321.00001.C001.PICI.TypeB.variant0002.Set1.prt	2117265	2127895	NZ_CP068241.1	11-B-312	
STSP128.0321.00001.C001.PICI.TypeB.variant0002.Set1.prt	468	10209	NZ_CP070306.1	SM9054	
STSP129.0321.00001.C001.PICI.TypeB.variant0002.Set1.prt	3217	12958	NZ_CP070305.1	SM3655	
STSP130.0321.00001.C001.PICI.TypeB.variant0002.Set1.prt	2319956	2330425	NZ_CP070964.1	SB1-57	
STTE001.0321.00001.C001.PICI.TypeB.variant0002.Set1.prt	5333071	5339252	NZ_CP043959.1		139
STWA001.0321.00001.C001.PICI.TypeB.variant0002.Set1.prt	2430065	2440790	NZ_CP032159.1		22.1
STWA001.0321.00002.C001.PICI.TypeB.variant0002.Set2.prt	2376453	2388530	NZ_CP033098.1	SWO	
STWA001.0321.00003.C001.PICI.TypeB.variant0002.Set1.prt	2385026	2395748	NZ_CP038242.1	GD01	
STWA001.0321.00004.C001.PICI.TypeB.variant0002.Set1.prt	2182747	2192763	NZ_CP053477.1	WB224	
STWA001.0321.00005.C001.PICI.TypeB.variant0002.Set1.prt	2005016	2015506	NZ_CP054017.1	FDAARGOS_754	
STWA001.0321.00006.C001.PICI.TypeB.variant0002.Set1.prt	1475660	1486410	NZ_CP061041.1	WS479	
STWA001.0321.00007.C001.PICI.TypeB.variant0002.Set1.prt	894914	904943	NZ_LR134242.1	NCTC4133	
STWA001.0321.00008.C001.PICI.TypeB.variant0002.Set1.prt	2368910	2380305	NZ_LR134244.1	NCTC7291	
TEAL001.0321.00001.C001.PICI.TypeA.Set1.prt	2899689	2908805	NZ_CP042806.1	ORNL	
THIN005.0321.00001.C001.PICI.TypeB.variant0001.Set1.prt	679715	692680	NZ_CP020046.1	ATCC 15466	
THSP021.0321.00001.C001.PICI.TypeA.Set1.prt	869830	879621	CP037895.1	FW80	
THSP037.0321.00001.C001.PICI.TypeB.variant0002.Set1.prt	1304068	1312022	NC_013889.1	K90mix	
THVE001.0321.00001.C001.PICI.TypeB.variant0001.Set1.prt	1425246	1432168	NZ_CP011367.1	D301	
TISP001.0321.00001.C001.PICI.TypeB.variant0001.Set1.prt	2257995	2264502	NZ_CP035282.1	JN-28	
TRAZ001.0321.00001.C001.PICI.TypeB.variant0001.Set2.prt	1870239	1884354	NC_015577.1	ZAS-9	
TRSP003.0321.00002.C001.PICI.TypeB.variant0001.Set1.prt	2109439	2133104	NZ_CP063212.1	19OD0592	
TUIM001.0321.00001.C001.PICI.TypeB.variant0002.Set1.prt	3192639	3206020	LR586016.1	MBLW1	
VASP012.0321.00001.C001.PICI.TypeB.variant0003.Set1.prt	3431629	3440924	NZ_LR594659.1	unknown	
XAAU001.0321.00001.C001.PICI.TypeB.variant0002.Set1.prt	3818623	3833452	CP000781.1	Py2	

chRoom. The “TAG” column reports the name used in the phylogenetic tree.

Tag
NZ_CP022752.1_PICI_1
NC_016077.1_PICI_1
NZ_CP045135.1_PICI_1
NZ_LR134473.1_PICI_1
NZ_CP025030.1_PICI_1
NC_015422.1_PICI_1
NC_008340.1_PICI_1
NZ_CP049259.1_PICI_1
NZ_CP048649.1_PICI_1
NZ_AP023367.1_PICI_1
CP053688.1_PICI_1
NZ_CP038613.1_PICI_1
CP025682.1_PICI_1
NZ_CP009596.1_PICI_1
NZ_CP009605.1_PICI_1
NZ_CP053991.1_PICI_1
NZ_CP053997.1_PICI_1
NZ_CP059725.1_PICI_1
NZ_CP015350.1_PICI_1
NZ_CP007755.1_PICI_1
NZ_CP010433.1_PICI_1
NZ_CP017098.1_PICI_1
NZ_CP010433.1_PICI_2
NZ_CP042940.1_PICI_1
NZ_CP047190.1_PICI_1
NZ_CP045589.1_PICI_1
NZ_CP010433.1_PICI_3
NZ_CP009045.1_PICI_1
NZ_CP022724.1_PICI_1
NZ_CP031703.1_PICI_1
NZ_CP031154.1_PICI_1
NZ_CP069248.1_PICI_1
NZ_CP069249.1_PICI_1

NC_011835.1_PICI_1
NC_017214.2_PICI_1
NC_017217.1_PICI_1
NC_012814.1_PICI_1
NC_017867.1_PICI_1
NC_017215.1_PICI_1
NC_017216.2_PICI_1
NC_017866.1_PICI_1
NC_021593.1_PICI_1
NZ_CP007522.1_PICI_1
NZ_CP017150.1_PICI_1
NZ_CP009744.1_PICI_1
NZ_CP013363.1_PICI_1
NZ_CP013404.1_PICI_1
NZ_CP009128.1_PICI_1
NZ_CP016638.1_PICI_1
NZ_CP016636.1_PICI_1
NZ_CP033702.1_PICI_1
NZ_CP009298.1_PICI_1
NZ_CP013417.1_PICI_1
NZ_CP033076.1_PICI_1
NZ_CP013409.1_PICI_1
NZ_CP020392.1_PICI_1
NZ_CP004089.1_PICI_1
CP029256.1_PICI_1
unknown_PICI_1
NZ_CP011132.1_PICI_1
NZ_CP056289.1_PICI_1
NZ_CP056289.1_PICI_2
NZ_CP054278.1_PICI_1
NC_010723.1_PICI_1
NZ_CP006903.1_PICI_1
NC_014393.1_PICI_1
NC_009706.1_PICI_1
NC_011837.1_PICI_1
NZ_CP012573.1_PICI_1

NC_008593.1_PICI_1
NZ_CP016757.1_PICI_1
NZ_CP025746.1_PICI_1
NZ_CP065628.1_PICI_1
NZ_CP066023.1_PICI_1
NZ_CP022054.2_PICI_1
NZ_CP033784.1_PICI_1
NZ_CP033898.1_PICI_1
NZ_CP028924.1_PICI_1
NZ_CP014279.1_PICI_1
NZ_CP019963.1_PICI_1
NZ_CP046863.1_PICI_1
NZ_CP054583.1_PICI_1
NZ_CP067087.1_PICI_1
NZ_CP065668.1_PICI_1
NZ_CP060292.1_PICI_1
NC_002937.3_PICI_1
NC_002937.3_PICI_2
NC_017310.1_PICI_1
NZ_CP027238.1_PICI_1
NZ_CP015231.1_PICI_1
NZ_CP017057.1_PICI_1
NZ_CP009106.2_PICI_1
NZ_CP007275.1_PICI_1
NZ_CP012633.1_PICI_1
NZ_CP015020.1_PICI_1
NZ_CP015240.1_PICI_1
NZ_CP015241.1_PICI_1
NZ_CP015229.1_PICI_1
NZ_CP015229.1_PICI_2
NZ_CP015228.1_PICI_1
NZ_CP013663.1_PICI_1
NZ_CP013663.1_PICI_2
NZ_CP012693.1_PICI_1
NZ_CP012693.1_PICI_2
NZ_CP018237.1_PICI_1

NZ_CP018245.1_PICI_1
NZ_CP018245.1_PICI_2
NZ_CP018243.1_PICI_1
unknown_PICI_2
unknown_PICI_3
NZ_CP018247.1_PICI_1
NZ_CP018241.1_PICI_1
NZ_CP018250.1_PICI_1
NZ_CP010122.1_PICI_1
NZ_CP019778.1_PICI_1
NZ_CP020106.1_PICI_1
NZ_CP021335.1_PICI_1
NZ_CP021335.1_PICI_2
NZ_CP019560.1_PICI_1
NZ_CP034966.1_PICI_1
NZ_CP021288.1_PICI_1
NZ_CP023349.1_PICI_1
NZ_CP024830.1_PICI_1
CP024815.1_PICI_1
CP024815.1_PICI_2
CP024815.1_PICI_3
CP024815.1_PICI_4
NZ_CP024815.1_PICI_1
CP024815.1_PICI_5
NZ_CP024134.1_PICI_1
NZ_CP023673.1_PICI_1
NZ_CP026755.1_PICI_1
NZ_CP019273.1_PICI_1
NZ_CP019243.1_PICI_1
NZ_CP025318.1_PICI_1
NZ_CP027219.1_PICI_1
NZ_CP027221.1_PICI_1
NZ_CP027307.1_PICI_1
NZ_CP027310.1_PICI_1
NZ_CP027338.1_PICI_1
NZ_CP027338.1_PICI_2

NZ_CP027340.1_PICI_1
NZ_CP027347.1_PICI_1
NZ_CP027390.1_PICI_1
NZ_CP027390.1_PICI_2
NZ_CP027442.1_PICI_1
NZ_CP027442.1_PICI_2
NZ_CP027452.1_PICI_1
NZ_CP027472.1_PICI_1
NZ_CP027548.1_PICI_1
NZ_CP027548.1_PICI_2
NZ_CP027371.1_PICI_1
NZ_CP027371.1_PICI_2
NZ_CP027437.1_PICI_1
NZ_CP027449.1_PICI_1
NZ_CP027555.1_PICI_1
NZ_CP027579.1_PICI_1
NZ_CP027599.1_PICI_1
NZ_CP027599.1_PICI_2
NZ_CP027573.1_PICI_1
NZ_CP027577.1_PICI_1
NZ_CP027582.1_PICI_1
NZ_CP027312.1_PICI_1
NZ_CP027331.1_PICI_1
NZ_CP027331.1_PICI_2
NZ_CP027342.1_PICI_1
NZ_CP027342.1_PICI_2
NZ_CP027352.1_PICI_1
NZ_CP027352.1_PICI_2
NZ_CP027373.1_PICI_1
NZ_CP027380.1_PICI_1
NZ_CP027387.1_PICI_1
NZ_CP027388.1_PICI_1
NZ_CP027362.1_PICI_1
NZ_CP027544.1_PICI_1
NZ_CP027544.1_PICI_2
NZ_CP027546.1_PICI_1

NZ_CP027546.1_PICI_2
NZ_CP028192.1_PICI_1
NZ_CP028381.1_PICI_1
NZ_CP029692.1_PICI_1
NZ_CP029741.1_PICI_1
NZ_CP028432.1_PICI_1
NZ_CP030767.1_PICI_1
NZ_CP030939.1_PICI_1
CP031134.1_PICI_1
NZ_CP031256.1_PICI_1
NZ_CP031546.1_PICI_1
NZ_CP033635.1_PICI_1
NZ_CP033635.1_PICI_2
NZ_CP025903.1_PICI_1
NZ_CP025859.1_PICI_1
NZ_AP018802.1_PICI_1
NZ_AP018808.1_PICI_1
NZ_AP018808.1_PICI_2
NZ_CP034792.1_PICI_1
NZ_CP034794.1_PICI_1
NZ_CP034799.1_PICI_1
NZ_CP034801.1_PICI_1
NZ_CP034808.1_PICI_1
NZ_CP035486.1_PICI_1
NZ_CP037945.1_PICI_1
NZ_CP037945.1_PICI_2
NZ_CP037941.1_PICI_1
NZ_CP037941.1_PICI_2
NZ_CP037943.1_PICI_1
NZ_CP038505.1_PICI_1
NZ_CP028592.1_PICI_1
NZ_CP028596.1_PICI_1
NZ_CP028598.1_PICI_1
NZ_CP028596.1_PICI_2
NZ_CP028603.1_PICI_1
NZ_CP028614.1_PICI_1

NZ_CP028626.1_PICI_1
NZ_CP028623.1_PICI_1
NZ_CP028632.1_PICI_1
NZ_CP028635.1_PICI_1
NZ_CP028620.1_PICI_1
NZ_CP028635.1_PICI_2
NZ_CP028647.1_PICI_1
NZ_CP028623.1_PICI_2
NZ_CP028668.1_PICI_1
NZ_CP028674.1_PICI_1
NZ_CP028671.1_PICI_1
NZ_CP028617.1_PICI_1
NZ_CP028620.1_PICI_2
NZ_CP028623.1_PICI_3
NZ_CP028644.1_PICI_1
NZ_CP028623.1_PICI_4
NZ_CP028662.1_PICI_1
NZ_CP028662.1_PICI_2
NZ_CP028677.1_PICI_1
NZ_CP028680.1_PICI_1
NZ_CP040390.1_PICI_1
NZ_CP040919.1_PICI_1
NZ_CP041304.1_PICI_1
NZ_CP015853.1_PICI_1
NZ_CP044311.1_PICI_1
NZ_CP044313.1_PICI_1
NZ_CP044314.1_PICI_1
NZ_CP044315.1_PICI_1
NZ_CP032145.1_PICI_1
NZ_CP032789.1_PICI_1
NZ_CP032791.1_PICI_1
NZ_CP032793.1_PICI_1
NZ_CP032795.1_PICI_1
NZ_CP032795.1_PICI_2
NZ_CP032801.1_PICI_1
NZ_CP032801.1_PICI_2

NZ_CP032803.1_PICI_1
NZ_CP032811.1_PICI_1
NZ_CP032811.1_PICI_2
NZ_CP040305.1_PICI_1
NZ_CP040307.1_PICI_1
NZ_CP040307.1_PICI_2
NZ_CP040309.1_PICI_1
NZ_CP040311.1_PICI_1
NZ_CP040313.1_PICI_1
NZ_CP040314.1_PICI_1
NZ_CP040314.1_PICI_2
NZ_CP040316.1_PICI_1
NZ_CP040316.1_PICI_2
NZ_CP045827.1_PICI_1
NZ_CP035545.1_PICI_1
NZ_CP045975.1_PICI_1
NZ_CP046009.1_PICI_1
NZ_CP046527.1_PICI_1
unknown_PICI_4
NZ_CP041431.1_PICI_1
NZ_CP041433.1_PICI_1
NZ_CP048304.1_PICI_1
NZ_CP048337.1_PICI_1
NZ_CP048934.1_PICI_1
NZ_CP050862.1_PICI_1
NZ_CP050862.1_PICI_2
NZ_CP050865.1_PICI_1
NZ_CP050865.1_PICI_2
NZ_CP051725.1_PICI_1
NZ_CP051714.1_PICI_1
NZ_CP052877.1_PICI_1
NZ_CP037449.1_PICI_1
NZ_CP053234.1_PICI_1
NZ_CP054363.1_PICI_1
NZ_AP023190.1_PICI_1
NZ_AP023197.1_PICI_1

NZ_CP050498.1_PICI_1
NZ_CP054236.1_PICI_1
NZ_CP058682.2_PICI_1
NZ_CP058682.2_PICI_2
NZ_AP022261.1_PICI_1
NZ_AP022287.1_PICI_1
NZ_AP022298.1_PICI_1
NZ_CP061206.1_PICI_1
NZ_CP024659.1_PICI_1
NZ_CP061337.1_PICI_1
NZ_CP061339.1_PICI_1
NZ_CP061914.1_PICI_1
NZ_CP062204.1_PICI_1
NZ_CP063046.1_PICI_1
NZ_CP063153.1_PICI_1
NZ_AP022533.1_PICI_1
NZ_CP063983.1_PICI_1
NZ_AP022811.1_PICI_1
NZ_AP022815.1_PICI_1
NZ_CP065607.1_PICI_1
NZ_CP065607.1_PICI_2
NZ_CP066144.1_PICI_1
NZ_CP061101.1_PICI_1
NZ_CP066806.1_PICI_1
NZ_AP024205.1_PICI_1
CP068035.1_PICI_1
NZ_CP068035.1_PICI_1
NZ_CP068035.1_PICI_2
NZ_CP068829.1_PICI_1
NZ_CP068804.1_PICI_1
NZ_CP068800.1_PICI_1
NZ_CP068810.1_PICI_1
NZ_CP068803.1_PICI_1
NZ_CP068796.1_PICI_1
NZ_CP069447.1_PICI_1
NZ_CP069500.1_PICI_1

NZ_CP069500.1_PICI_2
NZ_CP069517.1_PICI_1
NZ_CP069522.1_PICI_1
NZ_CP070041.1_PICI_1
NZ_CP070045.1_PICI_1
NZ_LT601384.1_PICI_1
NZ_LR134079.1_PICI_1
NZ_LR134079.1_PICI_2
NZ_LR134092.1_PICI_1
NZ_LR217818.1_PICI_1
NZ_LR882978.1_PICI_1
NZ_CP014314.1_PICI_1
NZ_CP015846.1_PICI_1
NZ_CP015846.1_PICI_2
NZ_CP015842.1_PICI_1
NZ_CP015842.1_PICI_2
NZ_CP015843.2_PICI_1
NZ_CP015843.2_PICI_2
NZ_CP016625.1_PICI_1
NZ_CP017434.1_PICI_1
NZ_CP017436.1_PICI_1
NZ_CP017438.1_PICI_1
NZ_CP017446.1_PICI_1
NZ_CP017442.1_PICI_1
NZ_CP033605.1_PICI_1
NZ_AP018488.1_PICI_1
NZ_AP018488.1_PICI_2
NZ_CP035366.1_PICI_1
NZ_CP040572.1_PICI_1
NZ_CP038282.1_PICI_1
NZ_CP038284.1_PICI_1
NZ_CP038287.1_PICI_1
NZ_CP038287.1_PICI_2
NZ_CP038290.1_PICI_1
NZ_CP038292.1_PICI_1
NZ_CP038295.1_PICI_1

NZ_CP038300.1_PICI_1
NZ_CP038302.1_PICI_1
NZ_CP038302.1_PICI_2
NZ_CP038305.1_PICI_1
NZ_CP038309.1_PICI_1
NZ_CP038316.1_PICI_1
NZ_CP038319.1_PICI_1
NZ_CP038328.1_PICI_1
NZ_CP038328.1_PICI_2
NZ_CP038333.1_PICI_1
NZ_CP038336.1_PICI_1
NZ_CP038339.1_PICI_1
NZ_CP038344.1_PICI_1
NZ_CP038346.1_PICI_1
NZ_CP038353.1_PICI_1
NZ_CP038360.1_PICI_1
NZ_CP038366.1_PICI_1
NZ_CP038369.1_PICI_1
NZ_CP038376.1_PICI_1
NZ_CP038380.1_PICI_1
NZ_CP038383.1_PICI_1
NZ_CP038389.1_PICI_1
NZ_CP038394.1_PICI_1
NZ_CP038394.1_PICI_2
NZ_CP038398.1_PICI_1
NZ_CP038402.1_PICI_1
NZ_CP038412.1_PICI_1
NZ_CP038414.1_PICI_1
NZ_CP038416.1_PICI_1
NZ_CP038419.1_PICI_1
NZ_CP038421.1_PICI_1
NZ_CP038423.1_PICI_1
NZ_CP039834.1_PICI_1
NZ_CP039834.1_PICI_2
NZ_CP039837.1_PICI_1
NZ_CP040107.1_PICI_1

NZ_CP066753.1_PICI_1
NZ_CP066748.1_PICI_1
NZ_CP062778.1_PICI_1
NZ_CP062700.1_PICI_1
NZ_CP062702.1_PICI_1
NZ_CP062705.1_PICI_1
NZ_CP062708.1_PICI_1
NZ_CP062711.1_PICI_1
NZ_CP062713.1_PICI_1
NZ_CP062715.1_PICI_1
NZ_CP062717.1_PICI_1
NZ_CP062719.1_PICI_1
NZ_CP062721.1_PICI_1
NZ_CP062723.1_PICI_1
NZ_CP062723.1_PICI_2
NZ_CP062725.1_PICI_1
NZ_CP062727.1_PICI_1
NZ_CP062729.1_PICI_1
NZ_CP062729.1_PICI_2
NZ_CP062780.1_PICI_1
NZ_CP062780.1_PICI_2
NZ_CP062731.1_PICI_1
NZ_CP062731.1_PICI_2
NZ_CP062733.1_PICI_1
NZ_CP062733.1_PICI_2
CP062739.1_PICI_1
NZ_CP062739.1_PICI_1
NZ_CP062739.1_PICI_2
NZ_CP062736.1_PICI_1
NZ_CP062736.1_PICI_2
NZ_CP062742.1_PICI_1
NZ_CP062744.1_PICI_1
NZ_CP062744.1_PICI_2
NZ_CP062746.1_PICI_1
NZ_CP062749.1_PICI_1
NZ_CP062752.1_PICI_1

NZ_CP062755.1_PICI_1
NZ_CP062761.1_PICI_1
NZ_CP062758.1_PICI_1
NZ_CP062763.1_PICI_1
NZ_CP062766.1_PICI_1
NZ_CP062782.1_PICI_1
NZ_CP062769.1_PICI_1
NZ_CP062771.1_PICI_1
NZ_CP062774.1_PICI_1
NZ_AP019761.1_PICI_1
NC_017626.1_PICI_1
NZ_CP031922.1_PICI_1
NZ_CP031922.1_PICI_2
CP007799.1_PICI_1
NZ_CP022686.1_PICI_1
NZ_CP035751.1_PICI_1
NZ_CP031912.1_PICI_1
NZ_CP031908.1_PICI_1
NC_011353.1_PICI_1
NC_013008.1_PICI_1
NC_013361.1_PICI_1
NC_013361.1_PICI_2
NC_013353.1_PICI_1
NC_013364.1_PICI_1
NC_013941.1_PICI_1
NC_017628.1_PICI_1
NC_017632.1_PICI_1
NC_017651.1_PICI_1
NC_017651.1_PICI_2
NZ_CP031919.1_PICI_1
NZ_CP015832.1_PICI_1
NZ_CP015831.1_PICI_1
unknown_PICI_5
NZ_CP022050.2_PICI_1
NZ_CP043539.1_PICI_1
NZ_CP044148.1_PICI_1

NZ_CP044148.1_PICI_2
NC_017656.1_PICI_1
NZ_CP028116.1_PICI_1
NZ_CP028116.1_PICI_2
NZ_CP028126.1_PICI_1
NZ_CP028126.1_PICI_2
NZ_CP031343.1_PICI_1
NZ_CP031355.1_PICI_1
NZ_CP035767.1_PICI_1
NZ_CP031341.1_PICI_1
NZ_CP035772.1_PICI_1
NZ_CP035770.1_PICI_1
NZ_CP035768.1_PICI_1
NZ_CP031350.1_PICI_1
NZ_CP031349.1_PICI_1
NZ_CP031345.1_PICI_1
NZ_CP028112.1_PICI_1
NZ_CP028117.1_PICI_1
NZ_CP028379.1_PICI_1
NZ_CP015244.1_PICI_1
NZ_CP031916.1_PICI_1
NZ_CP006027.1_PICI_1
NZ_CP006027.1_PICI_2
NZ_CP006262.1_PICI_1
NZ_CP006262.1_PICI_2
NZ_CP009072.1_PICI_1
NZ_CP008805.1_PICI_1
NZ_CP010304.1_PICI_1
NZ_CP023541.1_PICI_1
NZ_CP038355.1_PICI_1
NZ_CP038355.1_PICI_2
NZ_CP024240.1_PICI_1
NZ_CP028320.1_PICI_1
NZ_CP062855.1_PICI_1
NC_014624.2_PICI_1
NZ_CP014872.1_PICI_1

NC_008601.1_PICI_1
NZ_CP009633.1_PICI_1
NZ_CP017641.1_PICI_1
NZ_LN831027.1_PICI_1
NZ_CP024700.1_PICI_1
NZ_CP024731.1_PICI_1
NZ_CP024705.1_PICI_1
NZ_LR593886.1_PICI_1
NZ_CP043930.1_PICI_1
CP011853.1_PICI_1
NZ_CP011853.1_PICI_1
NC_016906.1_PICI_1
CP025435.1_PICI_1
NZ_CP046257.1_PICI_1
CP016594.1_PICI_1
CP029604.1_PICI_1
NZ_CP045875.1_PICI_1
NZ_CP025197.1_PICI_1
NC_022997.1_PICI_1
NZ_CP019433.1_PICI_1
NZ_CP012507.1_PICI_1
NZ_CP035103.1_PICI_1
NZ_CP015907.1_PICI_1
NZ_CP015899.1_PICI_1
NZ_CP015901.1_PICI_1
NZ_CP032430.1_PICI_1
NZ_CP032451.1_PICI_1
NZ_CP041280.1_PICI_1
NZ_CP029544.1_PICI_1
NC_008527.1_PICI_1
NC_009004.1_PICI_1
NC_017949.1_PICI_1
NZ_CP035223.1_PICI_1
NZ_CP035224.1_PICI_1
NZ_CP047628.1_PICI_1
NZ_CP047630.1_PICI_1

NC_018649.1_PICI_1
NZ_CP014886.1_PICI_1
NC_015520.1_PICI_1
NZ_CP065729.1_PICI_1
CP046531.1_PICI_1
NZ_CP065792.1_PICI_1
NZ_CP030092.1_PICI_1
NZ_CP017156.1_PICI_1
NZ_LT906462.1_PICI_1
NZ_CP027667.1_PICI_1
NZ_CP027228.1_PICI_1
NZ_CP014960.1_PICI_1
NZ_CP065266.1_PICI_1
NZ_CP065269.1_PICI_1
NZ_AP022565.1_PICI_1
NZ_AP022579.1_PICI_1
NZ_AP022591.1_PICI_1
NZ_AP022591.1_PICI_2
NZ_AP022604.1_PICI_1
NZ_LR134355.1_PICI_1
AP022612.1_PICI_1
NZ_CP020821.1_PICI_1
NZ_AP022613.1_PICI_1
CP020809.1_PICI_1
NZ_AP022603.1_PICI_1
NZ_CP011269.1_PICI_1
NZ_CP011269.1_PICI_2
AP022601.1_PICI_1
CP000656.1_PICI_1
NZ_LR026975.1_PICI_1
NZ_AP022609.1_PICI_1
NZ_AP024254.1_PICI_1
NZ_AP024255.1_PICI_1
NZ_AP024244.1_PICI_1
NC_016946.1_PICI_1
NZ_AP023287.1_PICI_1

NZ_AP022567.1_PICI_1
NZ_AP022590.1_PICI_1
NZ_AP022583.1_PICI_1
NZ_AP024257.1_PICI_1
NZ_AP024257.1_PICI_2
NZ_CP025546.1_PICI_1
NZ_AP022570.1_PICI_1
NZ_AP022574.1_PICI_1
NZ_AP022575.1_PICI_1
NZ_CP011773.1_PICI_1
NZ_CP015596.1_PICI_1
unknown_PICI_6
NZ_CP065373.1_PICI_1
NZ_CP015773.2_PICI_1
NZ_CP012095.1_PICI_1
NZ_CP027035.1_PICI_1
NZ_CP007809.1_PICI_1
NC_018143.2_PICI_1
NZ_CP009100.1_PICI_1
NZ_CP009101.1_PICI_1
NZ_CP009101.1_PICI_2
NZ_CP009426.1_PICI_1
NZ_CP009427.1_PICI_1
NZ_CP009427.1_PICI_2
NZ_CP012506.2_PICI_1
NZ_CP010330.1_PICI_1
NZ_CP010330.1_PICI_2
NZ_CP046529.1_PICI_1
NZ_CP046529.1_PICI_2
NZ_CP016794.1_PICI_1
NZ_CP016888.1_PICI_1
NZ_CP011510.1_PICI_1
NZ_CP013475.1_PICI_1
NZ_CP017920.1_PICI_1
NZ_CP018303.1_PICI_1
NZ_CP018305.1_PICI_1

NZ_CP018302.1_PICI_1
NZ_CP018301.1_PICI_1
NZ_CP018301.1_PICI_2
NZ_CP018300.1_PICI_1
NZ_CP018304.1_PICI_1
NZ_CP018778.1_PICI_1
NZ_CP020381.2_PICI_1
NZ_CP017593.1_PICI_1
NZ_CP017594.1_PICI_1
NZ_CP017595.1_PICI_1
NZ_CP017596.1_PICI_1
NZ_CP017597.1_PICI_1
NZ_CP017598.1_PICI_1
NZ_CP022014.1_PICI_1
NZ_AP017901.1_PICI_1
NZ_AP018034.1_PICI_1
NZ_AP018035.1_PICI_1
NZ_AP018036.1_PICI_1
NZ_CP023573.1_PICI_1
NZ_CP023573.1_PICI_2
NZ_CP023574.1_PICI_1
NZ_CP023574.1_PICI_2
NZ_CP023575.1_PICI_1
NZ_CP023575.1_PICI_2
NZ_CP023576.1_PICI_1
NZ_CP023576.1_PICI_2
NZ_CP023577.1_PICI_1
NZ_CP023577.1_PICI_2
NZ_CP023578.1_PICI_1
NZ_CP023578.1_PICI_2
NZ_CP023579.1_PICI_1
NZ_CP023579.1_PICI_2
NZ_CP023580.1_PICI_1
NZ_CP023580.1_PICI_2
NZ_CP023581.1_PICI_1
NZ_CP023581.1_PICI_2

NZ_CP023582.1_PICI_1
NZ_CP023582.1_PICI_2
NZ_CP023583.1_PICI_1
NZ_CP023583.1_PICI_2
NZ_CP023584.1_PICI_1
NZ_CP023584.1_PICI_2
NZ_CP023585.1_PICI_1
NZ_CP023585.1_PICI_2
NZ_CP023586.1_PICI_1
NZ_CP023586.1_PICI_2
NZ_CP023587.1_PICI_1
NZ_CP023587.1_PICI_2
NZ_CP023588.1_PICI_1
NZ_CP023588.1_PICI_2
NZ_CP023589.1_PICI_1
NZ_CP023589.1_PICI_2
NZ_CP023590.1_PICI_1
NZ_CP023590.1_PICI_2
NZ_CP023591.1_PICI_1
NZ_CP023591.1_PICI_2
NZ_CP023583.1_PICI_3
NZ_CP023592.1_PICI_1
NZ_CP023593.1_PICI_1
NZ_CP023593.1_PICI_2
NZ_CP023594.1_PICI_1
NZ_CP023594.1_PICI_2
NZ_CP023595.1_PICI_1
NZ_CP023595.1_PICI_2
NZ_CP023596.1_PICI_1
NZ_CP023596.1_PICI_2
NZ_CP023593.1_PICI_3
NZ_CP023597.1_PICI_1
NZ_CP023593.1_PICI_4
NZ_CP023597.1_PICI_2
NZ_CP023599.1_PICI_1
NZ_CP023599.1_PICI_2

NZ_CP023600.1_PICI_1
NZ_CP023600.1_PICI_2
NZ_CP023601.1_PICI_1
NZ_CP023601.1_PICI_2
NZ_CP023602.1_PICI_1
NZ_CP023602.1_PICI_2
NZ_CP023603.1_PICI_1
NZ_CP023603.1_PICI_2
NZ_CP023604.1_PICI_1
NZ_CP023604.1_PICI_2
NZ_CP023605.1_PICI_1
NZ_CP023602.1_PICI_3
NZ_CP023606.1_PICI_1
NZ_CP023606.1_PICI_2
NZ_CP023607.1_PICI_1
NZ_CP023607.1_PICI_2
NZ_CP023608.1_PICI_1
NZ_CP023608.1_PICI_2
NZ_CP023609.1_PICI_1
NZ_CP023609.1_PICI_2
NZ_CP023610.1_PICI_1
NZ_CP023610.1_PICI_2
NZ_CP023611.1_PICI_1
NZ_CP023611.1_PICI_2
NZ_CP023612.1_PICI_1
NZ_CP023612.1_PICI_2
NZ_CP023581.1_PICI_3
NZ_CP023613.1_PICI_1
NZ_CP023614.1_PICI_1
NZ_CP023594.1_PICI_3
NZ_CP023615.1_PICI_1
NZ_CP023615.1_PICI_2
NZ_CP023616.1_PICI_1
NZ_CP023616.1_PICI_2
NZ_CP023605.1_PICI_2
NZ_CP023617.1_PICI_1

NZ_CP023580.1_PICI_3
NZ_CP023580.1_PICI_4
NZ_CP023619.1_PICI_1
NZ_CP023619.1_PICI_2
NZ_CP023620.1_PICI_1
NZ_CP023620.1_PICI_2
NZ_CP023621.1_PICI_1
NZ_CP023621.1_PICI_2
NZ_CP023622.1_PICI_1
NZ_CP023622.1_PICI_2
NZ_CP023623.1_PICI_1
NZ_CP023623.1_PICI_2
NZ_CP023624.1_PICI_1
NZ_CP023624.1_PICI_2
NZ_CP023625.1_PICI_1
NZ_CP023625.1_PICI_2
NZ_CP023626.1_PICI_1
NZ_CP023626.1_PICI_2
NZ_CP023627.1_PICI_1
NZ_CP023627.1_PICI_2
NZ_CP023628.1_PICI_1
NZ_CP023628.1_PICI_2
NZ_CP023629.1_PICI_1
NZ_CP023629.1_PICI_2
NZ_CP023630.1_PICI_1
NZ_CP023630.1_PICI_2
NZ_CP023631.1_PICI_1
NZ_CP023631.1_PICI_2
NZ_CP023632.1_PICI_1
NZ_CP023632.1_PICI_2
NZ_CP023633.1_PICI_1
NZ_CP023620.1_PICI_3
NZ_CP023634.1_PICI_1
NZ_CP023634.1_PICI_2
NZ_CP023635.1_PICI_1
NZ_CP023635.1_PICI_2

NZ_CP023636.1_PICI_1
NZ_CP023628.1_PICI_3
NZ_CP023637.1_PICI_1
NZ_CP023637.1_PICI_2
NZ_CP023638.1_PICI_1
NZ_CP023638.1_PICI_2
NZ_CP023626.1_PICI_3
NZ_CP023639.1_PICI_1
NZ_CP023640.1_PICI_1
NZ_CP023597.1_PICI_3
NZ_CP018778.1_PICI_2
NZ_CP023637.1_PICI_3
NZ_CP025594.1_PICI_1
NZ_CP025594.1_PICI_2
NZ_CP025595.1_PICI_1
NZ_CP023602.1_PICI_4
NZ_CP025600.1_PICI_1
NZ_CP025600.1_PICI_2
NZ_CP025601.1_PICI_1
NZ_CP025601.1_PICI_2
NZ_CP025604.1_PICI_1
NZ_CP025604.1_PICI_2
NZ_CP025605.1_PICI_1
NZ_CP025605.1_PICI_2
NZ_CP025608.1_PICI_1
NZ_CP025608.1_PICI_2
NZ_CP025598.1_PICI_1
NZ_CP025598.1_PICI_2
NZ_CP025603.1_PICI_1
NZ_CP025603.1_PICI_2
NZ_CP025607.1_PICI_1
NZ_CP025607.1_PICI_2
NZ_CP023596.1_PICI_3
NZ_CP025596.1_PICI_1
NZ_CP025597.1_PICI_1
NZ_CP025597.1_PICI_2

NZ_CP025599.1_PICI_1
NZ_CP023597.1_PICI_4
NZ_CP025602.1_PICI_1
NZ_CP025602.1_PICI_2
NZ_CP025599.1_PICI_2
NZ_CP023619.1_PICI_3
NZ_CP022578.1_PICI_1
NZ_CP022577.1_PICI_1
NZ_CP030093.1_PICI_1
NZ_CP019610.1_PICI_1
NZ_CP019611.1_PICI_1
NZ_CP019611.1_PICI_2
NZ_CP019612.1_PICI_1
NZ_CP019613.1_PICI_1
NZ_CP029065.1_PICI_1
NZ_CP029065.1_PICI_2
NZ_CP041207.1_PICI_1
NZ_CP041207.1_PICI_2
NZ_CP044345.1_PICI_1
NZ_CP046308.1_PICI_1
NZ_CP046308.1_PICI_2
NZ_CP046309.1_PICI_1
NZ_CP046309.1_PICI_2
NZ_CP046728.2_PICI_1
NZ_CP048071.1_PICI_1
NZ_CP053092.1_PICI_1
NZ_CP054013.1_PICI_1
NZ_CP054013.1_PICI_2
NZ_CP054014.1_PICI_1
NZ_CP043995.1_PICI_1
NZ_CP043996.1_PICI_1
NZ_CP043997.1_PICI_1
NZ_CP041788.1_PICI_1
NZ_CP041789.1_PICI_1
NZ_CP041790.1_PICI_1
NZ_CP041792.1_PICI_1

NZ_CP041791.1_PICI_1
NZ_CP041793.1_PICI_1
NZ_CP041796.1_PICI_1
NZ_CP041797.1_PICI_1
NZ_CP041799.1_PICI_1
NZ_CP041800.1_PICI_1
NZ_CP041800.1_PICI_2
NZ_CP041801.1_PICI_1
NZ_CP041801.1_PICI_2
NZ_CP041804.1_PICI_1
NZ_CP041804.1_PICI_2
NZ_CP041805.1_PICI_1
NZ_CP041805.1_PICI_2
NZ_CP041806.1_PICI_1
NZ_CP041806.1_PICI_2
NZ_CP041807.1_PICI_1
NZ_CP041808.1_PICI_1
NZ_CP041809.1_PICI_1
NZ_CP041810.1_PICI_1
NZ_CP041811.1_PICI_1
NZ_CP041812.1_PICI_1
NZ_CP041813.1_PICI_1
NZ_CP041813.1_PICI_2
NZ_CP041814.1_PICI_1
NZ_CP041815.1_PICI_1
NZ_CP041816.1_PICI_1
NZ_CP041816.1_PICI_2
NZ_CP017596.1_PICI_2
NZ_CP041818.1_PICI_1
NZ_CP041818.1_PICI_2
NZ_CP041819.1_PICI_1
NZ_CP041820.1_PICI_1
NZ_CP041821.1_PICI_1
NZ_CP041822.1_PICI_1
NZ_CP041823.1_PICI_1
NZ_CP041824.1_PICI_1

NZ_CP041823.1_PICI_2
NZ_CP041826.1_PICI_1
NZ_CP041826.1_PICI_2
NZ_CP041827.1_PICI_1
NZ_CP041827.1_PICI_2
NZ_CP041828.1_PICI_1
NZ_CP041828.1_PICI_2
NZ_CP041829.1_PICI_1
NZ_CP041830.1_PICI_1
NZ_CP041831.1_PICI_1
NZ_CP041832.1_PICI_1
NZ_CP041833.1_PICI_1
NZ_CP041833.1_PICI_2
NZ_CP041834.1_PICI_1
NZ_CP041834.1_PICI_2
NZ_CP041835.1_PICI_1
NZ_CP041836.1_PICI_1
NZ_CP041837.1_PICI_1
NZ_CP041837.1_PICI_2
NZ_CP041838.1_PICI_1
NZ_CP041838.1_PICI_2
NZ_CP041839.1_PICI_1
NZ_CP041839.1_PICI_2
NZ_CP041840.1_PICI_1
NZ_CP041840.1_PICI_2
NZ_CP041841.1_PICI_1
NZ_CP041841.1_PICI_2
NZ_CP041842.1_PICI_1
NZ_CP041843.1_PICI_1
NZ_CP041844.1_PICI_1
NZ_CP041844.1_PICI_2
NZ_CP041845.1_PICI_1
NZ_CP041845.1_PICI_2
NZ_CP041846.1_PICI_1
NZ_CP041847.1_PICI_1
NZ_CP041848.1_PICI_1

NZ_CP041849.1_PICI_1
NZ_CP041850.1_PICI_1
NZ_CP041851.1_PICI_1
NZ_CP041852.1_PICI_1
NZ_CP041853.1_PICI_1
NZ_CP041853.1_PICI_2
NZ_CP041854.1_PICI_1
NZ_CP041855.1_PICI_1
NZ_CP041856.1_PICI_1
NZ_CP041857.1_PICI_1
NZ_CP041858.1_PICI_1
NZ_CP041859.1_PICI_1
NZ_CP041860.1_PICI_1
NZ_CP041861.1_PICI_1
NZ_CP041862.1_PICI_1
NZ_CP041862.1_PICI_2
NZ_CP041863.1_PICI_1
NZ_CP041864.1_PICI_1
NZ_CP041865.1_PICI_1
NZ_CP041866.1_PICI_1
NZ_CP041867.1_PICI_1
NZ_CP041868.1_PICI_1
NZ_CP041869.1_PICI_1
NZ_CP041870.1_PICI_1
NZ_CP041871.1_PICI_1
NZ_CP041871.1_PICI_2
NZ_CP041872.1_PICI_1
NZ_CP041873.1_PICI_1
NZ_CP041873.1_PICI_2
NZ_CP041874.1_PICI_1
NZ_CP041875.1_PICI_1
NZ_CP041875.1_PICI_2
NZ_CP041876.1_PICI_1
NZ_CP070338.1_PICI_1
NZ_CP071127.1_PICI_1
NZ_CP009101.1_PICI_3

NZ_CP071128.1_PICI_1
NZ_CP071128.1_PICI_2
NZ_LR027516.1_PICI_1
NZ_LR027516.1_PICI_2
NC_002755.2_PICI_1
NC_000962.3_PICI_1
NC_000962.3_PICI_2
NC_018143.2_PICI_2
NC_018143.2_PICI_3
NC_009565.1_PICI_1
NC_022350.1_PICI_1
NC_022350.1_PICI_2
NC_009525.1_PICI_1
NC_009525.1_PICI_2
NZ_CP016972.1_PICI_1
NZ_CP016972.1_PICI_2
NC_021251.1_PICI_1
NC_017522.1_PICI_1
NZ_CP002885.1_PICI_1
NC_016768.1_PICI_1
NC_012943.1_PICI_1
NC_018078.1_PICI_1
NC_020559.1_PICI_1
NC_020559.1_PICI_2
NZ_CP012090.1_PICI_1
NC_017524.1_PICI_1
NZ_CP002871.1_PICI_1
NZ_CP002882.1_PICI_1
NZ_CP002883.1_PICI_1
NC_020089.1_PICI_1
NC_020089.1_PICI_2
NZ_CP007803.1_PICI_1
NC_021740.1_PICI_1
NC_021740.1_PICI_2
NZ_HG813240.1_PICI_1
NZ_CP007027.1_PICI_1

NZ_CP007027.1_PICI_2
NZ_AP014573.1_PICI_1
NZ_AP014573.1_PICI_2
NC_008726.1_PICI_1
NC_007964.1_PICI_1
NC_007964.1_PICI_2
NZ_LT828648.1_PICI_1
NZ_CP042301.2_PICI_1
NZ_CP038033.1_PICI_1
NC_006361.1_PICI_1
NZ_CP041695.1_PICI_1
NC_014117.1_PICI_1
NZ_CP015958.1_PICI_1
NZ_CP045072.1_PICI_1
NZ_CP045073.1_PICI_1
NZ_CP041745.1_PICI_1
NZ_CP014167.1_PICI_1
NZ_CP012277.1_PICI_1
NZ_CP012289.1_PICI_1
NZ_CP065915.1_PICI_1
NZ_CP017113.1_PICI_1
NC_007948.1_PICI_1
NC_014215.1_PICI_1
NZ_CP045553.1_PICI_1
NZ_CP024712.1_PICI_1
NZ_CP069306.1_PICI_1
NZ_CP024899.1_PICI_1
NZ_CP044284.1_PICI_1
NZ_CP050124.1_PICI_1
NZ_CP017241.1_PICI_1
NZ_CP063450.1_PICI_1
NC_023150.1_PICI_1
NZ_CP035503.1_PICI_1
NZ_CP032762.1_PICI_1
NZ_CP018063.1_PICI_1
NZ_CP013511.1_PICI_1

NZ_CP013511.1_PICI_2
NZ_CP012749.1_PICI_1
NZ_CP064065.1_PICI_1
NZ_CP034766.1_PICI_1
NZ_CP069535.1_PICI_1
NZ_CP023510.1_PICI_1
NZ_CP030180.1_PICI_1
NZ_CP061771.1_PICI_1
NZ_CP018917.1_PICI_1
NZ_CP011511.1_PICI_1
NZ_CP026766.1_PICI_1
NZ_CP049285.1_PICI_1
NZ_CP049285.1_PICI_2
NZ_CP068090.1_PICI_1
NC_007613.1_PICI_1
unknown_PICI_7
NZ_CP021793.1_PICI_1
NZ_CP021808.1_PICI_1
CP021800.1_PICI_1
NZ_CP065020.1_PICI_1
NC_015590.1_PICI_1
NC_018700.1_PICI_1
NZ_CP021219.1_PICI_1
NZ_CP020105.1_PICI_1
CP060122.1_PICI_1
NZ_CP031266.1_PICI_1
NZ_AP019698.1_PICI_1
NZ_CP025023.1_PICI_1
NZ_CP023076.1_PICI_1
NZ_CP042286.1_PICI_1
NC_021670.1_PICI_1
NZ_CP007539.2_PICI_1
NZ_CP007499.1_PICI_1
NZ_CP009361.1_PICI_1
NZ_CP009361.1_PICI_2
NZ_CP009554.1_PICI_1

NZ_LN626917.1_PICI_1
NZ_CP026960.1_PICI_1
NZ_CP026071.1_PICI_1
NZ_CP026079.1_PICI_1
NZ_CP026069.1_PICI_1
NZ_CP026069.1_PICI_2
NZ_CP026072.1_PICI_1
NZ_CP026958.1_PICI_1
NZ_CP026958.1_PICI_2
NZ_CP026074.1_PICI_1
NZ_CP007670.1_PICI_1
NZ_CP012593.1_PICI_1
NZ_CP012593.1_PICI_2
NZ_CP010890.1_PICI_1
NZ_CP012756.1_PICI_1
NZ_CP012756.1_PICI_2
NZ_CP013137.1_PICI_1
NZ_CP009828.1_PICI_1
NZ_CP012015.1_PICI_1
NZ_CP012013.1_PICI_1
NZ_CP012018.1_PICI_1
NZ_CP012012.1_PICI_1
NZ_AP014942.1_PICI_1
NZ_CP011685.1_PICI_1
NZ_CP011685.1_PICI_2
NZ_CP013953.1_PICI_1
NZ_CP013953.1_PICI_2
NZ_CP013955.1_PICI_1
NZ_CP013955.1_PICI_2
NZ_CP012692.1_PICI_1
NZ_CP013182.1_PICI_1
NZ_CP020020.1_PICI_1
NZ_CP015447.2_PICI_1
NZ_CP009361.1_PICI_3
NZ_CP021905.1_PICI_1
NZ_CP009361.1_PICI_4

NZ_CP021905.1_PICI_2
NZ_AP014921.1_PICI_1
NZ_AP014921.1_PICI_2
NZ_CP023500.1_PICI_1
NZ_CP020741.1_PICI_1
NZ_CP017684.1_PICI_1
NZ_CP017684.1_PICI_2
NZ_CP017682.1_PICI_1
NZ_CP017679.1_PICI_1
NZ_CP017677.1_PICI_1
NZ_CP018629.1_PICI_1
NZ_CP020656.1_PICI_1
NZ_CP020354.1_PICI_1
NZ_CP028165.1_PICI_1
NZ_CP023561.1_PICI_1
NZ_CP029166.1_PICI_1
NZ_CP029166.1_PICI_2
NZ_CP028468.1_PICI_1
NZ_CP028468.1_PICI_2
NZ_CP029629.1_PICI_1
NZ_CP029627.1_PICI_1
NZ_CP029649.1_PICI_1
NZ_CP029675.1_PICI_1
NZ_CP029675.1_PICI_2
NZ_CP029681.1_PICI_1
NZ_CP029671.1_PICI_1
NZ_CP029685.1_PICI_1
NZ_CP027788.1_PICI_1
NZ_CP030323.1_PICI_1
NZ_CP022582.1_PICI_1
NZ_CP022903.1_PICI_1
NZ_CP022903.1_PICI_2
NZ_CP022903.1_PICI_3
NZ_CP022903.1_PICI_4
NZ_CP022900.1_PICI_1
NZ_CP022900.1_PICI_2

NZ_CP009828.1_PICI_2
NZ_CP022893.1_PICI_1
NZ_CP022893.1_PICI_2
NZ_CP022899.1_PICI_1
NZ_CP022899.1_PICI_2
NZ_CP022900.1_PICI_3
NZ_CP022901.1_PICI_1
NZ_CP022893.1_PICI_3
NZ_CP022894.1_PICI_1
NZ_CP022892.1_PICI_1
NZ_CP022910.1_PICI_1
NZ_CP022908.1_PICI_1
NZ_CP022906.1_PICI_1
NZ_CP022905.1_PICI_1
NZ_CP022905.1_PICI_2
NZ_CP031537.1_PICI_1
NZ_CP031537.1_PICI_2
NZ_CP031537.1_PICI_3
NZ_CP031661.1_PICI_1
NZ_CP031661.1_PICI_2
NZ_CP031673.1_PICI_1
NZ_AP017891.1_PICI_1
NZ_AP018349.1_PICI_1
NZ_CP032481.1_PICI_1
NZ_CP033865.1_PICI_1
NZ_CP034098.1_PICI_1
NZ_CP034349.1_PICI_1
NZ_AP019305.1_PICI_1
NZ_AP019305.1_PICI_2
NZ_AP019306.1_PICI_1
NZ_AP019306.1_PICI_2
NZ_CP034486.1_PICI_1
NZ_CP029474.1_PICI_1
NZ_CP030138.1_PICI_1
NZ_CP038021.1_PICI_1
NZ_CP032481.1_PICI_2

NZ_CP038269.1_PICI_1
NZ_CP038268.1_PICI_1
NZ_CP039156.1_PICI_1
NZ_CP038819.1_PICI_1
CP039157.1_PICI_1
unknown_PICI_8
NZ_CP039167.1_PICI_1
NZ_CP039992.1_PICI_1
NZ_CP040622.1_PICI_1
NZ_CP040622.1_PICI_2
NZ_CP029198.1_PICI_1
NZ_CP029198.1_PICI_2
NZ_CP040801.1_PICI_1
NZ_CP041010.1_PICI_1
NZ_AP019751.1_PICI_1
NZ_CP040622.1_PICI_3
NZ_CP040622.1_PICI_4
NZ_CP042043.1_PICI_1
NZ_CP042153.1_PICI_1
NZ_CP042153.1_PICI_2
NZ_CP042107.1_PICI_1
NZ_CP042157.1_PICI_1
NZ_CP042157.1_PICI_2
NZ_CP031265.1_PICI_1
NZ_CP041037.1_PICI_1
NZ_CP042650.1_PICI_1
NZ_CP039448.1_PICI_1
NZ_CP039448.1_PICI_2
NZ_CP043302.1_PICI_1
NZ_AP020324.1_PICI_1
NZ_AP020311.1_PICI_1
NZ_AP020311.1_PICI_2
NZ_AP020318.1_PICI_1
NZ_AP020320.1_PICI_1
NZ_AP020322.1_PICI_1
NZ_CP044106.1_PICI_1

NZ_AP019542.1_PICI_1
NZ_AP019542.1_PICI_2
NZ_AP019543.1_PICI_1
NZ_AP019543.1_PICI_2
NZ_AP019545.1_PICI_1
NZ_AP019545.1_PICI_2
NZ_CP045866.1_PICI_1
NZ_CP045472.1_PICI_1
NZ_CP033112.1_PICI_1
NZ_CP033112.1_PICI_2
NZ_CP033114.1_PICI_1
NZ_CP033112.1_PICI_3
NZ_AP019712.1_PICI_1
NZ_CP033977.1_PICI_1
NZ_CP033977.1_PICI_2
NZ_CP033977.1_PICI_3
NZ_CP047321.1_PICI_1
NZ_CP047321.1_PICI_2
NZ_CP047321.1_PICI_3
NZ_CP047778.1_PICI_1
NZ_CP047778.1_PICI_2
NZ_CP047805.1_PICI_1
NZ_CP047805.1_PICI_2
NZ_CP047779.1_PICI_1
NZ_CP047783.1_PICI_1
NZ_CP047781.1_PICI_1
NZ_CP047781.1_PICI_2
NZ_CP047782.1_PICI_1
NZ_CP047782.1_PICI_2
NZ_CP047786.1_PICI_1
NZ_CP047788.1_PICI_1
NZ_CP047793.1_PICI_1
NZ_CP047793.1_PICI_2
NZ_CP047791.1_PICI_1
NZ_CP047791.1_PICI_2
NZ_CP047801.1_PICI_1

NZ_CP047801.1_PICI_2
NZ_CP047798.1_PICI_1
NZ_CP047852.1_PICI_1
NZ_CP047799.1_PICI_1
NZ_CP047851.1_PICI_1
NZ_CP047809.1_PICI_1
NZ_CP047809.1_PICI_2
NZ_CP048643.1_PICI_1
NZ_CP048643.1_PICI_2
NZ_CP053070.1_PICI_1
NZ_CP053075.1_PICI_1
NZ_CP053185.1_PICI_1
NZ_CP053634.1_PICI_1
NZ_CP053636.1_PICI_1
NZ_CP045435.1_PICI_1
NZ_CP059156.1_PICI_1
NZ_CP059156.1_PICI_2
NZ_CP059155.1_PICI_1
NZ_CP059155.1_PICI_2
NZ_CP058615.1_PICI_1
NZ_CP058615.1_PICI_2
NZ_CP058615.1_PICI_3
NZ_CP058613.1_PICI_1
NZ_CP058613.1_PICI_2
NZ_CP058613.1_PICI_3
NZ_CP062469.1_PICI_1
NZ_CP062467.1_PICI_1
NZ_CP062465.1_PICI_1
NZ_CP062471.1_PICI_1
NZ_CP060584.1_PICI_1
NZ_CP060584.1_PICI_2
NZ_CP066488.1_PICI_1
NZ_CP066488.1_PICI_2
NZ_CP066492.1_PICI_1
NZ_CP066492.1_PICI_2
NZ_CP060491.1_PICI_1

NZ_CP060491.1_PICI_2
NZ_AP024170.1_PICI_1
NZ_CP069799.1_PICI_1
NZ_LS483301.1_PICI_1
NZ_LS483301.1_PICI_2
NZ_LS483300.1_PICI_1
NZ_LS483309.1_PICI_1
NZ_LS483314.1_PICI_1
NZ_LS483317.1_PICI_1
NZ_LS483311.1_PICI_1
NZ_LS483324.1_PICI_1
NZ_LS483350.1_PICI_1
NZ_LS483484.1_PICI_1
NZ_LR027876.1_PICI_1
NZ_LR027876.1_PICI_2
NZ_LR027870.1_PICI_1
NZ_LR027878.1_PICI_1
NZ_LR027873.1_PICI_1
NZ_LR027873.1_PICI_2
NZ_LR027877.1_PICI_1
NZ_LR027877.1_PICI_2
NZ_LR027869.1_PICI_1
NZ_LR027869.1_PICI_2
NZ_LR027874.1_PICI_1
NZ_LR130509.1_PICI_1
NZ_LR130511.1_PICI_1
NZ_LR130515.1_PICI_1
NZ_LR133917.1_PICI_1
NZ_LR134087.1_PICI_1
NZ_LR134093.1_PICI_1
NZ_LR134093.1_PICI_2
NZ_LR134139.1_PICI_1
NZ_LR134351.1_PICI_1
NZ_LR134351.1_PICI_2
NZ_LR822060.1_PICI_1
NZ_LR822060.1_PICI_2

NZ_LR822060.1_PICI_3
NZ_LR822061.1_PICI_1
NZ_LR822061.1_PICI_2
NZ_LR822061.1_PICI_3
NC_002951.2_PICI_1
NC_002758.2_PICI_1
NC_002758.2_PICI_2
NC_002745.2_PICI_1
NC_007622.1_PICI_1
NC_002952.2_PICI_1
NC_009782.1_PICI_1
NC_009782.1_PICI_2
NC_017341.1_PICI_1
NC_017341.1_PICI_2
NC_017342.1_PICI_1
NC_022113.1_PICI_1
NC_017331.1_PICI_1
NC_013450.1_PICI_1
NZ_CP025395.1_PICI_1
NC_017349.1_PICI_1
NC_017347.1_PICI_1
NC_017347.1_PICI_2
NC_016928.2_PICI_1
NC_022443.1_PICI_1
NC_022442.1_PICI_1
NZ_CP010526.1_PICI_1
NZ_CP006630.1_PICI_1
NZ_CP012409.1_PICI_1
NC_022222.1_PICI_1
NC_022604.1_PICI_1
NZ_CP065711.1_PICI_1
NC_012121.1_PICI_1
NZ_AP018586.1_PICI_1
NZ_AP018586.1_PICI_2
NZ_AP018587.1_PICI_1
NZ_CP031271.1_PICI_1

NZ_CP051643.1_PICI_1
NZ_CP053957.1_PICI_1
NZ_CP042341.1_PICI_1
NZ_CP031274.1_PICI_1
NZ_CP023694.1_PICI_1
NZ_CP027422.1_PICI_1
NZ_CP033735.1_PICI_1
NZ_CP033735.1_PICI_2
NZ_CP018776.1_PICI_1
NZ_CP033460.1_PICI_1
NZ_CP018842.1_PICI_1
NZ_CP022247.1_PICI_1
NZ_CP013943.1_PICI_1
NZ_CP030246.1_PICI_1
NZ_CP033782.1_PICI_1
NZ_CP034115.1_PICI_1
NZ_CP035643.1_PICI_1
NZ_CP060528.1_PICI_1
NZ_CP060794.1_PICI_1
NZ_CP061029.1_PICI_1
NZ_CP066303.1_PICI_1
NZ_CP069215.1_PICI_1
NZ_CP069473.1_PICI_1
NZ_CP069951.1_PICI_1
NZ_CP069954.1_PICI_1
NZ_CP070057.1_PICI_1
NZ_LT571449.1_PICI_1
NZ_LR134536.1_PICI_1
NZ_LR735429.1_PICI_1
NZ_CP068069.1_PICI_1
CP063443.1_PICI_1
NC_007168.1_PICI_1
NZ_CP033732.1_PICI_1
NZ_CP046301.1_PICI_1
NZ_CP054006.1_PICI_1
NZ_CP020618.1_PICI_1

NZ_CP014022.1_PICI_1
NZ_CP014022.1_PICI_2
NZ_CP017069.2_PICI_1
NZ_CP017069.2_PICI_2
NZ_CP023539.1_PICI_1
NZ_CP023539.1_PICI_2
NZ_CP023970.1_PICI_1
NZ_CP023970.1_PICI_2
NZ_CP041726.1_PICI_1
NZ_CP041726.1_PICI_2
NZ_CP041722.1_PICI_1
NZ_CP041722.1_PICI_2
NZ_CP038807.1_PICI_1
NZ_CP038807.1_PICI_2
NZ_AP021848.1_PICI_1
NZ_AP021848.1_PICI_2
NZ_CP063143.1_PICI_1
NZ_CP060160.1_PICI_1
NZ_CP060160.1_PICI_2
NZ_LS483312.1_PICI_1
NC_013893.1_PICI_1
NC_014217.1_PICI_1
NZ_CP017463.1_PICI_1
NC_022737.1_PICI_1
NZ_CP009913.1_PICI_1
NZ_CP014144.1_PICI_1
NZ_LR134274.1_PICI_1
NZ_CP014057.2_PICI_1
NZ_CP022056.1_PICI_1
NZ_CP022093.1_PICI_1
NZ_CP054444.1_PICI_1
NZ_CP054440.1_PICI_1
NZ_CP054575.1_PICI_1
NZ_CP054575.1_PICI_2
NZ_CP054575.1_PICI_3
NZ_CP054831.1_PICI_1

NC_007350.1_PICI_1
NZ_CP066042.1_PICI_1
NZ_CP017428.1_PICI_1
NZ_CP017428.1_PICI_2
NZ_CP015642.1_PICI_1
NZ_CP015642.1_PICI_2
NZ_CP022881.1_PICI_1
NZ_CP022881.1_PICI_2
NZ_CP048834.1_PICI_1
CP054916.1_PICI_1
NZ_CP068241.1_PICI_1
NZ_CP070306.1_PICI_1
NZ_CP070305.1_PICI_1
NZ_CP070964.1_PICI_1
NZ_CP043959.1_PICI_1
NZ_CP032159.1_PICI_1
NZ_CP033098.1_PICI_1
NZ_CP038242.1_PICI_1
NZ_CP053477.1_PICI_1
NZ_CP054017.1_PICI_1
NZ_CP061041.1_PICI_1
NZ_LR134242.1_PICI_1
NZ_LR134244.1_PICI_1
NZ_CP042806.1_PICI_1
NZ_CP020046.1_PICI_1
CP037895.1_PICI_1
NC_013889.1_PICI_1
NZ_CP011367.1_PICI_1
NZ_CP035282.1_PICI_1
NC_015577.1_PICI_1
NZ_CP063212.1_PICI_1
LR586016.1_PICI_1
NZ_LR594659.1_PICI_1
CP000781.1_PICI_1

Supplementary Table 7. Representatives of the major classes of helper-satellite systems described in the literature, included in the phylogen

Satellite_name	Satellite_accession	Satellite_file_name	Satellite_notes	Reference	Host
SaPI1	U93688	U93688.gb	-	https://doi.org/10.1046/j.1365-2958.[illegible]	S. aureus RN4282
P4	X51522	X51522.gb	-	https://doi.org/10.1016/0042-6822(7	E. coli
PLE1	KC152960	KC152960.gb	-	https://doi.org/10.1038/nature11927	V. cholerae
EcCICFT073	-	EcCICFT073.gb	Slice from AE01407[illegible]	https://doi.org/10.1038/s41396-018-[illegible]	E. coli CFT073

etic analyses.

Host_accession	Helper_name	Helper_accession	Helper_file_name
-	Staphylococcus phage 80alpha	NC_009526.1	NC_009526.gb
-	Enterobacteria phage P2	NC_001895.1	NC_001895.gb
-	Vibrio phage ICP1	NC_015157.1	NC_015157.gb
AE014075	Enterobacteria phage lambda	NC_001416.1	NC_001416.gb

Supplementary Table 8. Results from the tBLASTn search querying the NCBI GenBank database, restricted to bacteria (taxonomy ID: 2), with the pro

Query protein	Hit genome accession	Start position of the alignment	End position of the alignment	Phage name	E_value	Q_cov
SEA_MINIFLAYER_1	CP012749	5908550	5908978	Rhodococcus sp.	1.79E-13	0.922077922
SEA_MINIFLAYER_1	CP096563	3568697	3569074	Rhodococcus qin	4.30E-11	0.818181818
SEA_MINIFLAYER_2	CP015163	7814541	7816022	Amycolatopsis al	9.17E-107	0.988188976
SEA_MINIFLAYER_2	CP060131	5427318	5428727	Pseudonocardia	3.80E-101	0.938976378
SEA_MINIFLAYER_2	CP021748	1013295	1014794	Streptomyces alt	1.08E-100	0.984251969
SEA_MINIFLAYER_2	CP054925	2930854	2932323	Amycolatopsis sp	8.09E-99	0.978346457
SEA_MINIFLAYER_2	CP023689	1450846	1452330	Streptomyces ch	1.02E-98	0.992125984
SEA_MINIFLAYER_2	CP016793	655206	656738	Lentzea guizhoue	5.72E-98	0.988188976
SEA_MINIFLAYER_2	CP026652	7344659	7346161	Streptomyces de	8.86E-93	0.992125984
SEA_MINIFLAYER_2	CP050692	7076994	7078418	Streptomyces an	1.24E-90	0.962598425
SEA_MINIFLAYER_2	CP066831	9475685	9477163	Streptomyces lili	1.28E-90	0.986220472
SEA_MINIFLAYER_2	AP018920	2880442	2881833	Pseudonocardia	3.66E-83	0.94488189
SEA_MINIFLAYER_2	CP117709	1552230	1553642	Streptomyces sp.	2.15E-82	0.952755906
SEA_MINIFLAYER_2	CP015576	329155	330588	Campylobacter h	4.88E-26	0.93503937
SEA_MINIFLAYER_2	CP027287	354354	355793	Campylobacter fe	2.07E-25	0.938976378
SEA_MINIFLAYER_2	CP002221	824011	825339	Hydrogenobacte	1.41E-24	0.958661417
SEA_MINIFLAYER_2	AP011112	824020	825348	Hydrogenobacte	1.41E-24	0.958661417
SEA_MINIFLAYER_2	CP053841	882926	884335	Campylobacter b	2.48E-22	0.923228346
SEA_MINIFLAYER_2	CP040464	240213	241625	Campylobacter h	3.01E-21	0.92519685
SEA_MINIFLAYER_2	CP044173	1395592	1397028	Campylobacter je	1.30E-20	0.940944882
SEA_MINIFLAYER_2	CP022470	692805	694241	Campylobacter je	1.39E-20	0.940944882
SEA_MINIFLAYER_2	AP025981	1303536	1304972	Campylobacter je	1.39E-20	0.940944882
SEA_MINIFLAYER_2	AP025963	1347464	1348900	Campylobacter je	1.39E-20	0.940944882
SEA_MINIFLAYER_2	AP025957	445584	447020	Campylobacter je	1.39E-20	0.940944882
SEA_MINIFLAYER_2	AP025977	536107	537543	Campylobacter je	5.41E-20	0.940944882
SEA_MINIFLAYER_2	CP109817	1381847	1383283	Campylobacter c	1.14E-19	0.940944882
SEA_MINIFLAYER_2	CP109819	1381974	1383410	Campylobacter c	1.14E-19	0.940944882
SEA_MINIFLAYER_2	CP076503	335211	336647	Campylobacter c	1.21E-19	0.940944882
SEA_MINIFLAYER_2	CP041584	629856	631292	Campylobacter je	1.29E-19	0.940944882
SEA_MINIFLAYER_2	AP026005	1208028	1209464	Campylobacter je	1.33E-19	0.940944882
SEA_MINIFLAYER_2	AP026007	1208058	1209494	Campylobacter je	1.33E-19	0.940944882

SEA_MINIFLAYER_2	CP045048	1085104	1086540	Campylobacter je	1.33E-19	0.940944882
SEA_MINIFLAYER_2	AP025965	486319	487755	Campylobacter je	1.33E-19	0.940944882
SEA_MINIFLAYER_2	CP017868	1371641	1373077	Campylobacter c	1.58E-19	0.940944882
SEA_MINIFLAYER_2	CP017865	1396039	1397475	Campylobacter c	1.58E-19	0.940944882
SEA_MINIFLAYER_2	CP086657	563364	564800	Campylobacter c	1.58E-19	0.940944882
SEA_MINIFLAYER_2	CP017878	1396811	1398247	Campylobacter c	1.58E-19	0.940944882
SEA_MINIFLAYER_2	CP059360	869921	871357	Campylobacter c	1.58E-19	0.940944882
SEA_MINIFLAYER_2	CP040616	705278	706714	Campylobacter je	1.65E-19	0.940944882
SEA_MINIFLAYER_2	CP047480	313101	314537	Campylobacter je	1.65E-19	0.940944882
SEA_MINIFLAYER_2	CP040610	739312	740748	Campylobacter s	1.65E-19	0.940944882
SEA_MINIFLAYER_2	CP076509	771409	772845	Campylobacter c	1.85E-19	0.940944882
SEA_MINIFLAYER_2	CP068566	334768	336204	Campylobacter c	1.85E-19	0.940944882
SEA_MINIFLAYER_3	CP012749	5905173	5906435	Rhodococcus sp.	2.97E-81	0.912946429
SEA_MINIFLAYER_3	AP025544	2118897	2120219	Rhodococcus hoa	3.17E-80	0.975446429
SEA_MINIFLAYER_3	CP096563	3560891	3562210	Rhodococcus qin	4.07E-80	0.982142857
SEA_MINIFLAYER_3	CP021748	1011900	1013192	Streptomyces alb	5.48E-72	0.879464286
SEA_MINIFLAYER_3	CP021748	8044302	8045522	Streptomyces alb	1.21E-23	0.912946429
SEA_MINIFLAYER_3	CP026652	7346264	7347565	Streptomyces de	2.12E-70	0.879464286
SEA_MINIFLAYER_3	CP026652	4600286	4601599	Streptomyces de	3.77E-12	0.886160714
SEA_MINIFLAYER_3	CP117709	1550858	1552159	Streptomyces sp.	7.07E-69	0.908482143
SEA_MINIFLAYER_3	CP023689	1449458	1450729	Streptomyces cha	3.62E-66	0.879464286
SEA_MINIFLAYER_3	CP016793	656971	658323	Lentzea guizhoue	5.82E-62	0.892857143
SEA_MINIFLAYER_3	CP016793	2145656	2146912	Lentzea guizhoue	1.03E-14	0.90625
SEA_MINIFLAYER_3	CP066831	9477207	9478541	Streptomyces lilii	1.78E-58	0.930803571
SEA_MINIFLAYER_3	CP050692	7078552	7079928	Streptomyces an	5.78E-58	0.9375
SEA_MINIFLAYER_3	CP060131	5428867	5430156	Pseudonocardia	5.50E-52	0.886160714
SEA_MINIFLAYER_3	CP015163	7816057	7817376	Amycolatopsis al	8.52E-48	0.908482143
SEA_MINIFLAYER_3	CP015163	217473	218753	Amycolatopsis al	2.90E-12	0.919642857
SEA_MINIFLAYER_3	CP065000	2325562	2326779	Acidobacteria ba	1.22E-37	0.910714286
SEA_MINIFLAYER_3	CP054925	2929502	2930839	Amycolatopsis sp	3.24E-37	0.917410714
SEA_MINIFLAYER_3	CP107020	1843402	1844709	Brachybacterium	1.61E-30	0.970982143
SEA_MINIFLAYER_3	CP089608	3083089	3084390	Mycolicibacteriu	3.65E-29	0.939732143
SEA_MINIFLAYER_3	CP089608	162757	164058	Mycolicibacteriu	1.20E-26	0.939732143
SEA_MINIFLAYER_3	CP089608	5724066	5725319	Mycolicibacteriu	8.45E-20	0.899553571
SEA_MINIFLAYER_3	CP089608	5825489	5826673	Mycolicibacteriu	1.29E-15	0.897321429
SEA_MINIFLAYER_3	CP020809	6249925	6251199	Mycobacterium (	1.02E-28	0.919642857

SEA_MINIFLAYER_3	CP017150	3647534	3648772	Brevibacterium a	6.31E-28	0.935267857
SEA_MINIFLAYER_3	CP017150	2120045	2121283	Brevibacterium a	8.17E-28	0.935267857
SEA_MINIFLAYER_3	CP017150	3163711	3164949	Brevibacterium a	5.04E-27	0.935267857
SEA_MINIFLAYER_3	AP022595	4196298	4197527	Mycolicibacteriu	6.60E-28	0.886160714
SEA_MINIFLAYER_3	CP025332	3457882	3459120	Brevibacterium a	7.31E-28	0.935267857
SEA_MINIFLAYER_3	CP053189	1324694	1325788	Streptomyces sp.	5.38E-27	0.823660714
SEA_MINIFLAYER_3	CP095749	1231134	1232336	Streptomyces yu	8.85E-27	0.910714286
SEA_MINIFLAYER_3	CP095749	4863756	4865042	Streptomyces yu	2.75E-17	0.888392857
SEA_MINIFLAYER_3	CP118574	1353672	1354874	Streptomyces sp.	1.47E-26	0.910714286
SEA_MINIFLAYER_3	CP041616	1837484	1838686	Ornithinimicrobi	2.88E-26	0.915178571
SEA_MINIFLAYER_3	CP104302	177187	178434	Mycolicibacteriu	2.96E-26	0.901785714
SEA_MINIFLAYER_3	AP022575	209480	210712	Mycobacterium s	3.49E-26	0.892857143
SEA_MINIFLAYER_3	CP079869	4249029	4250270	Mycobacterium s	1.94E-25	0.897321429
SEA_MINIFLAYER_3	AP022574	3121421	3122710	Mycolicibacteriu	2.08E-25	0.955357143
SEA_MINIFLAYER_3	CP079865	6252899	6254134	Mycobacterium s	5.42E-25	0.899553571
SEA_MINIFLAYER_3	CP079865	4116463	4117617	Mycobacterium s	2.42E-20	0.850446429
SEA_MINIFLAYER_3	CP023435	2854814	2856109	Mycobacterium s	5.94E-25	0.930803571
SEA_MINIFLAYER_3	CP023435	3521021	3522295	Mycobacterium s	5.66E-12	0.9375
SEA_MINIFLAYER_3	AP022565	4616229	4617506	Mycolicibacteriu	9.23E-25	0.924107143
SEA_MINIFLAYER_3	AP023287	5117629	5118906	Mycolicibacteriu	1.15E-24	0.928571429
SEA_MINIFLAYER_3	CP016396	887922	889211	Mycobacterium a	2.68E-24	0.941964286
SEA_MINIFLAYER_3	CP089223	797626	798915	Mycobacterium a	2.68E-24	0.941964286
SEA_MINIFLAYER_3	CP046507	696664	697953	Mycobacterium a	2.68E-24	0.941964286
SEA_MINIFLAYER_3	CP085977	3922612	3923901	Mycobacterium a	2.68E-24	0.941964286
SEA_MINIFLAYER_3	CP085978	242892	244181	Mycobacterium a	2.68E-24	0.941964286
SEA_MINIFLAYER_5	CP096563	3559449	3560303	Rhodococcus qin	3.43E-63	0.966216216
SEA_MINIFLAYER_5	AP023172	3072262	3073125	Rhodococcus qin	1.85E-56	0.962837838
SEA_MINIFLAYER_5	AP025544	2120779	2121642	Rhodococcus hoa	6.72E-56	0.962837838
SEA_MINIFLAYER_5	CP117709	1549030	1549914	Streptomyces sp.	3.37E-36	0.945945946
SEA_MINIFLAYER_5	CP060131	5433968	5434882	Pseudonocardia	1.45E-29	0.962837838
SEA_MINIFLAYER_5	CP066831	9480787	9481701	Streptomyces lili	3.69E-28	0.962837838
SEA_MINIFLAYER_5	CP026652	7349793	7350632	Streptomyces de	2.31E-26	0.905405405
SEA_MINIFLAYER_5	CP023689	1446465	1447322	Streptomyces ch	2.49E-26	0.915540541
SEA_MINIFLAYER_5	CP021748	1008893	1009741	Streptomyces alb	7.50E-26	0.905405405
SEA_MINIFLAYER_5	CP050692	7081960	7082874	Streptomyces an	1.23E-25	0.962837838
SEA_MINIFLAYER_5	CP016793	667795	668694	Lentzea guizhoue	1.40E-25	0.945945946

SEA_MINIFLAYER_5	CP054925	2926492	2927379	Amycolatopsis sp	1.62E-22	0.935810811
SEA_MINIFLAYER_5	CP015163	7819474	7820397	Amycolatopsis al	3.83E-18	0.983108108
SEA_MINIFLAYER_6	CP028491	2397081	2397512	Carboxydocella t	1.85E-43	1
SEA_MINIFLAYER_6	AP026691	2367935	2368357	Rhodococcus qin	2.67E-41	0.986111111
SEA_MINIFLAYER_6	CP050124	2504317	2504739	Rhodococcus ery	3.93E-41	0.986111111
SEA_MINIFLAYER_6	CP050124	2545333	2545746	Rhodococcus ery	1.95E-37	0.965277778
SEA_MINIFLAYER_6	AP011115	4362320	4362742	Rhodococcus op	4.42E-41	0.986111111
SEA_MINIFLAYER_6	CP028514	2222024	2222425	Carboxydocella t	1.22E-40	0.9375
SEA_MINIFLAYER_6	CP027557	914980	915396	Rhodococcus rhc	3.04E-40	0.986111111
SEA_MINIFLAYER_6	CP070351	49704	50126	Gordonia sp. PDI	5.49E-40	1
SEA_MINIFLAYER_6	CP054207	3959322	3959741	Rhodococcus qin	3.06E-38	0.979166667
SEA_MINIFLAYER_6	CP000141	1499888	1500262	Carboxydotherm	6.33E-38	0.881944444
SEA_MINIFLAYER_6	CP065594	5206583	5207002	Prescottella equi	1.02E-35	0.979166667
SEA_MINIFLAYER_6	CP059900	3270927	3271358	Tomitella gaofuii	1.75E-34	0.986111111
SEA_MINIFLAYER_6	CP003347	3659139	3659546	Mycobacterium i	9.13E-33	0.958333333
SEA_MINIFLAYER_6	CP025435	2020869	2021279	Gordonia sp. YC-	1.62E-31	0.972222222
SEA_MINIFLAYER_6	AP025186	846964	847380	Rhodococcus sp.	5.63E-30	0.972222222
SEA_MINIFLAYER_6	CP000479	756417	756821	Mycobacterium a	2.22E-27	0.958333333
SEA_MINIFLAYER_6	CP036402	1560578	1560952	Egibacter rhizosp	2.47E-27	0.875
SEA_MINIFLAYER_6	CP096563	3558978	3559364	Rhodococcus qin	3.07E-25	0.930555556
SEA_MINIFLAYER_6	CP046171	6523899	6524315	Nocardia brasilie	2.37E-24	0.986111111
SEA_MINIFLAYER_6	CP078145	1217891	1218307	Nocardia iowens	1.38E-23	0.986111111
SEA_MINIFLAYER_6	CP082962	1827008	1827424	Desulfovibrio sp.	3.31E-23	0.986111111
SEA_MINIFLAYER_6	AP022606	5109693	5110106	Mycobacterium l	2.54E-21	0.986111111
SEA_MINIFLAYER_6	AP022606	23808	24221	Mycobacterium l	2.54E-21	0.986111111
SEA_MINIFLAYER_6	CP026538	4161176	4161535	Solidesulfovibrio	6.32E-19	0.854166667
SEA_MINIFLAYER_6	CP062008	5377798	5378157	Mycolicibacteriu	2.21E-18	0.854166667
SEA_MINIFLAYER_6	AP010904	474403	474816	Desulfovibrio ma	3.04E-18	0.979166667
SEA_MINIFLAYER_6	CP014646	1379520	1379909	Thauera humirec	6.22E-16	0.909722222
SEA_MINIFLAYER_6	CP045508	3856029	3856457	Desulfolutivibrio	1.30E-15	0.972222222
SEA_MINIFLAYER_6	CP045506	3972606	3973034	Desulfolutivibrio	1.30E-15	0.972222222
SEA_MINIFLAYER_6	CP101406	220837	221223	Mycobacterium l	3.84E-15	0.895833333
SEA_MINIFLAYER_6	CP045504	3125252	3125668	Desulfovibrio sul	4.14E-13	0.979166667
SEA_MINIFLAYER_6	CP045504	4188506	4188841	Desulfovibrio sul	9.30E-12	0.8125
SEA_MINIFLAYER_6	CP045504	877008	877343	Desulfovibrio sul	9.30E-12	0.8125
SEA_MINIFLAYER_6	CP099605	1715769	1716191	Comamonadacea	1.40E-11	0.972222222

SEA_MINIFLAYER_6	AP019700	1278648	1279034	Stella humosa AT	1.96E-11	0.916666667
SEA_MINIFLAYER_6	CP038440	2646012	2646413	Brevundimonas s	5.45E-11	0.958333333
SEA_MINIFLAYER_10	CP017299	3042821	3043162	Rhodococcus sp.	3.27E-12	0.907692308
SEA_MINIFLAYER_26	CP012749	5908550	5908978	Rhodococcus sp.	1.80E-13	0.922077922
SEA_MINIFLAYER_26	CP096563	3568697	3569074	Rhodococcus qin	4.31E-11	0.818181818

Supplementary Table 9. Results from the tBLASTn search querying the NCBI GenBank database, restricted to bacteria (taxonomy ID: 2), with the prote

Query protein	Hit genome accession	Start position of the alignment	End position of the alignment	Phage name	E_value	Q_cov
CDS_1	AP025544	2116365	2116724	Rhodococcus hoagii U19 DN	6.83E-20	0.77564103
CDS_3	AP025544	2118897	2120204	Rhodococcus hoagii U19 DN	5.91E-145	0.94408602
CDS_3	CP096563	3560939	3562210	Rhodococcus qingshengii JC	1.68E-141	0.91612903
CDS_3	CP012749	5905164	5906435	Rhodococcus sp. 008	3.39E-139	0.91612903
CDS_3	CP021748	1011894	1013237	Streptomyces alboflavus str	1.03E-96	0.9139785
CDS_3	CP026652	7346216	7347571	Streptomyces dengpaensis :	3.78E-96	0.91612903
CDS_3	CP060131	5428831	5430165	Pseudonocardia petroleoph	3.81E-85	0.91612903
CDS_3	CP117709	1550813	1552165	Streptomyces sp. MMBL 11-	2.60E-82	0.94193548
CDS_3	CP066831	9477204	9478517	Streptomyces liliifuscus stra	4.97E-79	0.91182796
CDS_3	CP023689	1449452	1450774	Streptomyces chartreusis st	1.41E-78	0.9139785
CDS_3	CP016793	656932	658323	Lentzea guizhouensis strain	2.05E-75	0.91827957
CDS_3	CP050692	7078567	7079904	Streptomyces antibioticus s	7.92E-72	0.91182796
CDS_3	CP015163	7816048	7817382	Amycolatopsis albispora str	1.71E-63	0.91612903
CDS_3	CP054925	2929499	2930839	Amycolatopsis sp. Hca4 chr	1.22E-62	0.91612903
CDS_3	CP065000	2325556	2326839	Acidobacteria bacterium isc	3.01E-48	0.9655914
CDS_3	CP030797	3553708	3555066	Brevibacterium linens strair	3.28E-24	0.91827957
CDS_3	CP012382	6597799	6598968	Streptomyces ambofaciens	7.96E-24	0.83870968
CDS_3	CP095749	1231158	1232390	Streptomyces yunnanensis :	1.22E-23	0.92258065
CDS_3	CP095749	4863810	4865045	Streptomyces yunnanensis :	2.88E-12	0.84946237
CDS_3	CP118574	1353696	1354928	Streptomyces sp. WZ-12 chi	1.53E-23	0.92258065
CDS_3	CP023702	6996681	6997802	Streptomyces nitrosporeus	4.97E-23	0.80430108
CDS_3	CP053189	1324562	1325833	Streptomyces sp. Jing01 chr	7.91E-23	0.94193548
CDS_3	AP022599	3245678	3246847	Mycolicibacterium pulveris .	1.03E-21	0.86236559
CDS_3	CP079869	4249035	4250297	Mycobacterium sp. SMC-4 c	4.14E-21	0.90752688
CDS_3	CP060716	1811517	1812680	Leucobacter denitrificans st	4.74E-21	0.87956989
CDS_3	CP021744	1384147	1385481	Streptomyces albireticuli str	1.75E-20	0.97849462
CDS_3	CP092981	5365595	5366881	Mycobacterium marinum st	1.06E-19	0.92473118
CDS_3	HE572590	1187129	1188427	Mycobacterium canettii CIP	1.55E-19	0.93333333
CDS_3	HE572590	4138671	4139972	Mycobacterium canettii CIP	3.05E-18	0.93333333
CDS_3	FO203509	1052540	1053838	Mycobacterium canettii CIP	1.64E-19	0.93333333
CDS_3	AP022562	207630	208961	Mycobacterium novum JCM	3.16E-19	0.98064516
CDS_3	CP059853	2244553	2245701	Glutamicibacter nicotianae	3.40E-19	0.84301075

CDS_3	CP073075	1883334	1884584	Gordonia polyisoprenivorar	4.53E-19	0.92258065
CDS_3	AP017457	1130964	1132232	Aurantimicrobium minutum	5.05E-19	0.9311828
CDS_3	FO203507	3436760	3438046	Mycobacterium canettii CIP	1.89E-18	0.92473118
CDS_3	CP025332	3457879	3459156	Brevibacterium aurantiacun	2.52E-18	0.97634409
CDS_3	CP065373	5352447	5353709	Mycolicibacterium mengxia	2.79E-18	0.9139785
CDS_3	CP065373	1663534	1664793	Mycolicibacterium mengxia	1.69E-12	0.92903226
CDS_3	CP017150	3163675	3164952	Brevibacterium aurantiacun	2.84E-18	0.97634409
CDS_3	CP017150	2120009	2121286	Brevibacterium aurantiacun	3.81E-18	0.97634409
CDS_3	CP017150	3647531	3648808	Brevibacterium aurantiacun	4.21E-18	0.97634409
CDS_3	CP089608	162784	164049	Mycolicibacterium fortuitur	4.60E-18	0.9139785
CDS_3	CP089608	3083098	3084363	Mycolicibacterium fortuitur	1.59E-16	0.9139785
CDS_3	CP089608	5724048	5725319	Mycolicibacterium fortuitur	2.34E-16	0.91827957
CDS_3	CP104918	1929100	1930248	Glutamicibacter halophytoc	5.04E-18	0.84301075
CDS_4	CP012749	5904697	5905080	Rhodococcus sp. 008	1.19E-11	0.87969925
CDS_5	CP096563	3559449	3560321	Rhodococcus qingshengii JC	1.27E-88	0.95819936
CDS_5	AP025544	2120740	2121648	Rhodococcus hoagii U19 DN	8.43E-85	0.98392283
CDS_5	AP023172	3072256	3073128	Rhodococcus qingshengii CS	3.09E-77	0.94533762
CDS_5	CP117709	1549021	1549938	Streptomyces sp. MMBL 11-	1.82E-75	0.95498392
CDS_5	CP026652	7349757	7350665	Streptomyces dengpaensis s	2.22E-62	0.95498392
CDS_5	CP023689	1446432	1447322	Streptomyces chartreusis st	1.82E-61	0.92604502
CDS_5	CP021748	1008860	1009777	Streptomyces alboflavus str	2.00E-59	0.95498392
CDS_5	CP016793	667774	668703	Lentzea guizhouensis strain	6.10E-57	0.95176849
CDS_5	CP050692	7081972	7082871	Streptomyces antibioticus s	7.83E-52	0.91961415
CDS_5	CP060131	5433944	5434876	Pseudonocardia petroleoph	4.51E-47	0.95498392
CDS_5	CP066831	9480793	9481698	Streptomyces liliifuscus stra	7.86E-47	0.92604502
CDS_5	CP054925	2926486	2927376	Amycolatopsis sp. Hca4 chr	2.42E-41	0.91318328
CDS_5	CP015163	7819504	7820397	Amycolatopsis albispora str	1.18E-36	0.91961415
CDS_6	CP017299	3042791	3043147	Rhodococcus sp. YL-1	2.59E-17	0.86713287
CDS_6	CP096563	3554665	3555024	Rhodococcus qingshengii JC	1.15E-14	0.86713287
CDS_6	AP025544	2122183	2122518	Rhodococcus hoagii U19 DN	2.40E-14	0.81818182
CDS_6	CP012749	5901996	5902355	Rhodococcus sp. 008	1.00E-13	0.86713287
CDS_6	AP023172	3073642	3073998	Rhodococcus qingshengii CS	2.67E-13	0.86713287
CDS_7	CP017299	3043207	3043572	Rhodococcus sp. YL-1	2.52E-13	0.86029412
CDS_7	AP023172	3074037	3074396	Rhodococcus qingshengii CS	6.10E-11	0.88970588
CDS_8	AP011115	7604537	7605262	Rhodococcus opacus B4 DN	1.56E-79	0.96414343
CDS_8	CP096563	3078331	3079056	Rhodococcus qingshengii JC	2.22E-73	0.96414343

CDS_8	CP017299	3040507	3041232	Rhodococcus sp. YL-1	3.03E-73	0.96414343
CDS_8	AP023172	3037435	3038160	Rhodococcus qingshengii CS	2.21E-72	0.96414343
CDS_8	CP014941	3123515	3124153	Rhodococcus sp. BH4	2.09E-65	0.84860558
CDS_8	AP025544	2111923	2112636	Rhodococcus hoagii U19 DN	4.02E-30	0.89243028
CDS_8	CP049748	3262945	3263694	Rhodococcus fascians A21d	1.22E-25	0.9561753
CDS_8	CP025959	2268286	2268972	Rhodococcus sp. djl-6-2 chr	2.01E-25	0.88446215
CDS_10	CP117866	6922794	6923216	Streptomyces sp. T12 chrom	9.07E-17	0.98611111
CDS_10	CP020563	5264167	5264520	Kitasatospora albolonga stra	5.75E-13	0.86111111
CDS_10	CP027022	4380874	4381227	Streptomyces sp. WAC0028	6.46E-13	0.86111111
CDS_10	CP023693	3224382	3224735	Streptomyces cinereoruber	6.46E-13	0.86111111
CDS_10	CP018627	8222454	8222816	Streptomyces hygroscopicu	6.84E-13	0.96527778
CDS_10	CP077658	4045276	4045629	Streptomyces sp. GMY02 ch	3.31E-12	0.86111111
CDS_10	CP077658	5255324	5255677	Streptomyces sp. GMY02 ch	2.69E-11	0.86111111
CDS_10	CP077658	4019190	4019543	Streptomyces sp. GMY02 ch	6.06E-11	0.86111111
CDS_10	CP077658	5449093	5449452	Streptomyces sp. GMY02 ch	6.12E-11	0.86111111
CDS_10	CP073778	3432760	3433113	Streptomyces lavendulae su	4.38E-12	0.86111111
CDS_10	CP024985	3851159	3851512	Streptomyces lavendulae su	4.38E-12	0.86111111
CDS_10	CP101750	3732305	3732637	Streptomyces sp. Je 1-369 c	1.51E-11	0.79861111
CDS_10	CP022545	7386220	7386582	Streptomyces sp. 11-1-2	1.88E-11	0.96527778
CDS_10	CP036534	3550292	3550648	Streptomyces sp. VN1 chror	1.98E-11	0.80555556
CDS_10	CP016795	3499586	3499942	Streptomyces olivaceus stra	2.40E-11	0.80555556
CDS_10	CP094676	8036667	8037023	Streptomyces sp. CB09030 c	3.08E-11	0.80555556
CDS_10	CP101137	1476407	1476805	Streptomyces sp. M92 chror	5.05E-11	0.95138889
CDS_11	CP040244	3633857	3634126	Streptomyces exfoliatus stra	6.69E-28	0.96629214
CDS_11	CP086322	3929205	3929408	Streptomyces sp. ST13-2-2 c	9.32E-28	0.76404494
CDS_11	CP034587	2211697	2211978	Streptomyces luteoverticilla	3.04E-22	0.94382023
CDS_11	CP023690	1776541	1776753	Streptomyces spectabilis str	4.75E-22	0.79775281
CDS_11	CP019724	3775993	3776217	Streptomyces pactum strair	4.99E-22	0.84269663
CDS_11	CP066801	2130778	2130993	Streptomyces sp. HSG2 chro	1.17E-21	0.80898876
CDS_11	CP066801	2601769	2602047	Streptomyces sp. HSG2 chro	3.16E-18	0.92134832
CDS_11	CP026652	3716420	3716656	Streptomyces dengpaensis s	6.19E-21	0.92134832
CDS_11	CP096907	5606035	5606250	Streptomyces sp. LRE541 ch	1.35E-20	0.80898876
CDS_11	CP096907	4718303	4718530	Streptomyces sp. LRE541 ch	1.09E-16	0.85393258
CDS_11	CP101137	2422288	2422527	Streptomyces sp. M92 chror	1.52E-20	0.93258427
CDS_11	CP045096	6959559	6959768	Streptomyces phaeolivaceu	1.75E-20	0.78651685
CDS_11	CP045096	3559484	3559687	Streptomyces phaeolivaceu	1.06E-11	0.76404494

CDS_11	CP045096	5341819	5342034	Streptomyces phaeolivaceu	2.04E-11	0.80898876
CDS_11	CP016795	4648760	4648999	Streptomyces olivaceus stra	2.46E-20	0.93258427
CDS_11	CP016795	3620746	3620949	Streptomyces olivaceus stra	1.02E-11	0.76404494
CDS_11	CP114283	641771	641980	Streptomyces aurantiacus s	2.54E-20	0.78651685
CDS_11	CP036534	4605704	4605943	Streptomyces sp. VN1 chror	2.56E-20	0.93258427
CDS_11	CP113836	1098215	1098499	Amycolatopsis sp. HUAS 11-	2.85E-20	0.95505618
CDS_11	CP113836	5488714	5488944	Amycolatopsis sp. HUAS 11-	6.65E-20	0.86516854
CDS_11	CP113836	6022721	6022930	Amycolatopsis sp. HUAS 11-	1.08E-14	0.78651685
CDS_11	CP113836	6463445	6463675	Amycolatopsis sp. HUAS 11-	1.63E-12	0.87640449
CDS_11	CP113836	5543802	5544056	Amycolatopsis sp. HUAS 11-	4.31E-11	0.94382023
CDS_11	CP086120	3810849	3811082	Streptomyces gobiensis stra	2.91E-20	0.85393258
CDS_11	CP086120	3492248	3492448	Streptomyces gobiensis stra	1.84E-17	0.75280899
CDS_11	CP072827	5087386	5087625	Streptomyces mobaraensis	7.19E-20	0.91011236
CDS_11	CP071872	4970116	4970328	Streptomyces formicae stra	9.62E-20	0.79775281
CDS_11	CP040752	4134483	4134689	Streptomyces rectiverticillat	1.51E-19	0.7752809
CDS_11	CP102512	3620907	3621122	Streptomyces sp. CA-21006.	3.09E-19	0.80898876
CDS_11	CP001738	5519316	5519537	Thermomonospora curvata	5.38E-19	0.85393258
CDS_11	CP001738	5520211	5520438	Thermomonospora curvata	2.36E-16	0.85393258
CDS_11	CP094262	2465138	2465341	Streptomyces mobaraensis	1.05E-18	0.76404494
CDS_11	CP083590	2458368	2458571	Streptomyces mobaraensis	1.05E-18	0.76404494
CDS_11	CP099837	7478928	7479158	Nocardiopsis exhalans strair	1.87E-18	0.88764045
CDS_11	CP099837	6055846	6056055	Nocardiopsis exhalans strair	7.77E-17	0.78651685
CDS_11	FN554889	5454543	5454755	Streptomyces scabiei 87.22	9.31E-16	0.79775281
CDS_11	FN554889	5454866	5455093	Streptomyces scabiei 87.22	3.38E-11	0.85393258
CDS_11	CP080647	5407063	5407275	Streptomyces akebiae strair	1.99E-17	0.79775281
CDS_11	CP032427	7071720	7071932	Streptomyces griseorubigin	2.77E-17	0.79775281
CDS_11	CP088911	3888857	3889069	Yinghuangia sp. ASG 101 ch	9.35E-17	0.7752809
CDS_11	CP028834	4339701	4339928	Streptomyces sp. M2 chrom	9.82E-17	0.85393258
CDS_11	CP029254	5697745	5697975	Streptomyces spongiicola st	1.35E-16	0.86516854
CDS_11	CP107567	3260255	3260482	Streptomyces peucetius stra	1.47E-15	0.85393258
CDS_11	CP023694	1544369	1544569	Streptomyces coeruleorubic	1.62E-15	0.75280899
CDS_11	CP116256	2872704	2872904	Streptomyces rubrogriseus :	8.53E-14	0.75280899
CDS_11	CP050693	6784867	6785118	Streptomyces sp. 891-h chr	1.86E-15	0.84269663
CDS_11	CP009110	6922083	6922298	Amycolatopsis methanolica	5.53E-15	0.80898876
CDS_11	CP009110	362889	363092	Amycolatopsis methanolica	5.98E-13	0.76404494
CDS_11	CP009110	5852187	5852396	Amycolatopsis methanolica	2.74E-12	0.78651685

CDS_11	CP009110	3261426	3261626	Amycolatopsis methanolica	1.61E-11	0.75280899
CDS_19	AP025544	2116365	2116724	Rhodococcus hoagii U19 DN	6.83E-20	0.77564103
CDS_20_trur	AP025544	2116802	2117545	Rhodococcus hoagii U19 DN	4.60E-98	0.80063291
CDS_20_trur	CP012749	5907792	5908538	Rhodococcus sp. 008	7.62E-95	0.80063291
CDS_20_trur	CP096563	3567894	3568640	Rhodococcus qingshengii JC	1.34E-92	0.80063291
CDS_20_trur	CP016793	655194	655964	Lentzea guizhouensis strain	4.98E-76	0.79746835
CDS_20_trur	CP060131	5427318	5428037	Pseudonocardia petroleoph	1.55E-70	0.75316456
CDS_20_trur	CP023689	1451608	1452330	Streptomyces chartreusis st	4.39E-68	0.76582279
CDS_20_trur	CP021748	1014081	1014812	Streptomyces alboflavus str	1.99E-67	0.76898734
CDS_20_trur	CP015163	7814550	7815272	Amycolatopsis albispora str	3.01E-67	0.75632911
CDS_20_trur	CP026652	7344653	7345399	Streptomyces dengpaensis s	4.32E-64	0.7721519
CDS_20_trur	CP054925	2931601	2932335	Amycolatopsis sp. Hca4 chr	1.11E-58	0.76582279
CDS_20_trur	CP117709	1552935	1553666	Streptomyces sp. MMBL 11-	1.57E-58	0.76898734
CDS_20_trur	CP049016	1852949	1853647	Nitrospirales bacterium LBB	6.83E-20	0.76898734
CDS_20_trur	CP027287	355122	355802	Campylobacter fetus subsp.	4.55E-15	0.76898734
CDS_20_trur	CP040464	240942	241607	Campylobacter hyointestina	9.73E-15	0.75316456
CDS_20_trur	CP002221	824725	825402	Hydrogenobacter thermoph	1.30E-13	0.75949367
CDS_20_trur	AP011112	824734	825411	Hydrogenobacter thermoph	1.30E-13	0.75949367

Supplementary Table 10. Genome accession numbers of the phages from the BE cluster and their corresponding hosts.

Phage_name	Phage_genome_accession	Host_name	Host_genome_accession
Angela	ON970591	Streptomyces griseus ATCC 10137	CP032543.1
Bartholomune	OK310502	Streptomyces lividans JI 1326	CP009124.1
Birchlyn	MK801722	Streptomyces griseus ATCC 10137	CP032543.1
Bmoc	MT310865	Streptomyces griseofuscus ATCC 23916	CP051006.1
BoomerJR	MK359351	Streptomyces lividans JI 1326	CP009124.1
Bordeaux	MN369757	Streptomyces griseus ATCC 10137	CP032543.1
Braelyn	MN096371	Streptomyces griseus ATCC 10137	CP032543.1
Daubenski	MN444876	Streptomyces griseus ATCC 10137	CP032543.1
EGole	MK494112	Streptomyces griseus ATCC 10137	CP032543.1
Evy	MK977711	Streptomyces griseus ATCC 10137	CP032543.1
Genie2	MK359332	Streptomyces lividans JI 1326	CP009124.1
IchabodCrane	MN428060	Streptomyces scabiei RL-34	FN554889.1
Jay2Jay	KM652554	Streptomyces lividans JI 1326	CP009124.1
JimJam	ON970590	Streptomyces griseus ATCC 10137	CP032543.1
Karimac	MH590599	Streptomyces griseus ATCC 10137	CP032543.1
LukeCage	MH590597	Streptomyces griseus ATCC 10137	CP032543.1
MindFlayer	MW291014	Streptomyces mirabilis NRRL B-2400	CP074102.1
MulchMansion	MT897905	Streptomyces lividans JI 1326	CP009124.1
NootNoot	MF347636	Streptomyces griseus ATCC 10137	CP032543.1
Paradiddles	MF347637	Streptomyces griseus ATCC 10137	CP032543.1
Peebs	MF347638	Streptomyces lividans JI 1326	CP009124.1
Pepperwood	OQ190480	Streptomyces lividans JI 1326	CP009124.1
Quaran19	ON260828	Streptomyces griseus ATCC 10137	CP032543.1
SaltySpitoon	ON260819	Streptomyces griseus ATCC 10137	CP032543.1
Samisti12	MF347639	Streptomyces griseus ATCC 10137	CP032543.1
Spilled	ON970579	Streptomyces griseus ATCC 10137	CP032543.1
Squillium	ON108650	Streptomyces lividans JI 1326	CP009124.1
Stanimal	OP021680	Streptomyces lividans JI 1326	CP009124.1
Starbow	MH576964	Streptomyces griseus ATCC 10137	CP032543.1
StarPlatinum	MH576965	Streptomyces griseus ATCC 10137	CP032543.1
Sushi23	MF358542	Streptomyces griseus ATCC 10137	CP032543.1
Targaryen	MZ958750	Streptomyces lividans JI 1326	CP009124.1
Teutsch	MK460248	Streptomyces griseus ATCC 10137	CP032543.1

TomSawyer	MN369750	Streptomyces griseus ATCC 10137	CP032543.1
Tribute	MN369743	Streptomyces griseus ATCC 10137	CP032543.1
Warpy	MF358541	Streptomyces griseus ATCC 10137	CP032543.1
Wipeout	MN484599	Streptomyces griseus ATCC 10137	CP032543.1
Wofford	MH576968	Streptomyces griseus ATCC 10137	CP032543.1
Yaboi	MH727564	Streptomyces lividans JI 1326	CP009124.1

Supplementary Table 11. Inferred gene copy numbers for tRNA genes (grouped by anticodon) for the phages belonging to the BE cluster.

Name	Accession_nu	ttt	ttc	ttg	tct	tcc	tcg	tat	tac	taa	tag	tgt	tgc	tga	tgg	ctt	ctc	ctg	cct	ccc	cca	ccg	cat	cac	caa	cag
Angela	ON970591	1	1	1	0	1	1	1	1	0	1	1	1	1	1	1	1	1	1	0	1	0	1	0	1	0
Bartholomune	OK310502	1	1	1	1	1	1	1	1	0	1	1	1	1	1	1	1	1	1	0	1	0	1	0	1	0
Birchlyn	MK801722	1	1	1	1	1	1	1	1	1	1	1	1	1	1	1	1	1	1	0	1	0	1	0	1	0
Bmoc	MT310865	1	1	1	0	1	1	1	1	0	1	1	1	1	1	1	1	1	1	0	1	0	1	0	1	0
BoomerJR	MK359351	1	1	1	0	1	1	1	1	1	1	1	1	1	1	1	1	1	1	0	1	0	1	0	1	0
Bordeaux	MN369757	1	1	1	1	1	1	1	1	1	1	1	1	1	1	1	1	1	1	0	1	0	1	0	1	0
Braelyn	MN096371	1	1	1	1	1	0	1	1	0	1	1	1	1	1	1	1	1	1	0	1	0	1	0	1	0
Daubenski	MN444876	1	1	1	0	1	1	1	1	0	1	1	1	0	1	1	1	1	1	0	1	0	1	0	1	0
EGole	MK494112	1	1	1	1	1	1	1	1	0	1	1	1	1	1	1	1	1	1	0	1	0	1	0	1	0
Evy	MK977711	1	1	1	1	1	1	1	1	0	1	1	1	0	1	1	1	1	1	0	1	0	1	0	1	0
Genie2	MK359332	1	1	1	0	1	1	1	1	1	1	1	1	1	1	1	1	1	1	0	1	0	1	0	1	0
IchabodCrane	MN428060	1	1	1	1	1	1	1	1	1	1	1	1	1	1	1	1	1	1	0	1	0	1	0	1	0
Jay2Jay	KM652554	1	1	1	1	1	1	1	1	0	1	1	1	0	1	1	1	1	1	0	1	0	1	0	1	0
JimJam	ON970590	1	1	1	1	1	1	1	1	1	1	1	1	1	1	1	1	1	0	0	1	0	1	0	1	0
Karimac	MH590599	1	1	1	1	1	1	1	1	1	1	1	1	1	1	1	1	1	1	0	1	0	1	0	1	0
LukeCage	MH590597	1	1	1	1	1	1	1	1	0	1	1	1	1	1	1	1	1	1	0	1	0	1	0	1	0
MindFlayer	MW291014	1	1	1	1	1	1	1	1	1	1	1	1	1	1	1	1	1	1	0	1	0	1	0	1	0
MulchMansion	MT897905	1	1	1	0	1	1	1	1	0	1	1	1	1	1	1	1	1	1	0	1	0	1	0	1	0
NootNoot	MF347636	1	1	1	1	1	1	1	1	0	1	1	1	1	1	1	1	1	1	0	1	0	1	0	1	0
Paradiddles	MF347637	1	1	1	1	1	1	1	1	0	1	1	1	1	1	1	1	1	1	0	1	0	1	0	1	0
Peebs	MF347638	1	1	1	1	1	1	1	1	0	1	1	1	1	1	1	1	1	1	0	1	0	1	0	1	0
Pepperwood	OQ190480	1	1	1	1	1	1	1	1	0	1	1	1	1	1	1	1	1	1	0	1	0	1	0	1	0
Quaran19	ON260828	1	1	1	1	1	1	1	1	1	1	1	1	1	1	1	1	1	0	0	1	0	1	0	1	0
SaltySpitoon	ON260819	1	1	1	1	1	1	1	1	1	1	1	1	1	1	1	1	1	1	0	1	0	1	0	1	0
Samisti12	MF347639	1	1	1	1	1	1	1	1	0	1	1	1	1	1	1	1	1	1	0	1	0	1	0	1	0
Spilled	ON970579	1	1	1	1	1	1	1	1	1	1	1	1	1	1	1	1	1	1	0	1	0	1	0	1	0
Squillium	ON108650	1	1	1	1	1	1	1	1	0	1	1	1	1	1	1	1	1	1	0	1	0	1	0	1	0
Stanimal	OP021680	1	1	1	0	1	1	1	1	1	1	1	1	1	1	1	1	1	0	0	1	0	1	0	1	0
Starbow	MH576964	1	1	1	1	1	1	1	1	1	1	1	1	1	1	1	1	1	1	0	1	0	1	0	1	0
StarPlatinum	MH576965	1	1	1	1	1	1	1	1	0	1	1	1	1	1	1	1	1	1	0	1	0	1	0	1	0
Sushi23	MF358542	1	1	1	1	1	1	1	1	0	1	1	1	1	1	1	1	1	1	0	1	0	1	0	1	0
Targaryen	MZ958750	1	1	1	1	1	1	1	1	0	1	1	1	0	1	1	1	1	1	0	1	0	1	0	1	0
Teutsch	MK460248	1	1	1	1	1	1	1	1	0	1	1	1	1	1	1	1	1	1	0	1	0	1	0	1	0

TomSawyer	MN369750	1	1	1	1	1	1	1	1	1	1	1	1	1	1	1	1	1	0	0	1	0	1	0	1	0
Tribute	MN369743	1	1	1	1	1	1	1	1	0	1	1	1	1	1	1	1	1	1	0	1	0	1	0	1	0
Warpy	MF358541	1	1	1	1	1	1	1	1	0	1	1	1	0	1	1	1	1	1	0	1	0	1	0	1	0
Wipeout	MN484599	1	1	1	1	1	1	1	1	1	1	1	1	1	1	1	1	1	1	0	1	0	1	0	1	0
Wofford	MH576968	1	1	1	1	1	1	1	1	1	1	1	1	1	1	1	1	1	1	0	1	0	1	0	1	0
Yaboi	MH727564	1	1	1	0	1	1	1	1	1	1	1	1	1	1	1	1	1	0	0	1	0	1	0	1	0

cgt	cgc	cga	cgg	att	atc	ata	atg	act	acc	aca	acg	aat	aac	aaa	aag	agt	agc	aga	agg	gtt	gtc	gta	gtg	gct	gcc	gca	gcg	gat	gac	gaa	gag
1	0	0	0	0	0	0	0	0	0	0	1	0	0	0	0	0	0	0	0	1	1	0	1	1	1	1	0	1	1	1	1
1	0	0	0	0	0	0	0	0	0	0	1	0	0	0	0	0	0	0	0	1	1	1	1	1	1	1	0	1	1	1	1
1	0	0	0	0	0	0	0	0	0	0	1	0	0	0	0	0	0	0	0	1	1	1	1	0	1	1	0	0	1	1	1
1	0	0	0	0	0	0	0	0	0	0	1	0	0	0	0	0	0	0	0	1	1	1	1	1	1	1	0	1	1	0	1
1	0	0	0	0	0	0	0	0	0	0	1	0	0	0	0	0	0	0	0	1	1	1	1	1	1	1	0	0	1	1	1
1	0	0	0	0	0	0	0	0	0	0	1	0	0	0	0	0	0	0	0	1	1	1	1	1	1	1	0	0	1	1	1
1	0	0	0	0	0	0	0	0	0	0	0	0	0	0	0	0	0	0	0	1	1	1	1	1	1	1	0	1	1	1	1
1	0	0	0	0	0	0	0	0	0	0	1	0	0	0	0	0	0	0	0	1	1	0	1	1	1	1	0	0	1	1	1
1	0	0	0	0	0	0	0	0	0	0	1	0	0	0	0	0	0	0	0	1	1	1	1	1	1	1	0	1	1	1	1
1	0	0	0	0	0	0	0	0	0	0	1	0	0	0	0	0	0	0	0	1	1	1	1	1	1	1	0	1	1	1	1
1	0	0	0	0	0	0	0	0	0	0	1	0	0	0	0	0	0	0	0	1	1	1	1	1	1	1	0	0	1	1	1
1	0	0	0	0	0	0	0	0	0	0	1	0	0	0	0	0	0	0	0	1	1	1	1	0	1	1	0	0	1	1	1
1	0	0	0	0	0	0	0	0	0	0	1	0	0	0	0	0	0	0	0	1	1	1	1	1	1	1	0	1	1	1	1
1	0	0	0	0	0	0	0	0	0	0	1	0	0	0	0	0	0	0	0	1	1	1	1	0	1	1	0	0	1	1	1
1	0	0	0	0	0	0	0	0	0	0	1	0	0	0	0	0	0	0	0	1	1	1	1	0	1	1	0	0	1	1	1
1	0	0	0	0	0	0	0	0	0	0	1	0	0	0	0	0	0	0	0	1	1	1	1	0	1	1	0	0	1	1	1
1	0	0	0	0	0	0	0	0	0	0	1	0	0	0	0	0	0	0	0	1	1	1	1	0	1	1	0	0	1	1	1
1	0	0	0	0	0	0	0	0	0	0	1	0	0	0	0	0	0	0	0	1	1	0	1	1	1	1	0	1	1	1	1
1	0	0	0	0	0	0	0	0	0	0	1	0	0	0	0	0	0	0	0	1	1	1	1	1	1	1	0	1	1	1	1
1	0	0	0	0	0	0	0	0	0	0	1	0	0	0	0	0	0	0	0	1	1	1	1	1	1	1	0	1	1	1	1
1	0	0	0	0	0	0	0	0	0	0	1	0	0	0	0	0	0	0	0	1	1	1	1	1	1	1	0	1	1	1	1
1	0	0	0	0	0	0	0	0	0	0	1	0	0	0	0	0	0	0	0	1	1	1	1	1	1	1	0	1	1	1	1
1	0	0	0	0	0	0	0	0	0	0	1	0	0	0	0	0	0	0	0	1	1	1	1	0	1	1	0	0	1	1	1
1	0	0	0	0	0	0	0	0	0	0	1	0	0	0	0	0	0	0	0	1	1	1	1	0	1	1	0	0	1	1	1
1	0	0	0	0	0	0	0	0	0	0	1	0	0	0	0	0	0	0	0	1	1	1	1	1	1	1	0	1	1	1	1
1	0	0	0	0	0	0	0	0	0	0	1	0	0	0	0	0	0	0	0	1	1	1	1	0	1	1	0	0	1	1	1
1	0	0	0	0	0	0	0	0	0	0	1	0	0	0	0	0	0	0	0	1	1	1	1	1	1	1	0	1	1	1	1
1	0	0	0	0	0	0	0	0	0	0	1	0	0	0	0	0	0	0	0	1	1	1	1	1	1	1	0	1	1	1	1
1	0	0	0	0	0	0	0	0	0	0	1	0	0	0	0	0	0	0	0	1	1	1	1	0	1	1	0	0	1	1	1
1	0	0	0	0	0	0	0	0	0	0	1	0	0	0	0	0	0	0	0	1	1	1	1	0	1	1	0	0	1	1	1
1	0	0	0	0	0	0	0	0	0	0	1	0	0	0	0	0	0	0	0	1	1	1	1	1	1	1	0	1	1	1	1
1	0	0	0	0	0	0	0	0	0	0	1	0	0	0	0	0	0	0	0	1	1	1	1	1	1	1	0	1	1	1	1
1	0	0	0	0	0	0	0	0	0	0	1	0	0	0	0	0	0	0	0	1	1	1	1	1	1	1	0	1	1	1	1

1	0	0	0	0	0	0	0	0	0	0	1	0	0	0	0	0	0	0	0	1	1	1	1	0	1	1	0	1	1	1	1
1	0	0	0	0	0	0	0	0	0	0	1	0	0	0	0	0	0	0	0	1	1	1	1	1	1	1	0	1	1	1	1
1	0	0	0	0	0	0	0	0	0	0	1	0	0	0	0	0	0	0	0	1	1	1	0	1	1	1	0	1	1	1	1
1	0	0	0	0	0	0	0	0	0	0	1	0	0	0	0	0	0	0	0	1	1	1	1	0	1	1	0	0	1	1	1
1	0	0	0	0	0	0	0	0	0	0	1	0	0	0	0	0	0	0	0	1	1	1	1	1	1	1	0	0	1	1	1
1	0	0	0	0	0	0	0	0	0	0	1	0	0	0	0	0	0	0	0	1	1	1	1	1	1	1	0	1	1	1	1

ggt	ggc	gga	ggg
1	1	1	0
1	1	1	0
1	1	1	0
1	1	1	0
1	1	1	0
1	1	1	0
1	1	1	0
1	1	1	0
1	1	1	0
1	1	1	0
1	1	1	0
1	1	1	0
1	1	1	0
1	1	1	0
1	1	1	0
1	1	1	0
1	1	1	0
1	1	1	0
1	1	1	0
1	1	1	0
1	1	1	0
1	1	1	0
1	1	1	0
1	1	1	0
1	1	1	0
1	1	1	0
1	1	1	0
1	1	1	0
1	1	1	0
1	1	1	0
1	1	1	0
1	1	1	0
1	1	1	0

1	1	1	0
1	1	1	0
1	1	1	0
1	1	1	0
1	1	1	0
1	1	1	0

Supplementary Table 12. Inferred gene copy numbers for tRNA genes (grouped by anticodon) for the hosts of the phages belonging to the BE cluster.

**Name**	**Accession_**	**ttt**	**ttc**	**ttg**	**tct**	**tcc**	**tcg**	**tat**	**tac**	**taa**	**tag**	**tgt**	**tgc**	**tga**	**tgg**	**ctt**	**ctc**	**ctg**	**cct**	**ccc**	**cca**	**ccg**
Streptomyces griseus ATCC 10137	CP032543	1	1	1	1	1	0	0	1	1	1	1	1	1	1	1	1	1	1	1	1	1
Streptomyces lividans JI 1326	CP009124	1	1	1	1	1	0	0	1	1	1	1	1	1	1	1	1	1	1	1	1	1
Streptomyces griseofuscus ATCC 23916	CP051006	1	1	1	1	1	1	0	1	1	1	1	1	1	1	1	1	1	1	1	1	1
Streptomyces scabiei RL-34	FN554889	1	1	1	1	1	0	1	1	1	1	1	1	1	1	1	1	1	1	1	1	1
Streptomyces mirabilis NRRL B-2400	CP074102	1	1	1	1	1	0	0	1	1	1	1	1	1	1	1	1	1	1	1	1	1

cat	cac	caa	cag	cgt	cgc	cga	cgg	att	atc	ata	atg	act	acc	aca	acg	aat	aac	aaa	aag	agt	agc	aga	agg	gtt	gtc	gta	gtg	gct	gcc	gca	gcg	gat	gac
1	1	1	1	1	1	1	1	0	0	0	0	0	0	0	1	0	0	0	0	0	0	0	0	1	1	1	1	0	1	1	1	1	1
1	1	1	1	1	1	1	1	0	0	0	0	0	1	0	1	0	0	0	0	0	0	0	0	1	1	1	1	0	1	1	0	1	1
1	1	1	1	1	1	1	1	0	0	0	0	0	0	0	1	0	0	0	0	0	0	0	0	1	1	1	1	0	1	1	1	1	1
1	1	1	1	1	1	1	1	0	0	0	0	0	0	0	1	0	0	0	0	0	0	0	0	1	1	1	1	1	1	1	1	1	1
1	1	1	1	1	1	1	1	0	0	0	0	0	0	0	1	0	0	0	0	0	0	0	0	1	1	1	1	1	1	1	1	1	1

gaa	gag	ggt	ggc	gga	ggg
1	1	1	1	1	1
1	1	1	1	1	1
1	1	1	1	1	1
1	1	1	1	1	1
1	1	1	1	1	1
